# Addressing chemically-induced obesogenic metabolic disruption: selection of chemicals for *in vitro* human PPARα, PPARγ transactivation, and adipogenesis test methods

**DOI:** 10.3389/fendo.2024.1401120

**Published:** 2024-07-08

**Authors:** Eren Ozcagli, Barbara Kubickova, Miriam N. Jacobs

**Affiliations:** Radiation, Chemical and Environmental Hazards (RCE), Department of Toxicology, UK Health Security Agency (UKHSA), Harwell Science and Innovation Campus, Chilton, Oxfordshire, United Kingdom

**Keywords:** adipogenesis, obesogen, peroxisome proliferator-activated receptor, metabolic disruption, integrated testing strategy, test guideline, validation

## Abstract

Whilst western diet and sedentary lifestyles heavily contribute to the global obesity epidemic, it is likely that chemical exposure may also contribute. A substantial body of literature implicates a variety of suspected environmental chemicals in metabolic disruption and obesogenic mechanisms. Chemically induced obesogenic metabolic disruption is not yet considered in regulatory testing paradigms or regulations, but this is an internationally recognised human health regulatory development need. An early step in the development of relevant regulatory test methods is to derive appropriate minimum chemical selection lists for the target endpoint and its key mechanisms, such that the test method can be suitably optimised and validated. Independently collated and reviewed reference and proficiency chemicals relevant for the regulatory chemical universe that they are intended to serve, assist regulatory test method development and validation, particularly in relation to the OECD Test Guidelines Programme. To address obesogenic mechanisms and modes of action for chemical hazard assessment, key initiating mechanisms include molecular-level Peroxisome Proliferator-Activated Receptor (PPAR) α and γ agonism and the tissue/organ-level key event of perturbation of the adipogenesis process that may lead to excess white adipose tissue. Here we present a critical literature review, analysis and evaluation of chemicals suitable for the development, optimisation and validation of human PPARα and PPARγ agonism and human white adipose tissue adipogenesis test methods. The chemical lists have been derived with consideration of essential criteria needed for understanding the strengths and limitations of the test methods. With a weight of evidence approach, this has been combined with practical and applied aspects required for the integration and combination of relevant candidate test methods into test batteries, as part of an Integrated Approach to Testing and Assessment for metabolic disruption. The proposed proficiency and reference chemical list includes a long list of negatives and positives (20 chemicals for PPARα, 21 for PPARγ, and 11 for adipogenesis) from which a (pre-)validation proficiency chemicals list has been derived.

## 1 Introduction

The global incidence of obesity and metabolic disorders is growing dramatically (1, 2). Noncommunicable diseases, including metabolic syndrome, are estimated to account for 74% of all deaths globally (3). The increasing incidence of obesity, type 2 diabetes, insulin resistance and hypertension are commonly considered to be a consequence of lifestyle, particularly high dietary intakes of sugar, processed food, and *trans*-unsaturated fatty acids, large portion sizes, and decreased physical activity (4). An association with socioeconomic status, age, sex and ethnicity is often reported, but environmental and industrial contaminants are also considered to play a role in altering metabolism in humans and contributing to the epidemic of non-communicable diseases including metabolic syndrome. Metabolic disruption, in the context of energy metabolism, is a complex process involving multiple tissues, organs, and relevant molecular targets. This disruption can manifest in various forms, including dysregulated glucose metabolism, impaired lipid metabolism, altered hormonal signalling, and dysfunctional mitochondrial function (5).

Indeed, the roles of chemical and environmental factors are increasingly acknowledged (1, 2, 6, 7), in addition to the established nutrition and lifestyle causative factors (8). These factors, especially exposure to environmental chemicals, contribute to the complexity of metabolic disorders and highlight the need for a comprehensive understanding of their underlying mechanisms in order to develop regulatory relevant test methods to adequately assess the hazards of MDCs. At the molecular level, metabolic disruption may involve abnormal activity or expression of enzymes, receptors, transporters, and signalling molecules, all of which can be perturbed by chemical exposure.

The economic impact of chemically-induced metabolic disruption for the three groups of chemicals for which the weight of evidence of causing metabolic disruption in humans is strongest (*p,p’*-dichlorodiphenyldichloroethylene (*p,p’*-DDE), phthalates, bisphenol A (BPA)) is estimated to exceed €18 billion (with moderate probability) in Europe (6, 9). Developing appropriate human-relevant test methods to adequately assess these putative chemical hazards is needed for front-end public health protection, to reduce the contribution that chemical hazards may make towards metabolic disruption. This will also support greener more sustainable chemistry development, reducing potentially regrettable chemical substitutions.

To address the need for elucidating and identifying metabolic disruption chemicals, the European Commission has dedicated funding to the development and (pre-)validation of test methods and Integrated Approaches to Testing and Assessment (IATA) under the Horizon 2020 Research and Innovation framework, to three projects of the EURION cluster: EDCMET (10), GOLIATH (7), and OBERON (11). To this end, the EU-funded Horizon 2020 GOLIATH project (7) (https://beatinggoliath.eu/; https://cordis.europa.eu/project/id/825489) aims to address the lack of methods for testing EDCs that disrupt metabolism and metabolic functions. These chemicals collectively referred to as “metabolism disrupting compounds” (MDCs) are natural and anthropogenic chemicals that can promote metabolic changes that can ultimately contribute to the development of obesity, diabetes, and/or fatty liver in humans. The project has focused on the main cellular targets of metabolic disruption: Peroxisome proliferator activated receptor (PPAR) test methods, hepatocytes, pancreatic endocrine cells, myocytes and adipocytes-and using a mechanistic and mode of action or adverse outcome pathway (AOP) type framework, has generated key information on MDC-related modes of action (7). The project committed to take forward between three to five test methods towards pre-validation, depending upon the test method optimisation and test method readiness to enter the OECD Test Guideline Programme. To support *in vitro* test method development and validation, careful and relevant chemical selection needs to be conducted for each endpoint, and with clear understanding of the test methods, including their potential limitations.

Here we report on the selection of recommended reference chemicals in relation to human white adipose tissue adipogenesis, PPARα and PPARγ agonism, to support the (pre-)validation of these test methods within the GOLIATH project. Related chemical selection reviews previously published from the same project include the chemical selection for steatosis (12) and CYP induction chemical selection augmentation (13).

There are several types of fat tissue in the human body, each with its own characteristics and functions. The two main types of fat are white adipose tissue (WAT) and brown adipose tissue (BAT), also there is a transitional type known as beige or brite adipose tissue. Brown fat cells have a significant number of mitochondria, which play a central role in energy production, compared to white fat cells (14). Activation of brown fat may contribute to increased energy expenditure, which may play a role in preventing or managing obesity. Brown fat has the ability to burn calories to produce heat, and individuals with higher amounts of active brown fat may have an increased capability to resist weight gain. This is important in the context of preventing obesity-related metabolic diseases (15). However, the focus of this chemical selection review is specific to the mechanistic understanding in relation to an increase in WAT in particular *via* the well understood Molecular Initiating Events (MIEs) of PPARγ activation, but also the role PPARα. PPAR agonists are known to help alleviate the underlying metabolic dysregulation observed in metabolic syndrome and insulin resistance by targeting different aspects of metabolism. They improve lipid profile, enhance insulin sensitivity, reduce inflammation, and promote metabolic homeostasis (16). Although relevant to metabolic disruption, chemical selection regarding BAT will be conducted separately, and is not part of the scope of this paper.

Nutritionally, the PPARs evolved as a fatty acid-activated nuclear receptors, where long chain fatty acids are the key molecular targets, to effect regulation of signal transduction processes and gene regulation, which in turn dictate their roles in health and disease (17–19). Fatty acids are important players in the development of the pathology of cardiovascular and endocrine diseases, thus understanding of the roles of fatty acids is useful for identifying chemical characteristics that contribute to the potential to interact with the PPARs. PPARα activation results in biological mechanisms leading to fatty acid oxidation in the liver cells as a response e.g., to extended fasting and/or energy deprivation. PPARγ activation, on the other hand, stimulates storage of fatty acids, including in the adipose tissues (20). This has essentially been the basis for the development of pharmaceuticals such as the thiazolidinediones for PPARγ, and fibrates for PPARα (21, 22), to lower triglyceride or glucose levels.

In the literature, a number of chemicals have been implicated in relation to obesity, including bisphenols, pesticides, phthalates, metals, and perfluorinated compounds. Here we present a more thorough analysis of chemicals that may (or may not) be implicated in the mode of action of adipogenesis. From the MIE(s) of PPARα and PPARγ activation, the later key event (KE) of WAT adipogenesis stimulation, with the intention that it can be a basis for developing and optimizing human adipogenesis-relevant *in vitro* test methods that can be integrated together as a test method battery, as part of an IATA for metabolic disruption.

### 1.1 PPARs

The PPARs are a well understood family of orphan nuclear receptors within a family of transcription factors involved in gene regulation of key physiological processes, with fundamental roles in regulating energy balance. Three closely related receptors: PPARα, β/δ, and γ, are variously expressed in the liver, kidney, heart, hematopoietic and adipose tissue. With a central role in hepatogenesis, PPARα is expressed primarily in liver, but also kidney, heart and muscle. PPARδ is ubiquitous and has a fundamental role in embryo development, it allows normal cells to better cope with adverse nutrient and energy situations, it has a therapeutic role in burning fat, although if over-expressed it can promote inflammation and tumorigenesis (23). Selective ligands for PPARδ are lacking (24), and PPARδ is not the focus of this chemical selection objective.

A number of prevalent metabolic disorders such as obesity, atherosclerosis and type 2 diabetes mellitus are associated with a shift in this balance, so PPARα and PPARγ in particular, are of interest pharmacologically (25). Model agonists include xenobiotics that elicit increases in the number and size of peroxisomes when administered to rodents, and also can induce hepatocellular carcinoma development via a non-genotoxic mechanism (26).

PPARγ has a central regulatory role in adipogenesis, lipogenesis and lipid storage in the liver and adipose tissue, and adipokine production and is pivotal for whole body insulin sensitivity primarily in the muscle (25, 27). It is found in adipocytes, the large intestine, and monocyte lineage cells.

PPARα regulates key steps in lipid and fibrate metabolism, fatty acid oxidation and fasting response. It is the molecular target for naturally occurring plant fatty acids and peroxisome proliferators, including pharmaceuticals, phthalates and pesticides (28, 29). PPARγ ligands include fatty acids, prostaglandins and pharmaceuticals such as the group of antidiabetic drugs, thiazolidinediones (glitazones) (25, 30, 31). As with other ligand-activated nuclear receptors, the PPARs bind to their cognate DNA elements as heterodimers with the Retinoid X Receptor (RXR), and then activate the transcription of downstream genes, including e.g., Cytochrome P450 (CYP)-mediated metabolism CYP4A genes. This can lead to enhanced CYP metabolism of the ligand as reviewed previously (31), and activation of genes involved in the regulation of energy homeostasis (32).

Activation of PPARα induces the expression of CYP enzymes involved in the ω-oxidation pathway, such as CYP4A, which catalyzes the oxidation of fatty acids (29). PPARγ also regulates the expression of certain CYP enzymes, particularly in adipose tissue, with potential regulatory effects in the expression of Cyp2f2 (33).

**Figure 1:**
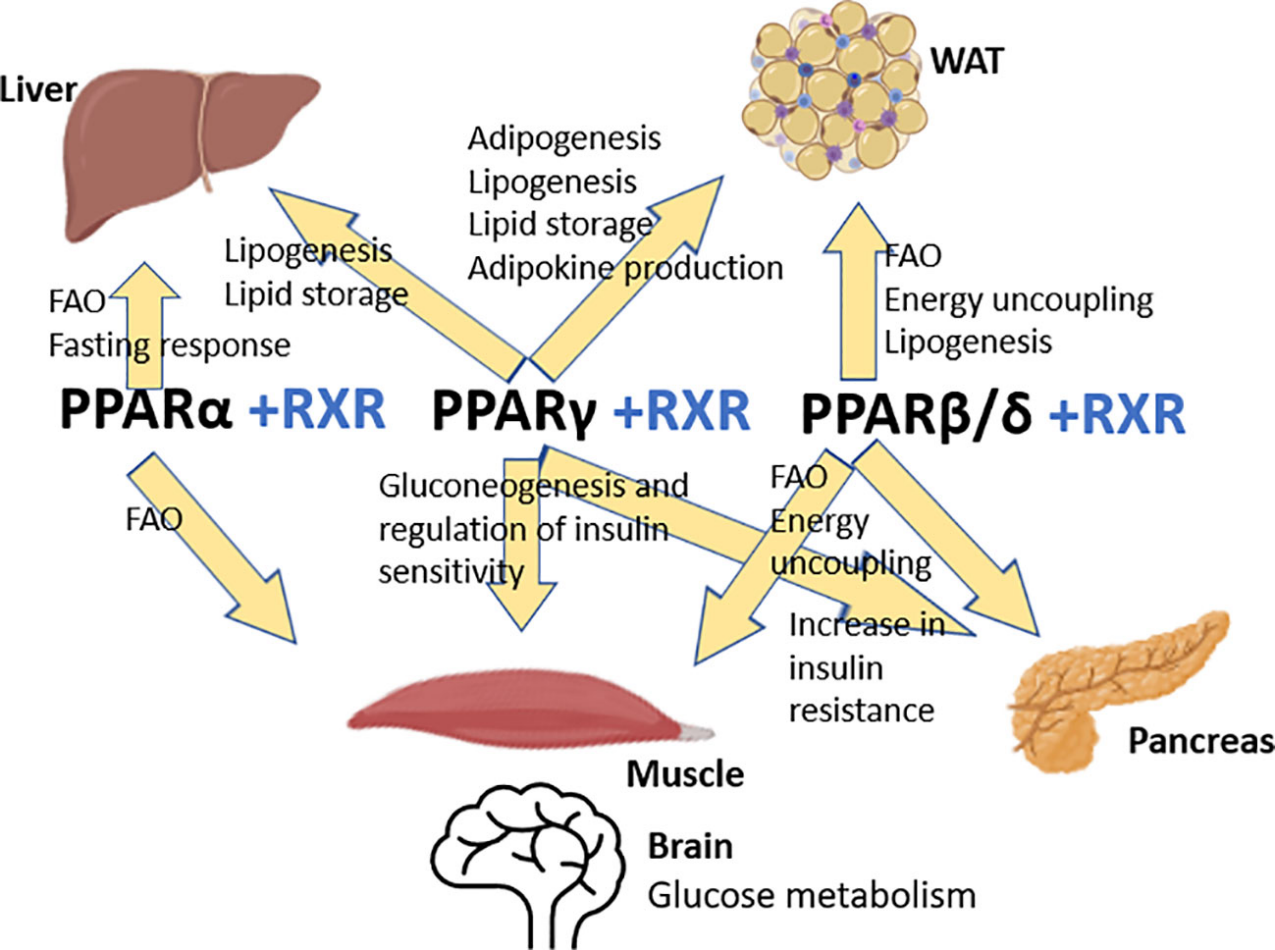
The role of the PPARs in fat and glucose metabolism (modified from Evans et al., 2004, organ illustrations from Biorender.com and Microsoft PowerPoint). FAO, Fatty acid oxidation; WAT, White adipose tissue.

As RXR is relevant for the PPAR MIE, the scope of the chemical selection review, took this into consideration. **Figure 1** gives an overview of the PPARs and their roles in adipogenesis.

### 1.2 Adipogenesis

Adipogenesis is the molecular and cellular process by which undifferentiated cells, typically mesenchymal stem cells, differentiate and develop into mature adipocytes (fat cells) capable of storing and releasing lipids (34). This process plays a fundamental role in the development and maintenance of adipose tissue, which is responsible for storing energy in the form of fat. It is also crucial for energy homoeostasis; adipocytes store excess energy, releasing it during times of need. If adipocytes become hypertrophic, owing e.g., to chronic energy excess, they also can become insulin-resistant. They lose their ability to appropriately respond to physiological levels of insulin and other mediators of energy metabolism/expenditure, such that energy expenditure is impaired, and lipid storage becomes dysfunctional, leading to excess accumulation of (visceral) body fat. Dysfunctional adipocytes are unable to respond adequately to physiological levels of insulin and other mediators of energy metabolism/expenditure. Fatty acids are released into the circulation and accumulate in other organs and are stored as body fat. The dysregulated secretion of endocrine factors from hypertrophic and dysfunctional adipocytes contributes to the development of systemic inflammation, insulin resistance, and metabolic disruption, leading to pathological changes in various organs and tissues implicated in obesity-related metabolic disorders (35).

Adipocytes originate from mesenchymal stem cells (MSCs), which are multipotent cells that can differentiate into various cell types, including adipocytes, osteoblasts, and chondrocytes through a complex and multi-step process comprising a network of transcription factors (36, 37). Adipogenic signalling pathways (e.g. Wnt, BMP or Hedgehog signalling) and transcription factors such as PPARγ, C/EBPs, sterol regulatory element-binding protein (SREBP), and glucocorticoid receptor (GR) are considered as master regulators of genes in adipogenesis (38) and their differential expression/activation determines the adipocytic phenotype (i.e., WAT, BAT, or beige adipocytes).

Adipocyte differentiation can be induced in cell culture by the stimulation of MSCs with isobutylmethylxanthine (IBMX), dexamethasone, and insulin, in particular for the murine 3T3-L1 cell line (39). Multipotent human MSCs are reported to require a PPARγ agonist to effectively promote differentiation (40). The 3T3-L1 cell line is a well-established model for studying adipogenesis and has been extensively used to elucidate the transcriptional cascade and molecular mechanisms underlying adipocyte differentiation. Greater details on the relative merits of the murine and human cell systems are reviewed in Kassotis et al., 2022 (41) and references therein. Stimulation by IBMX inhibits phosphodiesterases, leading to increased intracellular levels of cyclic AMP (cAMP) and activation of C/EBP-δ. Dexamethasone induces expression of C/EBP-α, C/EBP-β and -δ, in turn, these induce the expression of C/EBP-α and PPARγ2. Insulin stimulates both adipogenesis and lipogenesis through induction of SREBP-1c and other transcription factors, in addition to directly inhibiting lipolysis (41). Whilst GR is therefore highly relevant to PPARγ with respect to the promotion of cellular differentiation (42, 43), and therefore consideration of GR ligands is included in this chemical selection, chemical selection and pre-validation specifically focused upon the GR was not included in the original GOLIATH proposal, and is currently being addressed by the PEPPER platform (L’association Pepper (ed-pepper.eu)).

Adipogenesis can be assessed and quantified *in vitro* by measuring endpoints such as the degree of intracellular lipid accumulation, the expression of adipogenic genes and their corresponding protein products. This is therefore the basis of *in vitro* test method development, that, when ready, can be proposed for test guideline development, as part of the obesity endpoint aspect of an IATA for metabolic disruption.

### 1.3 Testing strategies

Currently, there are no existing test guidelines, approaches or testing strategies for metabolic disrupting chemicals, but the need for such methods has been expressed in a number of international and European reports (44–46), and the intention to develop and optimise such test methods was part of the European Commission Horizon 2020 call that the GOLIATH consortium has striven to address.

For regulatory purposes, test methods need to demonstrate that they are able to address the intended chemical applicability domain and to classify chemicals correctly (47–50). *In vitro* test method tools can provide mechanistic information to elucidate the modes of action leading to apical adverse outcomes, thereby contributing to the reduction of *in vivo* testing (51), especially when combined within IATAs (50). To facilitate the development of IATAs, to improve human health relevance and better utilise *in vivo* data, new approach methodologies (NAMs) can also provide concentration-response information. Historically, for many OECD *in vitro* Test Guidelines (TGs), the initial focus has been on (dichotomous) hazard identification particularly for classification and labelling needs, but also based upon the extent to which the results from the validation study can be reliably interpreted. However, to enable *in vitro* test methods to be used beyond prioritisation for subsequent testing in animal studies, to develop utility within IATAs, it is of value if a test method can also provide continuous (quantitative) data to inform upon the potency of a chemical (47, 52). This will enable the characterisation of the influence of physico-chemical properties on variability across the concentration response being generated (52).

Here we report on a minimum set of tentative provisional proficiency chemicals for test method development, optimisation and (pre-)validation testing for molecular and tissue-level effects with relevance towards metabolic disruption. Specifically, chemicals were selected to probe molecular transactivation of the human nuclear receptors PPARα and PPARγ and also adipogenesis (for the latter, commitment of human mesenchymal stem cells to the adipose lineage, and promotion of lipid accumulation in WAT adipocytes is key).

## 2 Methods

The selection of chemicals, suitable for the industrial chemical applicability domain and associated test method regulatory purposes was based upon the following criteria: i) structural diversity to address the physico-chemical properties applicability domain of the industrial chemical universe (e.g. biocides, pesticides, plasticizers, flame retardants, industrial coatings, pharmaceuticals etc.), for which the test method is intended to predict endpoint-specific toxicity (i.e., PPARα, PPARγ, adipogenesis), ii) receptor cross-talk in lipid homeostasis, and iii) be structurally relevant for the biological role of the endpoint, as shown for example with known natural or endogenous ligands, and in PPAR molecular modelling receptor ligand binding studies, as often conducted in drug discovery (31, 53–58).

At the outset of the project, in 2020, a targeted literature review was performed, for each endpoint, utilising expert knowledge in the fields of nuclear receptor activation, adipogenesis, nutrition and metabolic disruption, together with (guidance from) highly relevant well-documented reviews, to focus and retrieve pertinent chemical and target endpoint literature evidence. The literature search was later updated and supplemented in relation to PPAR and adipogenesis obesity key events in late 2023, to include relevant recently published articles for each chemical and test method. Details regarding the literature search are provided in **Supplementary Material 1**: Literature search.

The data were primarily prioritised and evaluated according to greatest human relevance and data reproducibility, with support from data generated within the GOLIATH project, e.g. Garoche et al. (59), for candidate proficiency chemicals. Based on this initial expert input, key candidate chemicals were identified, and the Scopus and PubMed databases were iteratively queried for additional, related chemicals with substantial literature support. Where identified, prototypical chemicals from literature review articles were included in the preliminary candidate list. Chemicals with the highest assessment with a reasonably well documented weight of supporting evidence, relevance towards metabolic disruption mechanisms, and relevance towards OECD test method development, were prioritized and are proposed as tentative chemicals for test method development, optimisation, proficiency, and (pre-)validation testing. These are summarised in **Table 1**, together with concentration information, CAS Number and 2D structures, and are itemised for PPARα, PPARγ and adipogenesis in, **Tables 2A–C** respectively, with the detailed review provided in **Supplementary Material 2**: **Table 1**. Structural diversity was assessed by structural examination in combination, with an understanding of known ligands and molecular pharmacophore and docking studies for the PPARs (31, 54–56, 58, 225).

**Table 1:** Summary chemical selection table for the pre-validation of hPPARα and hPPARγ transactivation, and hMSC adipogenesis test methods.

Chemical	Cas No. Molecular weight Log P	Structure	Use	Test methods Recommended to include in assays: ag/antag ● inactive ○			Key References (See also **Supplementary Material 2: Table 1**)	Comments/inclusion in test method chemical set?
bold font: selected chemicals				hPPARα	hPPARγ	hMSC adipogenesis (lipid accumulation)		
(aR)-4-chloro-a-[3-(trifluoromethyl) phenoxy]benzeneacetic acid, (MBX-102/JNJ39659100) Arhalofenate MBX-102	24136–23-0 415.8 g/mol na	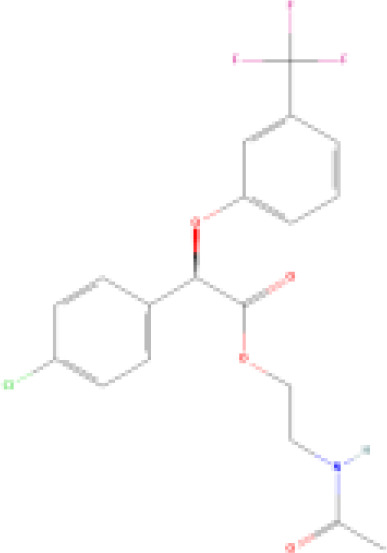	Experimental pharmaceutical	○ -	● + Weak agonist	?	(60)	hPPARγ weak positive. Mechanistically interesting, suitable back-up chemical. Triglyceride lowering effect of MBX-102 is PPARα independent. Limited amount of supporting literature retrieved, therefore lower priority for test method development.
15-Deoxy-Δ12,14-prostaglandin J2 (15d-PGJ2)	87893–55-8 316.4 g/mol 3.983	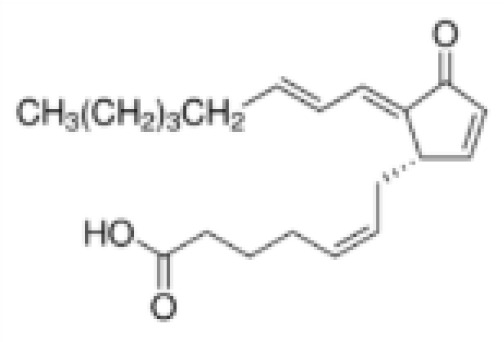	Metabolite of endogenous prostaglandin (PGJ2)	?	● + Strong agonist	?	(19, 61, 62)	hPPARγ strong positive, endogenous ligand. Difficult to work with due to (low) stability, but reliably works as an agonist in the PPARγ transactivation assay.
1alpha,25-Dihydroxyvitamin D3 (calcitriol)Active metabolite of Vitamin D3) (OHVitD3)	32222–06-3 416.6 g/mol 5	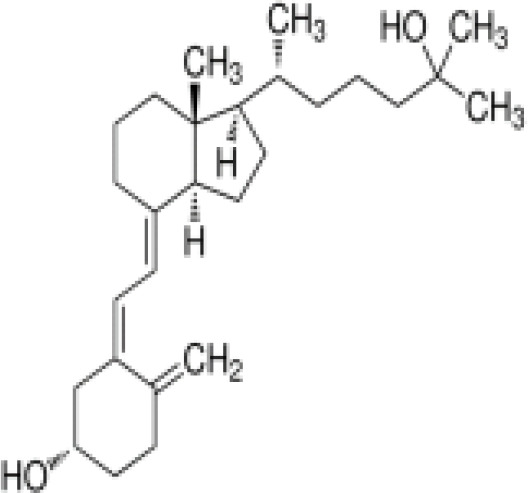	Nutrient/essential vitamin/vitamin supplement/ Hormone Vitamin D3 analog Metabolite of Vitamin D3	?	?	Unknown	(63–78)	Vitamin D active metabolite, inhibits intracellular fat accumulation. Therapeutic benefits in reducing body fat mass. Potential crosstalk with RXR. Part of signalling pathway in adipose/osteoblast development. Expensive chemical.
**3,3’,5,5’ Tetrabromobisphenol A (TBBPA)**	79–94-7 543.9 g/mol at 25°C: 6.53 (pH 3.05), 4.75 (pH 7.53), 3.00 (pH 8.12), 1.25 (pH 9.18)	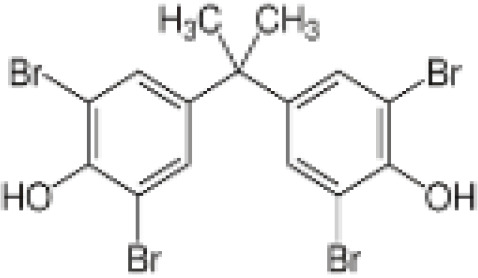	Flame retardant	○ -/?	● + Strong agonist	● +Moderate inducer 10 μM induced adipogenesis in 3T3-L1 cells	(7, 59, 62, 79–83)	hPPARγ andadipogenesis positive; hPPARα negative/non-agonist Structurally similar to BPA but different activity in PPARs. Active metabolite: sulfate (but TBBPA sulfate is not stable over time/difficult to store).
AGN194204 (IRX4204)	220619–73-8 352.5 g/mol na	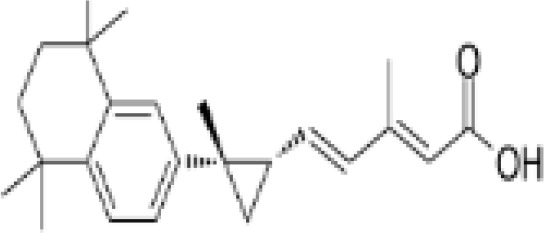	Pharmaceutical	● Weak antagonist	?	?	(84, 85)	PPARα weak antagonist. Selective RXR agonist (0.1–1 nM); slight activation of RARα/β/γ 0.1–1 µM, no activation of FXR, LXRα/β, PPARγ up to 11–10 µM. Anti-inflammatory and anticarcinogenic properties. Limited amount of supporting literature retrieved, therefore lower priority for test method development.
**Bisphenol A (BPA)**	80–05-7 228.29 g/mol 3.32	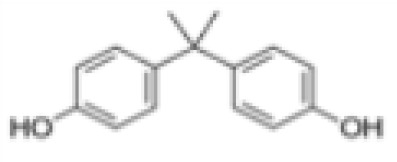	Plasticiser, industrial chemical	○ -	○ -	-/~	(7, 62, 79, 81, 83, 86–127)	hPPARα/γ negative Adipogenesis: Mixed reports. Negative in all adipogenic cell lines except one, murine 3T3-L1 cell line, likely this is because the cell line is already induced, so results for this cell line are borderline/weakly active. 3T3-L1 cell line is less human relevant than primary human cells. Sexual dimorphism: mostly female-specific adipogenesis
Chlorpyrifos (CPF)	2921–88-2 350.6 g/mol 4.96	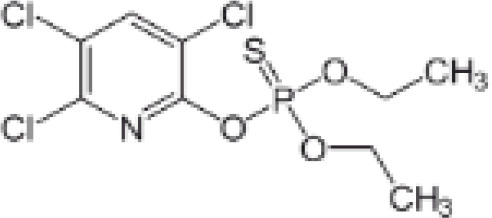	Organophosphate pesticide	○ -	● + Weak agonist	○ -	(93, 128–135)	Some animal studies showed an association between CPF and obesity as well as metabolic disruption. CPF oxon is the major *in vivo* metabolite. The *in vitro* evidence is limited. One study showed 0.1, 1, and 10 μM chlorpyrifos inhibited the osteogenic differentiation capacity of human MSCs, although the potential of MSCs to differentiate into adipocytes was not tested. Likely genotoxicity and developmental neurotoxicity, lower priority in terms of prospective use in the EU.
Clofibrate	637–07-0 242.70 g/mol 3.3	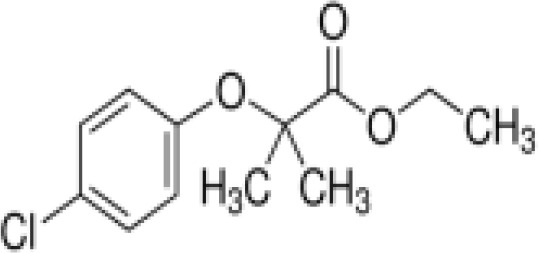	Pharmaceutical, fibrate	● + Weak-moderate agonist up to 10^–4^ µM	● + Weak-moderate agonist	?-	(136–143)	hPPARα/γ moderate positive. Widely used for lowering triglyceride levels. However, the metabolite clofibric acid is more active. 50% on PPARα (-10 µM), 40% PPARγ.
**Clofibrate metabolite: Clofibric acid**	882–09-7 214.64 g/mol na	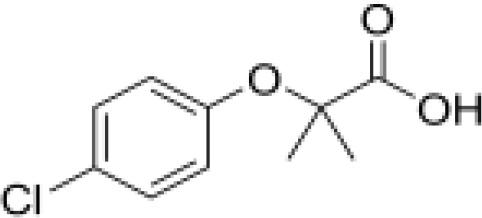	Herbicide and pharmaceutical; active metabolite of clofibrate	● + Moderate agonist (EC_50_ = 50.0 µM)	● + Weak-Moderate agonist (but weaker than PPARα)	?-	(59, 144)	hPPARα/γ moderate/weak positive. Clofibrate metabolite; more active than clofibrate, therefore preferred over the parent chemical.
**Dichlorodiphenyl-dichloroethylene** **(p,p’-DDE)**	72–55-9 318.0 g/mol 6.51	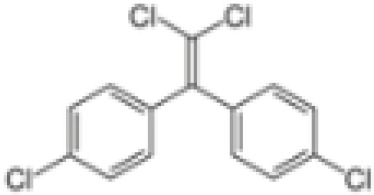	Pesticide metabolite	○ -	○ -	○ ?-	(59, 83, 93, 104, 145–154)	hPPARα/γ negative, adipogenesis negative for hMSC but pp’-DDE is adipogenic in 3T3-L1 cells (30–100 µM). The evidence is mixed from human (epidemiological) studies: no clear association with obesity. On the Stockholm POPs Convention list.
**Docosahexaenoic acid (DHA)**	6217–54-5 328.5 g/mol na	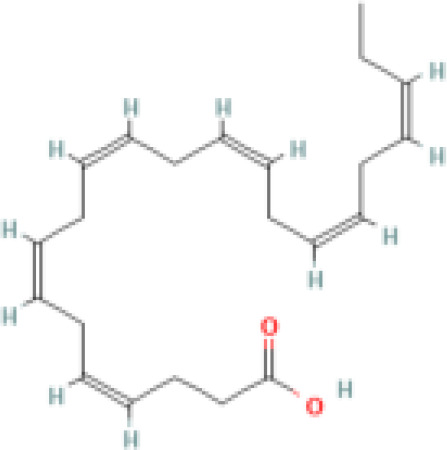	Nutrient, long chain PUFA essential fatty acid	● + Strong agonist	● + Strong agonist	?	(19, 155–158)	hPPARα/γ positive. DHA cheaper than EPA and two times more potent on PPARs
Eicosapentaenoic acid (EPA)	10417–94-4 302.5 g/mol 6.1	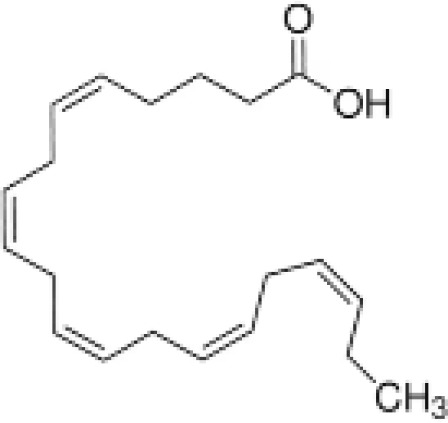	Nutrient, long chain PUFA essential fatty acid	● +Moderate agonist	● +Moderate agonist	?	(19, 31, 155, 159)	hPPARα/γ positive.
Fludioxonil	131341–86-1 248.18 g/mol 4.12 at 25°C	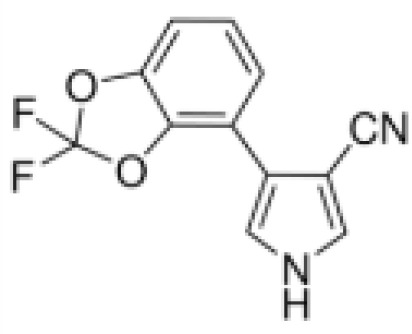	Non-systemic fungicide	?	○ -/?	● + Strong (significant at 0.2 µM)	(40, 59, 62, 160, 161)	RXRα agonist (AC50 = 14.3 µM). Adipogenic in 3T3-L1 and mBMSCs.
GW3965 hydrochloride	405911–17-3 618.5 g/mol na	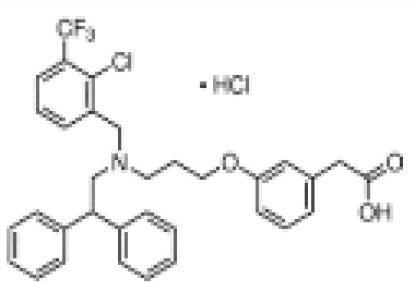	Pharmaceutical candidate	?	● + Weak agonist	● + Weak/moderate inducer	(59, 161–166)	Potent and selective liver X receptor (LXR)β agonist; commits human mesenchymal cells to adipose lineage. LXR is involved in the tissue distribution of fat (visceral vs. sub-cutaneous vs. skeletal muscle) and activation reverses cholesterol transport. LXR activation induces steatosis and may affect pancreatic beta cells. Crosstalk with PPARγ. Limited amount of supporting literature retrieved, therefore lower priority for test method development, although interesting and important mechanistically.
GW7647	265129–71-3 502.8 g/mol na	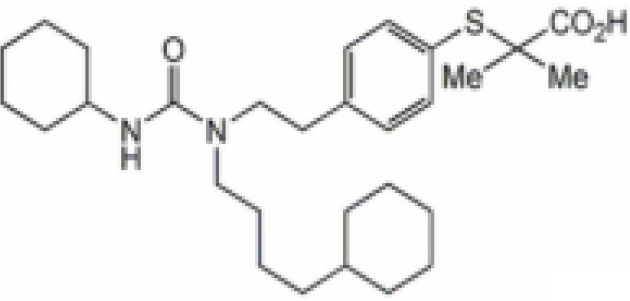	Pharmaceutical Selective	● + Selective agonist, positive control 10 nM	● + Moderate agonist	?	(32, 59, 140, 167–169)	Selective agonist for PPARα; 1000x less potent on PPARγ than on PPARα
LGD1069 (Targretin) Bexarotene	153559–49-0 348.5 g/mol 6.9	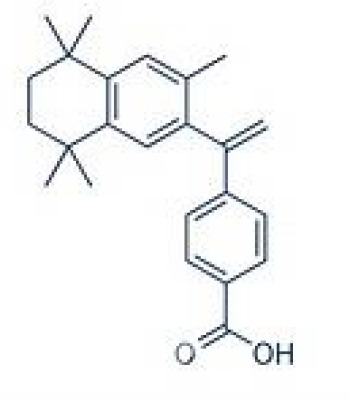	Pharmaceutical	○ -	○ -	?	(170–173)	Predicted to be positive for adipogenesis in the 3T3-L1 Filer et al., 2022 model (174). Selective RXR agonist. An RAR activator at high concentrations. 9-cis RA was excluded because it activates RAR similarly as all-trans RA (atRA) and it can be photo- or thermally isomerized to other forms (same with atRA). The best available RXR ligands are LGD100268 and AGN 194204. Limited amount of supporting literature retrieved, therefore lower priority for test method development.
**Mono-(2-Ethylhexyl) Phthalate (MEHP)** **DEHP metabolite**	4376–20-9 278.34 g/mol na	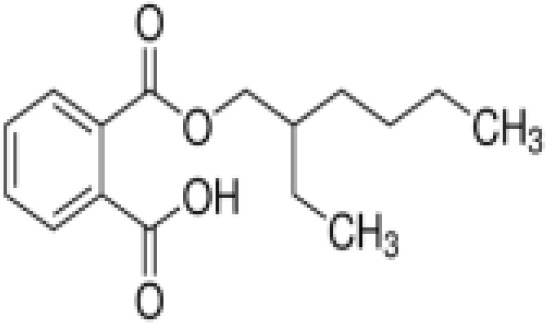	Phthalate, plasticiser	● + Moderate agonist	● + Moderate agonist	● + Moderate 10 μM and was maximal at 100 μM	(59, 81, 93, 102, 119, 175–188)	hPPARα/γ/adipogenesis positive. Active metabolite of DEHP Activity via PPARγ is mediated via the metabolite, MEHP, not the parent chemical DEHP. Cytotoxic at higher concentrations in the adipogenesis assay.
**Perfluorohexanoic acid (PFHXA)**	307–24-4 314.05 g/mol na	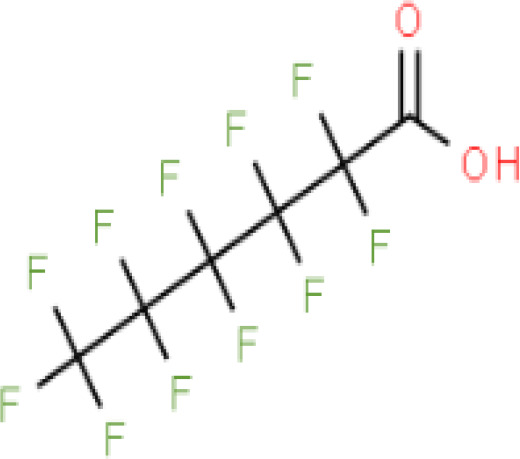	Breakdown product of PFAS. Used in stain resistance, carpets, photographic film & PFCAs substitute	○ -	○ -	?	(59, 189, 190)	hPPARα/γ negative. Tentative negative for adipogenesis, despite association with altered blood/serum lipid composition. Pronounced reproductive toxicity. Member of polyfluorinated chemicals group, for which application of international restrictions are foreseeable.
**Perfluorooctanoic acid (PFOA)**	335–67-1 414.07 g/mol 6.3	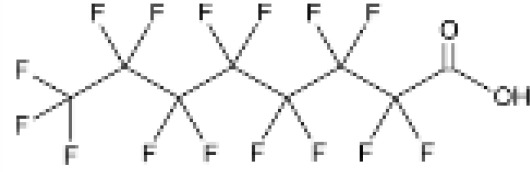	Industrial chemical, non-stick coating	● + Strong agonist	● + Strong agonist	● + Weak inducer	(59, 81, 83, 93, 100, 104, 149, 190–192)	hPPARα/γ/adipogenesis positive. PFOA-family compounds with 8–9 carbon backbone have greater activity than those with 7 and 10 carbons. PFAS: strong association of grandmaternal exposure with obesity in granddaughters. Both, negative and positive for lipid accumulation in murine 3T3-L1 pre-adipocytes and mixed evidence from human models (*in vitro* and epidemiology). Human *in vivo*: some indication for association with altered lipid profile (esp. triglycerides), but no clear correlation with obesity, fat mass, or body weight. Listed under Stockholm convention on POPs.
**Phytanic acid**	14721–66-5 312.5 g/mol na	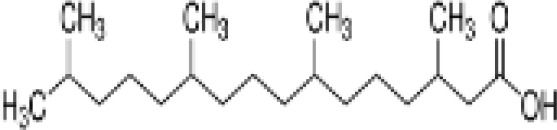	Dietary lipid	● + Very weak agonist 10^–4^ µM	● + Weak-moderate agonist	?	(31, 188, 193–198)	hPPARα/γ weak positive. Data suggest that phytanic acid is a natural agonist for RXR at physiological concentrations, while it is more likely that it is the metabolite pristanic acid, rather than phytanic acid itself, that acts as PPARα agonist. Phytanic acid, but not pristanic acid, mediates the beneficial effects of phytol derivatives on brown adipocyte differentiation (193)
**Phytanic acid metabolite: Pristanic acid**	1189–37-3 298.5 g/mol na	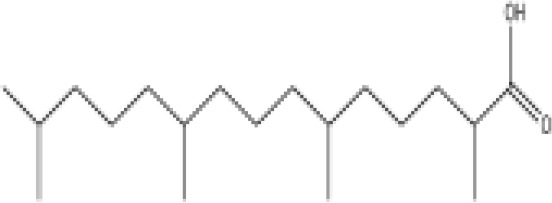	Dietary lipid. Metabolite of phytanic acid	● + Strong agonist 1 µM	● + Weak-moderate agonist 10 µM	?	(31, 188, 193, 195, 196)	hPPARα/γ positive, more potent in hPPARα. Active metabolite of phytanic acid (see notes above).
**Rosiglitazone (ROSI)**	122320-73-4 357.4 g/mol 2.4	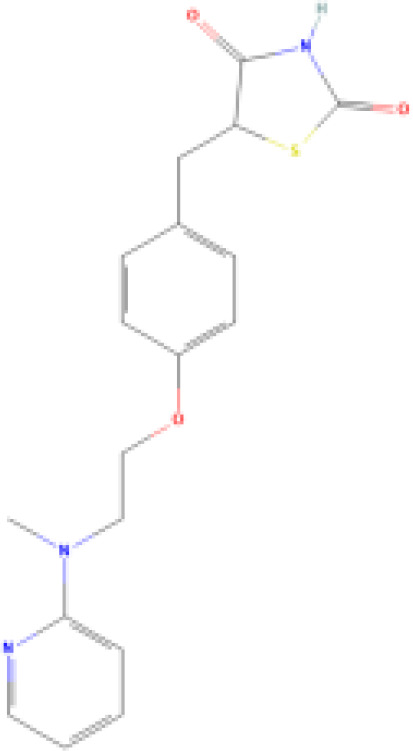	Pharmaceutical	○ -	● + Positive control	● + Positive control	(59, 81, 82, 93, 119, 191)	Standard positive control for PPARγ 1000x less potent on PPARα than on PPARγ (168), but not reproduced. Induces fluorescence at conc. of 10^–5^M max. conc. tested 10^–6^M in cell line HG5LN.
**Tesaglitazar/** **AZ 242**	251565–85-2 408.5 g/mol na	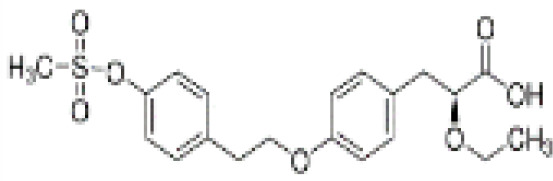	Pharmaceutical	● + Selective moderate agonist 3 µM	● + Strong agonist 40 nM	?-	(62, 199, 200)	hPPARα/γ moderate/strong positive respectively. No substantive evidence for adipogenesis induction.
**Tributyltin (TBT) chloride**	1461–22-9 325.50 g/mol 4.76	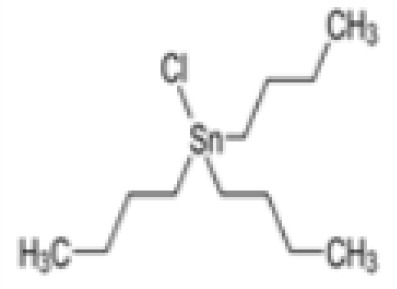	Fungicide	○ -	● + Partial agonist (weak)	● + Strong inducer adipogenic differentiation (3T3-L1)	(7, 82, 100, 102, 104, 119, 201–208)	hPPARα negative. More an effect on RXR. Most reproducible results across assays. Inhibition of luciferase in reporter gene assays, so not good to use. All Rexinoids inhibit GAL4 luciferase. Can be used in hMSC adipogenesis assay. Sexual dimorphism: male specific (209, 210)
**Triclosan (TCS)**	3380–34-5 289.5 g/mol 4.76	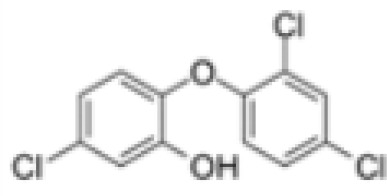	Bacteriocide	○ -	○ -	○ -	(59, 79, 104, 127, 177, 211–222)	hPPARα/γ/adipogenesis negative. Human studies: while some studies indicate adipogenesis potential, overall, the weight of evidence suggest TCS does not induce obesity/adipogenesis/lipid accumulation in adipocytes, in humans.
**Triphenyl phosphate (TPP)**	115–86-6 326.3 g/mol 4.59	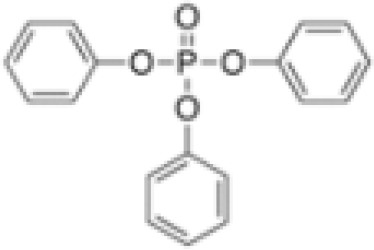	Industrial chemical: Adhesives and sealants, coating products, cosmetics and personal care products	○ -	● + Strong agonist	● + Agonist at high dose (>1µM)	(59, 62, 104, 223)	hPPARα negative, hPPARγ strong positive, with greater relative potency than PFOA.
TTNPB, 4-[(E)-2-(5,6,7,8-Tetrahydro-5,5,8,8-tetramethyl-2-naphthalenyl)-1-propenyl] benzoic acid, Arotinoid acid	71441–28-6 348.5 g/mol na	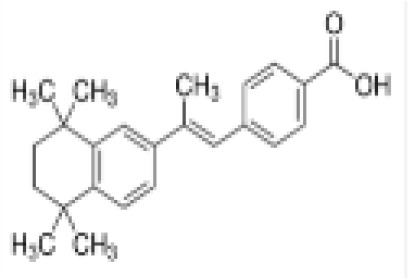	Pharmaceutical	?	?	○ - Strong inhibitor of adipogenesis; unlike retinoic acids (9cRA) that promotes adipogenesis	(224)	RAR activator (all 3 RARs). Limited amount of supporting literature retrieved, therefore lower priority for test method development.

Natural/endogenous ligands for LXR, oxysterols, and positive control for GR, dexamethasone, are lower priority chemical considerations at this stage. It is established that there is no GR crosstalk for the PPAR transactivation candidate test methods, and the intended minimum number of chemicals to be proposed.

Chemicals listed in bold are recommended as higher-priority as they have more robust literature support for test method development. Unless stated differently, activity information refers to agonism and statistical significance is p ≤ 0.05.

+, active; -, inactive; ~, equivocal; ?, uncertain/unknown; na, not available.

Source of Log P data: pubchem accessed date 3 November 2023. Chemicals in alphabetical order.

Table 2ASelected chemicals and activity bands for the PPARα assay.

**Table:** 

Chemical	Cas No.	Structure	Use	hPPARα ag/antag ⚫ inactive ⚪
Negative				
**Bisphenol A (BPA)**	80-05-7	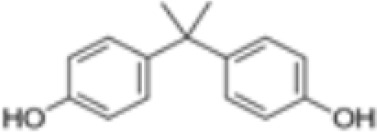	Plasticiser, industrial chemical	⚪ -
**Triphenylphosphate (TPP)**	115-86-6	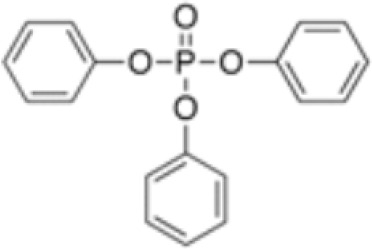	Industrial chemical	⚪ -
**Dichlorodiphenyldichloroethylene** **(pp’-DDE)**	72-55-9	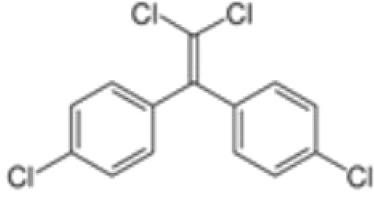	Pesticide metabolite (Stockholm POPs list)	⚪ -
**Triclosan (TCS)**	3380-34-5	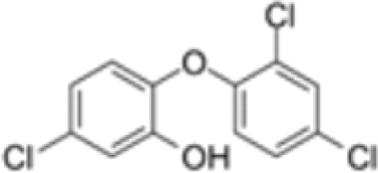	Bacteriocide	⚪ -
**Rosiglitazone (ROSI)**	122320-73-4	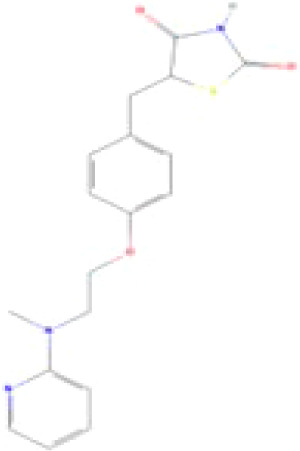	Pharmaceutical	⚪ -
**Chlorpyrifos (CPF)**	2921-88-2	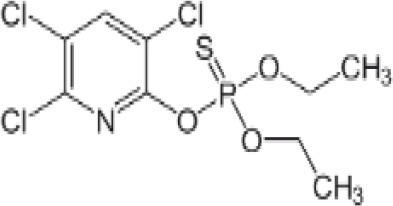	Organophosphate pesticide	⚪ -
**Perfluorohexanoic acid (PFHXA)**	307-24-4	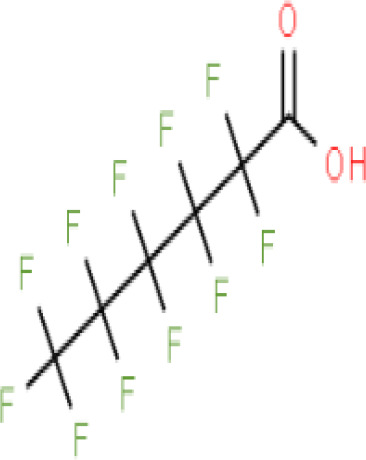	Breakdown product of PFAS	⚪ -
**(aR)-4-chloro-a-[3-(trifluoromethyl)phenoxy]benzeneacetic acid, (MBX-102/JNJ39659100)** **Arhalofenate** **MBX-102**	24136-23-0	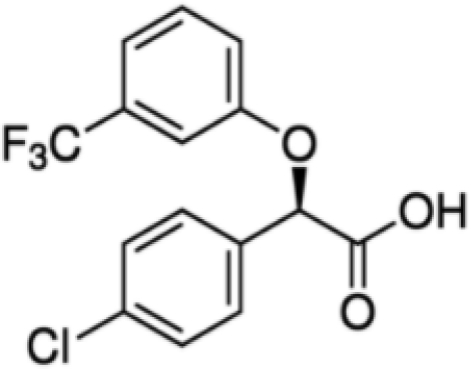	Experimental pharmaceutical	⚪ -
**Tetrabrominated BPA (TBBPA)**	79-94-7	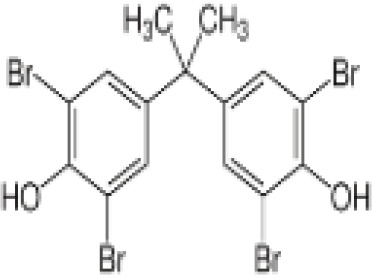	Flame retardant	⚪ -/?
**LGD1069 (Targretin) Bexarotene**	153559-49-0	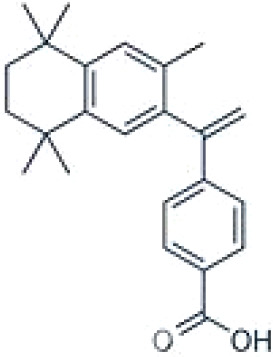	Pharmaceutical	⚪ -
Weak activity				
**Phytanic acid**	14721-66-5	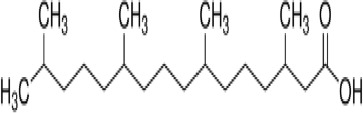	Dietary lipid	⚫ + Very weak agonist 10^-4^ µM
**Clofibrate**	637-07-0	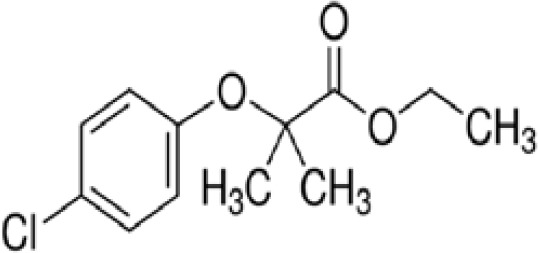	Pharmaceutical, fibrate	⚫ + Weak-moderate agonist up to 10^-4^ µM
**AGN194204 (IRX4204)**	220619-73-8	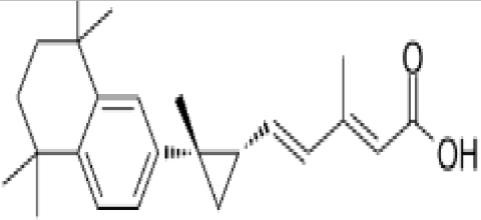	Pharmaceutical	⚫ Weak antagonist
Weak to moderate activity				
**Mono-(2-Ethylhexyl) Phthalate (MEHP)**	4376-20-9	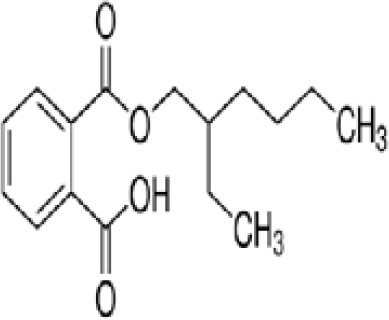	Phthalate, plasticiser	⚫ + Moderate agonist
**Eicosapentaenoic acid (EPA)**	10417-94-4	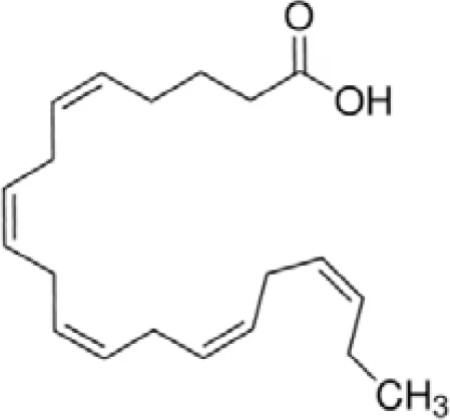	Nutrient, long chain PUFA essential fatty acid	⚫ + Moderate agonist
**Tesaglitazar/AZ242**	251565-85-2	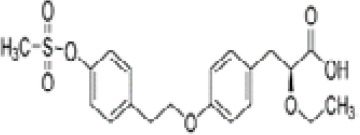	Pharmaceutical	⚫ + Selective moderate agonist 3 µM
**Clofibric acid**	882-09-7	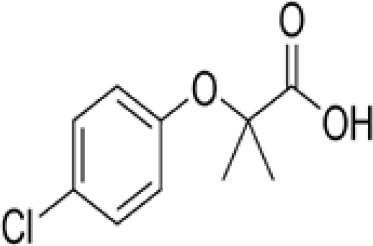	Herbicide and pharmaceutical; active metabolite of clofibrate	⚫ + Moderate agonist (EC_50_ = 50.0 µM)
Strong activity				
**Perfluorooctanoic acid (PFOA)**	335-67-1	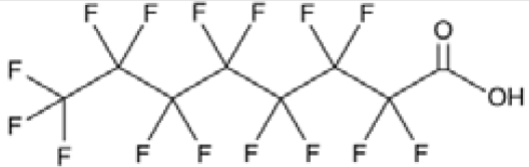	Industrial chemical, non-stick coating	⚫ + Strong agonist
**Pristanic acid**	1189-37-3	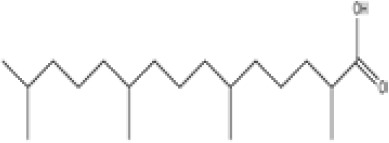	Dietary lipid	⚫ + Strong agonist 1 µM
**Docosahexaenoic acid (DHA)**	6217-54-5	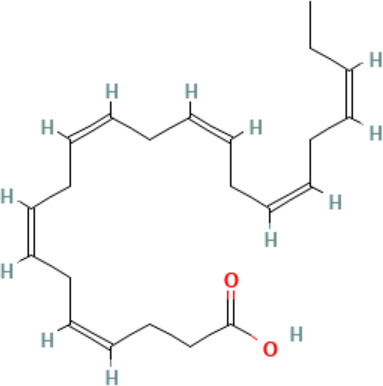	Nutrient, long chain PUFA essential fatty acid	⚫ + Strong agonist
Positive control				
**GW7647**	265129-71-3	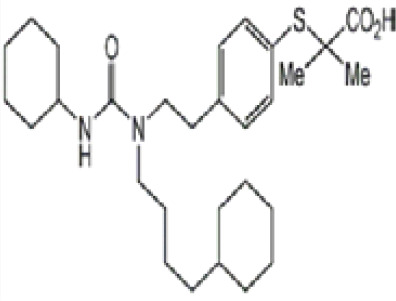	Pharmaceutical candidate	⚫ + Selective agonist, positive control10 nM

**Table 2B:** Selected chemicals and activity bands for the PPARγ assay.

Chemical	Cas No.	Structure	Use	hPPARγ ag/antag ⚫ inactive ⚪
Negative				
**Bisphenol A (BPA)**	80-05-7	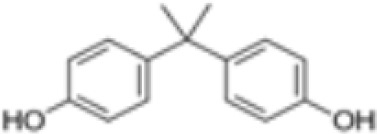	Plasticiser, industrial chemical	⚪ -
**Dichlorodiphenyldichloroethylene** **(pp’-DDE)**	72-55-9	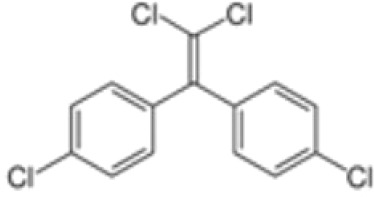	Pesticide metabolite (Stockholm POPs list)	⚪ -
**Triclosan (TCS)**	3380-34-5	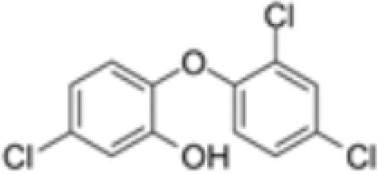	Bacteriocide	⚪ -
**Perfluorohexanoic acid (PFHXA)**	307-24-4	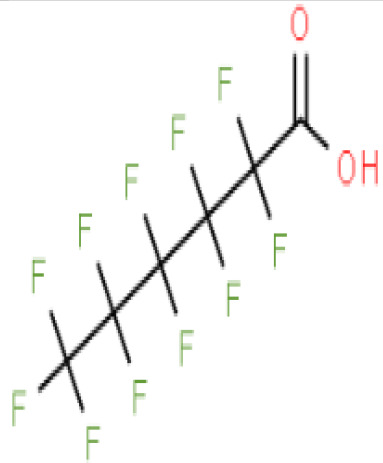	Breakdown product of PFAS	⚪ -
**LGD1069 (Targretin) Bexarotene**	153559-49-0	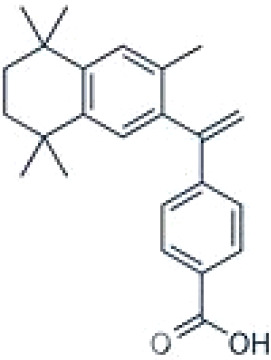	Pharmaceutical	⚪ -
Weak activity				
**Chlorpyrifos (CPF)**	2921-88-2	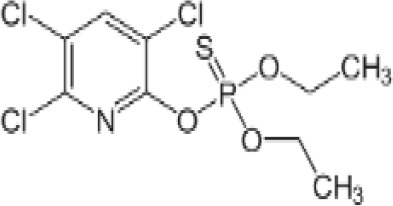	Organophosphate pesticide	⚫ + Weak agonist
**Clofibrate**	637-07-0	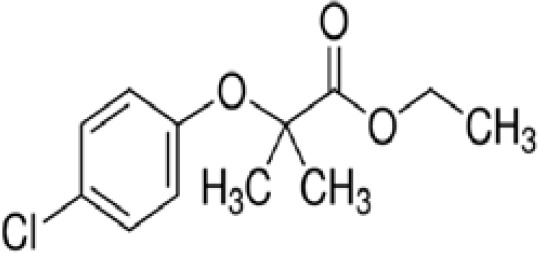	Pharmaceutical, fibrate	⚫ + Weak-moderate agonist
**Phytanic acid**	14721-66-5	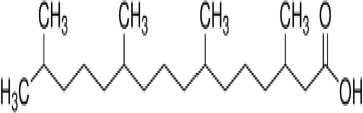	Dietary lipid	⚫ + Weak-moderate agonist
**(aR)-4-chloro-a-[3-(trifluoromethyl)phenoxy]benzeneacetic acid, (MBX-102/JNJ39659100)** **Arhalofenate** **MBX-102**	24136-23-0	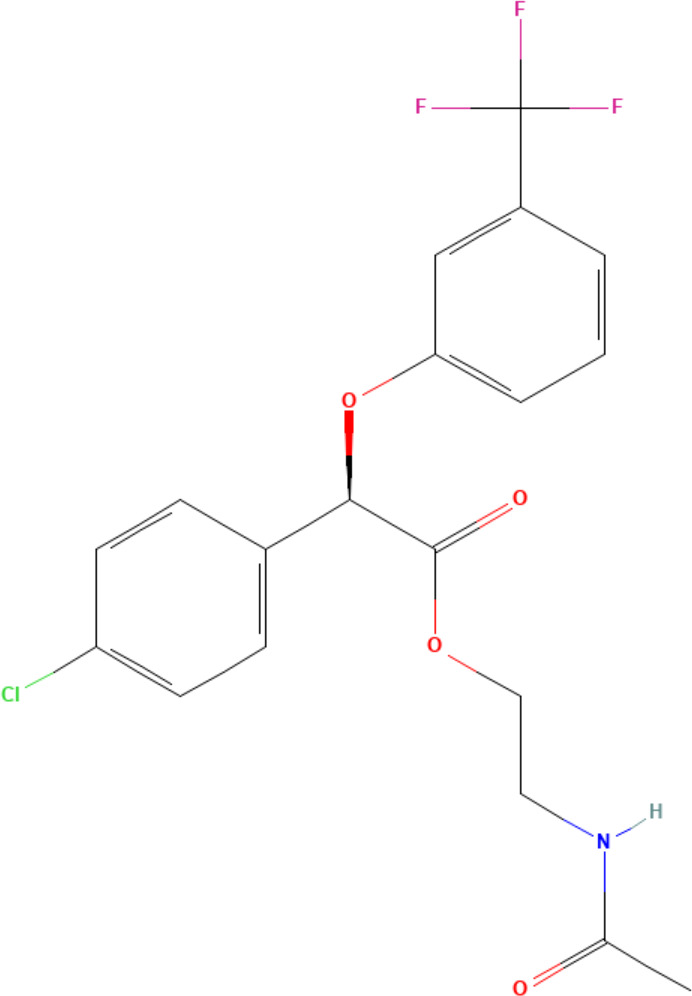	Experimental pharmaceutical	⚫ + Weak agonist
**GW3965 hydrochloride**	405911-17-3	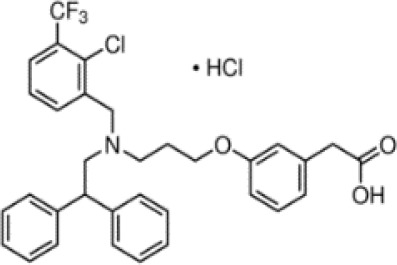	Pharmaceutical candidate	⚫ + Weak agonist
Weak to moderate activity				
**Mono-(2-Ethylhexyl) Phthalate (MEHP)**	4376-20-9	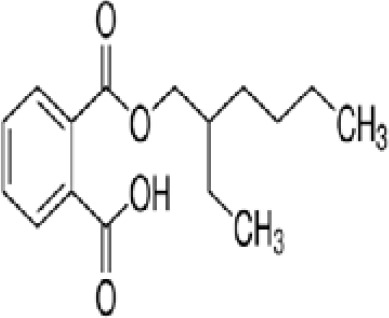	Phthalate, plasticiser	⚫ + Moderate agonist
**GW7647**	265129-71-3	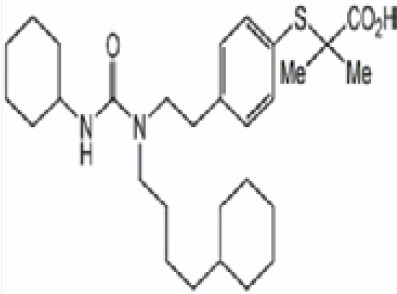	Pharmaceutical candidate	⚫ + Moderate agonist
**Eicosapentaenoic acid (EPA)**	10417-94-4	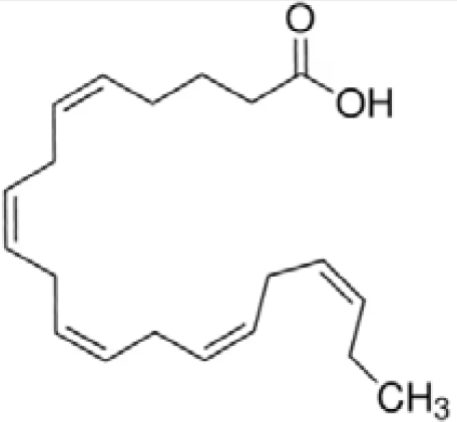	Nutrient, long chain PUFA essential fatty acid	⚫ + Moderate agonist
**Clofibric acid**	882-09-7	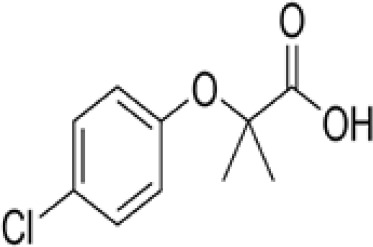	Herbicide and pharmaceutical; active metabolite of clofibrate	⚫ + Weak-Moderate agonist (but weaker than PPARα)
**Pristanic acid**	1189-37-3	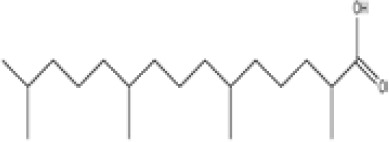	Dietary lipid	⚫ + Weak-moderate agonist 10 µM
Strong activity				
**Triphenyl phosphate (TPP)**	115-86-6	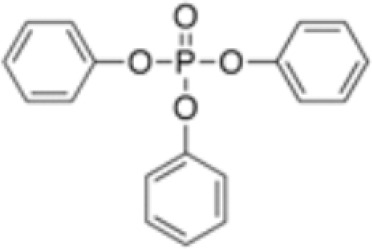	Industrial chemical: Adhesives and sealants, coating products, cosmetics and personal care products	⚫ + Strong agonist
**Docosahexaenoic acid (DHA)**	6217-54-5	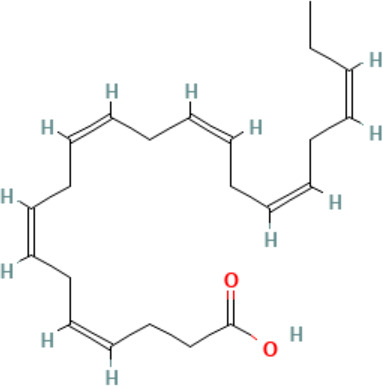	Nutrient, long chain PUFA essential fatty acid	⚫ + Strong agonist
**Tetrabrominated BPA (TBBPA)**	79-94-7	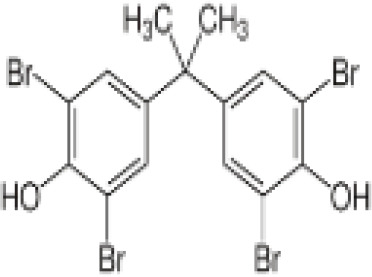	Flame retardant	⚫ + Strong agonist
**Perfluorooctanoic acid (PFOA)**	335-67-1	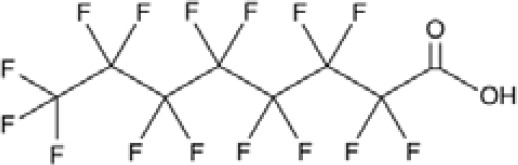	Industrial chemical, non-stick coating	⚫ + Strong agonist
**15-Deoxy-Δ12,14-prostaglandin J2 (15d-PGJ2)**	87893-55-8	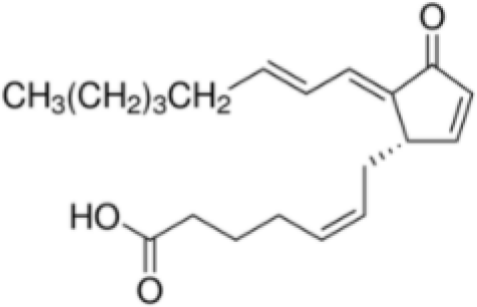	Metabolite of endogenous prostaglandin (PGJ2)	⚫ + Strong agonist
**Tesaglitazar/** **AZ 242**	251565-85-2	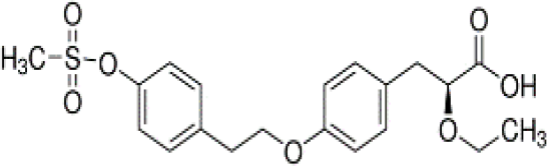	Pharmaceutical	⚫ + Strong agonist 40 nM
Positive control				
**Rosiglitazone (ROSI)**	122320-73-4	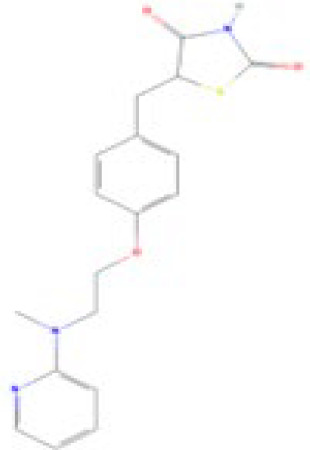	Pharmaceutical	⚫ + Positive control

**Table 2C:** Selected chemicals and activity bands for the hMSC adipogenesis assay.

Chemical	Cas No.	Structure	Use	hMSC adipogenesis (lipid accumulation) ag/antag ⚫ inactive ⚪
Negative				
**Triclosan (TCS)**	3380-34-5	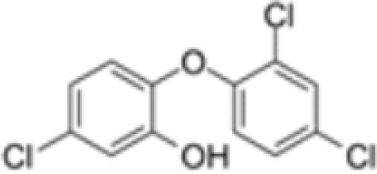	Bacteriocide	⚪ -
**TTNPB, 4-[(E)-2-(5,6,7,8-Tetrahydro-5,5,8,8-tetramethyl-2-naphthalenyl)-1-propenyl] benzoic acid, Arotinoid acid**	71441-28-6	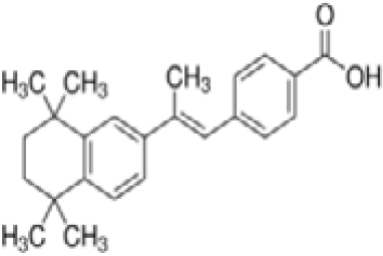	Pharmaceutical	⚪ - Strong inhibitor of adipogenesis; unlike retinoic acids (9cRA) that promotes adipogenesis
**Dichlorodiphenyldichloroethylene** **(pp’-DDE)**	72-55-9	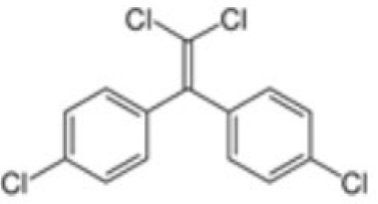	Pesticide metabolite (Stockholm POPs list)	⚪ ?-
**Chlorpyrifos (CPF)**	2921-88-2	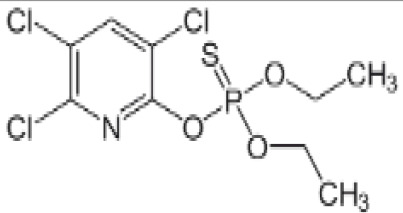	Organophosphate pesticide	⚪ -
Weak activity				
**Perfluorooctanoic acid (PFOA)**	335-67-1	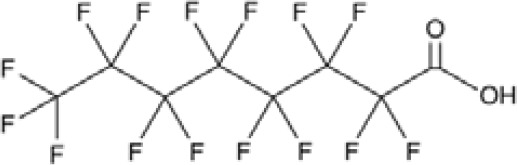	Industrial chemical, non-stick coating	⚫ + Weak inducer
Weak to moderate activity				
**GW3965 hydrochloride**	405911-17-3	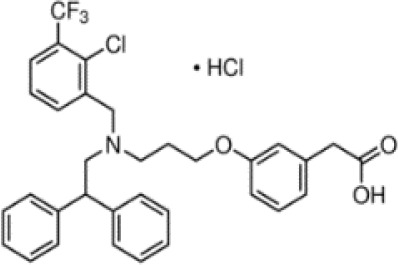	Pharmaceutical candidate	⚫ + Weak/moderate inducer
**Tetrabrominated BPA (TBBPA)**	79-94-7	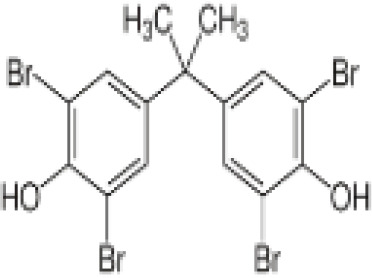	Flame retardant	⚫ +Moderate inducer 10 μM induced adipogenesis in 3T3-L1 cells
**Mono-(2-Ethylhexyl) Phthalate (MEHP)**	4376-20-9	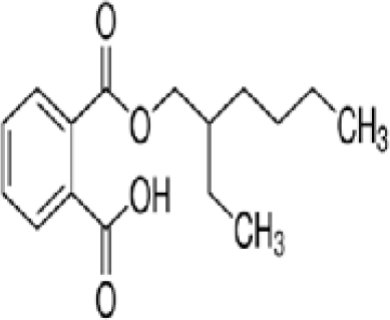	Phthalate, plasticiser	⚫ + Moderate 10 μM and was maximal at 100 μM
**Triphenyl phosphate (TPP)**	115-86-6	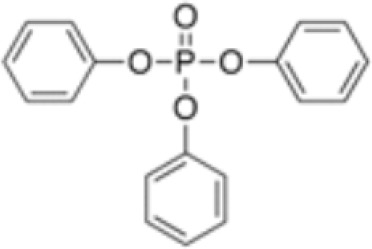	Industrial chemical: Adhesives and sealants, coating products, cosmetics and personal care products	⚫ + Agonist at high dose (>1µM)
Strong activity				
**Fludioxonil**	131341-86-1	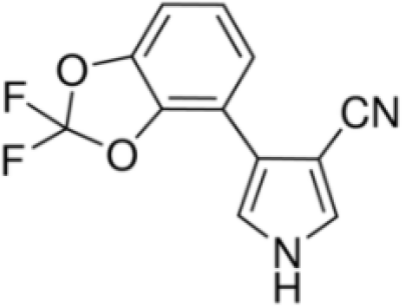	Non-systemic fungicide	⚫ + Strong (significant at 0.2 µM)
**Tributyltin (TBT) chloride**	1461-22-9	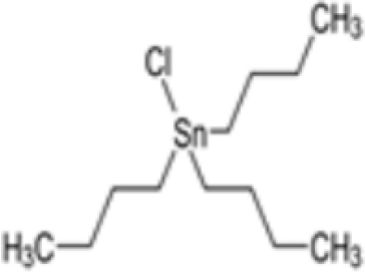	Fungicide	⚫ + Strong inducer adipogenic differentiation (3T3-L1)
Positive control				
**Rosiglitazone (ROSI)**	122320-73-4	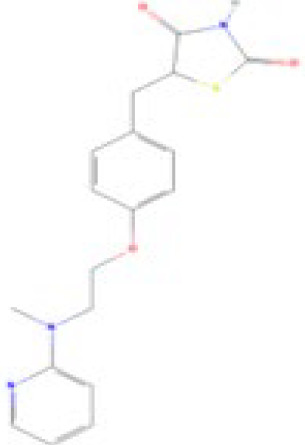	Pharmaceutical	⚫ + Positive control

As documented in **Supplementary Material 2: Table 1**: The Weight of Evidence (WoE) approach undertaken to evaluate the literature obtained first examined the scope of each paper, describing the study or review undertaken, the range of concentrations/doses tested where given, and collated the uncertainties and limitations of each paper reviewed, to arrive at a summary of the literature for each chemical, in relation to the specific endpoints under scrutiny.

Chemical selection coverage considered specific reference chemicals for the hPPARα and hPPARγ transactivation test methods and the adipogenesis test method individually. It also included chemicals that are in common with PPARα and PPARγ, PPARα but not PPARγ, and then PPARγ and not PPARα. And then also in combination with the (hMSC) adipogenesis test method evaluates the capacity of chemicals to stimulate the formation of (*in vitro*) human adipocytes. Additionally, the PPAR heterodimerisation partner RXR was also considered, as studies have shown that activation of RXRα without direct binding of PPARγ can lead to increased lipid accumulation in hMSCs. This has been shown for example for 2,4-di-tert-butylphenol (2,4-DTBP) (226) and for tributyltin (TBT) (227) and this mechanism is considered perturb the development of WAT.

The aim was also to have as broad a coverage as realistically possible within a minimum number of chemicals for which there is robust evidence, to address and probe the chemical applicability domain that the metabolic disruption IATA for obesity needs to address. As indicated for the development of testing strategies, the chemical applicability domain needs to reflect the chemical sector universe for which the test method will be utilised, and the chemicals selected and subsequently tested can start to gauge the relative points of departure for the mechanism and/or endpoints under investigation.

In common with, and expanding upon the primary considerations for the selection of chemicals (48) and as indicated in previous publications (12, 13), the following aspects also guided the selection and prioritization of candidate chemicals: Availability and international considerations; cost; human mechanistic relevance; reproducibility; range of activity; inclusion of metabolites and avoidance of undefined chemical mixtures.

### 2.1 Availability and international limitations in transport and use of certain chemicals

The chemicals were selected to accommodate national and international limitations on use, as the pre-validation reference chemicals will ultimately be intended for use in OECD TGs, with global distribution and use. Consequently, chemicals listed under the Stockholm Convention on Persistent Organic Pollutants (228) or being proposed to be added, were generally avoided, with respect to validation and subsequent TG development.

However, some chemical classes are of high current interest, and a relatively rich amount of information was available for these, particularly the perfluorinated chemicals (189–191, 229). Whilst it is very useful to learn more about the toxicity of these chemicals and confirm test method performance in the development/optimisation phase, for (pre-)validation purposes, it is preferred to keep the reference and proficiency chemicals that also are on the POPs list, to an absolute minimum, as, in addition to the disposal issues, this may lead to practical difficulties in running the tests and acceptance for some OECD member countries.

Additional chemicals to be excluded are those that have (variable) global restrictions with respect to substance abuse, such as anabolic steroids, and (illegal) drugs such as cannabinoids. Thus, while there are interesting data available for anabolic steroids and cannabinoid chemical classes, with potency for the endpoints of interest (230–233), these were not suitable for test method validation purposes.

### 2.2 Cost

Chemicals that are rare, difficult to obtain and costly are generally not be prioritised unless there are no alternatives. Ultimately the components of a validated test method that is intended for OECD (TG) adoption needs to be globally and financially accessible for all OECD stakeholders. There also needs to be consideration of longer-term production and availability of the proficiency chemicals as far as reasonable. Specific issues in this regard for these chemicals were checked and concerns were not identified.

### 2.3 Species relevance

Chemicals were selected based on high relevance to humans, wherever possible. This is because the test methods are being developed to address human relevant mechanisms/endpoints. Species specific differences are well known for PPAR transactivation and metabolism studies (57, 59), although for both human and mouse mesenchymal stem cells, cannabidiol for example has been shown to activate mouse and human PPARγ, but not its heterodimeric partner RXR. As a consequence of PPARγ activation, increased lipid accumulation and the expression of adipogenic genes was shown for both species *in vitro* (230).

Human MSC data are considered to be more human relevant than murine 3T3-L1 data, as discussed further in the following section. Whilst the 3T3-L1 mouse pre-adipocyte cell line is extensively utilised for identifying adipogenic chemicals, and it is easier to use a cell line, rather than primary cells, the species relevance with respect to induction of human adipogenesis is weaker. This cell line is already induced/committed to the (pre-)adipocyte lineage, so data generated are indicating the potential for promotion but not initiation of adipogenesis. For this particular cell line, data often differ for certain chemicals, as compared to a more human relevant model using primary hMSCs (41) and pre-validation (Hoffmann et al., manuscript in preparation) and lineage derived cells (201), and thus greater weight in the literature review was given to data generated using *in vitro* test systems that better address initiation of adipogenesis.

### 2.4 Reproducibility and approaches needed where data is not well reproduced

With test method validation in mind, chemicals need to be selected on the basis of reproducible data, not a single literature report. Where data have been reported to not be reproducible, as for example with the PPAR activity data from Tox21 (40, 160), these data were not used. Janesick et al. (160) report that in trying to reproduce the ToxCast data only 5/21 of the top scoring ToxCast™ PPARγ activators were activators, three were PPARγ antagonists, the remainder were inactive. The authentic PPARγ activators identified induced adipogenesis in 3T3-L1 cells and murine MSCs. Only 7 of the 17 chemicals predicted to be active by the ToxCast™ ToxPi promoted adipogenesis, one inhibited adipogenesis, and two of the 7 predicted negatives were also adipogenic. Of these 9 adipogenic chemicals, three activated PPARγ, and one activated RXRα. It can therefore be concluded that ToxCast™ PPARγ and RXRα assays and some of the computational tool predictions available from the US EPA Chemical Dashboard, are suitable for high throughput screening (HTS) in the identification of potential hazards and prioritisation of chemicals from large chemical libraries that could not otherwise be screened in lower-throughput model systems. However, for the test method applications intended here, the lack of correlation with laboratory measurements of PPARγ and RXR meant that the data were not sufficiently robust to support the chemical selection. Therefore, taken in isolation, this set of 3T3-L1 data was considered to be unreliable for identifying new PPAR and adipogenesis reference chemicals, but was useful in contributing to the WoE. Notably, the 3T3-L1 cell line is derived from mouse embryonic fibroblasts, and while it shares some similarities with human adipocytes, there are significant species-specific differences in gene expression, metabolism, and response to stimuli. As with many and indeed most cell lines that haven’t been validated, there can be variability in the differentiation efficiency of 3T3-L1 cells between different laboratories and experimental conditions, as well as strain drift. Factors such as passage number, cell confluency, and the composition of differentiation media can influence the extent and kinetics of adipocyte differentiation in the cell line, leading to inconsistencies in experimental results. Due to the limitations and variability associated with 3T3-L1 cell-based assays, hazard assessment for regulatory purposes needs to be cautious if relying solely on data generated from these models in regulatory decisions. However, such data does have utility as supporting information, with value for future applications, as data robustness and reliability is improved.

Independently, Filer et al. (174) assessed the discrepancies and uncertainty of ToxPi predictions using the different ToxCast datasets, focusing on the accuracy to predict activity of chemicals inducing adipogenesis and metabolic disruption in murine 3T3-L1 preadipocytes (174). Poor predictive performance was considered to be a consequence of a number of factors such as reliance on a single model, the development phase/stage of ToxCast, the cytotoxicity scoring and correction system, the weighting approaches and chemical selection utilised (174). While an adjusted prediction model yielded a balanced accuracy of 0.97 in predicting adipogenesis of a ToxCast chemical subset, the authors recommend that an extended verification of the predictive capacity with more chemicals (38 in training set, 30 in test set) is needed to better cover the diverse chemical space. Examining the models developed by Filer et al., for the purposes of this chemical selection, it was noted that the focus was more on semi-volatile chemicals and use of the 3T3-L1 cell line. Use of semi-volatile chemicals, without further technical adjustments to prevent chemical cross-contamination (“chemical creep”), such as using adhesive seals on the testing plates is a confounding hazard that requires strict control, volatile chemicals generally need to be avoided in validation studies. Chemical data from Filer et al. (174) were therefore included where relevant, to support the chemicals selected (and this is noted in **Supplementary Material 2: Table 1**). Further, it has also been recognised that expression levels in cell sources of 3T3-L1 cells can vary and this can influence the classification of (adipogenic) chemicals. Gene expression assays have shown that PPARγ expression is higher in OP9 cells compared to ATCC 3T3-L1 cells. This could partially account for the lower relative fold induction observed in these cells. However, Zenbio 3T3-L1 cells exhibited the highest relative fold inductions, showing no difference with those from ATCC 3T3-L1 (86). Furthermore reported reproducibility issues and variation in 3T3-L1 preadipocytes laboratory strains (86) and different protocol induction/exposure periods and plasticware also substantially affected the results generated, as also noted above. For example, in some protocols, there was a distinction between the induction period (with “induction cocktail” or similar, often containing e.g., IBMX, insulin, dexamethasone) and exposure to test chemicals (in medium with/without induction chemicals).

Finally, while PPARγ activation is considered necessary and sufficient for adipogenesis in humans and cell culture systems, including mouse models and cell lines such as 3T3-L1, the fundamental mechanisms of adipogenesis are highly conserved across species, including humans. Activation of the signalling pathway may occur through mechanisms other than or in addition to receptor activation (25, 234).

Pharmaceutical chemicals with a defined mode of action are included, so that there is sufficient coverage of mechanistically well understood weak-moderate actives, such that target specificity is addressed as well as reasonably possible. Principally, GW3965 is included as a weak PPARγ agonist and weak adipogenesis inducer, and tesaglitazar as a weak PPARα agonist.

### 2.5 Range of activity

Chemicals selected should preferably have a range of activity that spans negative, low/weak, moderate, and high/strong activity in the test method, as compared to the positive control, but this may not always be feasible, - depending upon the quality and weight of evidence of the supporting literature. To verify the selected potency bands (negative, weak, moderate and strong) and to ensure their validity and applicability towards the target *in vitro* test method, subject matter experts reviewed the literature for information relating to concentration and dose -response, consistency and biological relevance and (potential) uncertainties. From this review (**Supplementary Material 2**: **Table 1**) putative bands of activity ranging from negative, weak to moderate and strong were determined and are provided in **Tables 1** and **2A–C**. These potency bands will be confirmed following (pre-)validation of the respective test methods.

At least 25% of the chemicals in the full reference set should be negatives, ideally more, up to 50%. While a lower share of negatives can be acceptable during test method development, optimisation and early proficiency testing, recommendations should be improved as confidence in the test method is gained aiming at ultimately achieving 25–50% negative proficiency chemicals for (pre-)validation studies. The use of tools to identify and exclude chemicals with potentially promiscuous activity was an addition to the chemical selection process, and it will be important to continue to look for clusters of *in vitro* assays that may indicate non-specific activity through a common molecular target. Therefore, all chemicals included here were also assessed with respect to their potential to deliver false-positive results in screening assays due to non-specific reactive chemistry interference, using the “pan assay interference compounds (PAINS) remover” tool (version Demo 0.99) (235). None of the structures were identified as active/positive for non-specific reactive chemistry interference using this filtering tool.

**Tables 1**, **2A–C** and the **Supplementary Material 2: Table 1.** provide summary and full review details respectively.

### 2.6 Inclusion of active human metabolite(s) of parent chemicals

Some chemical metabolites may have increased or reduced toxicity. OECD expert group recommendations are to include some active metabolites where possible (159, 236, 237). If a respective model has limited metabolic capacity, the inclusion of both the parent chemical and the active metabolite(s) is relevant.

### 2.7 Avoidance of undefined chemical mixtures

To ensure clear attribution of effects observed with the endpoint in question, when characterising both a chemical and a candidate test method, single and pure chemicals need to be used. Purity needs to be documented. Undefined isomeric and racemic mixtures of a specific chemical should be avoided. Isomeric mixtures of the same chemical are variable and consequently can result in highly variable results. This has been observed for nonylphenol in some early optimisation and validation efforts for ER binding, for example (Jacobs personal communication), and so is best avoided, unless the isomer distribution can be clearly and consistently quantified, isomers can be tested individually, and there is additional justification for using such a chemical.

### 2.8 IATA development considerations with respect to chemical selection across the GOLIATH metabolic disruption test methods

As indicated in the methods section 2, this chemical selection was developed to support the design and development of a conceptual IATA for metabolic disruption, with specific reference chemicals for each PPARα and PPARγ transactivation test method, and the WAT adipogenesis test method, but also to selectively probe each one of the three test methods, as well as consideration of the heterodimerisation partner RXR. Thus, the chemicals were selected to provide some overlap, and thus strengthen the mechanistic evidence linking molecular and adverse organ/tissue-level effects and support the development of both Adverse Outcome Pathways (AOPs) and IATAs for metabolic disruption. This approach to chemical selection is being recommended to enhance regulatory relevant development of IATAs and the design of validation studies for the NAMs addressing the IATA KEs (238).

## 3 Results

### 3.1 The PPARα and PPARγ transactivation test methods

As diabetes and metabolic diseases are often associated with high blood glucose and lipid levels, drugs that activate both PPARα/γ are included. In addition to the environmental contaminants and pharmaceuticals known to perturb this receptor, inclusion of natural ligands will assist in developing the predictive models for PPAR transactivation test methods.

Learnings from medicinal chemistry developments and molecular docking studies (7, 59, 239–243), contribute to the selection, especially in relation to physico-chemical properties. For example, the addition of fluorine to the headsets of experimental PPAR ligands, increase potency and persistence, and can indicate the likelihood of respective PPAR ligand binding, as seen *in vitro*, for example, with the PFAS class of chemicals (59).

Looking at the PPARs heterodimerisation partner RXR, RXR ligands such as CD3264, TBT, or 9-*cis* retinoic acid are reported to interfere in reporter cell lines such as the HG5LN GAL4-PPAR, by decreasing the signal induced by the PPAR ligands according to their Kd for RXRs (59). Therefore, the RXR ligand TBT is included in the chemical selection for an adipogenesis test method, and relevant RXR interactions reported in the literature are captured.

Whilst inclusion of more chemicals with antagonistic activity would be desirable for mechanistic understanding and complete characterisation of a chemical’s activity towards the respective receptor, such information was scarce, and support from human health-relevant studies (i.e., human *in vivo* information) was lacking. Therefore, the tentative preliminary proficiency chemicals proposed herein focus on PPAR agonist binding/activity modalities.

The hMSC-derived *in vitro* adipogenesis test method is the prototypical method that was considered as a potentially suitable candidate for human-relevant WAT adipogenesis (230). While the US EPA Tox21 screening battery utilises the murine 3T3-L1 preadipocyte cell line for identifying (environmental) obesogens with recently reported more satisfactory accuracy (174), the data did not satisfy the objectives here, with respect to greater accuracy of human relevance. The aim of this chemical selection was to identify chemicals with highest relevance towards the perturbation of initiation and promotion of human adipogenesis, and for a candidate test method to ideally capture both, the commitment of precursor cells such as MSCs, to the (WAT) adipocyte lineage, and the subsequent promotion of intracellular lipid accumulation.

Building upon the chemicals selected for PPARα and PPARγ, the chemicals to be utilised for this test method could also include modulators of the Wnt and other critical signalling pathways with a demonstrated role in adipogenesis (244–247), and also in relation to the PPAR heterodimerization partner RXR. With respect to the latter, pharmacological rexinoids such as targretin (170), and natural ligands e.g. retinoic acids (248) that have also been shown to directly interact with PPAR receptors (249, 250) were also considered. The putative potency bands derived were negatives and positives, in addition to the positive control (weak, weak to moderate, moderate and strong activity).

In summary, 20 chemicals, including back-up chemicals were identified as a source list for PPARα preliminary proficiency testing. Of these, 10 are negatives: BPA, TPP, pp’-DDE, TCS, ROSI, CPF, PFHXA, MBX-102, TBBPA and targretin; and 10 are positives: phytanic acid, clofibrate and AGN194204 (3 weak agonists); MEHP, EPA, tesaglitazar, and clofibric acid (4 weak-moderate agonists); PFOA, pristanic acid, DHA (3 strong agonists), plus the positive control chemical: GW7647. For PPARγ proficiency testing 21 chemicals were identified. Of which 5 are negatives: BPA, pp’-DDE, TCS, PFHXA and targretin; and 16 are positives: CPF, clofibrate, phytanic acid, MBX-102 and GW3965 hydrochloride (5 weak agonists); MEHP, GW7647, EPA, clofibric acid, and pristanic acid (5 moderate agonists), TPP, DHA, TBBPA, PFOA, 15d-PGJ2 and tesaglitazar (6 strong agonists), plus the positive control chemical: rosiglitazone. Fludioxonil was categorised as unknown for PPARγ, however there are some negative results.

For the hMSC adipogenesis test method we propose 11 proficiency chemicals. The following 4 negatives were identified: TCS, TTNPB, pp’-DDE, and CPF. 7 positives were identified, PFOA (1 weak), GW3965 hydrochloride, TBBPA, MEHP, TPP (4 moderate), fludioxonil and TBT chloride (2 strong). Rosiglitazone is the positive control for candidate adipogenesis test methods.

Phytanic acid, a natural PPAR agonist, regulates glucose metabolism in rat primary hepatocytes (251). Whilst phytanic acid induces beige adipocyte differentiation, (but does not for brown fat), this differentiation has been shown to be mediated by PPARα (252). It is also a natural RXR agonist at physiological concentrations and it mediates the favourable effects of phytol derivatives on BAT adipocyte differentiation, and also induces differentiation of UCP1 in mouse under *in vivo* and *in vitro* conditions, postulated to be via RXR especially in presence of other PPARγ activators (193). 50 μM phytanic acid treatment induced differentiation in 70% of the 3T3-L1 preadipocytes assessed by lipid droplet accumulation and aP2 mRNA induction (194), and in similar experiments this was considered to probably be mediated by RXR (195). However, adipocyte differentiation in mouse embryo fibroblast (C3H10T1/2) cells was reported to be very low, as few cells were differentiated following 50 μM phytanic acid treatment (193, 251). Thus, whilst phytanic acid has been implicated in the normal biological process of adipogenesis, further research is needed to elucidate its precise mechanisms of action and its role in adipose tissue biology including whether it is able to commit MSCs to the (WAT) adipocyte lineage, or rather promotes lipid accumulation and maturation in committed (pre-)adipocytes. Therefore, phytanic acid has been categorised in category unknown, and is currently a lower priority chemical for (initial) test method characterisation and (pre-)validation.

A summary of high priority chemicals is given in **Table 1**, and extensive background information is provided in **Supplementary Material 2: Tables 1** and **2**. Other RXR relevant chemicals are BPA, TBT, phytanic acid, calcitriol, targretin, AGN194204, fludioxonil and 2,4-DTBP. Studies have indicated that they can interact with RXR and affect its activity, potentially disrupting signalling pathways mediated by RXR-containing heterodimers (84, 87, 88, 160, 170, 194, 195, 202, 226, 253, 254).

**Figure 2:**
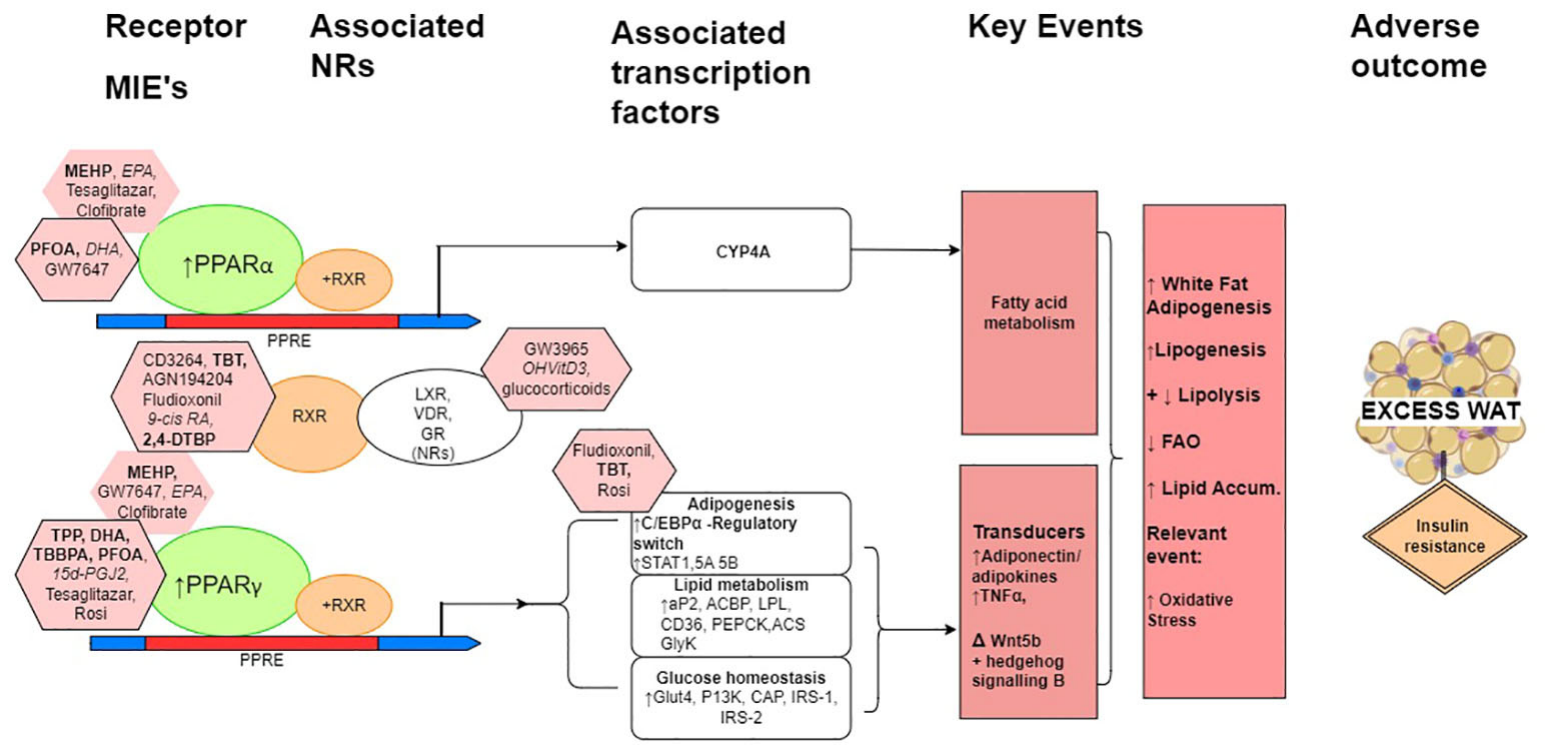
A schematic simplified overview model of the natural history leading to excess white adipose tissue with associated prototypical positive ligands indicated principally for the mechanisms mediated by the PPARs, RXR and adipogenesis. When heterodimerised with RXR, PPARα and PPARγ are the molecular initiating event (MIEs) gene regulators of PPAR response elements (PPREs), which maintain metabolic homeostasis, and when activated have multiple roles in adiposity and can lead to reduced insulin sensitivity and increased insulin resistance. (The heterodimerisation partner of the PPARs is also the heterodimerisation partner for a number of other nuclear receptors (NRs), of which LXR, VDR and GR are particularly relevant here.) In relation to adiposity, these include the induction of CYP4A gene transcription factors for lipid metabolism for PPARα to adipocyte differentiation, glucose homeostasis, and the expression and transcription of adipose tissue-secreted factors for PPARγ. C/EBPα, CCAAT/enhancer-binding protein α; STAT1, STAT5A and STAT5B, signal transducer and activator of transcription 1, 5A and 5B, respectively; aP2, fatty acid binding protein 2; ACBP, acyl-CoA–binding protein; LPL, lipoprotein lipase; CD36, cluster of differentiation 36; PEPCK, phosphoenolpyruvate carboxykinase; ACS, acyl-CoA synthetase; GyK, glycerol kinase; Glut4, glucose transporter 4; PI3K, phosphoinositide 3 kinase; IRS-1 and IRS-2, insulin receptor substrate 1 and 2, respectively. Pink octagons indicate prototypical ligands for the respective receptors that they are attached to, more potent ligands are indicated with the black border. MDCs are in bold, endogenous and natural ligands in italics, and pharmaceuticals in normal font. Negatives and weak chemicals from Tables 1 and 2 are not included in this figure.

Consideration of mechanistic and mode of action interactions of some prototypical reference/proficiency chemicals, within a putative natural history mechanistic model for overall increased adipogenesis are shown schematically in **Figure 2**. Perturbed PPAR activation is a primary MIE, that is an initial and early adaptive stimulus towards adipogenesis, with a subsequent increase in weight gain leading to obesity.

Here we sought reference chemicals specific to unique targets within specified molecular pathways, whilst also considering the broader IATA development that is critical for regulatory acceptance of these assays. Due to the higher complexity of the hMSC functional assay compared to molecular-level nuclear receptor transactivation assays, the relatively longer duration, and associated higher implementation cost, we recommend a smaller set of well-characterised prototypical chemicals, including limited pharmaceuticals, as a first tier of adipogenesis test method optimisation and proficiency testing, to demonstrate the robust performance of an assay. Subsequently, a larger chemical set, including more borderline or weakly active chemicals is introduced once the test method is more mature, as a second tier of test method optimisation and in preparation for (pre-)validation testing.

Such an approach is becoming increasingly advocated, given the recent regulatory acceptability difficulties for validated *in vitro* assays that do not have an IATA or guidance as to how to use them (*c.f. in vitro* CYP induction test method (13)), and EU regulatory concerns with respect to industry dossier submissions considering peroxisome proliferation and putative lack of relevance to human toxicity (229, 255, 256), including liver tumour formation (257). Some aspects of this, in relation to cell proliferation can already be ascertained in standard *in vivo* toxicity testing for pesticides and pharmaceuticals (e.g., in OECD TG 407/408) (258).

The provision of this minimum set of literature-supported proficiency chemicals will facilitate the development of regulatory relevant test methods for metabolic disruption in relation to obesity. The methodological considerations, in accordance with OECD Good *In Vitro* Method Practices (4), for the selection of chemicals will also support the chemical selection for other (*in vitro*) test methods, related to adverse outcomes consequent to metabolic disruption.

## 4 Discussion

Here we have described the evidence-based selection of suitable chemicals for the development and validation of PPARα and PPARγ as molecular-level events, and white adipose tissue adipogenesis as an apical organ/tissue-level adverse effect for obesity. In order to facilitate further characterisation and proficiency testing of three relevant *in vitro* test methods that can assess the MIE of the hPPARα and hPPARγ transactivation (luciferase-based receptor transactivation test methods using HG5LN GAL4-PPAR cell lines (59, 259) towards perturbed adiposity (hMSC primary cell test method). Understanding adipocyte biology is essential for understanding the pathophysiological basis of obesity and related metabolic diseases (such as type 2 diabetes). From this understanding, one can elucidate and support the development of suitable and more human relevant *in vitro* test methods towards become validated Test Guidelines.

By moving these test methods further along the road towards validation, the intention is to ultimately support regulatory applications in the assessment of chemical hazards towards the adverse health outcome of obesity/metabolic disruption, and to facilitate progress towards test guideline and IATA development. The initial list of proposed proficiency chemicals was further supported with review by international experts in regulatory test method development. However, it is acknowledged that while the PPARα to a lesser degree, and PPARγ in particular are key coordinators in the adipogenesis metabolic axis, this is not the entire mechanistic picture towards adverse adipogenicity leading to obesity (260–262). White adipocytes play essential roles in energy storage, insulation, and hormone regulation. Mature white adipocytes serve as the primary site for energy storage in the form of triglycerides. There is little cellular turnover, they expand, and they are very long-lived. A proliferating pool of MSCs and preadipocytes are the source of differentiated adipocytes that need to be created in a balanced manner to accommodate both reduced and excess energy intake. Effective means to regulate the differentiation of new adipocytes so that the number of cells balance the lipid storage need is essential, and C/EBPβ has been identified to be a critical regulatory switch controlling the transcriptional activation potential of preadipocytes and fibroblasts that can be stimulated to differentiate into mature adipocytes (25, 263). Abdou et al. (264) describe how the commitment to differentiation occurs stochastically within different cell cultures, with increasing strength of adipogenic stimulus leading to the commitment of increasing numbers of preadipocytes to differentiation and maturation. Members of the C/EBP transcription factor family are transcriptionally involved in the role of PPARγ as a primary effector of adipogenesis (25). There are further intricate mechanistic observations that are influential in capturing adversity in the mode of action landscape of the development of obesity. For example, the interactive pathways in relation to the GR (265), the liver-X receptor (LXR) and the heterodimerisation partner RXR, hepatic fibrosis, cholestasis and insulin are influential. Inflammatory cytokines (266), epigenetics and hormonal appetite control and satiety are also integral in the development and progression of obesity and metabolic syndrome. Epigenetic mechanisms, particularly histone modifications for example via HDAC1 and cofactors on C/EBPβ functions directly prior to the onset of commitment, play a contributory role in the initiation of the transcription of the commitment factors C/EBPα and PPARγ. Threshold-driven, cross-regulation of C/EBPα and PPARγ lead the cells towards terminal differentiation. In some cases, PPARγ is not the mechanistic trigger towards adipogenesis at all, as reported for example for the flame retardant dechlorane plus (267).

For another example, the antioxidant 2, 4-Di-tert-butylphenol (2,4-DTBP) increased lipid accumulation in hMSCs by activating the PPARγ-RXR heterodimer via RXRα but not directly binding to PPARγ, confirmatory evidence was shown in crystal studies of bound RXR (226). Indeed, other examples are also mediated by RXR, such as TBT, and others require further elucidation, such as phytanic acid. Receptors that also heterodimerise with RXR, such as VDR, can also be affected by RXR binding, and receptor competition for RXR heterodimerisation within a given tissue, is a broader consideration (31).

In relation to the cellular and tissue level, for adipogenesis test method models, whilst the weight of evidence appears to be stronger for the promotion of lipid accumulation in murine *in vitro* cell models (especially in 3T3-L1 cells), there is greater uncertainty in humans/human *in vitro* cell models. The adipogenicity of BPA appears to be particularly contentious. Whilst on balance, BPA is clearly positive in 3T3-L1 cells, in human primary pre-adipocytes, it is not a PPARγ agonist. Whilst BPA and BPS both induce adipogenesis, the results from Boucher et al. (88), show that BPS affects adipose specific transcriptional changes earlier than seen for BPA, and alters the expression of genes specifically related to adipogenesis and lipid metabolism (88).

Importantly, one study (89) found that in murine 3T3-derived cells, lipid accumulation was not observed without application of the “induction cocktail” *in vitro* (this was not investigated in other studies). Indeed Longo et al. and Kossotis et al. (41, 89), suggest that overall, the murine 3T3-L1 cell line is a useful but less relevant model for human adipogenesis, as mechanistically, they are already initiated/committed to the adipocyte lineage, and there is variability across batches and reproducibility issues between laboratory strains.

With respect to regulatory relevance for human health, for the evaluation of potential obesity related hazard(s), the essential points for clarification include the differences reported for different adipogenesis cell models in particular. These are most likely also explained by differences in initiated states and different protocols and induction methods. Independent HTS assessments of data generated by ToxCast and Tox21 in relation to metabolic disruption have found these to be inadequate with respect to reliability (160), and whilst there is great utility in HTS for the purposes of prescreening, for (pre)validation purposes with respect to the objective of the current exercise, they were not considered to contribute substantially to the WoE. However subsequent independent assessments are reported to improve ToxPi predictability (174), but as explained in section 2.4 this 3T3-L1 data could only be utilised as supporting information (160).

This exercise has been one of the more challenging with respect to recommendations for the selection of chemicals for test methods that are intended for a metabolic disruption IATA, here in relation to obesity, compared to single endpoints. Overall, a reasonable number of negatives and potential reference/proficiency chemicals with a range of activity have been elucidated from the literature and recommendations for (pre-)validation purposes of the individual test methods can be made, but also with consideration of their inclusion in an IATA. To that end, chemicals from the list generated herein are being selected and implemented in pre-validation studies of hPPARα and hPPARγ transactivation assays (59) and hMSC cells in the GOLIATH project. Further work is being taken up within other related projects, such as PARC, in relation to for example the testing of the proposed chemicals in a higher throughput 96 well format for the hMSC test method, and will be proposed in relevant follow-up projects, also with the intention of contributing to the OECD detailed review paper on metabolic disruption underway. Other relevant mechanisms, such as retinoid and PPARδ-dependent neurite outgrowth assay and hGR activation, are being (pre) validated by the PEPPER platform. The intention is that these test methods can ultimately be proposed as draft TGs, to be included in IATAs and ultimately define approaches, when fully validated in the future.

Collectively this work will contribute to the development of the OECD TG NAM toolbox to assess the potential chemical hazards from obesogenic chemicals. By contributing to a better mechanistic understanding of the contribution of chemical toxicity to the global obesity epidemic, we will also be able to support the development of safer-by-design chemicals and the future development of relevant endocrine modality chemical regulations, to better protect public health.

## Author contributions

EO: Data curation, Investigation, Writing – original draft, Writing – review & editing. BK: Data curation, Investigation, Methodology, Writing – original draft, Writing – review & editing. MJ: Conceptualization, Data curation, Funding acquisition, Investigation, Methodology, Project administration, Resources, Supervision, Validation, Visualization, Writing – original draft, Writing – review & editing.

## Supplementary material

Supplementary Material 1: Table 1. Details on PubMed literature search and its results (date: 2020 and Dec 2023).

Supplementary Material 2: Table 1. Detailed overview of the long list of literature considered with respect to the chemical selection for the pre-validation of the hPPARα, hPPARγ transactivation and hMSC adipogenesis test methods.

Supplementary Material 2: Table 2. Summary of other cell types and non-selected chemicals for the pre-validation of the hPPARα, hPPARγ transactivation, and hMSC adipogenesis test methods.
