# Erratum: Addressing chemically-induced obesogenic metabolic disruption: selection of chemicals for *in vitro* human PPARα, PPARγ transactivation, and adipogenesis test methods

**DOI:** 10.3389/fendo.2024.1491855

**Published:** 2024-10-25

**Authors:** Frontiers Production Office

**Affiliations:** Frontiers Media SA, Lausanne, Switzerland

**Keywords:** adipogenesis, obesogen, peroxisome proliferator-activated receptor, metabolic disruption, integrated testing strategy, test guideline, validation

Due to a production error, there was a mistake in **Tables 2A–C** as published. The molecular structures were incorrectly placed in these tables. The corrected **Tables 2A–C** appear below.

Table 2ASelected chemicals and activity bands for the PPARα assay.

**Table:** 

Chemical	Cas No.	Structure	Use	hPPARα ag/antag ● inactive ○
Negative				
**Bisphenol A (BPA)**	80-05-7	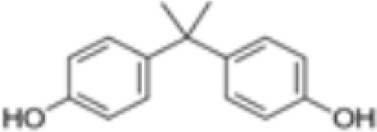	Plasticiser, industrial chemical	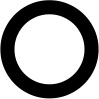 -
**Triphenylphosphate (TPP)**	115-86-6	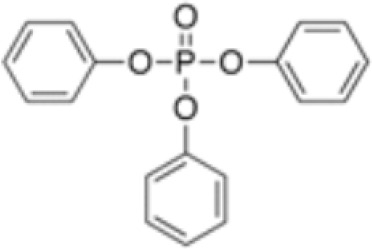	Industrial chemical	** 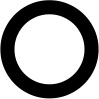 ** -
**Dichlorodiphenyldichloroethylene** **(pp’-DDE)**	72-55-9	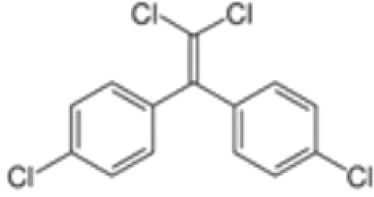	Pesticide metabolite (Stockholm POPs list)	** 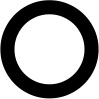 ** -
**Triclosan (TCS)**	3380-34-5	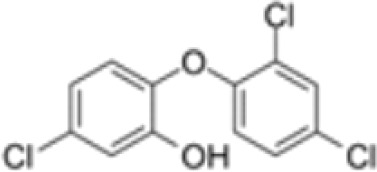	Bacteriocide	** 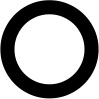 ** -
**Rosiglitazone (ROSI)**	122320-73-4	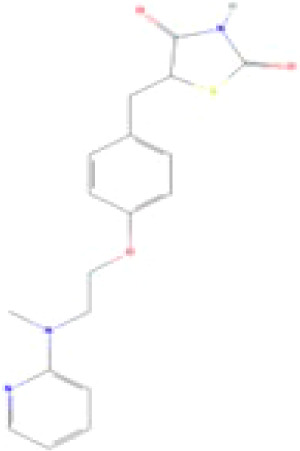	Pharmaceutical	** 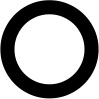 ** **-**
**Chlorpyrifos (CPF)**	2921-88-2	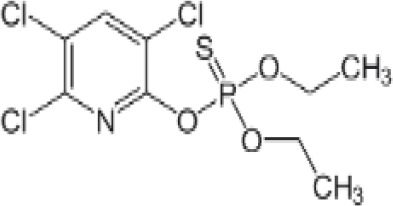	Organophosphate pesticide	** 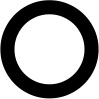 ** -
**Perfluorohexanoic acid (PFHXA)**	307-24-4	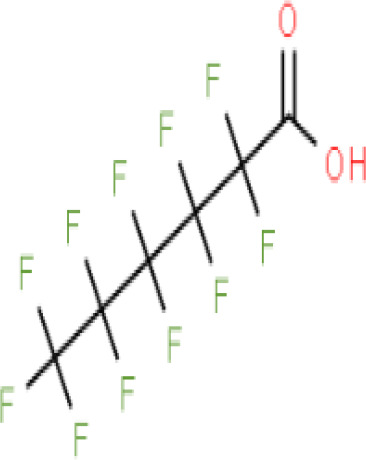	Breakdown product of PFAS	** 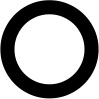 ** **-**
**(aR)-4-chloro-a-[3-(trifluoromethyl)phenoxy]benzeneacetic acid, (MBX-102/JNJ39659100)** **Arhalofenate** **MBX-102**	24136-23-0	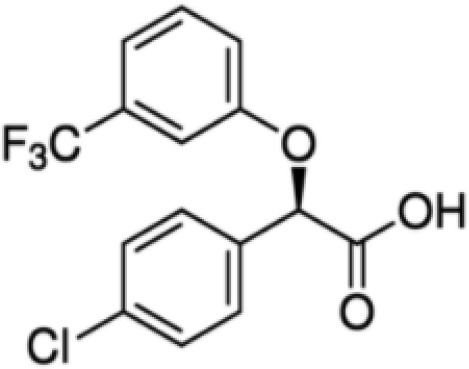	Experimental pharmaceutical	** 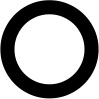 ** **-**
**Tetrabrominated BPA (TBBPA)**	79-94-7	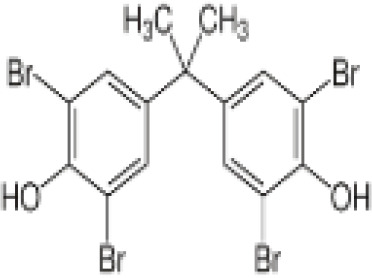	Flame retardant	** 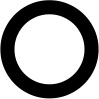 ** -/?
**LGD1069 (Targretin) Bexarotene**	153559-49-0	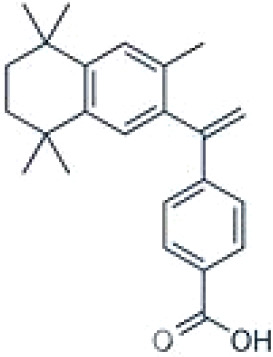	Pharmaceutical	** 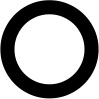 ** **-**
Weak activity				
**Phytanic acid**	14721-66-5	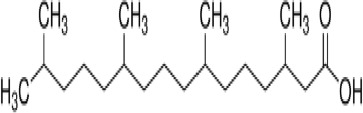	Dietary lipid	** 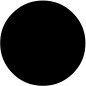 ** + Very weak agonist 10^-4^ µM
**Clofibrate**	637-07-0	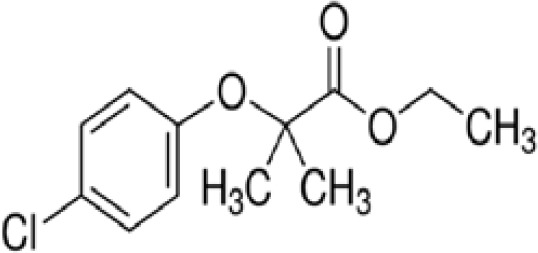	Pharmaceutical, fibrate	** 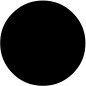 ** + Weak-moderate agonist up to 10^-4^ µM
**AGN194204 (IRX4204)**	220619-73-8	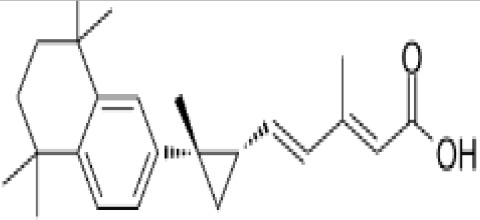	Pharmaceutical	** 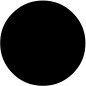 ** Weak antagonist
Weak to moderate activity				
**Mono-(2-Ethylhexyl) Phthalate (MEHP)**	4376-20-9	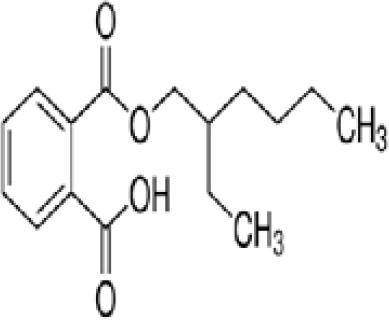	Phthalate, plasticiser	** 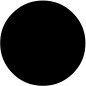 ** + Moderate agonist
**Eicosapentaenoic acid (EPA)**	10417-94-4	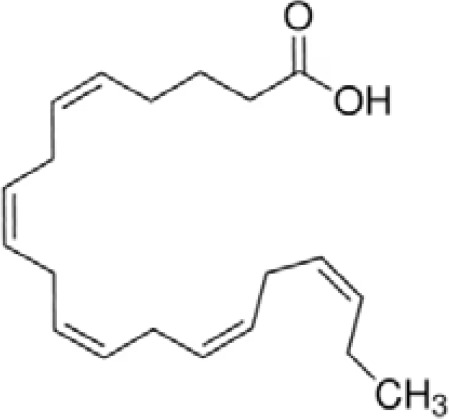	Nutrient, long chain PUFA essential fatty acid	** 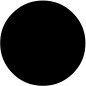 ** + Moderate agonist
**Tesaglitazar/AZ242**	251565-85-2	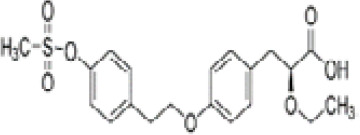	Pharmaceutical	** 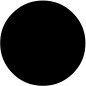 ** + Selective moderate agonist 3 µM
**Clofibric acid**	882-09-7	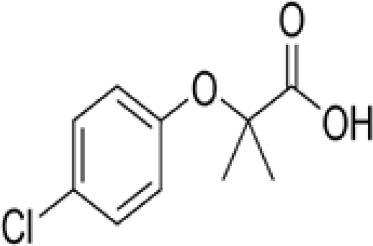	Herbicide and pharmaceutical; active metabolite of clofibrate	** 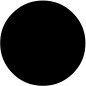 ** + Moderate agonist (EC_50_ = 50.0 µM)
Strong activity				
**Perfluorooctanoic acid (PFOA)**	335-67-1	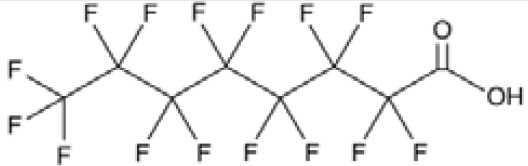	Industrial chemical, non-stick coating	** 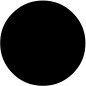 ** + Strong agonist
**Pristanic acid**	1189-37-3	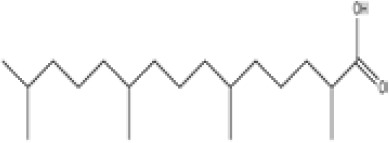	Dietary lipid	** 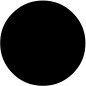 ** + Strong agonist 1 µM
**Docosahexaenoic acid (DHA)**	6217-54-5	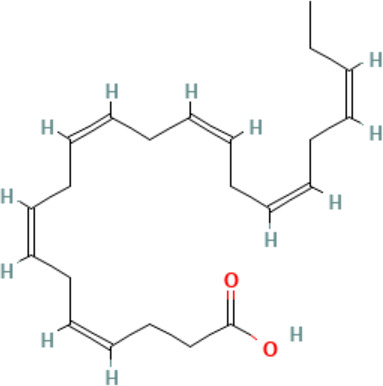	Nutrient, long chain PUFA essential fatty acid	** 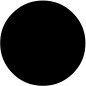 ** + Strong agonist
Positive control				
**GW7647**	265129-71-3	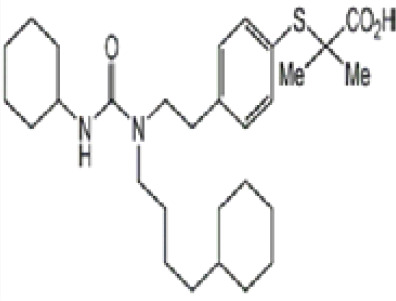	Pharmaceutical candidate	** 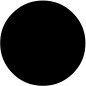 ** + Selective agonist, positive control10 nM

**Table 2B:** Selected chemicals and activity bands for the PPARγ assay.

Chemical	Cas No.	Structure	Use	hPPARγ ag/antag ● inactive ○
Negative				
**Bisphenol A (BPA)**	80-05-7	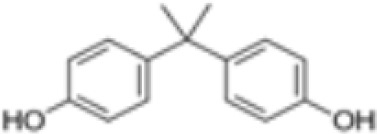	Plasticiser, industrial chemical	** 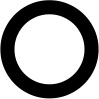 ** -
**Dichlorodiphenyldichloroethylene** **(pp’-DDE)**	72-55-9	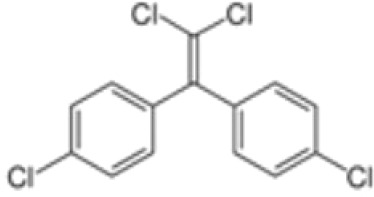	Pesticide metabolite (Stockholm POPs list)	** 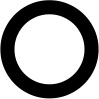 ** -
**Triclosan (TCS)**	3380-34-5	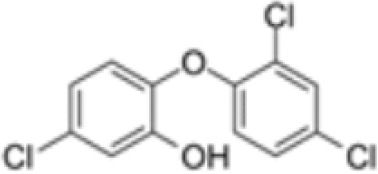	Bacteriocide	** 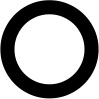 ** -
**Perfluorohexanoic acid (PFHXA)**	307-24-4	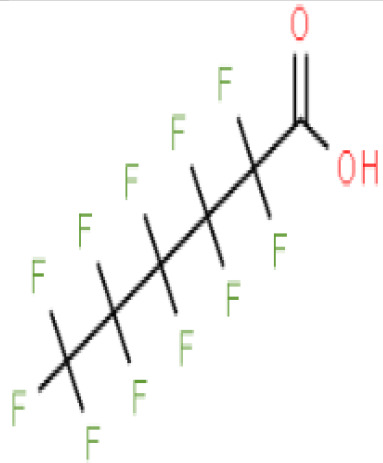	Breakdown product of PFAS	** 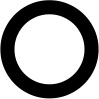 ** **-**
**LGD1069 (Targretin) Bexarotene**	153559-49-0	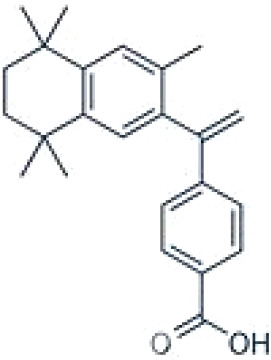	Pharmaceutical	** 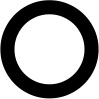 ** -
Weak activity				
**Chlorpyrifos (CPF)**	2921-88-2	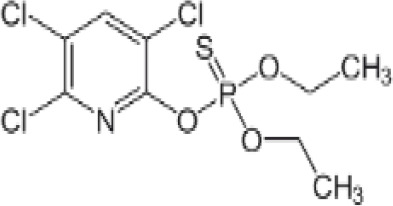	Organophosphate pesticide	** 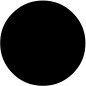 ** + Weak agonist
**Clofibrate**	637-07-0	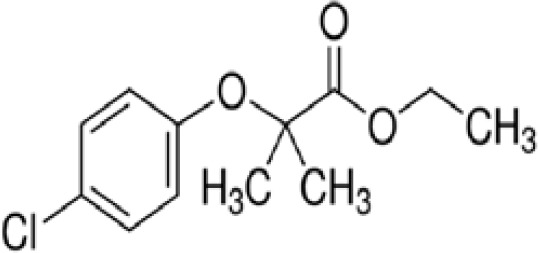	Pharmaceutical, fibrate	** 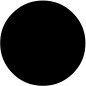 ** + Weak-moderate agonist
**Phytanic acid**	14721-66-5	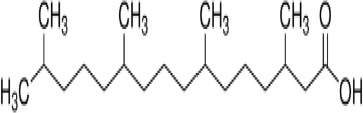	Dietary lipid	** 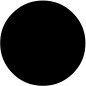 ** + Weak-moderate agonist
**(aR)-4-chloro-a-[3-(trifluoromethyl)phenoxy]benzeneacetic acid, (MBX-102/JNJ39659100)** **Arhalofenate** **MBX-102**	24136-23-0	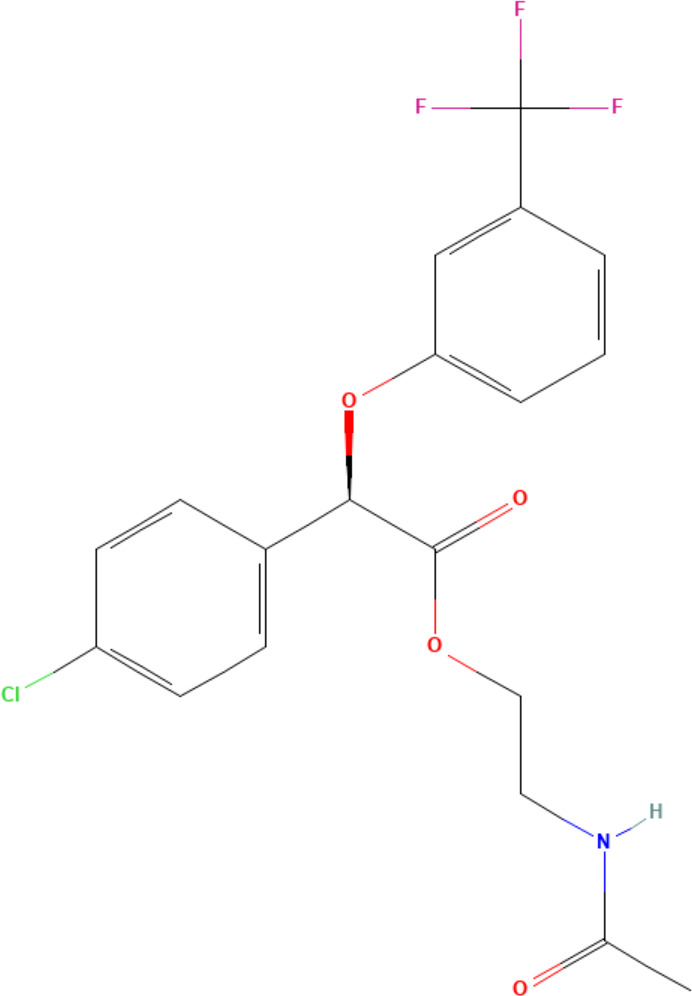	Experimental pharmaceutical	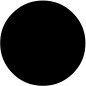 + Weak agonist
**GW3965 hydrochloride**	405911-17-3	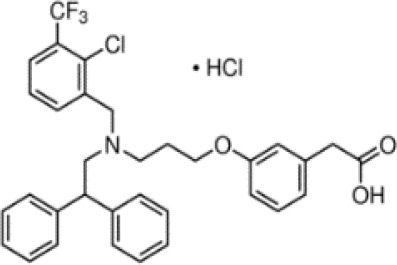	Pharmaceutical candidate	** 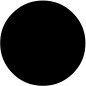 ** + Weak agonist
Weak to moderate activity				
**Mono-(2-Ethylhexyl) Phthalate (MEHP)**	4376-20-9	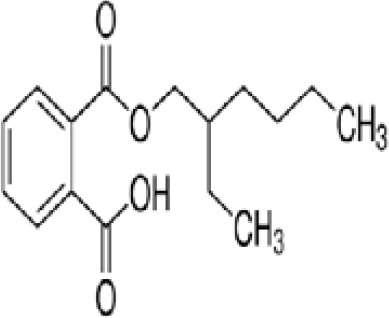	Phthalate, plasticiser	** 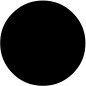 ** + Moderate agonist
**GW7647**	265129-71-3	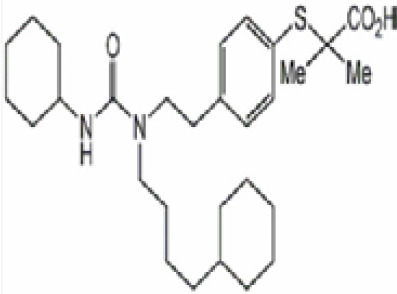	Pharmaceutical candidate	** 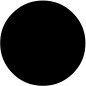 ** + Moderate agonist
**Eicosapentaenoic acid (EPA)**	10417-94-4	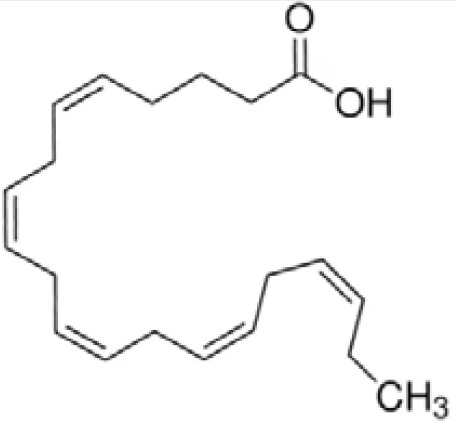	Nutrient, long chain PUFA essential fatty acid	** 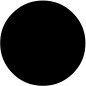 ** + Moderate agonist
**Clofibric acid**	882-09-7	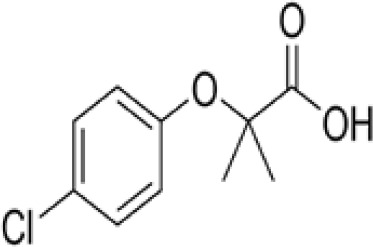	Herbicide and pharmaceutical; active metabolite of clofibrate	** 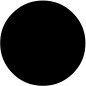 ** + Weak-Moderate agonist (but weaker than PPARα)
**Pristanic acid**	1189-37-3	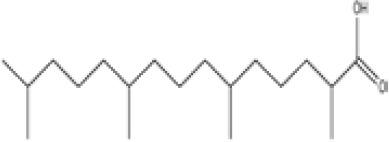	Dietary lipid	** 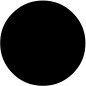 ** + Weak-moderate agonist 10 µM
Strong activity				
**Triphenyl phosphate (TPP)**	115-86-6	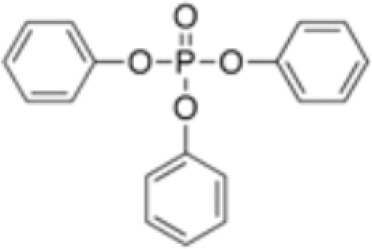	Industrial chemical: Adhesives and sealants, coating products, cosmetics and personal care products	** 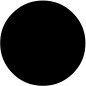 ** + Strong agonist
**Docosahexaenoic acid (DHA)**	6217-54-5	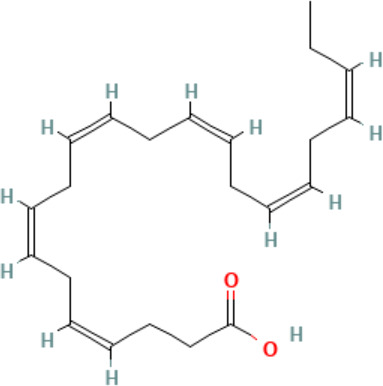	Nutrient, long chain PUFA essential fatty acid	** 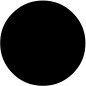 ** + Strong agonist
**Tetrabrominated BPA (TBBPA)**	79-94-7	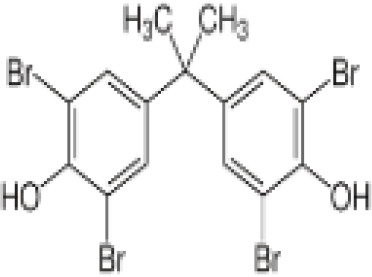	Flame retardant	** 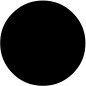 ** + Strong agonist
**Perfluorooctanoic acid (PFOA)**	335-67-1	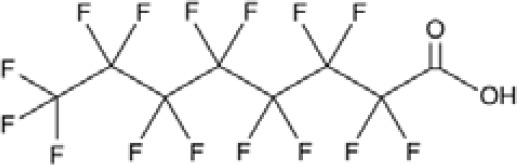	Industrial chemical, non-stick coating	** 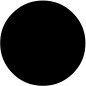 ** + Strong agonist
**15-Deoxy-Δ12,14-prostaglandin J2 (15d-PGJ2)**	87893-55-8	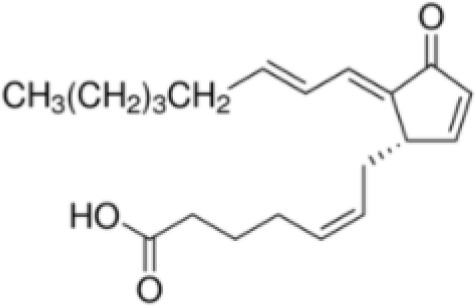	Metabolite of endogenous prostaglandin (PGJ2)	** 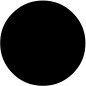 ** + Strong agonist
**Tesaglitazar/** **AZ 242**	251565-85-2	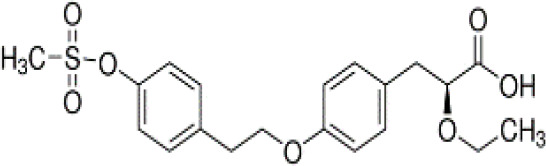	Pharmaceutical	** 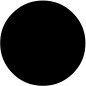 ** + Strong agonist 40 nM
Positive control				
**Rosiglitazone (ROSI)**	122320-73-4	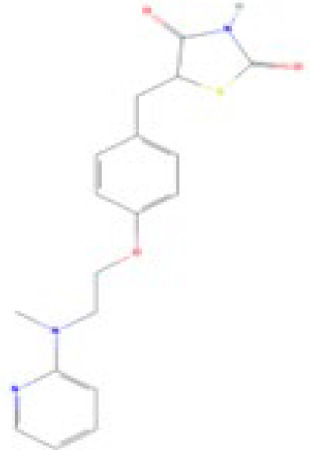	Pharmaceutical	** 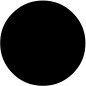 ** **+ Positive control**

**Table 2C:** Selected chemicals and activity bands for the hMSC adipogenesis assay.

Chemical	Cas No.	Structure	Use	hMSC adipogenesis (lipid accumulation) ag/antag ● inactive ○
Negative				
**Triclosan (TCS)**	3380-34-5	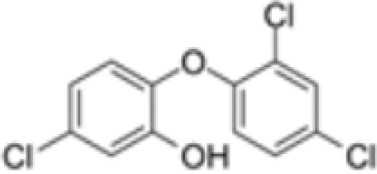	Bacteriocide	** 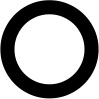 ** -
**TTNPB, 4-[(E)-2-(5,6,7,8-Tetrahydro-5,5,8,8-tetramethyl-2-naphthalenyl)-1-propenyl] benzoic acid, Arotinoid acid**	71441-28-6	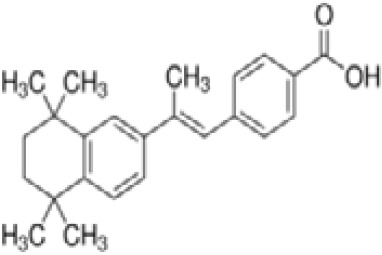	Pharmaceutical	** 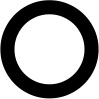 ** - Strong inhibitor of adipogenesis; unlike retinoic acids (9cRA) that promotes adipogenesis
**Dichlorodiphenyldichloroethylene** **(pp’-DDE)**	72-55-9	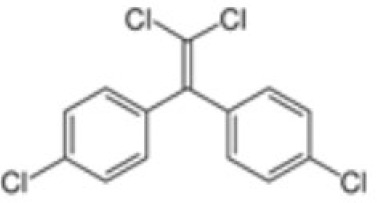	Pesticide metabolite (Stockholm POPs list)	** 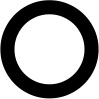 ** ?-
**Chlorpyrifos (CPF)**	2921-88-2	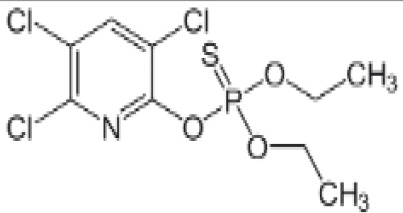	Organophosphate pesticide	** 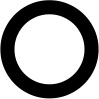 ** -
Weak activity				
**Perfluorooctanoic acid (PFOA)**	335-67-1	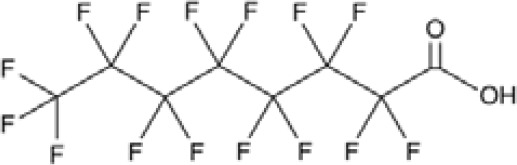	Industrial chemical, non-stick coating	** 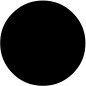 ** + Weak inducer
Weak to moderate activity				
**GW3965 hydrochloride**	405911-17-3	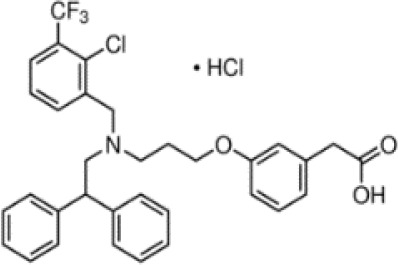	Pharmaceutical candidate	** 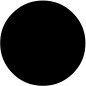 ** + Weak/moderate inducer
**Tetrabrominated BPA (TBBPA)**	79-94-7	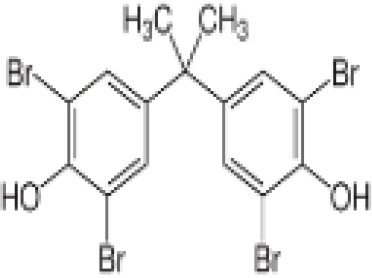	Flame retardant	** 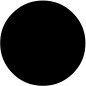 ** +Moderate inducer 10 μM induced adipogenesis in 3T3-L1 cells
**Mono-(2-Ethylhexyl) Phthalate (MEHP)**	4376-20-9	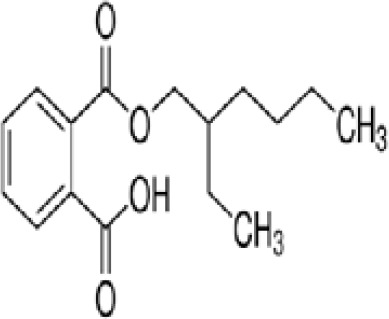	Phthalate, plasticiser	** 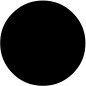 ** + Moderate 10 μM and was maximal at 100 μM
**Triphenyl phosphate (TPP)**	115-86-6	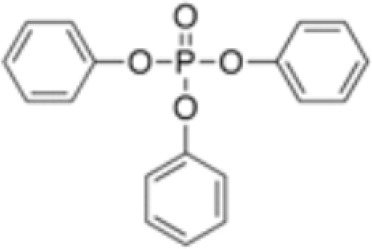	Industrial chemical: Adhesives and sealants, coating products, cosmetics and personal care products	** 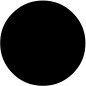 ** + Agonist at high dose (>1µM)
Strong activity				
**Fludioxonil**	131341-86-1	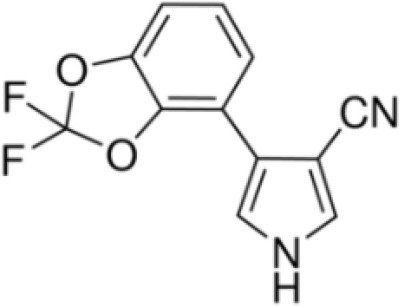	Non-systemic fungicide	** 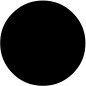 ** + Strong (significant at 0.2 µM)
**Tributyltin (TBT) chloride**	1461-22-9	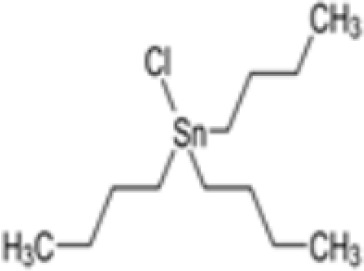	Fungicide	** 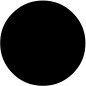 ** + Strong inducer adipogenic differentiation (3T3-L1)
Positive control				
**Rosiglitazone (ROSI)**	122320-73-4	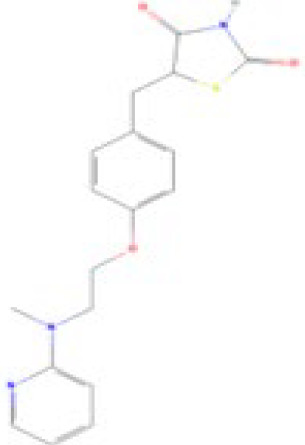	Pharmaceutical	** 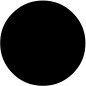 ** **+ Positive control**

The publisher apologizes for this mistake.

The original version of this article has been updated.

