# Supplementary material for: Addressing chemically-induced obesogenic metabolic disruption: selection of chemicals for in vitro human PPARα, PPARγ transactivation, and adipogenesis test methods
Source: Front Endocrinol (Lausanne). 2024 Jul 8;15:1401120. doi: 10.3389/fendo.2024.1401120 (PMC11260640; doi:10.3389/fendo.2024.1401120)
Supplement: Supplementary Material 1: Table 1. — Details on PubMed literature search and its results (date: 2020 and Dec 2023). [file DataSheet_1.docx]

***Supplementary Material 1***

**Literature Search**

At the outset of the project, in 2020, a targeted literature review was performed, for each endpoint, utilising expert knowledge in the fields of nuclear receptor activation, adipogenesis, nutrition and metabolic disruption, together with (guidance from) highly relevant well-documented reviews, to focus and retrieve pertinent chemical and target endpoint literature evidence. This guided the literature searches for specific chemicals under consideration. The literature search was later updated and supplemented in relation to PPAR and adipogenesis obesity key events in late 2023, to include relevant recently published articles for each chemical and test method.

The authors conducted literature searches in PubMed (2020 and late 2023). User queries for literature search are “chemicals AND test methods (hPPARα, PPARγ, hMSC adipogenesis) AND obesity related key events”. Articles appearing to be obviously not relevant and also non-English language abstracts were excluded from the results list based on their titles and/or abstracts. For the remaining references, the full articles were retrieved and further assessed for relevance.

Supplementary Material 1: Table 1. Details on PubMed literature search and its results (date: 2020 and Dec 2023)

| **Chemicals** | **Literature Search (date: 2020)**  *Includes 2020 literatures as well | | | | **Literature Search (date: December 2023)**  *Includes 2021, 2022, 2023 literatures and (Burkhardt et al., 2024) added in early 2024 | | | |  |
| --- | --- | --- | --- | --- | --- | --- | --- | --- | --- |
|  | **Number of hits** | **Number of hits for which the full articles were retrieved:** | **Number of retrieved full articles excluded after detailed assessment:** | **Number of references included in the evaluation** | **Number of hits** | **Number of hits for which the full articles were retrieved:** | **Number of retrieved full articles excluded after detailed assessment:** | **Number of references included in the evaluation** | **Total number of references included in the evaluation:** |
| (aR)-4-chloro-a-[3-(trifluoromethyl) phenoxy]benzeneacetic acid, (MBX-102/JNJ39659100)  Arhalofenate  MBX-102 | 5 | 4 | 3 | 1 | 1 | 1 | 0 | 1 | 2 |
| 15-Deoxy-Δ12,14-prostaglandin J2 (15d-PGJ2) | 71 | 31 | 29 | 2 | 14 | 8 | 7 | 1 | 3 |
| 1alpha,25-Dihydroxyvitamin D3 (calcitriol)  ~~(~~Active metabolite of Vitamin D3)  (OHVitD3) | 129 | 61 | 50 | 11 | 19 | 10 | 5 | 5 | 16 |
| 3,3’,5,5’ Tetrabromobisphenol A (TBBPA) | 14 | 7 | 1 | 6 | 4 | 3 | 1 | 2 | 8 |
| AGN194204 (IRX4204) | 6 | 5 | 3 | 2 | 0 | 0 | 0 | 0 | 2 |
| Bisphenol A (BPA) | 59 | 58 | 17 | 41 + 2 regulatory agency reports | 11 | 11 | 4 | 7 | 50 |
| Chlorpyrifos (CPF) | 62 | 19 | 13 | 6+3 regulatory agency reports | 25 | 11 | 11 | 0 | 9 |
| Clofibrate | 490 | 177 | 171 | 6+1 regulatory agency reports | 9 | 6 | 4 | 2 | 9 |
| Clofibrate metabolite: Clofibric acid | 182 | 56 | 55 | 1 | 3 | 2 | 1 | 1 | 2 |
| Dichlorodiphenyl-dichloroethylene  (p,p’-DDE) | 80 | 21 | 10 | 11 | 4 | 3 | 0 | 3 | 14 |
| Docosahexaenoic acid (DHA) | 32 | 18 | 14 | 4 | 4 | 2 | 0 | 2 | 6 |
| Eicosapentaenoic acid (EPA) | 13 | 8 | 4 | 4 | 1 | 1 | 1 | 0 | 4 |
| Fludioxonil | 5 | 3 | 0 | 3 | 2 | 2 | 0 | 2 | 5 |
| GW3965 hydrochloride | 48 | 27 | 21 | 6 | 11 | 7 | 6 | 1 | 7 |
| GW7647 | 10 | 10 | 4 | 6 | 3 | 3 | 2 | 1 | 7 |
| LGD1069 (Targretin) Bexarotene | 33 | 21 | 19 | 2 | 11 | 9 | 7 | 2 | 4 |
| Mono-(2-Ethylhexyl) Phthalate (MEHP)  DEHP metabolite | 51 | 50 | 33 | 17 | 25 | 25 | 23 | 2 | 19 |
| Perfluorohexanoic acid (PFHXA) | 4 | 4 | 3 | 1+1 regulatory agency reports | 6 | 6 | 5 | 1 | 3 |
| Perfluorooctanoic acid (PFOA) | 115 | 28 | 21 | 7 | 61 | 35 | 32 | 3 | 10 |
| Phytanic acid | 54 | 23 | 15 | 8 | 5 | 3 | 3 | 0 | 8 |
| Phytanic acid metabolite: Pristanic acid | 16 | 9 | 4 | 5 | 1 | 1 | 1 | 0 | 5 |
| Rosiglitazone (ROSI) | 343 | 176 | 170 | 6 | 11 | 6 | 4 | 2 | 8 |
| Tesaglitazar/  AZ 242 | 3 | 2 | 0 | 2 | 1 | 1 | 0 | 1 | 3 |
| Tributyltin (TBT) chloride | 109 | 32 | 18 | 14 | 15 | 8 | 6 | 2 | 16 |
| Triclosan (TCS) | 62 | 18 | 7 | 11 | 13 | 8 | 1 | 7 | 18 |
| Triphenyl phosphate (TPP) | 16 | 10 | 9 | 1 | 16 | 9 | 6 | 3 | 4 |
| TTNPB, 4-[(E)-2-(5,6,7,8-Tetrahydro-5,5,8,8-tetramethyl-2-naphthalenyl)-1-propenyl] benzoic acid, Arotinoid acid | 29 | 5 | 4 | 1 | 1 | 1 | 1 | 0 | 1 |
