# Supplementary material for: Addressing chemically-induced obesogenic metabolic disruption: selection of chemicals for in vitro human PPARα, PPARγ transactivation, and adipogenesis test methods
Source: Front Endocrinol (Lausanne). 2024 Jul 8;15:1401120. doi: 10.3389/fendo.2024.1401120 (PMC11260640; doi:10.3389/fendo.2024.1401120)
Supplement: Supplementary Material 2: Table 1. — Detailed overview of the long list of literature considered with respect to the chemical selection for the pre-validation of the hPPARα, hPPARγ transactivation and hMSC adipogenesis test methods. [file DataSheet_2.docx]

***Supplementary Material 2***

**Supplementary Table 1. Detailed overview of the long list of literature considered with respect to the chemical selection for the pre-validation of the hPPARα, hPPARγ transactivation and hMSC adipogenesis test methods.** Unless stated differently, p ≤ 0.05 in considered statistically significantly different. *ag: agonist, antag: antagonist.*

| **Chemical** | **Cas No.** | **Structure** | **Use** | **Model**  **and test chemical concentrations**  **Duration of exposure** | **hPPARα ag/antag** | **hPPARγ ag/antag** | **hMSC adipogenesis (lipid accumulation)** | **References** | **Edited extracts from original papers with summary conclusions** |
| --- | --- | --- | --- | --- | --- | --- | --- | --- | --- |
| **Bisphenol A (BPA)** | 80-05-7 | 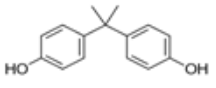 | Plasticiser industrial chemical | **SUMMARY** | No/ possibly weak agonism at high concentrations, of e.g. 10 μM. | No | Uncertain/ weak positive |  | BPA (and potentially some of its substitutes) inhibit important metabolic pathways and insulin signalling in adipocytes under environmentally relevant, low concentrations. It is considered to be related to PPARγ activity leading to increased adipose tissue, which may increase metabolic syndrome development risk, including insulin resistance. However, PPARα and PPARγ activation is unlikely to be solely responsible for observed adipogenic effects, if present. More likely to be mediated via ERα and ERβ agonism, non-classical Estrogen pathways and or other non-PPAR signalling pathways (Alonso-Magdalena et al., 2012; Babiloni-Chust et al., 2022; Martinez-Pinna et al., 2019) and on this basis, it is likely that there are sex specific effects.  Adipogenesis: weight of evidence is stronger for induction of lipid accumulation in murine *in vitro* cell models (especially in 3T3-L1 cells), with higher uncertainty in humans/ human *in vitro* cell models. Importantly, one study found that in murine 3T3-derived cells lipid accumulation was not observed without application of the “induction cocktail” *in vitro* (Longo et al., 2020)*,* not investigated in other studies. The murine 3T3 L1 cells however are less relevant for human adipogenesis, as mechanistically, they are already induced. Insulin availability is also causal (Atlas et al., 2014). |
|  |  |  |  | *In vitro,* 3T3-L1 murine cell line,  BPA (10 μM, 1 μM, 0.1 μM, 0.01 μM, 0.001 μM)  12 d |  |  | Uncertain | (Kassotis et al., 2021) | Interlaboratory reproducibility evaluation in 3T3-L1 cell lines by 10 laboratories in 5 different countries. Triglyceride accumulation activity and pre-adipocyte proliferation for ROSI (positive control) was 90-100 and 30-70% and for BPA 80-100 and 20-40%, respectively. |
|  |  |  |  | *In vitro,* 3T3-L1 murine cell line,  BPA (0.01 μM, 0.1 μM, 1 μM, 25 μM, 50 μM)  BPS (0.01 μM, 0.1 μM, 1 μM, 25 μM, 50 μM)  8 d |  |  |  | (Ahmed & Atlas, 2016) | Lipid accumulation, mRNA and protein expression of key adipogenic markers increased in 1-50 μM BPS induced 3T3-L1 cells (P<0.05).  Adipogenic markers expression and lipid accumulation was  higher with BPS treatment compared to BPA.  BPS exposure upregulated lipoprotein lipase, adipocyte protein 2, PPARγ, perilipin, adipsin and CCAAT/enhancer-binding protein alpha mRNA expression levels. PPARγ was reported to be activated 1.5-fold by BPS and BPA using PPRE-dependent luciferase (P<0.05).  BPS was able to competitively inhibit ROSI-activated PPARγ, while BPA did not suggesting that BPS interacts with PPARγ differently.  Simultaneous treatment with selective PPARγ antagonist GW9662 resulted in inhibition of BPS-, BPA-, ROSI- but not dexamethasone-dependent adipogenic differentiation.  BPA and BPS were observed to increase 3T3-L1 adipocyte in a concentration-depent manner. With respect to PPARγ induced adipogenesis. BPS is more potent adipogen in 3T3-L1 murine cell line compared to BPA. |
|  |  |  |  | Primary pre-adipocyte cell cultures  BPA (10 nM and 10 μM)  10 d |  |  |  | (Verbanck et al., 2017) | Significantly adipocyte differentiation/deregulation of mRNA/lncRNA and miRNA at low and high concentrations observed. Human primary adipocytes chronically exposed to BPA, BPS and BPF over 10 days were impacted during differention at low concentrations at the transcriptome level.  Interaction with non-coding RNAs (miRNA, long non-coding and small nucleolar RNAs) suggested the effect of the BPA, BPS and BPF on the gene transcription regulation and post-transcriptional mRNA processing. Impaired cellular events were observed at both the lower and higher concentrations. |
|  |  |  |  | Human preadipocytes of the Simpson-Golabi-Behmel syndrome (SGBS) cells  BPA (10 nM, 100 nM, 1 µM, or 10 µM)  12 d |  |  |  | (Schaffert et al., 2021) | Human preadipocyte cells were treated with BPA, BPS, BPB, BPF, BPAF, and GW9662 (PPARγ-antagonist) for differentiation.  Lipid production and global protein profiles were assessed via Oil Red O staining and LC-MS/MS-based proteomics respectively, after 12 days, exposure. All tested bisphenols bound to human PPARγ with similar efficacy as the natural ligand 15d-PGJ2 in vitro and provoked an antagonistic effect on PPARγ. All investigated bisphenols reduced lipid production during the human preadipocyte differentiation.  Adipogenesis and metabolic pathways were down-regulated. Pro-inflammatory pathways were up-regulated, MCP1 release was increased, and adiponectin decreased.  According to pAKT/AKT ratios measured, BPA, BPB and BPS significantly reduced insulin sensitivity upon insulin stimulation. |
|  |  |  |  | Murine mesenchymal stem cell line C3H/10T1/2 and embryonic stem cells (CGR8)  BPA (10 nM and 10 μM)  Undifferentiated growth (0-6 days)  Induction (6-8 days)  Terminal differentiation (8-12 days) |  |  |  | (Biemann et al., 2012) | BPA, DEHP and TBT were reported to affect the adipogenic differentiation of murine mesenchymal stem cells (MSC, C3H/10T1/2) and cell determination in a concentration-, stage- and compound-specific manner, including undifferentiated growth, adipogenic induction and terminal adipogenic differentiation. The final amount of differentiated adipocytes, cellular triglyceride content and mRNA expression of adipogenic marker genes (adiponectin, FABP4, PPARγ2, LPL) were quantified and compared with corresponding unexposed controls. BPA (10 μM) decreased subsequent adipogenic differentiation of MSC, when cells were exposed during undifferentiated growth. Final differentiated adipocyte amount was compared to unexposed cells by measuring cellular triglyceride content and mRNA expression of adipogenic marker genes (adiponectin, FABP4, PPARγ2, LPL).  Subsequent MSC adipogenic differentiation was reduced when cells were treated with 10 μM BPA during undifferentiated growth.  Undifferentiated murine embryonic stem cell treatment with investigated EDC did not reveal any change on subsequent adipogenic differentiation. |
|  |  |  |  | Human Subcutaneous Adipocytes and *in vitro,* (3T3-L1 murine cell line),  BPA (1nM and 100nM)  Adipogenesis phases: early (day 2), medium (day 6) and late (day 10) |  |  |  | (Valentino et al., 2013) | Insulin-induced glucose utilization is blocked significantly by 1nM BPA, without affecting adipocyte differentiation.  BPA exposure did not differantiate mRNA levels of PPARγ and GLUT4 adipogenic indicators.  Receptor phosphorylation and signalling activated by insulin was inhibited with BPA exposure.  With adipocyte incubation due to BPA exposure, there was an increase in IL-6 and IFN-γ and activation of JNK, STAT3 and NFkB pathways determined by multiplex ELISA analyses.  Cells exposed to SP600125, a JNK inhibitor, almost completely reversed the effect of BPA on insulin signalling and glucose utilization.  *Low dose BPA causes deterioration of adipose cell function by affecting inflammatory/insulin signalling pathways.  Adipocytes may combine BPA signals and cause further impairment of insulin sensitivity and inflammatory pathways. |
|  |  |  |  | *In vitro,* 3T3-L1 murine cell line and Human *in vitro*, (HepG2 cell line)  BPA (1 fM, 1 pM, 1 nM, 1 µM)  10 d |  | ag/antag |  | (Héliès-Toussaint et al., 2014) | BPA and BPS treatment increased lipid content in the 3T3-L1 cells.  Although a decrease in lipolysis of adipocytes was observed after bisphenol exposure, the increase in glucose uptake and leptin production was observed only with BPS exposure.  While these effects are associated with the modulation of SREBP-1c, PPARγ, aP2 and ERRα and γ genes after BPA exposure, it is noteworthy that BPS targets PGC1a and ERRγ genes.  According to these results BPA and BPS could be involved in obesity and steatosis processes through different metabolic pathways.  It is not possible to determine if BPA modulated insulin  sensitivity (no effect on glucose uptake).**Comment of (Le Magueresse-Battistoni et al., 2018). |
|  |  |  |  | *In vitro,* 3T3-L1 murine cell line,  BPA (10, 50 and 80 μM)  10 d |  |  |  | (Biasiotto et al., 2016) | BPA promoted adipocytes differentiation at the concentration of 50 and 80 μM. BPA effect in 3T3-L1 cells was associated to the specific activation of the ERα in undifferentiated cells and the ERβ in differentiated cells. BPA also activated the PPARγ upregulating a minimal 3XPPARE luciferase reporter and the PPARγ-target promoter of the aP2 gene in adipose cells, while it was not effective in preadipocytes.  Unusual *in vitro* protocol, as the initial plating of cells is very low with 103 cells per 12-well culture plate (instead of 50,000 cells).**Comment of (Le Magueresse-Battistoni et al., 2018). |
|  |  |  |  | Primary human adipocytes BPA-G (0.05µМ, 0.25 µМ)  14 d  *In vitro,* 3T3L1 murine preadipocytes  BPA-G (0.01 µМ, 0.25 µМ, 1 µМ and 10µМ)  8 d |  |  | Yes? | (Boucher et al., 2015) | 3T3L1 preadipocytes treated with 10 μM BPA-G.  Significant increase in lipid accumulation, mRNA expression of the adipogenic markers sterol regulatory element binding factor 1 (SREBF1) and lipoprotein lipase (LPL), and protein levels of LPL, aP2, and adipsin with BPA-G induction.  BPA-G exposure of primary human preadipocytes increased adipogenesis in primary human preadipocytes as detected by aP2 levels.  While exposure of cells to the ER antagonist fulvestrant (ICI) had no effect, BPA-G-induced increase in LPL and aP2 levels was significantly inhibited with co-treatment. No significant estrogenic activity was detected with BPA-G exposure.  BPA-G induces adipocyte differentiation and it is more than an inactive metabolite.  BPA-G induces adipogenesis and is inhibited by an ER antagonist but does not exhibit estrogenic activity. This shows that BPA-G does not have a classical ER transcriptional activation function and acts through an undefined pathway.  The concentration of 10 µM is several orders of magnitude higher than what would be expected in the general population, but is relevant for classification and labeling/ hazard assessment. Evidence that deconjugation of BPA-G did not occur in the in vitro models was not conclusive.**Comment of Le Magueresse-Battistoni et al. 2018 |
|  |  |  |  | *In vitro,* 3T3-L1 murine cell line,  BPA (0.01nM, 0.1nM, 1nM, 10 nM)  8 d |  |  |  | (Atlas et al., 2014) | In the absence of exogenous glucocorticoids, BPA can induce 3T3-L1 cell differentiation.  Synthetic glucocorticoid (dexamethasone) is required for differentiation of 3T3-L1 cells. This effect of BPA was observed in the deficiency of this hormone.  Additionally, BPA upregulated the mRNA expression and protein levels of fatty acid binding protein (aP2), a terminal marker of adipogenesis, in 3T3-L1 cells.  mRNA or protein levels of adipogenesis modulators like  PPARγ or CCAAT enhancer binding protein (C/EBP) α were not increased with BPA exposure.  Moreover, BPA increased the expression levels of the adipogenesis aP2 marker through its effect on the transcriptional activity of C/EBPδ and the GR in its promoter. |
|  |  |  |  | *In vitro,* 3T3-L1 murine cell line,  BPA (10 nM, 1 µM)  BPF (10 nM, 1 µM)  BPS (10 nM, 1 µM)  12 d |  |  | Yes (BPA & BPS) | (Drobna et al., 2019) | BPS and BPA increased the expression of various genes required for preadipocyte differentiation in 3T3-L1 cells with 12 days of exposure. However, BPF exposure reduced the expression of many genes at late differentiation.  Lipid concentrations of BPA and BPS exposed cells were increased compared to controls and BPF exposed cells.  Male mice exposed to both highest or lowest BPF concentrations had no effects on glucose levels or glucose tolerance, while they gained less weight compared to controls. |
|  |  |  |  | Mouse *in vitro,* OP9 cell line and 3T3-L1 murine cell line, (ATCC vs. Zenbio)  BPA (1nM, 10 nM, 0.1 µM, 1 µM, 10 µM)  7 d, 10 d, and 14 d |  |  |  | (Kassotis et al., 2017) | Cell proliferation and triglyceride accumulation evaluated with several induction periods by using different tissue culture conditions.  **Cell line and source had important role on the potency and efficacy of adipogenic chemicals.**  It was suggested by gene expression analyses that the differences between OP9 and 3T3-L1 cells were due to the differential expression of nuclear receptors involved in adipogenesis. There were differences depending on 3T3-L1 cell source as well (Kassotis et al., 2017).  Depending on the cell line and test chemical, exposure altered the potence and effectiveness of the response. **Large differences in triglyceride accumulation and cell proliferation were noted with different tissue culture plate brands.**  It is also clear that both triglyceride accumulation and cell proliferation should be assessed, as chemicals acting through one mechanism or the other may be otherwise missed. While the majority of laboratories appear to utilize the ATCC 3T3-L1 cells, the provenance of these cells is questionable and discordant responses are observed between these lots and in relation to the originally isolated 3T3-L1 cells (Zenbio).  These results reveal that the choice of cell system and differentiation protocol significantly affects adipogenic chemicals detection, thereby reproducibility of these studies are also affected.  **BPA was the less effective compound** when evaluated for triglyceride accumulation (based on maximum ROSI response), showing 23% in ATCC 3T3-L1 cells, 3% in Zenbio 3T3-L1 cells, and any in OP9 cells:   \| **Cell line** \| **EC20/IC20 (μM)** \| **EC50/IC50 (μM)** \| **Efficacy (relative to ROSI)** \| \| --- \| --- \| --- \| --- \| \| ATCC 3T3-L1 \| 0.58 ± 2.2E-4 \| 2.15 ± 4.6E-3 \| **23.0% ± 2.9%** \| \| Zenbio 3T3-L1 \| N/A \| N/A \| **2.9% ± 0.3%** \| \| OP9 \| N/A \| N/A \| N/A \| |
|  |  |  |  | *In vitro,* 3T3-L1 murine cell line,  BPA (0.01 μM, 0.1 μM, 0.5 μM, 1 μM, 10 μM, 25 μM)  Day 0 to 10: whole differentiation period Day 0 to 2: early phase Day 2 to 10: Middle-late phase |  |  |  | (Pomatto et al., 2018) | Concentrations ranging from 0.01 to 25 μM was evaluated for DiNP (Di-iso-nonyl-phthalate), DiDP (Di-iso-decyl-phthalate), DEGDB (Diethylene glycol dibenzoate), or TMCP (Tri-m-cresyl phosphate).  Proadipogenic PPARγ agonist ROSI and the plasticizer BPA were used as reference chemicals.  Unlike BPA, the four plasticizers were most effective in increasing lipid accumulation when added at the mid-to-late stage of differentiation. This suggests that there are different intracellular signalling pathways for BPA compared to tested plasticizers. |
|  |  |  |  | *In vitro,* 3T3-L1 murine cell line,  BPA (0.1 μM)  3 d |  |  |  | (Sargis et al., 2010) | BPA, dicyclohexyl phthalate (DCHP), endrin, and tolylfluanidine (TF) have been determined to stimulate GR significantly without significant PPARγ activation.  All four GR-active EDCs (BPA, DCHP, endrin, and TF) used at **100 nmol/l significantly stimulated adipocyte lipid accumulation compared to the differentiation cocktail alone** (Figure 4), although they were not able to fully recapitulate the effects of dexamethasone (range: 61–70% increase over differentiation cocktail alone). |
|  |  |  |  | *In vitro,* 3T3-L1 murine cell line,  BPA (0.3 μM, 1 μM, 3 μM, 10 μM, 30 μM, 100 μM for NR activation and 5 μM, 10 μM, 20 μM for lipid accumulation)  6 d | Yes/ borderline (only at 100 μM; 1.5-fold activation) | No | Yes/ borderline (at 20 μM only; barely 150% of control) | (Taxvig et al., 2012) | Four chemicals (BPA, mono-ethylhexyl phthalate, butylparaben, PCB 153) out of the eleven revealed elevation in adipogenesis.  Nuclear receptor activation was assessed in transient transfection assay with mouse PPARα and mouse PPARγ (i.e., not human receptors). |
|  |  |  |  | *In vitro,* 3T3-L1 murine cell line,  BPA (10 μM)  0, 2, 4, 6 and 8 d |  |  | Yes | (Wada et al., 2007) | Approx. 3-fold lipid accumulation with 10 μM BPA (compared to vehicle) upon 5-6 d exposure |
|  |  |  |  | Review |  |  | Yes (more uncertain in human epidemiological studies) | (Heindel et al., 2022) | ER agonist. Potential effects also mediated via glucocorticoid receptor, or, via epigenetic effects (transient effects on PPARγ promoter methylation).  Rodent *in vivo* studies: Exposure to BPA from the fetus to the postnatal period permanently caused metabolic disruption and inhibit body weight gain regulation.  BPA exposure has been determined to disrupt the homeostatic systems of pregnant female mice required to maintain normal body weight. After the fetal-neonatal organogenesis period,  BPA exposure disrupted the homeostatic systems of pregnant female mice involved in maintaining normal body weight, including long-term consequences.  In general, exposure in utero/in early life seems to have greater impact upon BPA-induced obesity/adiposity.  In humans *in vivo*, 12 of 15 cross-sectional studies showed a significant association between BPA levels and obesity in adult population. Some of them indicate gender dimorphism. Two substantive cohort studies reported enhanced obesity odds in girls (aged 2-7) while no effects were observed in boys. However, the evidence from other (smaller) birth cohorts is contradictory (no association or decreased BMI in three studies).  Overall, BPA stimulated the differentiation of adipocytes *in vitro* and increased food intake, body weight, and adipose tissue *in vivo* in multiple rodent studies. BPA also disrupted rodents’ immune function, gastrointestinal tract microbiome, liver, pancreatic β cell function, and the hypothalamic regulation of food intake. Human epidemiological evidence, however, is inconsistent, with some studies indicating obesogenic effects.  The *in vivo* (esp. Zebrafish, rodent and human) observations might be difficult to reproduce *in vitro*, given the multifaceted effects, including on appetite regulation and insulin/glucose homeostasis. |
|  |  |  |  | Human primary mesenchymal stem cells (3 donors: 2 male, 1 female),  BPA (0.1-100 μM)  10 d |  | No (or weak) | Yes | (Norgren et al., 2022) | 8 bisphenols (BPA, BPAF, BPB, BPC, BPF, BPS, TBBPA, TCBPA) and their mixtures; mixtures at sub-active concentrations reached active levels due to additive effects.  A benchmark concentration model was developed (based on BPA) for quantitative comparison of bisphenols.  High throughput image-based assay, evaluating adipocyte number, size and lipid content (specimen stained with BODIPY493/503 and Hoechst 33342).  No increased PPARγ gene expression was observed on d 7 following exposure to BPA. (No bisphenol increased PPARγ expression statistically significantly, but mor potent isomers (BPC) and halogenated bisphenols (TBBPA, TCBPA) induced PPARγ gene expression ≥1.5-fold.) |
|  |  |  |  | Human epidemiology (cross-sectional, n=2747 adults) NHANES 2003-2006 |  |  | Yes, likely | (Carwile & Michels, 2011) | General and central obesity have been found to be associated with higher BPA exposure in the general adult population of the United States.  Because of the cross-sectional characteristic of this study, observed reverse causation is a remarkable concern. Therefore, longitudinal studies are required for further identification of this association. |
|  |  |  |  | Human epidemiology (n=80 matching samples of pregnant women) |  |  | Uncertain, potentially yes | (Veiga-Lopez et al., 2015) | Levels of unconjugated BPA and BPA glucuronide were determined.  Sampling at: first trimester, delivery, matching term cord blood.  Effects indicate some sex dimorphism: effects more pronounced in female offspring.  Higher levels of uBPA exposure during first trimester and term are related with sex specific birth weight decrease and gestational length increase, respectively. |
|  |  |  |  | Human epidemiology (cross-sectional, n=3967 adults aged > 20 years) NHANES 2003-2008 |  |  | Yes, likely | (Shankar, Teppala, & Sabanayagam, 2012) | Association of urinary bisphenol levels with obesity endpoints (BMI ≥ 30 kg/m2 and waist circumference ≥ 102 cm in men and ≥ 88 cm in women).  Increased of urinary BPA levels and obesity measures are positively correlated independent of confounding factors including, smoking, alcohol consumption, and serum cholesterol levels  Positive association between increasing levels of urinary BPA and both measures of obesity, independent of potential confounding factors including, smoking, alcohol consumption, and serum cholesterol levels; relation was steady across sex and race-ethnic groups (all p-trend < 0.05). This should be further confirmed in future prospective studies. |
|  |  |  |  | Human epidemiology (cross-sectional, n=2104 adults) NHANES 2003-2008 |  |  | N/A (focus on metabolis syndrome, which was associated with increased BPA urinary levels) | (Teppala, Madhavan, & Shankar, 2012) | Rising urinary BPA levels were associated positively with MetS, independent of confounders such as age, gender, race/ethnicity, smoking, alcohol intake, physical activity, and urinary creatinine. |
|  |  |  |  | Human epidemiology (cross-sectional, n=1030 adults), Korea 2010-2012 |  |  | Yes | (Ko et al., 2014) | The definition of obesity was stated according to the circumference of waist ≥ 90 and 85 cm for males and females, respectively.  Individuals with highest urinary BPA levels had significantly higher waist circumferences compared to those in the lowest quartile (p = 0.0071). |
|  |  |  |  | meta-analysis of human epidemiology (six NHANES cycles, 2003-2014, cross-sectional, n=4604 children, n=10989 adults) |  |  | No/ uncertain (dislipidaemia focus) | (Dunder et al., 2019) | Dyslipidaemia was associated with urinary BPA concentrations in children (≤17 years old) and adults (≥18 years old)  The meta-analysis did not disclose any significant associations between urinary BPA concentrations and LDL-C, HDL-C, TC, TG and ApoB in children. In adults, the meta-analysis revealed negative regression coefficients for all five lipid variables. However, no associations were significant following Bonferroni correction for multiple tests. |
|  |  |  |  | Human, *in vitro*, primary mature human adipocytes from 8 donors  and PANC-1 human pancreatic cells,  BPA (1 nM, 10 nM, 100 nM),  12,24 and 48 h  *According to gene modulation data 10nM BPA 24 h for treatment was chosen for subsequent experiments. | N/A | N/A | Possibly yes | (Menale et al., 2015) | Primary mature human adipocytes from prepubertal, non-obese children (sex not specified).  BPA increases the expression of pro-inflammatory cytokines and the expression of FABP4 and CD36, two genes involved in lipid metabolism.  Mean lipid area and triglyceride content was significantly increased at (1 nM) and 10 nM, not at 100 nM (highest test concentration). Estradiol (E2, 1 nM) as an ER agonist positive control did not significantly increase lipid area or triglyceride content.  In PANC-1 cells, BPA decreases active insulin secretion and the mRNA expression of PCSK1 gene which is involved in insulin production. |
|  |  |  |  | *In vitro,* 3T3-L1 murine cell line,  BPA (1 nM)  18 d | N/A | Yes | Yes | (Ariemma et al., 2016) | Evaluated endpoints: cell differentiation, proliferation, gene expression and metabolic function of adipocytes.  Significant increase in pre-adipocyte proliferation was observed with BPA treatment (p<0.01). PPARγ, FABP4/AP2 and C/EBPα expressions in BPA differentiated 3T3-L1 adipocytes were elevated by 3.5, 1.5 and 3-fold, respectively. Significant elevation in lipid accumulation (p<0.05) and insulin-stimulated glucose utilization reduction (p<0.001) were observed in mature adipocytes. Also, Leptin, IL6 and IFNγ mRNA levels were significantly increased in mature adipocytes (p<0.05). |
|  |  |  |  | Review |  | Uncertain in humans. (PPARγ activation seems not to be essential for BPA adipogenic effects in human cell culture) |  | (Cimmino et al., 2020) | Transcription factors such as PPARγ, C/EBP, Nrf2, HOX, and HAND2, are involved in the effect of BPA on fat and liver homeostasis, the cardiovascular system, and cancer. Further epigenetic effects contribute to BPA pathological effects.  Differences in effects depending on exposure window (pre- or postnatal exposure): sex-specific effects (with a trend for stronger effects in female offspring; not species dependent).  The evidence in human cells is equivocal.  BPA exposure at different concentrations significantly increases PPARγ expression in adult human preadipocytes and in freshly cultured omental adipose tissue from children donors. In contrast, PPARγ does not emerge as an essential mediator of BPA action in human adipose-derived stem cells. (Differences could be attributed to experimental protocol differences.) |
|  |  |  |  | *In vitro,* committed 3T3-L1 and uncommitted NIH3T3 murine preadipocytes,  BPA (1 nM)  8-day  exposure |  | Possibly/ uncertain (transient promoter demethylation) | Uncertain (**not in absence of induction cocktail**) | (Longo et al., 2020) | BPA effects are mediate by epigenetic effects: PPARγ promoter methylation reduced (transient, reversible).  Effects on PPARγ promoter methylation do not affect preadipocyte commitment and/or differentiation.  **In the absence of an induction cocktail, BPA did not induce/promote adipogenesis and did not commit NIH3T3 cells to adipocyte lineage**. |
|  |  |  |  | THP-1 human-monocyte-derived macrophages and HEK293T cells,  BPA and BPS (1 μM, 10 μM, 100 μM)  12 h | N/A | Yes | No (body weight in mice) | (Gao et al., 2020) | BPA and BPS; stock solution prepared at 100 mM in DMSO; solvent control accounted for highest solvent concentration in test chemicals (not exceeding 0.1% DMSO in culture medium).  Activation of PPARγ and significant induction of (PPARγ-dependent) genes FABP4, CD36 and NR1H3. Molecular effects were confirmed in mice *in vivo* (female C57BL/6).  100 μM BPA activated PPARγ (transient transfection of HEK293T and THP-1 with PPRE reporter plasmids; gene expression analysis for PPARγ). (100 μM BPS also significant activation, but less potent than BPA). Conversely, 100 μM BPA also induced expression of the downstream genes CD36 and FABP4.  In 4-week-old mice (6 per group), 10 week exposure to 50 or 5000 ug/kg/day (all ≤ NOAEL) via drinking water did not alter body weight (gain) and no effect on glucose tolerance was observed. |
|  |  |  |  | *In silico* (molecular docking to human PPARs and RXRs, BPA and 18 analogues) | Yes | Yes | N/A | (Sharma et al., 2018) | Maestro Schrodinger 9.4, docking scores of bisphenols were compared with the known endogenous and exogenous ligands of hPPARs and hRXRs.  BPA demonstrated good binding efficiency, some analogues higher binding efficiency than BPA (notably BPPH with a high tendency to be absorbed into tissues). BPPH showed the strongest binding with hPPARα, hPPARb, hPPARγ, and hRXRa whereas two of the most toxic bisphenols, BPM and BPAF demonstrated strongest binding with hRXRb and hRXRy.  D scores of BPA: -5.942 (hPPARα), -7.463 (hPPPARβ), -6.689 (hPPARγ) (overall: “promising binding efficiencies with hPPARs”; lower value indicates better docking. Model ligands for PPARα include fibrates and DHA, for PPARγ glitazones). |
|  |  |  |  | Review for regulatory purposes |  |  | Uncertain in human epidemiological studies | (EFSA Panel on Food Contact Materials & Aids, 2015) | Inconsistent with the results of cross-sectional studies one prospective study found that a higher BPA concentration in maternal urine during pregnancy was associated with a lower level of obesity in daughters. A causal link between BPA exposure and metabolic effects in humans cannot be established.  A number of studies in pre- and postnatally exposed rats and mice indicate that BPA exposure could have an effect on metabolic function as evidenced by effects on glucose or insulin regulation or lipogenesis, and body weight gain (short-term studies). However, based on the results from other studies with a longer duration (e.g., 90 days) there is no convincing evidence that BPA is obesogenic after intrauterine exposure or in longer-term studies. |
|  |  |  |  | Review for regulatory purposes; response to draft EFSA Panel on Food Contact Materials, 2015 |  |  | Uncertain. BPA can have effects on metabolism (adipogenesis/ obesity not specified) | (ANSES, 2014) | The report highlights controversies in the conclusions made by EFSA. In contrast to EFSA, who focused on exposure of the general population/via food, ANSES extends the considered population to cover also occupational exposure, e.g., of cashiers handling thermal paper receipts.  Specifically, ANSES considers the available experimental evidence “sufficient to consider that BPA can have effects on metabolism”, and the most reasonable metabolism endpoint/process affected is glucose and insulin regulation and/or pancreatic morphology and function. |
|  |  |  |  | Human, epidemiology/ environmental exposure (adult males; n=76) |  |  | yes, likely (positive correlation of plasma BPA and visceral adiposity) | (Savastano et al., 2015) | Visceral adiposity (24 out of 76 subjects) correlated with higher plasma levels of BPA, triglycerides (elevated), and pro-inflammatory cytokines (esp. IL-6). |
|  |  |  |  | Human, epidemiology (representative Canadian household population; n=4733) |  |  | Yes | (Do et al., 2017) | The authors conclude that urinary bisphenols are associated with BMI-defined obesity (p-trend: 0.036 or 0.041 in two different models. Model 1: adjusted for sex, age, urinary creatinine; model 2: model 1 covariates plus race/ethnicity, alcohol consumption, junk food consumption). |
|  |  |  |  | Mouse, *in vivo* (male 6-week-old CD1 mice & C57BL/6J mice, n=6/group; 5 groups, 28 d exposure via diet to 0.05-50 ppm BPA) |  |  | No effect on body weight gain  Potentially increased perigonadal WAT (only at one dose) | (Marmugi et al., 2012) | Focus on effects in CD1 mice; confirmation study in C57BL/6J mice confirmed trend in effects/observations, but with smaller magnitude. Focus of the study on hepatic effects.  **No effect on body weight gain** or liver weight, plasma glucose, od cholesterol (LDL-C, HDL-C); plasma insulin was significantly increased at 5-500 µg/kg bw/d with reverse dose-response pattern (highest increase at lowest dose), significant increase in plasma triglycerides upon exposure to 500 µg/kg bw/d. Significant increase in perigonadal White Adipose Tissue weight upon exposure to 50 µg/kg bw/d only.  **Low doses** influence de novo hepatic lipogenesis in male CD1 mice, thereby contributing to hepatic steatosis.  Oral exposure corresponds to 0 (controls), 5, 50 (TDI), 500, or 5,000 µg BPA/kg bw/d (NOAEL), assuming diet consumption of 10% bw/d. |
|  |  |  |  | Mouse, *in vivo* (pregnant CD1 female mice, exposed daily orally via micro-pipettor from gestational day 9-18 (during period of differentiation of preadipocytes) to 5-50,000 µg/kg bw/d BPA; effects observed in male offspring only) |  |  | Yes (except highest dose)  male-specific effect | (Angle et al., 2013) | Litter size per BPA treatment group [µg/kg bw/d BPA]: 14 (negative control), 9 (BPA-5), 12 (BPA-50), 12 (BPA-500), 11 (BPA-5,000), 14 (BPA-50,000), 9 (DES-0.1/positive control). Study was concluded when males were 5 months old.  **Prenatal exposure to BPA levels at or below the current [2013] predicted NOAEL of 5000 µg/kg bw/d** resulted in significant increase in postnatal body weight gain, adipocyte number and volume and the overall amount of abdominal fat, altered food intake, serum insulin, adiponectin and leptin levels, and impaired glucose tolerance and insulin sensitivity in **male offspring**. These effects were not statistically significant at the highest dose [50,000 µg/kg bw/d]. |
|  |  |  |  | Human and mouse *in vitro,*  MSCs, and 3T3-L1 murine preadipocytes,  BPA (1nM, 10nM, 100 nM , 1 µM)  14 d |  | no activity | Uncertain (not in MSCs, induction in 3T3-L1 only) | (Chamorro-García et al., 2012) | BPA failed to promote adipogenesis in MSCs with all tested concentrations, but induced adipogenesis in 3T3-L1 cells at 10 Nm.  BPA neither activated nor antagonized RXR or PPARγ in transient transfection assays. |
|  |  |  |  | Human *in vitro,* reporter cell line  HGELN-GAL- PPARγ  BPA (10^-9^M, 10^-8^ M, 10^-7^ M, 10^-6^ M, 10^-5^ M)  16 h |  | No | N/A | (Riu et al., 2011) | The following stably transfected reporter cell lines were utilised: HGELN, HGELN­ERα, HGELN­ERβ, and HGELN­GAL-PPARγ. For PPARγ, 100 Nm ROSI were used as positive control/reference, for ERα/β 10 nM E2 were used as reference.  Increasing degree of BPA halogenation gradually increased the potency and magnitude of PPARγ activation, with simultaneously decreasing affinity to ERα/β. |
|  |  |  |  | Human, *in vivo* (analytical method development and application, n=21 serum samples) |  |  | N/A | (Dirtu et al., 2008) | BPA (0.71 ng mL−1) and TCS (0.52 ng mL−1) median concentrations in Belgian human serum samples were in line with data for human fluids which is previously reported. Slightly increased levels of TBBPA (0.08 ng mL−1) were determined in Belgium serum samples compared to Norwegian. |
|  |  |  | BPA, BPS | Human *in vitro,* Subcutaneous primary preadipocytes, n=5 donors,  (25 μM)  2-4 d | Unlikely for BPA;  Yes, potentially (for BPS) | Yes, potentially/ partially (more pronounced for BPS than for BPA) | N/A (but inferred from previous studies) | (Boucher et al., 2016) | 1 μM dexamathasone as positive control  RNA sequencing, followed by ingenuity pathway analysis (IPA). All genes with a false discovery rate (FDR) p < 0.05 and fold-change > +/- 1.5-fold up- or down-regulated compared to matched controls were considered significantly differentially expressed.  BPA: most perturbed pathways on day 2 included hepatic fibrosis, LXR/RXR activation and hepatic cholestasis. On day 4: LXR/RXRactivation and atherosclerosis signalling.  BPS: The top pathways, by statistical significance, on day 2 AMPK signalling, adipogenesis and cholesterol biosynthesis. On day 4: LXR/ RXR activation, AMPK signalling and PPARα/RXRα activation.  LXR has been shown to increase adipocyte differentiation through PPARγ up-regulation. |
|  |  |  |  | *In vitro,* human and polar bear reporter cell lines (0.5 pM – 25 μM)  24 h | No (5% relative response at 25 μM on hPPARα) | N/A | N/A | (Routti et al., 2019) | The authors transiently co-transfected COS7 cells with pCMX-GAL4-pbPPARα or pCMX-GAL4-hPPARα, tk(MH100)x4-luciferase, and pCMV-β-galactosidase. Activities of environmental contaminants were established against the known PPARα agonist WY-14643 and antagonist MK-886.  Polar bear PPARα (pbPPARα) was both quantitatively and qualitatively more susceptible than human PPARα (hPPARα) to transactivation by less lipophilic compounds. |
|  |  |  |  | *In vitro,* 3T3-L1 murine cell line  (10 μM, 50 μM, 80 μM)  8 d | N/A | N/A | Bordeline/ weak (10 μM BPA not active, 50 and 80 μM BPA induction ~1.5-2-fold)) | (Bastos Sales et al., 2013) | positive control: 1 μM troglitazone  Dose-dependent increase in adipocyte differentiation was shown with BPA treatment.  80 μM BPA led to a significant increase in global DNA methylation in 3T3-L1 cells. Modest global hypomethylation was observed in the murine cell line, but not in the human cell line (SK-N-AS cells). |
|  |  |  |  | *In vitro,* 3T3-L1 murine cell line  (0.1-50 μM)  10 d | N/A | Yes, weak/ borderline (hPPARγ CALUX assay; LOEC = 1 μM) | Yes, weak (LOEC: with insulin = 25 μM, without insulin = 12.5 μM) | (Pereira-Fernandes et al., 2013) | This is the first description and successful demonstration of Nile Red fluorescence for the quantification of lipid accumulation in 3T3-L1 cells, including the statistical derivation of a statistically robust fold-change classification threshold for non- weak and strong obesogenic chemicals.  Two exposure scenarios were tested: exposure with or without insulin in exposure medium. Positive control: 2 d MDI hormone cocktail induction followed by 8 d insulin-only stimulation.  The assay and classification threshold were derived using TBT and ROSI.  Quality criteria for test acceptance:   - MDI-positive control: degree of lipid accumulation (DLA) > 10 (> 4 for insulin co-exposure scenario) - Lipid accumulation threshold (LAT; equivalent to analytical chemistry LoQ): blank value [solvent control] + 10x[SD solvent control over all experiments] (LAT is based on variation observed in solvent control) - For single chemical treatment: LAT = 1.76 - For insulin co-exposure: LAT = 2.17   **Chemicals inducing a statistically significant DLA in at least 2 subsequent concentrations compared to the solvent control, but lower than the LAT are considered weak obesogens, whereas chemicals inducing a significant DLA higher than the LAT are considered strong obesogens**.  For the “strictly standardised mean difference (SSMD) method”, obesogens inducing a SSMD value higher than 4.7 are considered strong obesogens, whereas chemicals inducing SSMD value between 2 and 4.7 are selected as weak obesogens (SSMD < 2 = non-obesogen).  DLA max. At 50 μM: 1.38 without insulin, 1.85 with insulin.  It is noted that insulin can moderate the degree of adipogenesis/lipid accumiulation observed. |
|  |  |  |  | Human, *in vivo* (epidemiology/ biomonitoring, human adipose fat samples, n=20) | N/A | N/A | N/A | (Wang, Asimakopoulos, & Kannan, 2015) | Human adipose fat samples (N= 20) collected from New York City, USA, were analysed for the presence of environmental phenols, including BPA, benzophenone-3 (BP-3), TCS, and parabens, as well as heterocyclic aromatic compounds, including benzotriazole (BTR), benzothiazole (BTH), and their derivatives.  BPA and TCS were frequently detected in adipose tissues at concentrations (geometric mean [GM]: 3.95 ng/g wet wt for BPA and 7.21 ng/g wet wt for TCS) similar to or below the values reported for human urine. |
|  |  |  |  | Human or mouse *in vitro,*  3T3-L1 murine preadipocytes or OP9 mBMSCs  10 d  Primary human subcutaneous preadipocytes  14 d |  | No | Uncertain | (Kim et al., 2021) | Endpoints: lipid accumulation, RNA-Seq and confirmation of gene expression by RT-qPCR  Data-driven hierarchical clustering approach to identify PPARγ modulating chemicals, and transcriptional changes related to differentiation into white or brite/beige adipose tissue.  The authors compared a strong PPARγ therapeutic agonist that also was shown to modify PPARγ phosphorylation (i.e., ROSI, a chemical that was shown to modify only PPARγ phosphorylation (i.e., roscovitine), a weak PPARγ agonist and endogenous molecule (i.e., 15dPGJ2), and two known environmental PPARγ ligands [i.e., TBBPA and TPhP].  Important genes for predicting PPARγ ligand/modification status, specifically the down-regulation of *Rpl13* and the upregulation of *Cidec*.  In 3T3-L1: a similar lipid accumulation was observd with ROSI in the absence of dexamethasone, with dexamethasone no significant accumulation (~vehicle-level), in OP9 on vehicle-level.  Max. Tested concentration: 1x10-5 M  Max. Non-toxic concentration: 1x10-5 M |
|  |  |  |  | Human, *in vitro,*  hMSCs  BPA (for cytotoxicity: 1 µM, 3 µM, 10 µM, 30 µM, 100 µM, for gene expression: 3 µM, 30 µM, 100 µM)  72 h |  |  |  | (Burkhardt et al., 2024) | Positive control: ROSI (for cytotoxicity: 0.03 µM, 0.1 µM, 0.3 µM, 1 µM, 3 µM; for gene expression: 1 µM, 10 µM, 30 µM )  No effect on either overall glucose consumption, production of adipokines adiponectin and leptin, or lipid storage, size and total lipid content in the cell culture. Lipidomic analysis of cell culture medium revealed significant changes in the extracellular lipidome even no effects on any other studied endpoint observed.  BPA at concentration of 100 μM showed a trend to decrease GLUT4, ADIPOQ and LEP, albeit not statistically significant. |
| **3,3’,5,5’ Tetrabromobisphenol A (TBBPA)** | 79-94-7 | 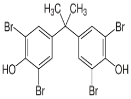 | Flame retardant | **SUMMARY** | No | Yes | Yes – moderate |  | Structurally similar to BPA but different activity in PPARs  Active metabolite: sulphate (but as TBBPA sulphate is not stable over time/difficult to store, guidance regarding specific storage and use of fresh batches needed in relevant SOP’s.) Likely to induce lipid accumulation in (pre-)adipocytes, via PPARγ activation. |
|  |  |  |  | Human, *in vivo* (analytical method development and application, n=21 serum samples) |  |  | N/A | (Dirtu et al., 2008) | BPA (0.71 ng mL−1) and TCS (0.52 ng mL−1) median concentrations in Belgian human serum samples were in line with data for human fluids which is previously reported. Slightly increased levels of TBBPA (0.08 ng mL−1) were determined in Belgium serum samples compared to Norwegian. |
|  |  |  |  | Human, *in vivo* (epidemiology, n=38 mother-infant pairs) |  |  | N/A | (Kim & Oh, 2014) | Concentration of TBBPA and hexabromocyclododecanes (HHBCDs) in serum was correlated with thyroid hormone levels. 26 infants with congenital hypothyroidism, 12 health infants, and their mothers were included.  Significant correlations were found between mothers and infants in TBBPA and HBCD concentrations, confirming high maternal transfer rate. Concentrations in infants rapidly decreased within 2-3 months after birth.  Analyte concentrations were not significantly different in the two infant groups. TBBPA correlated weakly with thyroid hormones, showing a positive relationship with FT4 but a negative relationship with T3. |
|  |  |  |  | *In vitro,* reporter cell lines 3T3-L1 murine cell line,  (10 μM)  8 d |  | Yes (max. Effect at ~3x10^-6^ M, LOEC: 10^-6^ M) | Yes, via PPARγ activation | (Riu et al., 2011) | Human, zebrafish, and *Xenopus* PPARγ reporter cell lines were constructed for transient transfection in HeLa cells. Additionally, the following stably transfected reporter celllines were utilised: HGELN, HGELN­ERα, HGELN­ERβ, and HGELN­GAL-PPARγ. For PPARγ, 100 nM ROSI were used as positive control/reference. TBBPA was cytotoxic at concentrations >10 μM.  BPA: no effect on PPARγ activation. Increasing degree of BPA halogenation gradually increased the potency and magnitude of PPARγ activation, with simultaneously decreasing affinity to Ers.  Species comparison revealed a similar PPARγ activation (3-4 fold activation vs. Vehicle control) for human, zebrafish, and *Xenopus* PPARγ with 10 μM TBBPA, however the affinities differed for the other three chemicals tested (10 μM TCBPA, 10 μM MEHP, 1 μM ROSI)  10 μM TBBPA induced lipid accumulation in 3T3-L1 murine preadipocytes as 1 μM ROSI (positive control), which was supressed by CD5477 (PPARγ antagonist). Lipid accumulation was less pronounced with 10 μM TCBPA.  Crystallography: 2.55 Å and 2.70 Å resolution for TBBPA­PPARγ and TCBPA­PPARγ complexes were acquired. |
|  |  |  |  | Zebrafish larvae (28 hpf wildtype AB/Tuebingen and transgenic *Tg(hPPARy-eGFP)*, 3x10^-10^ M – 10^-6^ M, 24 h) and reporter cell lines |  | Yes (LOEC hPPARγ and zPPARγ ~3x10^-7^ M) | Yes (LOEC 10^-7^ M) | (Riu et al., 2014) | LT-hPPARγ transgenic zebrafish (*Tg(hPPARy-eGFP)*): eGFP is expressed in the presence of active hPPARγ ligands (no cross-activation by RXR ligands).  28 hpf TG zebrafish embryos exposed to chemicals for 24 h for eGFP signal detection.  For late-onset weight gain, ZF were exposed to chemicals from 3-11 dpf (10 nM – 1 μM TBBPA) and assessed at 11 dpf (ORO staining) or 30 dpf (BMI measurement, 100 nM TBBPA only).  Both halogenated-BPAs, as well as TBBPA-sulphate induced lipid accumulation in zebrafish larvae, as well as late-onset weight gain in juvenile zebrafish. |
|  |  |  |  | Human and polar bear reporter cell lines, *in vitro,*  (0.5 pM – 25 μM)  24 h | No | N/A | N/A | (Routti et al., 2019) | The authors transiently co-transfected COS7 cells with pCMX-GAL4-pbPPARα or pCMX-GAL4-hPPARα, tk(MH100)x4-luciferase, and pCMV-β-galactosidase. Activities of environmental contaminants were established against the known PPARα agonist WY-14643 and antagonist MK-886.  Polar bear PPARα (pbPPARα ) was both quantitatively and qualitatively more susceptible than human PPARα (hPPARα ) to transactivation by less lipophilic chemicals. |
|  |  |  |  | Human *in vitro,* HG5LN reporter cell lines for PPARγ (10^-8^ – 5x10^-5^ M)  24 h | N/A | Yes (EC20: 0.1 μM, EC50: 0.2 μM, 40% max activity) | N/A | (Garoche et al., 2021) | human, mouse, zebrafish, and *Xenopus* PPARγ were tested using known hPPARγ ligands, and environmental chemicals.  HPPARγ and mPPARγ showed similar activities, with marked differences in xPPARγ (less pronounced) and zfPPARγ (more pronounced).  Only data from hPPARγ were extracted here (activity parameters for other receptors/ species is given in the publication). |
|  |  |  |  | Human or mouse *in vitro,*  3T3-L1 murine preadipocytes or OP9 mBMSCs  10 d  Primary human subcutaneous preadipocytes  14 d |  | Yes | Uncertain. No in 3T3-L1 (with and without dexamethasone), yes in OP9 | (Kim et al., 2021) | Endpoints: Lipid accumulation, RNA-Seq and confirmation of gene expression by RT-qPCR.  Data-driven hierarchical clustering approach to identify PPARγ modulating chemicals, and transcriptional changes related to differentiation into white or brite/beige adipose tissue.  The authors compared a strong PPARγ therapeutic agonist that also was shown to modify PPARγ phosphorylation (i.e., ROSI), a chemical that was shown to modify only PPARγ phosphorylation (i.e., roscovitine), a weak PPARγ agonist and endogenous molecule (i.e., 15dPGJ2), and two known environmental PPARγ ligands [i.e., TBBPA and TPhP].  Important genes for predicting PPARγ ligand/modification status, specifically the down-regulation of *Rpl13* and the upregulation of *Cidec*.  Max. Tested concentration: 2x10^-5^ M  Max. Non-toxic concentration: 2x10^-5^ M |
| **Tributyltin (TBT)** chloride | 1461-22-9 | 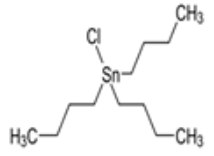 | Fungicide | **SUMMARY** | No | Yes (partial agonist) | Yes, strong (positive control) |  | All Rexinoids inhibit GAL4 luciferase in reporter gene assays, Therefore while rexinoids can be used in hMSC adipogenesis assay, they are not suitable for use in luciferase-based reporter gene analyses.  Activity mainly mediated via RXR binding.  Strong model inducer of adipogenesis/ lipid accumulation. Positive control chemical: lipid accumulation in pM-nM range. Highly cytotoxic. |
|  |  |  |  | *In vitro*, murine mesenchymal stem cell line C3H/10T1/2 and embryonic stem cells (CGR8)  TBT (1 nM and 100 nM)  Undifferentiated growth (0-6 days)  Induction (6-8 days)  Terminal differentiation (8-12 days) |  |  |  | (Biemann et al., 2012) | The final amount of differentiated adipocytes, cellular triglyceride content and mRNA expression of adipogenic marker genes (adiponectin, FABP4, PPARγ2, LPL) were measured and compared with corresponding unexposed cells. DEHP (100 μM) increased adipogenesis during the hormonal induction period.  Exposure of undifferentiated murine embryonic stem cells was demonstrated no effect on subsequent adipogenic differentiation of the investigated EDC. |
|  |  |  |  | Mouse *in vitro*, MSCs  TBT (5, 50, 100 nM)  14 d |  |  |  | (Chang et al., 2023) | ROSI (100 nM) and the selective RXR activator LG268 (100 nM) were used as positive controls for PPARγ or RXR activation, respectively.  miR-21, miR-33, and miR-223 were expressed consistent with an ability to differentially regulate target genes during adipogenesis. 24-hour exposure to 50nM TBT increased miR-223 levels, decreased expression of its target genes ZEB1, NFIB, and FOXP1 in MSCs.  ROSI and TBT increased miR-223 levels. This induction was inhibited by the PPARγ antagonist T0070907 but not by the RXR antagonists HX531 or UVI3003, placing miR-223 downstream of PPARγ. Chromatin immunoprecipitation confirmed TBT-induced binding of PPARγ to regulatory elements in the miR-223 promoter. miR-223 levels were elevated in white adipose tissue of F2 and F3 male descendants of pregnant F0 mouse dams exposed to  50nM TBT throughout gestation. miR-223 levels were potentiated in males fed an increased fat diet.  TBT induced miR-223 expression and increased adipogenesis in MSCs through the PPARγ pathway and that transgenerationally increased expression of miR-223 plays a crucial role in the obesity development caused by TBT exposure. |
|  |  |  |  | Zebrafish (wildtype AB/Tuebingen, 0.1 & 1 nM) |  | N/A | Yes | (Riu et al., 2014) | For late-onset weight gain, ZF were exposed to chemicals from 3-11 dpf (0.1 & 1 nM TBT) and assessed at 11 dpf (ORO staining, ~3-fold increase compared to vehicle control) and 30 dpf (BMI; 1 nM TBT only. Significantly increased BMI (~1.5-fold to 10 mg/cm^2^. P<0.001). |
|  |  |  |  | *In vitro*, 3T3-L1 murine preadipocytes,  10 nM, 50 nM  8 d | N/A | N/A | Yes (~2-fold induction, 10 nM TBT; ~2.5-fold with 50 nM TBT) | (Bastos Sales et al., 2013) | Positive control: 1 μM troglitazone  TBT induces both global DNA demethylation and adipocyte differentiation *in vitro*. |
|  |  |  |  | Rat, *in vivo* (adult female Wistar rats, 12 weeks old, 0.1 ug/kg/d oral, 15 d. N=10/ group)  Mouse *in vitro,* (differentiated 3T3-L1-derived murine adipocytes, 24 h, 10 or 100 nM) | N/A | Yes (incresed protein levels in WAT in female rats after 15 d oral exposure)) | Yes | (Bertuloso et al., 2015) | Increase in the body weight of the rats on the 15th day of oral exposure and associated with high parametrial (PR) and retroperitoneal (RP) WAT weights.  Increased the adiposity, inflammation and expression of ERα and PPARγ proteins in both RP and PR WAT. In 3T3-L1 cells, estrogen treatment reduced lipid droplets accumulation, however increased the ERα protein expression. In contrast, TBT-treatment increased the lipid accumulation and reduced the ERα expression.  Conclusion: TBT leads to adiposity in WAT specifically, impairing the metabolic functions of the liver and pancreas. |
|  |  |  |  | Rat, *in vivo* (adult female Wistar rats, 12 weeks old, 0.1 ug/kg/d oral, 15 d. N=10/ group) | N/A | Yes (protein expression) | Yes | (Ceotto Freitas-Lima et al., 2018) | WAT morphophysiology and adipokine profiles evaluated.  TBT increased body weight and adiposity, and induced dyslipidaemia, insulin resistance, and inflammatory infiltration of WAT. Adipokine levels were elevated (leptin (serum), TNFα, IL-6, IL-4); IL-13 and serum adiponectin were decreased.  While adipocyte diameter was increased by TBT, adipocyte number decreased. |
|  |  |  |  | Review | N/A | Yes | Yes | (Grün & Blumberg, 2006) | Organotin/ environmental obesogen exposure is of sepcial concern in combination with the typical high-calory/ high-fat Western diet (and other obesogenic lifestyle choices): then, predisposition of cells to adipocytes/WAT can lead to aggravated obesity. |
|  |  |  |  | Mouse *in vivo* (male C57BL/6 mice, 6 week old, 24 h, 0.3 mg/kg bw, i.p., or: Pregnant C57BL/6 mice, 0.05 & 0.5 mg/kg bw/d, i.p., GD 12–18 with TBT)  *In vitro*, 3T3-L1 murine preadipocytes, 10 & 100 nM,  7 d  Amphibian (*Xenopus laevis* tadpoles) *in vivo*, 1-10 nM, stage 48-64(66)) | No (mPPARα) | Yes (LOEC hPPARγ ~10 nM; K_d_ = 20 nM) | Yes (increased adiposity, but not necessarily bodyweight) | (Grün et al., 2006) | TBT is a nanomolar affinity ligand for RXRs and PPARγ.  In utero exposure induce increased lipid accumulation in adipose depots, liver, and testis of neonate mice and outcomes with epididymal adipose mass elevation in adults.  In *Xenopus*: formation of ectopic adipocytes in and around gonadal tissues after TBT, RXR- or PPARγ-ligand exposure.  Affinity to nuclear receptors has been measured by reporter gene assay in transiently transfected COS7 cells.  In male mice *in vivo*, a pronounced decrease was observed for RXRa/PPARγ, C/EBPα, and C/EBPδ in adipose tissue and testis. In contrast, TBT, AGN195203, and troglitazone significantly induced expression of the early adipogenic transcription factor C/EBPβ in liver and testis, whereas it was more weakly induced in adipose tissue.  For in utero exposure, offspring was killed at birth for histological examination, or followed-up (cross-fostered by unexposed dams) until 10 weeks old. TBT exposure resulted in disorganisation of hepatic and gonadal architecture and generally increased Oil Red O staining at birth. Adiposity was increased in liver, testis, inguinal adipose and mammary adipose tissue following in utero (GD 12-18) exposure to 0.5 mg TBT/kg bw/d. Postnatal observations revealed, that thile ependymial adipose tissue weight was significantly increased, there was no significant gain in bodyweight.  In *Xenopus*, dose-dependent increase in ectopic adipocyte formation posterior to the fat bodies in and around the gonads of both sexes after TBT or RXR/PPARγ ligand exposure (not upon estradiol treatment). 10 nM TBT ectopic adipocytes were observed in approximately 45–60% of animals; in males, testicular tissue was interspersed with, or replaced by, adipocytes along the anterior-posterior axis. |
|  |  |  |  | *In vitro*, 3T3-L1 murine preadipocytes,  0.1-50 nM  10 d | N/A | Yes, strong (hPPARγ CALUX assay; LOEC = 3 nM) | Yes, strong/ moderate (LOEC with/ without insulin = 6.25 nM) | (Pereira-Fernandes et al., 2013) | This is the first description and successful demonstration of Nile Red fluorescence for the quantification of lipid accumulation in 3T3-L1 cells, including the statistical derivation of a statistically robust fold-change classification threshold for non- weak and strong obesogenic chemicals.  Two exposure scenarios were tested: exposure with or without insulin in exposure medium. Positive control: 2 d MDI hormone cocktail induction followed by 8 d insulin-only stimulation.  The assay and classification threshold were derived using TBT and ROSI.  Quality criteria for test acceptance:   - MDI-positive control: degree of lipid accumulation (DLA) > 10 (> 4 for insulin co-exposure scenario) - Lipid accumulation threshold (LAT; equivalent to analytical chemistry LoQ): blank value [solvent control] + 10 x [SD solvent control over all experiments] (LAT is based on variation observed in solvent control) - For single chemical treatment: LAT = 1.76 - For insulin co-exposure: LAT = 2.17   **Chemicals inducing a statistically significant DLA in at least 2 subsequent concentrations compared to the solvent control, but lower than the LAT are considered weak obesogens, whereas chemicals inducing a significant DLA higher than the LAT are considered strong obesogens**.  For the “strictly standardised mean difference (SSMD) method”, obesogens inducing a SSMD value higher than 4.7 are considered strong obesogens, whereas chemicals inducing SSMD value between 2 and 4.7 are selected as weak obesogens (SSMD < 2 = non-obesogen).  The reference chemicals TBT and ROSI induced 3T3-L1 differentiation dose-dependently, with or without insulin; ROSI to a greater extent/magnitude than TBT.  Adipogenic potential was shown for all tested parabens, several musks and phthalate compounds and BPA. PPARγ activation was related to adipogenesis for parabens, phthalates and BPA, however not required for Tonalide induced obesogenic effects.  DLA max at 50 nM: 3.55 without insulin, 2.78 with insulin |
|  |  |  |  | *In vitro,* 3T3-L1 murine cell line,  50 nM  10 d | N/A | (inferred yes) | Yes, strong (reference obesogen) | (Pereira-Fernandes et al., 2014) | Study expanding on (Pereira-Fernandes et al., 2013); exposure scenario without insulin.  Gradual distinction corresponding to differences in induction of lipid accumulation could be made between test and reference obesogens based on transcription patterns. Marker genes that are able to distinguish between non, test,and reference obesogens were identified, including: *Fabp4/AaP2, Cd36 (clustered with Arxes2, Reep6); Plin2, Mrap, Pck1; Nr1h3 (*LXR*), Rxrg.* |
|  |  |  |  | Human, *in vitro,* hMSCs  TBT (for cytotoxicity: 0.3 µM, 1 µM, 3 µM, 10 µM, 30 µM; for gene expression: 1 µM, 10 µM, 30 µM)  72 h |  |  |  | (Burkhardt et al., 2024) | Positive control: ROSI (for cytotoxicity: 0.03 µM, 0.1 µM, 0.3 µM, 1 µM, 3 µM; for gene expression: 1 µM, 10 µM, 30 µM )  No effect on either overall glucose consumption, production of adipokines adiponectin and leptin, or lipid storage, size and total lipid content in the cell culture. Lipidomic analysis of cell culture medium revealed significant changes in the extracellular lipidome even no effects on any other studied endpoint observed. |
| **Perfluorooctanoic acid (PFOA)** | 335-67-1 | 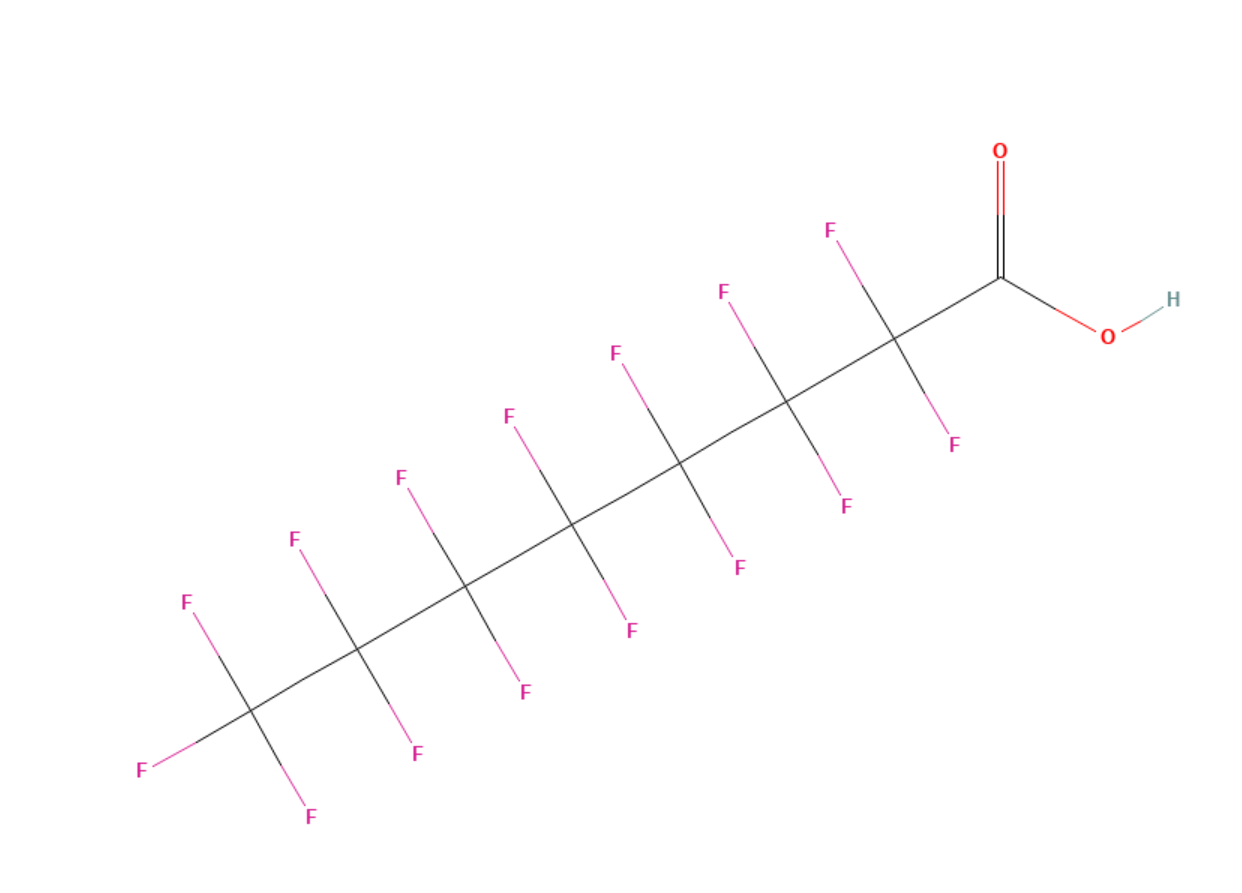 | Industrial chemical, non-stick coating, cosmetic ingredient | **SUMMARY** | Yes, strong agonist | Yes, moderate | Uncertain (possibly weak positive as suggested by Filer et al 2022) |  | PFOA-family chemicals with 8-9 carbon backbone have greater activity than with 7 and 10 carbons. PFAS: strong association of grandmaternal exposure with obesity in granddaughters.  Very varied applications, including in cosmetics, garments, cooking utensils, chrome plating, and others.  Both, negative and positive for lipid accumulation in murine 3T3-L1 pre-adipocytes and mixed evidence from human models (*in vitro* and epidemiology).  Human *in vivo*: some indication for association with altered lipid profile (esp. triglycerides), but no clear correlation with obesity, fat mass, or body weight.  Listed under Stockholm POPs Convention. |
|  |  |  |  | Human *in vitro,* reporter cell line (HGELN­GAL-PPARγ, 16 h, 10^-7^ – 10^-4^ M) |  | Yes (LOEC: 10^-5^ M) | N/A | (Riu et al., 2011) | For PPARγ, 100 nM ROSI were used as positive control/reference.  PFOS resulted at a higher relative activity at the max. Testing concentration (10^-4^ M): 80% PFOS vs. ~50% PFOA. LOEC was comparable. |
|  |  |  |  | Human and polar bear reporter cell lines, *in vitro,*  (0.5 pM – 25 μM)  24 h | Yes (relative activity of 25 μM: 91% in pbPPARα, 33% in hPPARα) | N/A | N/A | (Routti et al., 2019) | The authors transiently co-transfected COS7 cells with pCMX-GAL4-pbPPARα or pCMX-GAL4-hPPARα, tk(MH100)x4-luciferase, and pCMV-β-galactosidase. Activities of environmental contaminants were established against the known PPARα agonist WY-14643 and antagonist MK-886.  Polar bear PPARα (pbPPARα) was both quantitatively and qualitatively more susceptible than human PPARα (hPPARα) to transactivation by less lipophilic chemicals.  Relative activity of 25 μM PFOS: 4% in pbPPARα, 2% in hPPARα. |
|  |  |  |  | *In vitro,* 3T3-L1 murine cell line,  (10 μM)  8 d | N/A | N/A | Boderline/weak (~1.3-fold induction (stat. Significant) with 10 μM PFOA) | (Bastos Sales et al., 2013) | positive control: 1 μM troglitazone  10 μM PFOS: no induction  No effects on 3T3-L1 differentiation with PFOA treatment. |
|  |  |  |  | Human *in vitro,* HG5LN reporter cell lines for PPARγ (10^-6^ – 10^-4^ M)  24 h | N/A | Yes (EC20: 17 μM, EC50: 91 μM, 51% max activity) | N/A | (Garoche et al., 2021) | Human, mouse, zebrafish, and *Xenopus* PPARγ were tested using known hPPARγ ligands, and environmental chemicals.  HPPARγ and mPPARγ showed similar activities, with marked differences in xPPARγ (less pronounced) and zfPPARγ (more pronounced).  Only data fro hPPARγ were extracted here (activity parameters for other receptors/ species is given in the publication).  PFOS was less potent (max. Activity 36 %, EC20 21 μM). |
|  |  |  |  | Human *in vivo* epidemiology (n = 308) | N/A | N/A | N/A (elevated serum triglycerides, total and LDL cholesterol) | (Koshy et al., 2017) | World Trade Center Health Registry (PFAS-exposed, n=123) and (non-exposed) matched control group (n=185). Exposure to PFASs (serum levels) and association with cardiometabolic profile (arterial wall stiffness, BMI, insulin resistance, fasting total cholesterol, HDL, LDL and triglycerides) was assessed.  Significant, positive association of PFOA with (serum) triglycerides (beta coefficient = 0.14, 95% CI: 0.02, 0.27, 15.1 percent change), total cholesterol (beta coefficient = 0.09, 95% CI: 0.04, 0.14, 9.2 percent change), and LDL cholesterol (beta coefficient = 0.11, 95% CI: 0.03, 0.19, 11.5 percent change). |
|  |  |  |  | Human *in vivo* epidemiology (n = 222 pregnant women) | N/A | N/A | N/A (strong linear trend for elevated triglycerides) | (Spratlen et al., 2020) | World Trade Center birth cohort; association between 5 PFAS (PFOA, PFOA, PFHxS, PFNA, PFDS) in cord blood and cordblood lipids (total lipids, total cholesterol, triglycerides) was studied. |
|  |  |  |  | 3T3-L1 mouse preadipocytes, 5-100 μM,  16 d |  |  | Uncertain | (Watkins et al., 2015) | Slight increase in cell number (<2-fold), increased DNA content (~3-fold), slight decrease in cell size (~25%), decreased lipid area per cell (~30% decrease), increased total triglyceride (~4-fold). |
|  |  |  |  | *In vitro,* 3T3-L1 murine cell line,  PFOA (0.3 μM, 1 μM, 3 μM, 10 μM, 30 μM, 100 μM for NR activation and 20 μM, 50 μM, 75 μM for lipid accumulation)  6 d | Yes (LOEC 30 μM) | No/ borderline (non-significant 1.5-fold activation with 100 μM) | No | (Taxvig et al., 2012) | Four chemicals (BPA, mono-ethylhexyl phthalate, butylparaben, PCB 153) out of the eleven revealed elevation in adipogenesis.  Nuclear receptor activation was assessed in transient transfection assay with mouse PPARα and mouse PPARγ (i.e., not human receptors). |
|  |  |  |  | Human *in vivo* (epidemiology; prospective birth cohorts) | N/A | N/A | No association | (Cai et al., 2023) | Data obtained from two Belgian cohort studies including cord blood measurements of five organochlorines (DDE, HCB, PCB-138, -150, −180; N = 1418) and two PFAS (PFOA and PFOS; N = 346). Endpoints: infant growth (BMI z-score change between birth and 2 years), and childhood growth (BMI trajectory from birth to 8 years).  Prenatal exposure to PCB-153 and DDE was associated with increased and decreased infant growth, respectively (up to 2 years old); no evidence for more persistent effects. |
|  |  |  |  | Human, *in vitro,*  hMSCs  PFOA (for cytotoxicity: 3 µM, 10 µM, 30 µM, 100 µM, 300 µM; , for gene expression: 3 µM, 30 µM, 100 µM)  72 h |  |  |  | (Burkhardt et al., 2024) | Positive control: ROSI (for cytotoxicity: 0.03 µM, 0.1 µM, 0.3 µM, 1 µM, 3 µM; for gene expression: 1 µM, 10 µM, 30 µM )  No effect on either overall glucose consumption, production of adipokines adiponectin and leptin, or lipid storage, size and total lipid content in the cell culture. Lipidomic analysis of cell culture medium revealed significant changes in the extracellular lipidome even no effects on any other studied endpoint observed. |
| **Triphenyl phosphate (TPP)** | 115-86-6 | 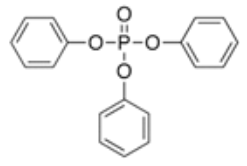 |  | **SUMMARY** | N/A (likely no) | Yes | Yes/ inducer from Filer et al 2022 predictions/experimental uncertainty |  | A report on the systematic literature review and weight-of-evidence expert elicitation of TPP inducing adipogenesis via the endocrine mode of action of PPARγ activation is being prepared (Beausoleil et al 2024, submitted).  14 *in vitro* studies providing data on PPARγ activation or adipogenesis, 3 *in vivo* studies providing mechanistic evidence (PPARγ activation), 5 *in vivo* studies linked to the adverse effect of obesity, and 2 epidemiological studies (summarised in Beausoleil et al 2024, submitted).  Diphenyl phosphate had no significant effect on cell proliferation and induced weaker adipogenesis.  References below are examples and not exhaustive. |
|  |  |  |  | *In vitro,* 3T3-L1 murine cell line,  (0.1-50 μM)  2-8 d | N/A | Yes (gene expression; induction only after 8 d exposure at all test concentrations (1-25 μM)) | Yes (at 10 & 25 μM; not at 50 μM) | (Cano-Sancho, Smith, & La Merrill, 2017) | Pre-adipocyte proliferation and subsequent adipogenic differentiation in 3T3-L1 cells was enhanced with TPP treatment, coinciding with elevated CEBP and PPARγ pathway transcription. TPP exposure in mature adipocytes increased the basal- and insulin stimulated- uptake of the glucose analog 2-NBDG. Inhibition of PI3K, a member of the insulin signalling pathway ablated this effect |
|  |  |  |  | Human *in vitro*, HG5LN reporter cell lines for PPARγ (10^-7^ – 10^-5^ M)  24h | N/A | No/ borderline (28% max activity, EC20/EC50 n.d.) | N/A | (Garoche et al., 2021) | Human, mouse, zebrafish, and *Xenopus* PPARγ were tested using known hPPARγ ligands, and environmental chemicals.  HPPARγ and mPPARγ showed similar activities, with marked differences in xPPARγ (less pronounced) and zfPPARγ (more pronounced).  Only data fro hPPARγ were extracted here (activity parameters for other receptors/ species is given in the publication). |
|  |  |  |  | Human or mouse *in vitro,*  (3T3-L1 murine preadipocytes or OP9 mBMSCs)  10 d  Primary human subcutaneous preadipocytes  14 d |  | Yes | Yes. In 3T3-L1 without dexamethasone and in OP9 more potent than ROSI | (Kim et al., 2021) | Endpoints: lipid accumulation, RNA-Seq and confirmation of gene expression by RT-qPCR  Data-driven hierarchical clustering approach to identify PPARγ modulating chemicals, and transcriptional changes related to differentiation into white or brite/beige adipose tissue.  The authors compared a strong PPARγ therapeutic agonist that also was shown to modify PPARγ phosphorylation (i.e., ROSI, a chemical that was shown to modify only PPARγ phosphorylation (i.e., roscovitine), a weak PPARγ agonist and endogenous molecule (i.e., 15dPGJ2), and two known environmental PPARγ ligands [i.e., TBBPA and TPhP].  Important genes for predicting PPARγ ligand/modification status, specifically the down-regulation of *Rpl13* and the upregulation of *Cidec*.  Max. Tested concentration: 2x10^-5^ M,  max. Non-toxic concentration: 1x10^-5^ M |
|  |  |  |  | Human, *in vitro,*  hMSCs  TPP (for cytotoxicity: 1 µM, 3 µM, 10 µM, 30 µM, 100 µM; for gene expression: 1 µM, 10 µM, 30 µM)  72 h |  |  |  | (Burkhardt et al., 2024) | Positive control: ROSI (for cytotoxicity: 0.03 µM, 0.1 µM, 0.3 µM, 1 µM, 3 µM; for gene expression: 1 µM, 10 µM, 30 µM )  30 µM TPP treatment increased adiponectin expression. |
| **Dichlorodiphenyldichloroethylene  (pp’-DDE)** | 72-55-9 | 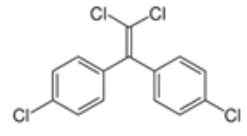 | Pesticide metabolite (Stockholm POPs list) | **SUMMARY** | No | No | Uncertain (potentially “No”) |  | Listed under the Stockholm Convention on Persistent Organic Pollutants (as metabolite of DDT).  p,p’-DDE is potentially weakly adipogenic in 3T3-L1 cells (30-100 µM), but the evidence is mixed from human (epidemiological) studies: no clear association with obesity. |
|  |  |  |  | Human and polar bear reporter cell lines, *in vitro,*  (0.5 pM – 25 μM)  24 h | Yes, weak activation (16% activity at 25 μM in h . Not active in pbPPARα) | N/A | N/A | (Routti et al., 2019) | The authors transiently co-transfected COS7 cells with pCMX-GAL4-pbPPARα or pCMX-GAL4-hPPARα, tk(MH100)x4-luciferase, and pCMV-β-galactosidase. Activities of environmental contaminants were established against the known PPARα agonist WY-14643 and antagonist MK-886.  Polar bear PPARα (pbPPARα) was both quantitatively and qualitatively more susceptible than human PPARα (hPPARα) to transactivation by less lipophilic chemicals. |
|  |  |  |  | *In vitro,* 3T3-L1 murine cell line  4,4-DDE (0.3 μM, 1 μM, 3 μM, 10 μM, 30 μM, 100 μM for NR activation and 20 μM, 50 μM, 75 μM for lipid accumulation)  6 d | No/ potentially antagonism | No/ potentially antagonism | No. | (Taxvig et al., 2012) | Four chemicals (BPA, mono-ethylhexyl phthalate, butylparaben, PCB 153) out of eleven showed an elevation in adipogenesis.  Nuclear receptor activation was assessed in transient transfection assays with mouse PPARα and mouse PPARγ (i.e., not human receptors).  Significant decrease in mPPARα and PPARγ-mediated luciferase signal was observed upon exposure to 30 μM CPF only. |
|  |  |  |  | *In vitro*, 3T3-L1 murine preadipocytes  (10-20 μM) 8 d |  | Yes (gene expression; 2-3-fold increase vs. Vehicle ontrol) | Yes, likely weak/ borderline | (J. Kim et al., 2016) | Increased expression of C/EBPα, PPARγ, FAS, ACC, adipose triglyceride lipase, and leptin.  Triglyceride content per cell was quantified (mg TG per mg total protein). Only 20 μM DDT induced significant increase in TG, however both test concentrations (10 and 20 μM) DDE induced significantly elevated TG levels. However, the margin was narrow: TG content vehicle control ~0.6, DDE ~0.7. |
|  |  |  |  | *In vitro*, 3T3-L1 murine preadipocytes  (1-100 μM)  8 d |  | No (2.5-20 μM; n.s. compared to vehicle control) | Yes (LOEC = 10 μM; up to ~1.5-fold) | (Mangum, Howell, & Chambers, 2015) | DDE exposure induced a concentration dependent elevation in intracellular neutral lipid accumulation accompanied by upregulation of genetic markers of differentiation (incl. FABP4, Srebp1c, leptin, Fas) as determined by Oil Red O staining and triglyceride assay.  Also, increased protein levels of C/EBPα, PPARγ, AMPKα, and ACC, while significant decrease of phosphorylated forms of AMPKα and ACC were observed. |
|  |  |  |  | Human *in vivo* (epidemiology, prospective youth cohort, n=318) | N/A | N/A | N/A (but indication on lowering serum leptin) | (Burns et al., 2014) | Monitored chemicals: DDE, hexachlorobenzene, β-hexachlorocyclohexane  Russian boys (n=499, enrolled during 2003-2005, age 8-9 years) with subsequent repeated measurements of serum glucose, insulin, lipids, leptin, and calculated homeostatic model assessment of insulin resistance (IR). N=318 subjects had baseline organochlorine pesticides and serum biomarkers re-measured at ages 10-13 years.  Higher DDE (quintile 5 vs. Quintile 1) was associated with **lower leptin**, with relative mean decreases of 61.8% (95%CI: 48.4%, 71.7%) in models unadjusted for BMI and 22.2% (95% CI: 7.1%, 34.9%) in models adjusted for BMI; the direct effect of DDE on leptin accounted for 27% of the total effect. |
|  |  |  |  | Mouse *in vivo* (C57BL/6 male mice, n=8/group, 8 weeks, 1 mg DDE/kg bw/d (oral gavage)) | N/A | N/A | N/A | (Liu et al., 2017) | Accumulation of DDE and β-HCH in organs, hepatic fatty acid composition, tricarboxylic acid cycle (TCA) metabolites and other metabolite profiles were analysed; gene expression for hepatic lipogenesis (DDE: increased) and beta-oxidation (DDE: decreased) was measured.  DDE accumulated in liver, alongside damaged mitochondria and changed hepatic lipid profile/ fatty acid composition.  In HepG2 cells, exposure to 1 or 10 mg DDE/mL did not significantly increase lipid accumulation (though a slight, non-significant increase was observed). |
|  |  |  |  | Human *in vivo* (epidemiology; prospective birth cohorts) | N/A | N/A | Uncertain/ not sustained | (Cai et al., 2023) | Data obtained from two Belgian cohort studies including cord blood measurements of five organochlorines (DDE, HCB, PCB-138, -150, −180; N = 1418) and two PFAS (PFOA and PFOS; N = 346). Endpoints: infant growth (BMI z-score change between birth and 2 years), and childhood growth (BMI trajectory from birth to 8 years).  Prenatal exposure to PCB-153 and DDE was associated with increased and decreased infant growth, respectively (up to 2 years old); no evidence for more persistent effects. |
|  |  |  |  | Human, *in vitro*, (human adipose-derived MSCs,  0.1-10 μM,  4-28 d | N/A | No (or only increased at 0.1 μM and only on day 10) | Uncertain/ no | (Pesta et al., 2018) | Gene expression and western blot analysis of markers of adipogenesis/adipocyte differentiation, lipid metabolism, and insulin signaling on days 0, 4, 10, 21, 28 od differentiation.  Significant increase of INSR, LIPE, FASN, SREBP1, OCT4 and AKT2 (not increased abundance of active Akt (Akt p-Ser473)) upon DDE exposure.  If there is a role of DDE in developing obesity in humans, this is likely by DDE affecting the insulin signalling pathway: effects of DDE were similar to effects of insulin itself.  Effects on lipid accumulation were not quantified/evaluated. However, phase-contrast microscopy images do not show a substantial difference between baseline differentiation and DDE exposure/addition. |
|  |  |  |  | Human *in vivo* (epidemiology; prospective cohort, n=571 75-year old Swedes) | N/A | N/A | N/A  (DDE associates wich plasma lipiproteins of different classes) | (Jugan et al., 2020) | Study subjects were a subset of the Swedish cohort *Prospective Investigation of the Vasculature of Uppsala Seniors (PIVUS)*, who were not prescribed lipid lowering medication.  Detectable levels of p,p’-DDE were determined in the plasma samples of all subjects. Increased p,p’-DDE levels were related to elevated concentrations of lipoproteins of all diameters, except for high density lipoprotein (HDL) of diameters between 14.3 nm–10.9 nm. Of the lipoprotein components, triglycerides were most uniformly associated with elevated p,p’-DDE across lipoproteins. P,p’-DDE was also associated with apolipoprotein B, but not apolipoprotein A1. |
|  |  |  |  | Human *in vivo* (epidemiology; prospective cohort, n=1016 70-year old adults) |  |  |  | (Salihovic et al., 2016) | Study subjects were a subset of the Swedish cohort *Prospective Investigation of the Vasculature of Uppsala Seniors (PIVUS)*, who were not prescribed lipid lowering medication. Associations between DDE and HCB exposure/plasma levels with global metabolomic profiles in serum samples were analysed.  Circulating levels ofp,p-DDE and HCB to be significantly associated with circulating levels of16 metabolites, belonging mostly to lipid metabolism pathways (incl. Fatty acids, glycerophospholipids, sphingolipids, and glycerolipids). |
|  |  |  |  | Human, *in vivo* (epidemilogy, n=54 adults) | N/A | N/A | Uncertain (possibly some extent of adipocyte hypertrophy, but **no association with obesity** observed. Strongest effects in lean women) | (Rolle-Kampczyk et al., 2020) | 54 individuals (30 women, 24 men) with a wide range of body mass index (BMI, 16–70 kg/m^2^) during laparoscopic abdominal surgeries. Chemicals analysed were: Propofol, 2-phenyltetralin, hexachlorobenzene, PCB153, DDE, ethyl tetradecanoate, 4,4-diisopropylbiphenyl, phytol acetate, hexachlorobiphenyl/ PCB X.  Adipose tissue concentrations of these chemicals are not significantly different between visceral and subcutaneous fat depots nor among women and men. However, the  bio-accumulation of different POPs in adipose tissue is significantly correlated with adipose tissue macrophage infiltration, adipocyte size and parameters of glucose metabolism. In both fat depots, the strongest correlations of POPs (Ethyl- tetradecanoate, 4,4′-Diisopropylbiphenyl, 2-Phenyltetralin, PCB153, Hexachlorobenzene) and adipose tissue macrophage infiltration were detected in lean individuals. Additional significant associations between adipose tissue POPs and glycemia parameters, insulin sensitivity, and inflammation suggest that **specific environmental chemicals may contribute to adipose tissue dysfunction, adipocyte hypertrophy, impaired glucose metabolism, systemic inflammation and variability in fat distribution, however not to obesity**.  Limitations of the study to detect effects between different subgroups (e.g., women/men, BMI subgroups) due to cohort size are acknowledged. |
|  |  |  |  | Rat *in vivo* (4-week old male Sprague Dawley rats, n=8/group, 2 mg/kg bw/d (oral gavage), 21 d) | N/A | N/A | Uncertain, likely yes (increased total body and fat pad weight) | (Liang et al., 2020) | p,p, DDE exposure increased body weight and fat content and impaired glucose homeostasis (oral glucose tolerance on day 14; DDE induced insulin resistance). DDE induced gut dysbiosis as indicated by an increased Firmicutes-to-Bacteroidetes ratio, which may impact energy harvest efficiency. Meanwhile, the plasma lipid metabolome profile was significantly altered by DDE. Furthermore, phosphatidylcholine, phosphatidylserine, phosphatidylethanolamine, and triacylglycerol were identified as key metabolites affected by DDE treatment, and these altered lipid metabolites were highly correlated with changed microbiota composition. |
|  |  |  |  | Human, *in vitro,*  hMSCs  pp-DDE (for cytotoxicity: 0.3 µM, 1 µM, 3 µM, 10 µM, 30 µM; for gene expression: 1 µM, 10 µM, 30 µM)  72 h |  |  |  | (Burkhardt et al., 2024) | Positive control: ROSI (for cytotoxicity: 0.03 µM, 0.1 µM, 0.3 µM, 1 µM, 3 µM; for gene expression: 1 µM, 10 µM, 30 µM )  No effect on either overall glucose consumption, production of adipokines adiponectin and leptin, or lipid storage, size and total lipid content in the cell culture. Lipidomic analysis of cell culture medium revealed significant changes in the extracellular lipidome even no effects on any other studied endpoint observed. |
| **Triclosan (TCS)** | 3380-34-5 | 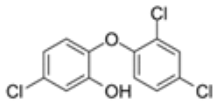 | Bacteriocide | **SUMMARY** | No | No | Uncertain (possibly “No”) |  | Some indication for interspecies differences related to PPARα activity (no activity for hPPARα, but possibly weakly active on mouse PPARα). Inducer suggested by Filer 2022 (Filer et al., 2022) prediction (also predicted active on PPARy: potency=0.34).  Human studies: while some studies indicate adipogenesis potential, overall, the weight of evidence suggests that TCS does not induce obesity/ adipogenesis/ lipid accumulation in adipocytes, in humans. |
|  |  |  |  | Human, *in vivo* (analytical method development and application, n=21 serum samples) |  |  | N/A | (Dirtu et al., 2008) | BPA (0.71 ng mL−1) and TCS (0.52 ng mL−1) median concentrations in Belgian human serum samples were in line with data for human fluids which is previously reported. Slightly increased levels of TBBPA (0.08 ng mL−1) were determined in Belgium serum samples compared to Norwegian. |
|  |  |  |  | Human, *in vivo* (epidemiology, prospective cohort) | N/A | N/A | No/ decrease | (Zamora et al., 2021) | Urinary metabolites (phthalate, phenols, parabens) were collected in 2008 among 73 females (ELEMENT study, Mexico); metabolic syndrome and its elements (abdominal obesity, hypertriglyceridemia, cholesterolemia, hypertension, hyperglycemia) were evaluated in 2017. Mean age at follow-up: 46.6 years, prevalence of metabolic syndrome at follow-up: 34.3%.  The odds of hyperglycaemia were 0.46 (95% CI: 0.18, 1.17 p < 0.10) times lower for every IQR increase in the sum of di-2-ethylhexyl phthalate metabolites (ΣDEHP). |
|  |  |  |  | Zebrafish *in vivo* (up to 5 d, 250 ug/L) | N/A | N/A | No | (Ho et al., 2016) | Four developmental stages probed by in situ hybridisation staining: 70–85% epiboly, 10–12 somite, prim-5, and 5 dpf.  Effects investigated on: dorsal ventral patterning, segmentation, brain development, and organ formation.  Neither phenotypic nor molecular changes were found after 5 days of 250 ug/L TCS exposure. However, lipid droplet accumulation in the yolk sac was observed. |
|  |  |  |  | Amphibian, *in vivo* (female *Xenopus tropicalis,* 50 ng/L, from tadpole stage throughout life cycle) | N/A (possibly: no effect) | N/A (possibly: no effect) | No/ decrease | (Regnault et al., 2018) | Frogs displayed glucose intolerance syndrome, liver steatosis, liver mitochondrial dysfunction, liver transcriptomic signature and pancreatic insulin hyper secretion typical of a pre-diabetes state.  No effects on peroxisome pathways detected (transcriptomic analysis). |
|  |  |  |  | Human, *in vivo* (epidemiology/ biomonitoring, human adipose fat samples, n=20) | N/A | N/A | N/A | (Wang, Asimakopoulos, & Kannan, 2015) | Human adipose fat samples (N= 20) collected from New York City, USA, were analysed for the presence of environmental phenols, including BPA, benzophenone-3 (BP-3), TCS, and parabens, as well as heterocyclic aromatic compounds, including benzotriazole (BTR), benzothiazole (BTH), and their derivatives.  BPA and TCS were frequently detected in adipose tissues at concentrations (geometric mean [GM]: 3.95 ng/g wet wt for BPA and 7.21 ng/g wet wt for TCS) similar to or below the values reported for human urine. |
|  |  |  |  | Human, *in vivo* (epidemiology, n=144, EuroMix project) | N/A | N/A | N/A | (Husøy et al., 2019) | 44 male and 100 female volunteers kept detailed diaries on their food consumption, personal care product use and handling of cash receipts. Urine samples were collected during the same 24-hour period and urinary levels of four parabens, five bisphenols, oxybenzone/benzophenone-3 (OXBE), TCS, triclocarban (TCC) and metabolites of eight phthalates and 1,2-cyclohexane dicarboxylic acid diisononyl ester (DINCH) were analysed.  The detection rate for the metabolites of phthalates and DINCH, and BPA and TCS in urine was 88–100%. |
|  |  |  |  | Human, *in vivo* (epidemiology, cross-sectional, n=79 children & adolescents aged 6-18, Iran, 2020) | N/A | N/A | Uncertain/ potentially yes | (Nasab et al., 2022) | Urine levels of TCS, methyl-TCS, triclocarban, and 2.4-dichlorophenol and their relation with anthropometric and demographic parameters were measured.  TCS urinary concentration: 4.32±2.08 ug/L (geometric mean).  All chemicals have a positive and significant association with BMI z-score and BMI (p-value < 0.01). TCS and methyl-TCS have a positive, strong, and substantial association (p-value < 0.01, r = 0.74). However, no significant association with waist circumference was evident.  It is not clear if/ to which extent data were corrected for demographic/socio-economic parameters. E.g., Parental education seems to significantly affect urinary TCS levels, especially in boys. |
|  |  |  |  | Human *in vivo* (meta-analysis of epidemiological studies) | N/A | N/A | No | (Liu et al., 2021) | Following a systematic literature search in PubMed, Cochrane Library, and Web of Science, seven epidemiological studies (n=5006 participants) from September 2014 – August 2018 were included for association of maternal exposure and neonatal birth weight. Three studies (n=5213 participants, July 2014 – September 2017) were identified for association of children’s exposure and children’s BMI.  Prenatal exposure was not associated with neonatal birth weight; children’s urinary TCS levels were not associated with altered BMI. |
|  |  |  |  | Human *in vivo* (epidemiology, prospective birth cohort, n=220 mother-child pairs) | N/A | N/A | No | (Kalloo et al., 2018) | Subjects recruited from Cincinnati, Ohio, USA. TCS was quantified in urine samples collected twice during pregnancy (maternal urine), then in children annually from 1 to 5 years of age, and once at 8 years. At 8 years old, child adiposity (BMI, waist circumference, and bioelectric impedance) were determined.  Overall, there was no association between TCS and child adiposity. |
|  |  |  |  | Human *in vivo* (epidemiology, cross-sectional child cohort, n=423 7-year-old children) |  |  | Yes (positive association with BMI z-score and %body fat) | (Hu et al., 2022) | Laizhou Wan Birth Cohort in Shandong, northern China. TCS was determined in spot urine samples.  TCS concentrations, when treated as continuous variables, were positively associated with BMI z-score (β = 0.12, 95% CI: 0.01, 0.24) and body fat percentage (β = 0.82, 95% CI: 0.13, 1.52). Further, higher TCS levels were associated with an approximate 2–3 fold increased risk of abdominal obesity (p-trend = 0.044). |
|  |  |  |  | Human *in vivo* (epidemiology, n=4037 adults) | N/A | N/A | Yes (increased BMI; stronger effects at low/moderate than at high TCS levels) | (Lankester et al., 2013) | US NHANES adult cohort (2003-2008).  Detectable TCS urine level was associated with a 0.9-point increase in BMI (p<0.001).  The 2nd, 3rd and 4th quartiles of urinary TCS compared to the lowest quartile were associated with BMI increases of 1.5 (p,0.001), 1.0 (p = 0.002), and 0.3 (p= 0.33) respectively. TCS metabolite 2,4-dichlorophenol was correlated with urinary TCS levels, but association with BMI was weaker than for TCS.  Possible effects of TCS on BMI via modulation of the microbiome are identified as a future research need. |
|  |  |  |  | Review with a focus on molecular pathways responsive to TCS | Uncertain (upregulated in zebrafish, but no appreciable induction in mice) | Possibly yes | Uncertain/ no | (Alfhili & Lee, 2019) | May induce oxidative stress, pro-inflammatory processes.  Signalling pathways affected by TCS exposure include endocrine pathways (estrogen & androgen signalling), cell proliferation/apoptosis/tumorigenesis (including via CAR activation).  Limited evidence for PPARα and PPARγ activity/induction: upregulation observed in *Danio rerio* and *Gallus gallus* embryo livers.  Differential modulation of TCS on PPARα in HepG2 cells and mouse hepatoma Hepa1c1c7 cells, distinct responses were observed by (Wu et al., 2014) including on PPARα-downstream target protein acyl-coenzyme A oxidase (decreased in HepG2, increased in Hepa1c1c7, higher DNA synthesis and blunted apoptosis through transforming growth factor (TGF-β) in rodent cell line). |
|  |  |  |  | Human and mouse *in vitro,* (human HepG2 and mouse Hepa1c1c7 hepatoma cells  (0.1-20 μM)  24-96 h | **No/ decrease for hPPARα**,  Yes mPPARα | N/A | N/A | (Wu et al., 2014) | Reporter gene assays for human and mouse PPARα activation, and investigation of PPARα downstream events, especially ACOX1 protein levels (increased in Hepa1c1c7 cells but decreased in HepG2 cells). Cytotoxicity was similar in both cell lines (EC50 > 20 μM; viability > ~85% up to 10 μM), despite differential PPARα effects.  PPARα **transcriptional activity was increased by TCS in a mouse PPARα reporter assay** (EC50 = 9.9 ± 1 μM) **and decreased in a human PPARα reporter assay** (IC50 = 14.3 ± 1 μM). Abundance of PPARα protein itself was not altered b y TCS. |
|  |  |  |  | Mouse, *in vivo*  male C57BL/6 mice, n=6/group, 6 week old, 0.08% TCS in chow diet,  8 m | No (mPPARα) | N/A | No (body weight not increased over 8 months) | (Yueh et al., 2020) | Wildtype and *Car*-null (*Car^-/-^*) mice strains were used.  Xenobiotic receptor screening was done at 10 μM TCS (24 h) in CV-1 cells for PXR, CAR, LXRα, FXR, VDR, PPARα, PPARβ, PPARγ ERα, ERβ, GR. **Only CAR** (i.e., not PPARα) was modestly activated (~2-fold). Further investigation indicated, that TCS is a CAR activator, but not a direct ligand for either mouse or human CAR.  TCS treatment increased hepatocyte proliferation, induced liver fibrosis, hepati inflammation, and ROS accumulation in mouse liver. Further, TCS increased the number (~4.5-fold) and size (~3.5-fold) of detectable hepatocellular carcinoma (upon stimulation/predisposition of male mice by a single i.p. dose of diethyl nitrosamine at 15 d old), independent of CAR. |
|  |  |  |  | Review for regulatory purposes (including GLP studies) | Uncertain (yes in mice, not in hamsters) | N/A | N/A | (SCCP, 2009) | In rodents (mice and rats), some evidence indicates PPARα agonism, especially at high doses, concluding that TCS is a peroxisome proliferator in mice. However, this was not observed/ replicated in hamster studies: in hamsters, the lack of induction of peroxisomal fatty acid oxidation and morphological evidence suggests that TCS is not a peroxisome proliferator. |
|  |  |  |  | Human, *in vitro,*  hMSCs  TCS (for cytotoxicity: 0.3 µM, 1 µM, 3 µM, 10 µM, 30 µM, for gene expression: 1 µM, 10 µM, 30 µM)  72 h |  |  |  | (Burkhardt et al. 2023) | Positive control: ROSI (for cytotoxicity: 0.03 µM, 0.1 µM, 0.3 µM, 1 µM, 3 µM; for gene expression: 1 µM, 10 µM, 30 µM )  30 µM TCS affected the morphologically assessed lipid storage and adipocyte size, as well as glucose consumption and mRNA expression of the glucose transporter GLUT1, leptin and adiponectin.  30 µM TCS treatment induced leptin expression. |
| **Rosiglitazone (ROSI)** | 122320-73-4 | 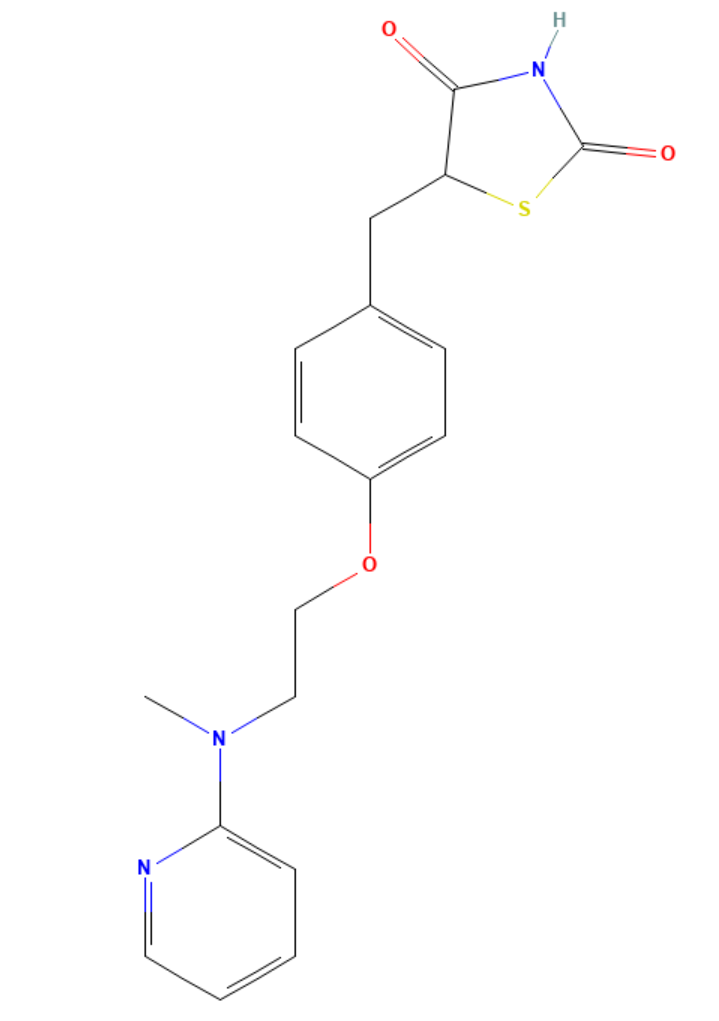 | **Pharmaceutical** | **SUMMARY** | No/ weak | **Yes, positive control** | **Yes, positive control** |  | **Standard positive control for PPARγ agonism and lipid accumulation/ adipogenesis studies.**  Prototypical PPARγ agonist; adipogenesis mediated via PPARγ.  However, interspecies differences have been reported: e.g., ROSI does not induce lipid accumulation nor does it activate PPARγ in zebrafish. In Filer et al 2022 (Filer et al., 2022) predicted as negative.  References below are not exhaustive; as, ROSI is the prototypical positive control in most (human and murine) *in vitro* adipogenesis test systems/ models. |
|  |  |  |  | Mouse *in vitro,* reporter cell lines  3T3-L1 murine preadipocytes  ROSI (1 μM)  8 d |  | Yes (LOEC hPPARγ: 10^-8^ M) | Yes | (Riu et al., 2011) | Human, zebrafish, and *Xenopus* PPARγ reporter plasmids were constructed for transient transfection into HeLa cells. Additionally, the following stably transfected reporter cell lines were utilised: HGELN, HGELN­ERα, HGELN­ERβ, and HGELN­GAL-PPARγ. For PPARγ, 100 nM ROSI were used as positive control/reference.  Species comparison of PPARγ activation: 1 μM ROSI resulted in ~8.5-fold activation of hPPARγ, and activated xPPARγ to a similar extent (~7.5-fold). However, **zPPARγ was not activated** and remained on the levels of vehicle control.  10 μM TBBPA induced lipid accumulation in 3T3-L1 murine preadipocytes as 1 μM ROSI (positive control). |
|  |  |  |  | Zebrafish larvae (28 hpf wildtype AB/Tuebingen and transgenic *Tg(hPPARy-eGFP)*, 3x10^-10^ M – 10^-6^ M, 24 h) and reporter cell lines |  | **No** for zPPARγ,  Yes for hPPARγ | No | (Riu et al., 2014) | LT-hPPARγ transgenic zebrafish (*Tg(hPPARy-eGFP)*): eGFP is expressed in the presence of active hPPARγ ligands (no cross-activation by RXR ligands).  28 hpf TG zebrafish embryos exposed to chemicals for 24 h for eGFP signal detection.  For late-onset weight gain, ZF were exposed to chemicals from 3-11 dpf (10 nM & 1 μM ROSI) and assessed at 11 dpf (ORO staining, do difference to vehicle control). |
|  |  |  |  | *In vitro*, 3T3-L1 murine cell line  (0.3 μM, 1 μM, 3 μM, 10 μM, 30 μM, 100 μM for NR activation and 1 μM, 5 μM, 10 μM for lipid accumulation)  6 d | N/A | Yes (LOEC 30 μM) | Yes (1 μM, lowest test concentration, >4-fold lipid accumulation induction) | (Taxvig et al., 2012) | Four chemicals (BPA, mono-ethylhexyl phthalate, butylparaben, PCB 153) out of the eleven revealed elevation in adipogenesis.  Nuclear receptor activation was assessed in transient transfection assays with mouse PPARα and mouse PPARγ (i.e., not human receptors). |
|  |  |  |  | *In vitro*, 3T3-L1 murine preadipocytes  (1.92-1000 nM)  10 d | N/A | Yes, strong (hPPARγ CALUX assay; LOEC = 30 nM) | Yes, stong (LOEC: with insulin = 16 nM, without insulin = 31 nM) | (Pereira-Fernandes et al., 2013) | This is the first description and successful demonstration of Nile Red fluorescence for the quantification of lipid accumulation in 3T3-L1 cells, including the statistical derivation of a statistically robust fold-change classification threshold for non- weak and strong obesogenic chemicals.  Two exposure scenarios were tested: exposure with or without insulin in exposure medium. Positive control: 2 d MDI hormone cocktail induction followed by 8 d insulin-only stimulation.  The assay and classification threshold was derived using TBT and ROSI.  Quality criteria for test acceptance:   - MDI-positive control: degree of lipid accumulation (DLA) > 10 (> 4 for insulin co-exposure scenario) - Lipid accumulation threshold (LAT; equivalent to analytical chemistry LoQ): blank value [solvent control] + 10x[SD solvent control over all experiments] (LAT is based on variation observed in solvent control) - For single chemical treatment: LAT = 1.76 - For insulin co-exposure: LAT = 2.17   **Chemicals inducing a statistically significant DLA in at least 2 subsequent concentrations compared to the solvent control, but lower than the LAT are considered weak obesogens, whereas chemicals inducing a significant DLA higher than the LAT are considered strong obesogens**.  For the “strictly standardised mean difference (SSMD) method”, obesogens inducing a SSMD value higher than 4.7 are considered strong obesogens, whereas chemicals inducing SSMD value between 2 and 4.7 are selected as weak obesogens (SSMD < 2 = non-obesogen).  The reference chemicals TBT and ROSI induced 3T3-L1 differentiation in a concentration-dependent manner, with or without insulin; ROSI to a greater extent/magnitude than TBT.  Adipogenic potential was shown for all tested parabens, several musks and phthalate compounds and BPA. PPARγ activation was related to adipogenesis for parabens, phthalates and BPA, however not required for Tonalide induced obesogenic effects. DLA max at 1 μM: 5.8 without insulin, 3.69 with insulin. |
|  |  |  |  | Human *in vitro,* HG5LN reporter cell lines for PPARγ (10^-10^ – 10^-6^ M)  24h | N/A | Yes (EC20: 9.2 nM, EC50: 24 nM, ref. Chemical) | N/A | (Garoche et al., 2021) | Human, mouse, zebrafish, and *Xenopus* PPARγ were tested using known hPPARγ ligands, and environmental chemicals.  HPPARγ and mPPARγ showed similar activities, with marked differences in xPPARγ (less pronounced) and zfPPARγ (more pronounced).  Only data from hPPARγ were extracted here (activity parameters for other receptors/ species is given in the publication). |
|  |  |  |  | I*n vitro,* 3T3-L1 murine preadipocytes, 10-200 nM, 16 d | N/A | N/A | Yes | (Watkins et al., 2015) | All test concentrations significantly (compared to solvent control) increased cell number, DNA content, and total triglyceride content, and decreased cell size. |
| **Mono-(2-Ethylhexyl) Phthalate (MEHP)** | 4376-20-9 | 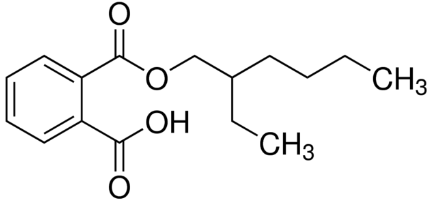 | **Phthalate, plasticiser** | **SUMMARY** | Yes, moderate agonist | Yes, moderate agonist | Yes |  | Active metabolite of DEHP.  Cytotoxic at higher concentrations in adipogenesis assay.  Potential for interspecies differences – esp. related to PPARα (inactive for hPPARα, while active in mPPARα)  Activity via PPARγ is mediated via the metabolite, MEHP, not the parent chemical DEHP. (*in vitro)* models with limited metabolic capacity/competence may therefore miss MEHP-effects when DEHP was tested, while *in vivo* models are more likely to inform on both, DEHP and MEHP-mediated effects.  Adipogenic effects supported by Filer et al 2022 for 3T3 L1. |
|  |  |  |  | *In vitro,* murine mesenchymal stem cell line C3H/10T1/2 and embryonic stem cells (CGR8)  DEHP (100 nM and 100 μM)  Undifferentiated growth (0-6 days)  Induction (6-8 days)  Terminal differentiation (8-12 days) |  |  |  | (Biemann et al., 2012) | The final amount of differentiated adipocytes, cellular triglyceride content and mRNA expression of adipogenic marker genes (adiponectin, FABP4, PPARγ2, LPL) were measured and compared with corresponding unexposed cells. DEHP (100 μM) increased adipogenesis during the hormonal induction period.  Exposure of undifferentiated murine embryonic stem cells was demonstrated no effect on subsequent adipogenic differentiation of the investigated EDC. |
|  |  |  |  | Human *in vitro,* reporter cell line (HGELN­GAL-PPARγ, 10^-7^ – 3x10^-5^ M)  16 h |  | Yes (max. Effect at ~3x10^-5^ M, LOEC: 3x10^-6^ M) | N/A | (Riu et al., 2011) | For PPARγ, 100 nM ROSI were used as positive control/reference. |
|  |  |  |  | *In vitro*, 3T3-L1 murine cell line  ROSI (0.3 μM, 1 μM, 3 μM, 10 μM, 30 μM, 100 μM for NR activation and 1 μM, 5 μM, 10 μM for lipid accumulation)  6 d | Yes (LOEC 3 μM) | Yes (LOEC 10 μM) | Yes (LOEC 10 μM) | (Taxvig et al., 2012) | Four chemicals (BPA, mono-ethylhexyl phthalate, butylparaben, PCB 153) out of the eleven revealed elevation in adipogenesis.  Nuclear receptor activation was assessed in transient transfection assay with mPPARα and mPPARγ (i.e., not human receptors). |
|  |  |  | DEHP | *In vitro*, 3T3-L1 murine preadipocytes  (0.1-50 μM)  10 d | N/A | Yes, weak (hPPARγ CALUX assay; LOEC = 3 μM) | No | (Pereira-Fernandes et al., 2013) | This is the first description and successful demonstration of Nile Red fluorescence for the quantification of lipid accumulation in 3T3-L1 cells, including the statistical derivation of a statistically robust fold-change classification threshold for non- weak and strong obesogenic chemicals.  Two exposure scenarios were tested: exposure with or without insulin in exposure medium. Positive control: 2 d MDI hormone cocktail induction followed by 8 d insulin-only stimulation.  The assay and classification threshold were derived using TBT and ROSI.  Quality criteria for test acceptance:   - MDI-positive control: degree of lipid accumulation (DLA) > 10 (> 4 for insulin co-exposure scenario) - Lipid accumulation threshold (LAT; equivalent to analytical chemistry LoQ): blank value [solvent control] + 10x[SD solvent control over all experiments] (LAT is based on variation observed in solvent control) - For single chemical treatment: LAT = 1.76 - For insulin co-exposure: LAT = 2.17   **Chemicals inducing a statistically significant DLA in at least 2 subsequent concentrations compared to the solvent control, but lower than the LAT are considered weak obesogens, whereas chemicals inducing a significant DLA higher than the LAT are considered strong obesogens**.  For the “strictly standardised mean difference (SSMD) method”, obesogens inducing a SSMD value higher than 4.7 are considered strong obesogens, whereas chemicals inducing SSMD value between 2 and 4.7 are selected as weak obesogens (SSMD < 2 = non-obesogen). |
|  |  |  |  | Human *in vitro,* HG5LN reporter cell lines for PPARγ (10^-7^ – 3x10^-5^ M)  24 h | N/A | Yes (EC20: 4.8 μM, EC50: 18 μM, 55% max activity) | N/A | (Garoche et al., 2021) | Human, mouse, zebrafish, and *Xenopus* PPARγ were tested using known hPPARγ ligands, and environmental chemicals.  hPPARγ and mPPARγ showed similar activities, with marked differences in xPPARγ (less pronounced) and zfPPARγ (more pronounced).  Only data from hPPARγ were extracted here (activity parameters for other receptors/ species given in the publication). |
|  |  |  | DEHP, MEHP | Human, *in vivo* (epidemiology, prospective cohort) | N/A | N/A | Uncertain/ no | (Zamora et al., 2021) | Urinary metabolites (phthalate, phenols, parabens) were collected in 2008 among 73 females (ELEMENT study, Mexico); metabolic syndrome and its elements (abdominal obesity, hypertriglyceridemia, cholesterolemia, hypertension, hyperglycemia) were evaluated in 2017. Mean age at follow-up: 46.6 years, prevalence of metabolic syndrome at follow-up: 34.3%.  The odds of hyperglycaemia were 0.46 (95% CI: 0.18, 1.17 p < 0.10) times lower for every IQR increase in the sum of di-2-ethylhexyl phthalate metabolites (ΣDEHP). |
|  |  |  | DEHP | Mouse *in vivo* (C57BL6J wild-type and genetical engineered mice, 500 mg/kg feed)  13 w | No (hPPARα),  Yes (mPPARα) | No/ uncertain | No (but also decreased food intake from week 4) | (Feige et al., 2010) | While DEHP-treated mice were protected from diet-induced obesity through PPARα-dependent activation of hepatic fatty acid catabolism, the activity of neither PPARβ nor PPARγ was affected.  However, the poor phenotype observed in response to DEHP in wild-type mice was surprisingly not observed in PPARα-humanized mice. These species differences are related with a different coregulator recruitment pattern. |
|  |  |  | DEHP, MEHP, ROSI | Review | Yes (MEHP only) | Yes (MEHP, but not the parent chemical DEHP) | N/A | (Desvergne, Feige, & Casals-Casas, 2009; Feige et al., 2007; Zoete, Grosdidier, & Michielin, 2007) | Co-crystallisation of PPARγ with ROSI or MEHP revealed similar binding patterns and interaction points with the PPARγ ligand binding domain. In contrast, the parent chemical DEHP was neither able to enter the ligand binding pocket of PPARγ, nor to maintain stable contacts with the required protein moieties.  The DEHP metabolite MEHP, but not the parent chemical, can activate PPARα as well as PPARγ. |
|  |  |  | DEHP, MEHP, MEOHP | Human, *in chemico*/ *in silico* | N/A | Yes (MEHP, not DEHP; EC10 = 1.2 μM)) | N/A | (Kratochvil et al., 2019) | Hydrogen/deuterium exchange mass spectrometry and docking, and surface plasmon resonance analysis of molecular binding to PPARγ were analysed for DEHP and its metabolites MEHP and MEOHP.  Binding to the ligand binding pocket of PPARγ was confirmed for the two metabolites MEHP and MEOHP, but not for the parent chemical DEHP. Upon binding of MEHP or MEOHP, conformational changes of the receptor resemble those observed with activation by the endogenous ligand 15-deoxy-delta-12,14-prostaglandin J2. This productive agonism was also confirmed by GeneBLAzer® PPARγ transactivation assay. For MEHP cytotoxicity IC_10_ of 203 µM and PPARγ activation EC_10_ of 1.2 µM were derived. |
|  |  |  | DEHP and 7 metabolites | Human, *in vivo* (epidemiology; subpopulation of NHANES study, n=6,005 women) | N/A | N/A | Yes (BMI and waist circumference) | (Yaghjyan et al., 2015) | Nonpregnant women aged ≥18 years without a history of diabetes were included in this study; seven urinary phthalate metabolites were monitored and correlated with BMI, waist circumference, total cholesterol, triglycerides, HDL-C and LDL-C. All examined metabolites are metabolites of DEHP.  BMI was associated positively with monobutyl phthalate (MBP) and mono-2-ethylhexyl phthalate (MEHP) (odds ratio (OR) = 1.13; 95% confidence interval (CI), 1.03–1.23 and OR= 1.12; 95% CI, 1.03–1.23, respectively). Waist circumference was associated positively with MBP (OR = 1.13; 95% CI, 1.03–1.24). A higher ratio of MEHP to mono-(2-ethyl-5-hydroxyhexyl) phthalate (MEHHP) was positively associated with both BMI (OR= 1.21; 95% CI, 1.09–1.34) and waist circumference (OR = 1.20; 95% CI, 1.10– 1.31). No other significant association observed. |
|  |  |  |  | Human, *in vivo* (epidemiology. N=97 pregnant women) | N/A | N/A | N/A | (Waits et al., 2020) | Urinary phthalate metabolites in the late third trimester were associated with oxidative/nitroso-active stress biomarkers |
|  |  |  |  | Human, *in vivo* (epidemiology, n=2,884 non-pregnant children aged 6-19 years; subpopulation of 2003-2008 NHANES) | N/A | N/A | No | (Trasande et al., 2013) | Urinary phthalate metabolites with low molecular weight were associated with increased odds of overweight, obesity and increased BMI z-score among non-hispanic black children. High molecular weight phthalates and DEHP metabolites did not show significant associations, and no significant associations were detected among other ethnic groups. |
|  |  |  |  | Human, *in vivo* (epidemiology; Australian Barwon infant study, n=841 pregnant women, urine spot samples at 36 weeks pregnancy) | N/A | N/A | N/A | (Sugeng et al., 2020) | Study on phthalate exposure from the diet, use of (volatile) household products, household characteristics, and personal care products.  DEHP was measured by MEHP metabolite and detected in 33% of samples (LOD: 4.1 µg/L). geometric mean concentration: 4.0 µg/L (95% CI: 3.8-4.1 µg/L). |
|  |  |  | DEHP | Human, *in vivo* (epidemiology; population-based prospective cohort. N=757-1128 mother-child pairs) | N/A | N/A | No | (Sol et al., 2020) | 2^nd^ trimester (but not 1^st^ or 3^rd^) maternal higher DEHP levels in urine was associated with a 0.18 (95% CI: 0.31-0.06) standard deviation score lower blood glucose concentration among boys aged 9.7 (±0.2) years.  Median maternal urinary phthalate concentrations in nM: 1^st^ trimester: 171.9 in boys, 174.2 in girls 2^nd^ trimester: 96.8 in boys, 89.4 in girls 3^rd^ trimester: 132.3 in boys, 146.8 in girls  Maternal urinary DEHP was not associated with childhood (measured at ~10 years) general or organ fat outcomes. |
|  |  |  | DEHP, MEHP | Systematic review and meta-analysis (**rodent**; n=31 included studies) | N/A | N/A | Uncertain (fat pad, but not body weight increased) | (Wassenaar & Legler, 2017) | Early life exposure to DEHP or MEHP was significantly associated with increased fat (pad) weight (mean difference 0.02, 95% CI: 0.00-0.03). The association with body weight was non-significant (mean difference: -0.14, 95% CI: -0.32-0.04). No meta-analysis could be conducted for other outcomes due to data scarcity (i.e. < 5 studies per outcome). |
|  |  |  |  | Human/ Monkey *in vitro,* HepG2 and COS-1, transiently transfected with reporter construct (300 μM) 24 h | Yes (via PPARα-LBD: 78-fold induction in COS-1) | Yes (via PPARγ-LBD: 39-fold induction in COS-1) | N/A | (Zomer et al., 2000) | Study focusing on PPAR and RXR activation by the branched-chain fatty acids/ phytol derivatives. MEHP was used as a positive control in one experiment. |
| GW7647 | 265129-71-3 | 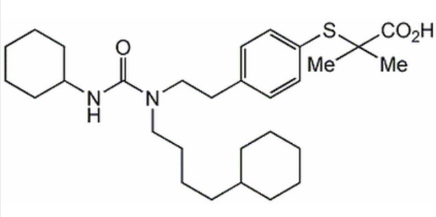 | Pharmaceutical | **SUMMARY** | **Yes, strong selective agonist** | Yes, moderate agonist | N/A, uncertain |  | Selective agonist for PPARα; substantially less potent on PPARγ than on PPARα |
|  |  |  |  | Human *in vitro,* HG5LN reporter cell lines for PPARγ (10^-9^ – 3x10^-6^ M) 24 h | N/A | Yes (EC20: 121 nM, EC50: 459 nM, 106% max activity) | N/A | (Garoche et al., 2021) | Human, mouse, zebrafish, and *Xenopus* PPARγ were tested using known hPPARγ ligands, and environmental chemicals.  HPPARγ and mPPARγ showed similar activities, with marked differences in xPPARγ (less pronounced) and zfPPARγ (more pronounced).  Only data fro hPPARγ were extracted here (activity parameters for other receptors/ species is given in the publication). |
|  |  |  |  | HG5LN reporter cell lines for hPPARα/d/y  (3x10^-10^ – 10^-5^ M)  24 h | Yes (EC50 = 6 ± 1 nM) | Yes (weaker; EC50 = 350 ± 53 nM) | N/A | (Seimandi et al., 2005) | GW7647 served as a potent PPARα agonist, although it also activated PPARd and PPARγ as previously reported at high concentrations. |
|  |  |  |  | Review | Yes (EC50: 6 nM (hPPARα), 1 nM (mPPARα)) | N/A | N/A | (Bougarne et al., 2018) | Fibrates are used in the pharmacological treatment of hypertriglyceridemia (48, 203) despite the low potencies of these first-generation agonists on PPARα. |
|  |  |  |  | Rat *in vitro*, primary rat hepatocytes,  (0.001-10 μM)  2-72 h | Yes | N/A | N/A | (McMullen et al., 2020) | Transcriptional response (ChIP-seq studies of PPARα binding and transcription binding motif identification for PPARα responsive genes) to GW7647 exposure (1 μM, 2 and 24 h).  In vitro results were verified in a limited rat *in vivo* study.  In rats, more down-regulated genes and pathways were identified than the authors had found in the human (McMullen et al., 2014), and PPARα binding motif differed for up-/downregulation. |
|  |  |  |  | Human *in vitro,* primary human hepatocytes,  (0.001-10 μM)  2-71 h | Yes | N/A | N/A | (McMullen et al., 2014) | ChIPseq studies at 2 and 24 h to assess genomic binding of PPARα.  192 genes expressed differentially. 51% of these genes revealed evidence for PPARα binding directly at PPARα response elements or through alternative mechanisms.  Key transcription factors associated with nongenomic targets following GW7647 treatment, including SP1, STAT1, ETS1, ERα, and HNF4α.  More than 80% of genes showing up-regulation, including ACOX1, CPT1, and APOA4. |
| Eicosapentaenoic acid (EPA) | 10417-94-4 | 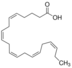 | Nutrient, PUFA essential fatty acid | **SUMMARY** | Yes | Yes | Uncertain (likely no effect) |  | hPPARα/γ positive  Long-chain polyunsaturated fatty acids (LCPUFA) are essential omega 3 (n-3) dietary fatty acid nutrients that regulate numerous cell and organ functions. Intracellular fatty acids or their metabolites regulate transcriptional activation of gene expression during adipocyte differentiation, and retinal and nervous system development. Regulation of gene expression by LCPUFA occurs at the transcriptional level and is mediated by the PPARs and hepatic nuclear factor 4α.  Adequate adult intake of 250 mg/day for eicosapentaenoic acid (EPA) plus DHA is recommended by EFSA 2010. |
|  |  |  |  | Position statement of the Academy of Nutrition and Dietetics and reviews | N/A | N/A | Uncertain/ no association of n=3 PUFA with obesity or lack thereof | (Uauy, Mena, & Rojas, 2000) (Jacobs & Lewis, 2002)  (Vannice & Rasmussen, 2014) | For the healthy adult population, dietary fat should provide 20- 35% of energy, with an increased n-3 polyunsaturated fatty acids consumption and limited saturated and trans-fat intake.  Dietary fatty acid intake recommendation for EPA+DHA: 250-500 mg/day  Alongside other eicosanoids (20C fatty acids), EPA is a prostaglandin precursor, and considered a hormone-like substance because eicosanoids are produced when stimulated, rapidly utilized and metabolised, and not stored in cells. Prostaglandins produced from EPA function as vasodilators and platelet anti-aggregators.  EPA, ARA, and DHA are also involved with gene expression, cytokine activity, cell signalling, and immune modulation.  From NHAMES 2009-2010: Mean daily intake of EPA among men was 40 mg and 30 mg for women. Overall, EPA and DHA seem to possibly (Grade II – “Fair” level of evidence/confidence) have beneficial effects to prevent cognitive decline in adults. Further, there is some evidence for beneficial effects on cardiovascular health, and anti-inflammatory properties, that could ameliorate metabolic disruption symptoms. No specific beneficial effect of DHA or EPA was discussed/ evident from the systematic literature review, though “Fair” level of evidence indicates that conjugated linoleic acid has a beneficial effect on body composition (i.e., relative decrease in fat mass) without effect on body weight. |
| **Docosahexaenoic acid (DHA)** | 6217-54-5 | 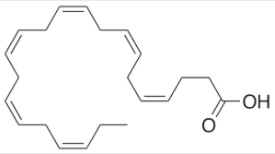 | Nutrient, PUFA essential fatty acid | **SUMMARY** | Strong agonist | Strong agonist | Uncertain. |  | Essential LCPUFA as for EPA. DHA also has significant effects on photoreceptor membranes involved in the signal transduction process, rhodopsin activation, and rod and cone development in the eye. For pregnant and lactating women, an additional 100-200 mg DHA is recommended to that indicated for EPA above . An adequate intake of 100 mg DHA is recommended for older infants (>6 months of age) and young children below the age of 24 months (EFSA, 2010).  DHA cheaper than EPA and 2x more potent on PPARs.  Adipogenesis: some indication (and mechanistic plausibility via PPARγ agonism) for increased adipogenesis/ lipid accumulation in adipocytes. However, concurrent anti-inflammatory properties and contribution to “healthier” lipid status/ lipid metabolism.  Proposed that adipogenesis could be linked to metabolically more active beige/brown adipose tissue, rather than white adipose tissue. No association with (visceral) obesity or on body fat. |
|  |  |  |  |  | Yes, increased in transactivation assay (0.1 mM: ~80%, 10 μM ~ 5% activity of pos. Control)  Decreased expression (n=3 PUFA, Perilla oil) | Yes, increased (transactivation assay) | Yes, increased adipocyte differentiation, but lower visceral adiposity (in rat) | (Uauy, Mena, & Rojas, 2000) | Fatty acids modulate their own metabolism, synthesis, and oxidation (metacrine regulation), and in concert with pancreatic hormones (e.g., insulin).  LCPUFA of both the n-3 and n-6 series reduce hepatic lipogenesis by decreasing the content and activity of enzymes involved in lipid synthesis (FAS, ACC, stearoyl-CoA carboxylase, and malic enzyme). This reduction in lipogenesis is explained by down regulation of gene transcription.  Fatty acids and/or their derived compounds induce the expression of adipocyte-specific gene products (i.e., aP2/FABP4, PEPCK, ACC, LPL) and stimulate adipocyte differentiation.  However, n=3 PUFA supplementation (Perilla oil) significantly reduces the growth of visceral adipose tissue in rats post weaning, despite similar total food consumption (epidymial fat pad weight reduced, and accompanied by PPARα downregulation).  PPARα seems to be activated by both, medium- and long-chain PUFAs.  Negative in Filer 2022 (Filer et al., 2022) predictions for 3T3L1. |
|  |  |  |  | Position statement of the Academy of Nutrition and Dietetics | N/A | N/A | Uncertain/ no association of n=3 PUFA with obesity or lack thereof | (Vannice & Rasmussen, 2014) | For the healthy adult population, dietary fat should provide 20- 35% of energy, with an increased n-3 polyunsaturated fatty acids consumption and limited saturated and trans-fat intake.  EPA, ARA, and DHA are also involved with gene expression, cytokine activity, cell signalling, and immune modulation.  From NHAMES 2009-2010: Mean daily intake of EPA among men was 40 mg and 30 mg for women. Mean daily intake of DHA was 80 mg for men and 60 mg for women.  Overall, EPA and DHA seem to possibly (Grad II – “Fair” level of evidence/confidence) have beneficial effects to prevent cognitive decline in adults. Further, there is some evidence for beneficial effects on cardiovascular health, and anti-inflammatory properties, that could ameliorate MD symptoms. No specific beneficial effect of DHA or EPA was discussed/ evident from the systematic literature review, though “Fair” level of evidence indicates that conjugated linoleic acid has a beneficial effect on body composition (i.e., relative decrease in fat mass) without effect on body weight. |
|  |  |  |  | *In vitro*, 3T3-L1 murine preadipocytes  25-200 μM DHA complexed with BSA (4:1),  1-6 d | N/A | N/A | No/ Decrease | (Kim et al., 2006) | DHA exposures were conducted in the presence of 0.2 mM α-tocopherol to prevent lipid peroxidation. BSA only was used as a control.  DHA had no effect on pre-confluent adipocyte proliferation, but inhibited differentiation-associated mitotic clonal expansion of post-confluent adipocytes at all test concentrations (p<0.01). DHA induced apoptosis in post confluent preadipocytes at 100 μM (48 h exposure) and 200 μM (24 and 48 h).  After 6 d exposure, DHA decreased mean droplet size and lipid area in differentiating adipocytes in a concentration-dependent manner, alongside decreased GPDH activity (p<0.01). In fully differentiated adipocytes, DHA increased basal lipolysis compared with the control (p<0.01). These results demonstrate that DHA may exert its antiobesity effect by inhibiting differentiation to adipocytes, inducing apoptosis in post confluent preadipocytes and promoting lipolysis. |
|  |  |  |  | Mouse, *in vivo* (obese aged (6 months) female mice,  DHA-enriched HFD)  18 m | N/A | N/A | Uncertain (amelioration of inflammation, and shift from WAT to beige adipocytes) | (Félix-Soriano et al., 2023) | Subcutaneous white adipose tissue (scWAT) was investigated.  mean adipocyte size (statistically significant, but not visually evident vs. HFD-only) and reversed the upregulation of lipogenic genes induced by the HFD were reduced by DHA-enriched diet by reaching values even less than in non-HFD control animals.  DHA supplementation led a metabolic remodelling of scWAT to a healthier phenotype by regulating genes having control over adipocyte lipid accumulation, **reducing inflammatory status, and inducing beige adipocyte markers** in aged obese mice.  No significant reduction of body weight, fat mass, scWAT mass, or visceral WAT mass was observed by DHA (compared to HFD only); all these markers were significantly increased vs. Non-HFD control. |
|  |  |  |  | Human, *in vivo* (clinical study, n=36 adults, 2 g/d algal DHA or placebo, 4.5 months) | N/A | N/A | Uncertain/ no effect on body weight | (Neff et al., 2011) | Randomised, controlled double-blind trial; 36 overweight or obese adults. Effects on plasma lipid and lipoprotein and other biomarkers in absence of weight loss were investigated.  Significant changes (p<0.001): VLDL particle size decreased, mean LDL and HDL particle size increased. Concentration significantly altered (p<0.01): large LDL and HDL particles increased, small LDL and medium HDL particles decreased.  Anti-inflammatory plasma IL-10 increased, but no effect change to glucose metabolism, insulin sensitivity, blood pressure, or other inflammatory markers. |
| Chlorpyrifos (CPF) | 2921-88-2 | 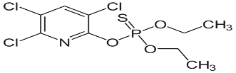 | Organophosphate pesticide | **SUMMARY** | No | No/ weak | No |  | Some animal studies showed an association between CPF and obesity as well as metabolic disruption. CPF oxon is the major *in vivo* metabolite – bioactive in serine hydrolase inhibition. The *in vitro* evidence is limited. One study showed 0.1, 1, and 10 μM CPF inhibited the osteogenic differentiation capacity of human MSCs, although the potential of MSCs to differentiate into adipocytes was not tested. Inducer, from Filer 2022 prediction (probably weakly active; score: 0.07) (Filer et al., 2022). EFSA Statement on human health assessment: <https://www.efsa.europa.eu/en/efsajournal/pub/5908>  December 2019: EU approvals for this and CPF-methyl not renewed due to likely genotoxicity and developmental neurotoxicity, therefore lower priority in terms of prospective use in the EU, as will be banned also on incoming produce (EFSA, 2019). |
|  |  |  |  | *In vitro*, 3T3-L1 murine cell line  CPF (0.3 μM, 1 μM, 3 μM, 10 μM, 30 μM, 100 μM for NR activation and 20 μM, 50 μM, 75 μM for lipid accumulation)  6 d | No/ potentially antagonism | No | No. Significant reduction in lipid accumulation at 50 and 75 μM | (Taxvig et al., 2012) | Four chemicals (BPA, MEHP, butylparaben, PCB 153) out of the eleven revealed elevation in adipogenesis.  Nuclear receptor activation was assessed in transient transfection assay with mouse PPARα and mouse PPARγ (i.e., not human receptors).  Significant decrease in mPPARα-mediated luciferase signal was observed upon exposure to 10 and 30 μM CPF (but not at 3 or 100 μM). |
|  |  |  |  | Human, *in vivo* (epidemiology, longitudinal CHAMACOS cohort, n=488 pregnant women) | N/A | N/A | No (no adverse association between maternal exposure and growth outcomes at birth) | (Eskenazi et al., 2004) | Cohort: longitudinal birth cohort study of the effects of pesticides and other environmental exposures on the health of pregnant women and their children living in the Salinas Valley (low-income population, often farm workers). < 20 weeks gestation at enrolment; 2 maternal urine samples during pregnancy (one during first half, second in second half); infant birth weight, crown–heel length, and head circumference were measured during delivery.  There is no adverse association between fetal growth and any measure of in utero organophosphate pesticide exposure.  On the contrary, some organophosphate pesticides were associated with increased body length and head circumference.  For umbilical cord cholinesterase activity, exposure in latter part of pregnancy seemed to have a stronger effect. |
|  |  |  |  | Human, *in vitro*, primary human bone marrow mesenchymal stem cells,  (0.1-10 μM) 2-21 d | N/A | N/A | No (Oil Red O staining, visual observation) | (Hoogduijn, Rakonczay, & Genever, 2006) | Cells from 3 different donors (sex not specified) were used for differentiation assays before passage 5. For adipogenic differentiation, cells were cultured for 14 d with MDI induction cocktail.  Micromolar concentrations of these anticholinergic insecticides (CPF or carbofuran) had no effect on MSC survival (MTT assay, up to 3 weeks), proliferation, or morphology (up to 4 weeks exposure), but limited MSC differentiation capacity by inhibiting osteogenic differentiation. |
|  |  |  |  | Review | N/A | N/A | Uncertain | (Li et al., 2019) | Focus of review was on CPF altering levels of reproductive hormones.  Some studies reporting altered body weight (conflicting data from rodent studies; reports of both increased and decreased body weight, or relative fat pad weight) upon exposure are listed. Further, reports on circulating lipids/lipid metabolites in rodent studies are presented, with a trend for increased triglyceride levels.  With respect to reproductive hormone levels, particularly testosterone seems to be consistently decreased (in the studies included), leading the authors to suggest a possible risk of metabolic syndrome development due to testosterone deficiency. |
|  |  |  |  | Review, weight-of-evidence evidence evaluation | N/A | N/A | N/A, but unlikely (no indidcation for adipogenesis/ obesity | (Prueitt et al., 2011) | Hypothesis-based weight-of-evidence evaluation of CPF neurodevelopmental effects.  Epidemiological and animal data not consistent to show associations with neurodevelopmental adverse outcomes at doses <acetylcholine esterase inhibition.  Epidemiological evidence of neurodevelopmental outcomes is most likely due to mechanisms other than acetylcholine esterase inhibition. |
|  |  |  |  | Human, *in vivo* (epidemiology, n=90 families, SUPERB study) | N/A | N/A | N/A | (Trunnelle et al., 2014) | Samples collected between 2007-2009, northern California, focusing on residential use of pyrethroid insecticides and CPF.  Urinary metabolite detected for CPF was TCPy (64.7% of samples, median concentration 1.47 ng/mL; < NHANES 2001-2002 representative study). No conclusions on adverse outcomes in the population were drawn. |
|  |  |  |  | Review of human health hazards for regulatory purposes | N/A | N/A | Not identified/ no | (EFSA, 2019) | Peer review of mammalian toxicity and human health by EFSA requested by the European Commission.  Based on this report, market approval in the EU was withdrawn in December 2019 (adopted by the European commission in January 2020).  Main toxicological concerns were the unclear genotoxic potential and neurodevelopmental effects. The latter was observed in rats *in vivo* and supported by epidemiological evidence related to developmental neurological outcomes in children and is most critical for non-renewal of market approval of CPF (and the structurally similar CPF-methyl) as the active ingredient of plant protection products.  There is no indication of increased body weight/adipogenesis. |
|  |  |  |  | Review; intergovernmental organization | N/A | N/A | Not identified/ no | (FAO, 2006; WHO, 2009) | There is no evidence suggesting adipogenesis. |
|  |  |  |  | Review for regulatory purposes (toxicological hazard for evaluation of chemicals listed under the Stockholm convention on POPs) | N/A | N/A | Not identified/ no | (Persistent Organic Pollutants Review Committee (POPRC), 2022) | Adverse effects on human health are mainly related to neurological/neurodevelopmental effects: exposure to CPF during pregnancy was associated with adverse neurodevelopmental outcomes in children, including changes in brain morphology and behavioural symptoms (in a matched mother-child pair epidemiological cohort). Acute poisoning events were dominated by neurotoxic effects and summarized as the cholinergic toxidrome with inhibition of (acetyl)cholinesterase as a central mechanism.  There is no evidence suggesting adipogenesis/lipid accumulation. |
| **Perfluorohexanoic acid (PFHXA)** | 307-24-4 | 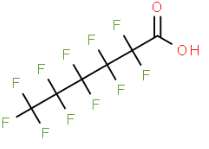 |  | **SUMMARY** | No | No | Uncertain/ tentative negative) |  | Pronounced reproductive toxicity  ECHA restriction proposal 25 March 2020.  Tentative negative for adipogenesis, despite association with altered blood/ serum lipid composition. |
|  |  |  |  | Human in vitro, HG5LN reporter cell lines for PPARγ | N/A | No  (For EC20, EC50 and max activity- nonactive) | N/A | (Garoche et al., 2021) | Human, mouse, zebrafish, and *Xenopus* PPARγ were tested using known hPPARγ ligands, and environmental chemicals.  HPPARγ and mPPARγ showed similar activities, with marked differences in xPPARγ (less pronounced) and zfPPARγ (more pronounced).  Only data fro hPPARγ were extracted here (activity parameters for other receptors/ species is given in the publication, though not for PPARα). |
|  |  |  |  | Human *in vivo* epidemiology (n = 222 pregnant women) | N/A | N/A | N/A (strong linear trend for elevated triglycerides) | (Spratlen et al., 2020) | World Trade Center birth cohort; association between 5 PFAS (PFOA, PFOA, PFHxS, PFNA, PFDS) in cord blood and cord blood lipids (total lipids, total cholesterol, triglycerides) was studied. |
| **Phytanic acid** | 14721-66-5 | 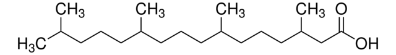 | Dietary lipid | **SUMMARY** | Yes, weak agonist 10-4 µM | Yes, weak agonist | Uncertain/ possibly via RXR |  | Available data suggest that, at physiological concentrations, phytanic acid is a natural RXR agonist. It is more likely that **the metabolite pristanic acid is responsible for the PPARα agonist effect rather than phytanic acid itself**.  Phytanic acid, but not pristanic acid, mediates the positive effects of phytol derivatives on **brown adipocyte differentiation** (Schluter et al., 2002). |
|  |  |  |  | Review | Yes | N/A | N/A | (Jacobs & Lewis, 2002) | Phytanic acid reviewed as natural physiological ligand for PPAR, also references therein |
|  |  |  |  | Review | Yes, potentially (agonist at 20 and 100 μM; Kd = 34 nM) | N/A | Uncertain (positive at 40 μM in mouse preadipocytes in one study, but negative in mouse embryo fibroblasts at 50 μM in another study. In vivo some indication of beneficial effects on bodyweight and WAT mass) | (Hellgren, 2010) | RXR (phytanic acid) and PPARα (possibly via metabolite pristanic acid) agonist.  Anecdotal evidence that fatty acid oxidation is increased in animals after phytol ingestion, but it is at present not possible to deduce whether phytanic acid is useful in the prevention of ectopic lipid deposition.  Inducts UCP1 expression (in human skeletal muscle).  Higher levels of serum phytanic acid possibly associated with increased risk of prostate cancer (causal link not supported yet). UCP1 is a marker of **brown adipocytes**.  Concentration of phytanic acid in serum from 250 healthy humans ranged from 0.04 to 11.5 μM, the median value was 1.6 μM.  Phytanic acid was defined as a high-affinity ligand, with a Kd of 34 nM for the free fatty acid and 11 nM for the CoA ester.  Phytanic acid was able to induce differentiation to white adipocytes from both 3T3-L1 cells and primary cultures of human preadipocytes. This was likely mediated via RXR. However, in mouse embryo fibroblasts (C3H10T1/2), adipocyte differentiation was very low upon exposure to 50 μM phytanic acid.  Some indication for loss of body weight in patients with Refsum’s disease, alongside decrease in WAT and absence of illness. |
|  |  |  |  | Mouse, *in vivo* and *in vitro,* brown preadipocytes isolated for primary culture | N/A | N/A | Yes, into brown adipocytes (1 μM; non-toxic up to 40 μM) | (Schluter et al., 2002) | 8-week-old Swiss mice on standard diet, containing 0.6545 ug phytanic acid per g feed.  1 μM induced significant increase in cells with adipocyte morphology (~25% of cells), accompanied by a significant increase in aP2 gene expression (3.1 ± 0.4-fold increase).  Differentiation UCP1 expression was mediated via RXR, not via PPARα. |
|  |  |  |  | Beef cattle, *in vitro,* subcutaneous primary WAT preadipocytes, 25-100 μM,  8-10 d (until 80% of cells were differentiated by 20 μM ROSI) | N/A | Yes (LOEC 50 μM) | Yes | (García-Rojas et al., 2010) | Subcutaneous adipose tissue was sampled from beef cattle at slaughter (n=30 animals for ROSI positive control, n=5 animals for all other chemicals), and dissociated, to obtain primary preadipocytes.  Preadipocyte differentiation after reaching confluence was induced by various treatments: ROSI (20 μM); unsaturated fatty acids: phytanic acid (25, 50, 100 μM) and pristanic acid (25, 50, 100 μM); retinoids: 9-cis retinoic acid (0.5, 0.75, 1 μM) and all-trans retinoic acid (0.5, 0.75, 1 μM); and carotenoids: β-carotene (10, 20, 30 μM) and lutein (10, 20, 30 μM).  In differentiated cells, PPARγ and PGC-1a gene expression was measured. Phytanic acid, all-trans retinoic acid, and 9-cis retinoic acid were the best activators of PPARγ expression, and the combination of 9-cis and all-trans retinoic acid was the best activator of PGC-1α expression (P < 0.05). |
|  |  |  |  | Human and Monkey *in vitro,*  HepG2 and COS-1, transiently transfected with reporter construct,  respectively  (50 μM and 12.5-100 μM)  24 h | Yes (via PPRE: induction 3-fold in HepG2, 7.2-fold in COS-1. Via PPARα-LBD: 9.0-fold induction in COS-1) | No/ negligible (2.5-fold) | N/A (potentially, via RXR) | (Zomer et al., 2000) | Both phytanic acid and pristanic acid activate PPARα in a concentration-dependent manner. Activation is observed via the ligand binding domain of PPARα as well as via a PPRE.  Phytanic acid also trans-activates all three RXR subtypes in a concentration-dependent manner.  **Phytanic acid itself, and not only its metabolite, pristanic acid, is a true physiological ligand for PPARα** (demonstrated in phytanic acid α-oxidation-deficient primary human fibroblasts).  Pristanic acid yielded higher induction rate (~10-times higher) via the PPARα ligand binding domain, but this was not observed with full-length PPARα both with and without RXRβ co-transfection: here, both fatty acids yielded similar induction rates (~50-fold PPARα alone; up to ~125-fold in co-transfected cells). |
|  |  |  |  | Human *in vivo,* (epidemiology; n=14 young adults, 4 weeks, 45 g milk fat/d with 0.24 or 0.13% phytanic acid via diet) | N/A | N/A | N/A (metabolic syndrome risk marker not altered upon intervention) | (Werner et al., 2011) | Double-blind, randomised experimental intervention study. Study participants aged 20-42 years, healthy, with BMI < 30.  Plasma phytanic acid increased in both groups with the highest increase in control group (24%) compared to phytanic acid group (15%). There were no significant effects of phytanic acid on risk markers for metabolic syndrome. |
|  |  |  |  | Transient transfection of human RXR in CHO K1 cells | N/A | N/A | N/A | (Kitareewan et al., 1996) | RXR activator at 4-64 μM; phytanic acid displaced 9cis retinoic acid from RXR with Ki=4 μM. |
|  |  |  |  | Human and mouse, *in vitro*, primary human and 3T3-L1 murine pre-adipocytes, respectively  (40-80 M)  1-2 w | Not likely | N/A | Uncertain/ yes, likely via RXR especially in presence of other PPARγ activators | (Schlüter et al., 2002) | Phytanic acid induced the adipocyte differentiation of 3T3-L1 cells as assessed by accumulation of lipid droplets and induction of the aP2 mRNA marker. This effect was mimicked by a synthetic activator of RXR (1 μM AGN19420) but not by a PPARα agonist (Wy14643) or by palmitic acid (80 μM).  Pre-adipocytes were exposed to fatty acids in non-differentiating medium after induction.  After 1 week (2 weeks), in 3T3-L1 cells: 40 μM phytanic acid yielded ~50% (70%) adipocytes in culture, 80 μM ~ 65% (>85%) adipocytes.  **In human WAT pre-adipocytes, differentiation into adipocytes was suppressed** (to ~65%) **without addition of a synthetic PPARγ activator** (BRL49653). |
| **Phytanic acid metabolite: Pristanic acid** | 1189-37-3 | 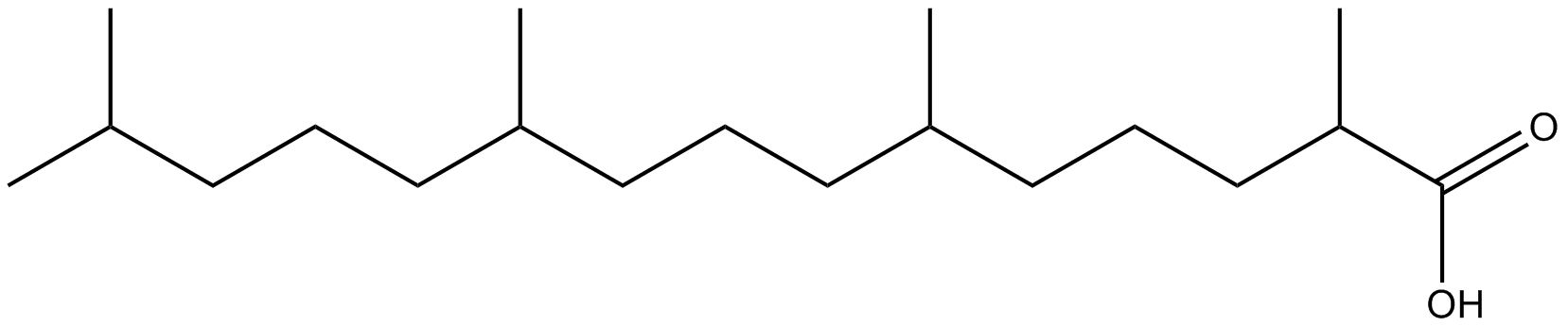 | Dietary lipid | **SUMMARY** | Yes, strong agonist | Yes, weak-moderate agonist | Uncertain |  | Metabolite of phytanic acid (see notes above). |
|  |  |  |  | Review | Yes |  |  | (Jacobs & Lewis, 2002) | Pristanic acid reviewed as natural physiological metabolite ligand for PPAR, also references therein |
|  |  |  |  | Mouse, *in vivo* and *in vitro* | N/A | N/A | No (1 μM; 10 μM toxic) | (Schluter et al., 2002) | 8-week-old Swiss mice on standard diet, containing 0.6545 ug phytanic acid per g feed.  Pristanic acid was more toxic to primary brown preadipocyte cultures than phytanic acid.  No signs of adipocyte differentiation were observed in pristanic acid-treated cells and aP2 mRNA levels were indistinguishable from non-treated cells (0.9 ± 0.3-fold induction) |
|  |  |  |  | Beef cattle, *in vitro*, subcutaneous primary WAT preadipocytes  (25-100 μM)  8-10 d  (until 80% of cells were differentiated by 20 μM ROSI) | N/A | Yes (LOEC 100 μM; reduced at 25 μM)) | Yes | (García-Rojas et al., 2010) | Subcutaneous adipose tissue was sampled from beef cattle at slaugther (n=30 animals for ROSI positive control, n=5 animals for all other chemicals), and dissociated to obtain primary preadipocytes.  Preadipocyte differentiation after reaching confluence was induced by various treatments: ROSI (20 μM); unsaturated fatty acids: phytanic acid (25, 50, 100 μM) and pristanic acid (25, 50, 100 μM); retinoids: 9-cis retinoic acid (0.5, 0.75, 1 μM) and all-trans retinoic acid (0.5, 0.75, 1 μM); and carotenoids: β-carotene (10, 20, 30 μM) and lutein (10, 20, 30 μM).  In differentiated cells, PPARγ and PGC-1a gene expression was measured. Phytanic acid, all-trans retinoic acid, and 9-cis retinoic acid were the best activators of PPARγ expression, and the combination of 9-cis and all-trans retinoic acid was the best activator of PGC-1α expression (P < 0.05). |
|  |  |  |  | Human/ Monkey *in vitro,* (HepG2 and COS-1, transiently transfected with reporter construct 50 μM (and 12.5-200 μM)  24 h | Yes (via PPRE: induction 34.2-fold in HepG2, 109.4-fold in COS-1. Via PPARα-LBD: 120.3-fold induction in COS-1) | No/ negligible (3.7-fold) | N/A (not likely; not an RXRb ligand) | (Zomer et al., 2000) | Both phytanic acid and pristanic acid activate PPARα concentration-dependent.  Activation is observed through a PPRE as well as ligand binding domain of PPARα.  Phytanic acid trans-activates all three RXR subtypes concentration dependent.  **Phytanic acid itself, and not only its metabolite, pristanic acid, is a true physiological ligand for PPARα** (demonstrated in phytanic acid α-oxidation-deficient primary human fibroblasts).  Via ligand binding domain, pristanic acid yielded higher induction rated (~10-times higher), but this was not observed with full-length PPARα with-without RXRb co-transfection: here, both fatty acids yielded similar induction rates (~50-fold PPARα alone; up to ~125-fold in co-transfected cells). |
|  |  |  |  | Review | Yes, likely (more potent than parent chemical: phytanic acid) | N/A | N/A | (Hellgren, 2010) | RXR (phytanic acid) and PPARα (possibly via metabolite pristanic acid) agonist.  Anecdotal evidence that fatty acid oxidation is increased in animals after phytol ingestion, but it is at present not possible to deduce whether phytanic acid is useful in the prevention of ectopic lipid deposition.  Induces UCP1 expression (in human skeletal muscle).  Higher levels of serum phytanic acid possibly associated with increased risk of prostate cancer (causal link not supported yet).  Median plasma level of pristanic acid was reported to be 0.5 μM in a healthy population, with individual variation between 0 and 4.3 μM. |
| 15-Deoxy-Δ12,14-prostaglandin J2 (15d-PGJ2) | 87893-55-8 | 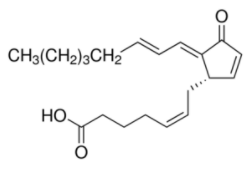 | Metabolite of endogenous prostaglandin (PGJ2) | **SUMMARY** | Uncertain/ weak | Yes, strong endogenous agonist | Uncertain/ potentially “Yes” |  | Endogenous PPARy agonist and ligand. |
|  |  |  |  | Review | Yes (but less potent than on PPARy) | Yes | N/A | (Uauy, Mena, & Rojas, 2000) | Direct binding to PPARy has been demonstrated. PGJ2-metabolites are highly specific PPARy activators.  PGJ-series activates all PPAR-isoforms, including PPARy. |
|  |  |  |  | Reporter gene constructs in CV-1 cells. (1, 3, and) 10 μM | Yes, but limited (up to ~4-fold induction) | Yes (>13-fold activation) | Yes (5 μM 15d-PGJ2; in 3T3-L1 murine preadipocytes) | (Forman et al., 1995) | 15d-PGJ2 was the most potent metabolite of arachidonic acid to activate PPRE. Adipocyte differentiation and lipid accumulation upon exposure of 3T3-L1 murine preadipocytes to 5 μM 15d-PGJ2 was accompanied by substantial expression of aP2 and adipsin. |
|  |  |  |  | Human or mouse *in vitro,*  (3T3-L1 murine preadipocytes or OP9 mBMSCs) 10 d  Primary human subcutaneous preadipocytes  14 d |  | Yes | Uncertain. In 3T3-L1 (with and without dexamethasone) and in OP9 increased lipid accummulation, but not statistically significant | (Kim et al., 2021) | Endpoints: lipid accumulation, RNA-Seq and confirmation of gene expression by RT-qPCR  Data-driven hierarchical clustering approach to identify PPARγ modulating chemicals, and transcriptional changes related to differentiation into white or brite/beige adipose tissue.  The authors compared a strong PPARγ therapeutic agonist that also was shown to modify PPARγ phosphorylation (i.e., ROSI, a chemical that was shown to modify only PPARγ phosphorylation (i.e., roscovitine), a weak PPARγ agonist and endogenous molecule (i.e., 15dPGJ2), and two known environmental PPARγ ligands [i.e., TBBPA and TPhP].  Important genes for predicting PPARγ ligand/modification status, specifically the down-regulation of *Rpl13* and the upregulation of *Cidec*.  Max. Tested concentration: 1x10^-6^ M  Max. Non-toxic concentration: 1x10^-6^ M |
| **Tesaglitazar/AZ242** | 251565-85-2 | 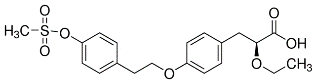 | Pharmaceutical | **SUMMARY** | Yes | Yes | Uncertain/ No |  | Clinical development discontinued in Phase III trials due to cardiac toxicity (related to reduced recruitment of PPAR co-activators PGC-1a and SIRT1).  Tesaglitazar improved insulin resistance. No clear and consistent effect on adipogenesis/ obesity was reported in different studies; studies were usually conducted with the aim to ameliorate symptoms of Type II Diabetes in subjects with/without obesity. There is no substantive evidence to suggest Tesaglitazar induces adipogenesis/ obesity. |
|  |  |  |  | In silico (hPPARα, mPPARγ) | Yes | Yes (more potent than on PPARα) | N/A | (Cronet et al., 2001) | Crystal structure of apo PPARα and apo PPARγ and in complex with the synthetic dual agonist tesaglitazar.  Tesaglitazar displaced a radioactive probe from the receptors (IC50 = 1.0 μM PPARα, 0.2 μM PPARγ). Recruitment of PPAR co-activator SRC-1: EC50 = 1.2 μM (PPARα) and 1.3 μM (PPARγ). |
|  |  |  |  | Mouse *in vivo* (wt and leptin receptor-deficient *db/db* C57BL/6 mice) | Yes | Yes | N/A | (Kalliora et al., 2019) | Evidence of adversity: cardiac dysfunction.  Cardiac dysfunction was observed in animals independent on diet (chow vs. High-fat) and despite lower plasma triglycerides and glucose levels.  Cardiac PGC-1a expression was reduced and/or showed increased acetylation, alongside decreased SIRT1 expression and overall lower mitochondrial abundance. |
|  |  |  |  | Human or mouse *in vitro,*  (3T3-L1 murine preadipocytes or OP9 mBMSCs)  10 d  Primary human subcutaneous preadipocytes  14 d |  | Yes | Yes. In OP9 and 3T3-L1 (with and without dexamethasone) more potent than ROSI | (Kim et al., 2021) | Endpoint: Lipid accumulation, using RNA-Seq and confirmation of gene expression by RT-qPCR.  Data-driven hierarchical clustering approach to identify PPARγ modulating chemicals, and transcriptional changes related to differentiation into white or brite/beige adipose tissue.  The authors compared a strong PPARγ therapeutic agonist that also was shown to modify PPARγ phosphorylation (i.e., ROSI, a chemical that was shown to modify only PPARγ phosphorylation (i.e., roscovitine), a weak PPARγ agonist and endogenous substance (i.e., 15dPGJ2), and two known environmental contaminant PPARγ ligands [i.e., TBBPA and TPP].  Important genes for predicting PPARγ ligand/modification status, specifically the down-regulation of *Rpl13* and the upregulation of *Cidec*.  Max. Tested concentration: 5x10^-6^ M,  max. Non-toxic concentration: 5x10^-6^ M |
| Clofibrate | 637-07-0 | 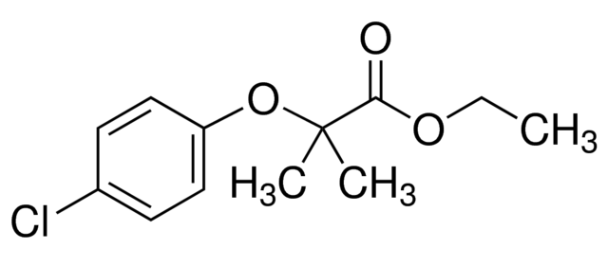 | Pharmaceutical, fibrate, pro-drug | **SUMMARY** | Yes, strong | Yes | Uncertain, likely no effect |  | Widely used for lowering triglyceride levels with PPARα agonism as the mode of action. However, the active metabolite is clofibric acid. |
|  |  |  |  | Review | Yes (EC50: 55 μM (hPPARα), 50 μM (mPPARα)) | N/A | N/A | (Bougarne et al., 2018) | Fibrates are used in the pharmacological treatment of hypertriglyceridemia despite the low potencies of these first-generation agonists on PPARα. |
|  |  |  |  | Human, *in vivo* (WHO review) | N/A (but identified as mode of action) | N/A | N/A (presumably no – no indication of increased bodyweight) | (Oliver, 2012; Report of the Committee of Principal Investigators, 1984) | Mean observation time: 13.2 years (5.3 years in clinical trial, 7.9 years follow-up period). N=15,745 men aged 30-59 years.  Overall, 1788 deaths were recorded in 208,000 man-years included in this report; 70 (11%) more deaths in clofibrate group compared to control – mainly during treatment period (fewer excess deaths in follow-up: only 5%). Excess deaths were due to a large variety of causes and not just due to ischaemic heart disease.  Overall, the results were contradictory: reduction in nonfatal myocardial infarction, less hypertension, no change in coronary mortality but an increase in non-cardiovascular mortality. Further, cholesterol gall stone formation was increased with clofibrate. |
|  |  |  |  | Human, *in vivo,* (clinical intervention; 3 years daily administration) | N/A | N/A | N/A (likely negative or decrease) | (Grundy et al., 1972) | Influence of clofibrate on cholesterol metabolism was studied in 24 patients with hyperlipidaemia + 5 patients with hypercholesterolaemia.  Except in fat-induced hypertriglyceridemia (two patients), clofibrate caused reduced plasma levels of triglycerides and cholesterol in all categories of hyperlipidemia. Clofibrate is believed to increase output of cholesterol while simultaneously inhibiting any compensatory increase in cholesterol synthesis. Effects on atherogenesis were not studied. |
|  |  |  |  | Review of fibrates’ mode of action | Yes | N/A | N/A (likely negative or decrease) | (Staels et al., 1998) | Treatment with fibrates results in a substantial decrease in plasma triglycerides and is usually associated with a moderate decrease in LDL cholesterol and an increase in HDL cholesterol.  In summary, both enhanced catabolism of triglyceride-rich particles and reduced secretion of VLDL underlie the hypo triglyceridemic effect of fibrates, whereas their effect on HDL metabolism is associated with changes in HDL apolipoprotein expression (increased levels of apoa-I and apoA-II proteins, under PPARα transcriptional control). |
| **Clofibrate metabolite:**  **Clofibric acid (CA)** | 882-09-7 | 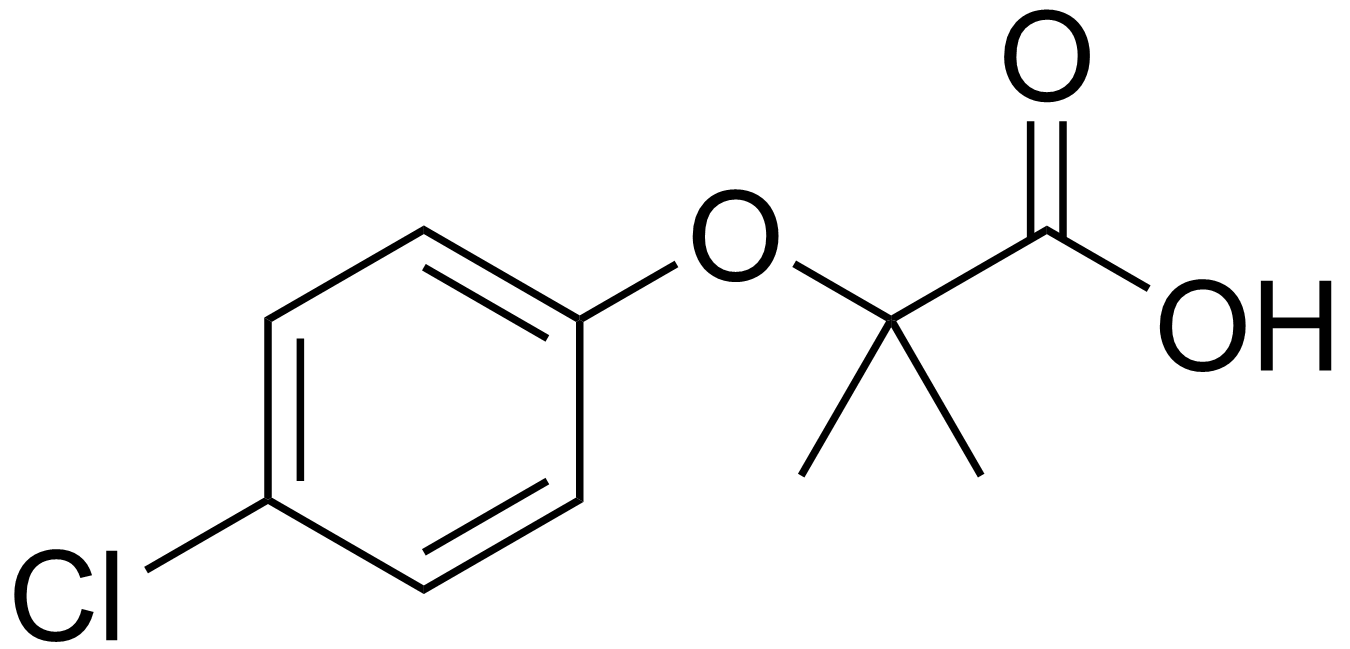 | Pharmaceutical; active metabolite of clofibrate | **SUMMARY** | Yes, moderate agonist  (EC50 = 50.0 µM) | Yes, moderate agonist | Uncertain/ N/A |  | Clofibrate active metabolite; more active than clofibrate, therefore preferred. Slightly less potent in PPARγ transactvation compared to PPARα. |
|  |  |  |  | HG5LN reporter cell lines for PPARγ | N/A | Yes (EC20: 5.1 μM, EC50: n.d, 39% max activity) | N/A | (Garoche et al., 2021) | Human, mouse, zebrafish, and *Xenopus* PPARγ were tested using known hPPARγ ligands, and environmental chemicals.  HPPARγ and mPPARγ showed similar activities, with marked differences in xPPARγ (less pronounced) and zfPPARγ (more pronounced).  Only data from hPPARγ were extracted here (activity parameters for other receptors/ species is given in the publication). |
|  |  |  |  | Mouse *in vivo* (8-week-old female BALB/c *nu/nu* mice, OVCAR-3 and DISS cells are used for creating xenograft mouse models)  Human *in vitro*, OVCAR-3 and DISS cells  (0, 0.5, 5, 50, or 500 μmol/L)  48 h for CR expression  72 h for cell growth assessment | Yes | N/A | N/A | (Yokoyama et al., 2007) | Cell lines derived from human ovarian cancer.  Treatment with CA suppressed tumour cell growth *in vitro* (dose-dependent inhibition of cell proliferation) and upon subcutaneous xenotransplantation (mice).  CA has an antitumor effect on cancer bearing mouse model created by using OVCAR-3 cells. This effect was determined with the reduction of tumour volume and also determined with cisplatin (positive control). in the CA group, significantly induced apoptosis and reduced number of micro vessels were observed compared to control and cisplatin groups.  Tumour mPGE and VEGF expressions, PGE2 concentrations in serum and ascites were significantly decreased in the CA group compared to cisplatin and control groups.  In cancerous peritonitis model created by using DISS cells, duration of survival increased in both CA and cisplatin groups compared to control group.  Under in vitro conditions CR expression (enzyme converting PGE2 into PGF2α) was investigated and its relationship with PGE2 decrease was evaluated by Western-blot. Increased CR, PPARα and BE expressions increased dose dependently expression was observed following CA exposure.  CA dose dependently inhibited cell growth in both cell lines.  CR might be directly involved in decreasing PGE2 level and lowering VEGF expression. |
| (aR)-4-chloro-a-[3-(trifluoromethyl) phenoxy]benzeneacetic acid, (MBX-102/JNJ39659100)  Arhalofenate  MBX-102 | 24136-23-0 | 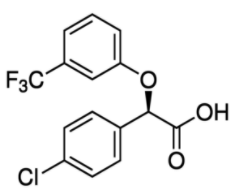 |  |  | Not active | Weak agonist | ? No |  | Experimental chemical.  Not a priority, but mechanistically interesting. |
|  |  |  |  |  | No (in rats *in vivo* and primary human hepatocytes) | Yes (weak selective agonist; ~5-fold induction at 100 μM) | No (no weight gain, no change in WAT weight) | (Chandalia et al., 2009) | Rodent *in vitro* (mouse PPAR reporter gene assays; transient transfection in HEK293T; 20-24 h exposure; 0.4-200 μM), *in vivo* (Zucker Diabetic Fatty rat (11 d, 100 mg/kg bw/d oral gavage) and Zucker Fatty rat (32 d, 100 mg/kg bw/d oral gavage)), and human *in vitro* (primary human hepatocytes; 24 h).  Selective, partial PPARγ agonist that lowers glucose. Triglyceride lowering effect of MBX-102 is PPARα independent.  PPARγ response induced by 100 μM MX-102 is only ~15% or max. induction by ROSI.  In rats, MX-102 significantly lowered plasma glucose, triglyceride, free fatty acid, and cholesterol levels from as early as 2 days of treatment. Reductions were stronger than with 4 mg/kg bw/d ROSI. |
| 1alpha, 25-Hydroxyvitamin D3 (calcitriol, OHVitD3) | 32222-06-3 | 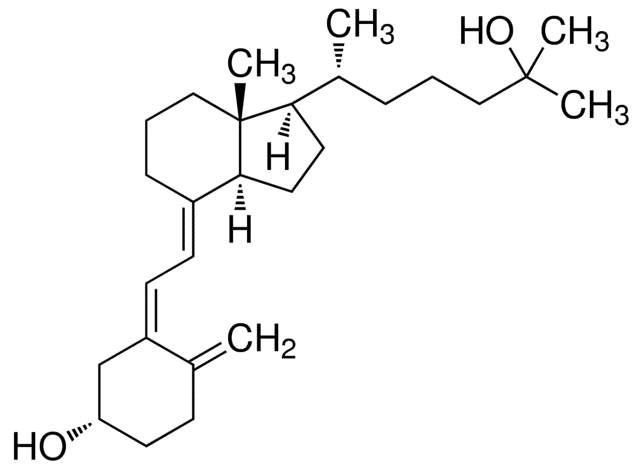 | Nutrient/ essential vitamin/ hormone metabolite | **SUMMARY** | Uncertain/ potentially weak agonist | Uncertain | No effect |  | Vitamin D3 active metabolite, endogenous VDR ligand, potential crosstalk with RXR.  Adipose tissue is a major storage compartment for Vit D and its (active) metabolite(s).  Inhibits intracellular fat accumulation.  Part of signalling pathway in adipose/ osteoblast development.  PPARγ: potentially interspecies differences: inactive in mice/rodent, (weakly?) active on hPPARγ.  Obesity is associated with low Vitamin D3 levels. Supplementation has no effect on increasing obesity/ body weight/ fat mass/ (human *in vivo*) adipogenesis to the contrary, it has therapeutic benefits by reducing body fat mass. |
|  |  |  |  | Human *in vitro*,  LNCaP cells (100 nM)  48 h | Yes | N/A | N/A (increased lipid accumulation in prostate cancer cells) | (Wang, Welsh, & Tenniswood, 2013) | Testosterone (5 nM) and OHVitD3 induced up-regulation of the PPARα mRNA and its downstream targets, leading to increased lipogenesis (in prostate cancer cells).  Endogenous vitamin D receptor ligand. |
|  |  |  |  | Human *in vivo* (clinical trial, 25 ug VitD/day vs. Placebo, 12 w) | N/A | N/A | No/ uncertain (body fat mass reduced, but no effect on body weight or waist circumference) | (Salehpour et al., 2012) | Double-blind, randomized, placebo-controlled, parallel-group trial. N=77 healthy overweight and obese women (age 38±8.1 years, BMI 29.8±4.1 kg/m^2^).  Serum 25(OH)D significantly increased in the vitamin D group compared to the placebo group (38.2±32.7 nmol/L vs. 4.6±14.8 nmol/L; P<0.001) and serum iPTH concentrations were decreased by vitamin D3 supplementation (-0.26±0.57 pmol/L vs. 0.27±0.56 pmol/L; P<0.001). Supplementation with vitamin D3 caused a statistically significant decrease in body fat mass in the vitamin D group compared to the placebo group (-2.7±2.1 kg vs. -0.47±2.1 kg; P<0.001). However, body weight and waist circumference did not change significantly in both groups. A significant reverse correlation between changes in serum 25(OH) D concentrations and body fat mass was observed (r = -0.319, P = 0.005).  It is, however known, that reduction in body weight and/or fat mass increases circulating VitD levels and that overweight/ obesity itself is a risk factor for hypovitaminosis D. |
|  |  |  |  | Systematic review and metaregression analysis | N/A | N/A | N/A, not likely (weight loss increases serum OHVitD3) | (Pannu, Zhao, & Soares, 2016) | Aim of the review was to confirm a volumetric dilution of vitamin D in obesity.  N=18 of 23 human trials were included in the analysis.  Meta regression analyses indicated a marginally significant effect of weight loss on unadjusted weighted mean difference of 25-OHD (β= −0.60 [95% confidence interval {CI}, −1.24 to +0.04] nmol/ L; P = .06) and after adjustment for study quality (Jadad score ≥3) (β= −0.64 [95% CI, −1.28 to +0.01] nmol/L; P = .05). The effect of percent fat mass on weighted mean difference of 25OHDwas also marginally significant before (β = −0.91 [95% CI, −1.96 to +0.15] nmol/L; P =.08) and after adjustment of study quality (β = −1.05 [95% CI, −2.18 to +0.08] nmol/L; P = .06). |
|  |  |  |  | Human *in vivo* (prospective intervention study, 24 months, n=383 overweight or obese women) | N/A | N/A | N/A, not likely (weight loss increases serum OHVitD) | (Rock et al., 2012) | Obesity is associated with lower serum 25-hydroxyvitamin D (direction of causality not evaluated).  383 overweight or obese women who participated in a 2-year clinical trial of a weight-loss program. Weight loss, presumably associated with a reduction in body fat, is associated with increased serum 25(OH)D concentration in overweight or obese women. |
|  |  |  |  | Human *in vivo* (prospective epidemiology, n=4659 elderly women, observed for 4.5 years) | N/A | N/A | N/A, not likely (weight loss increases serum OHVitD) | (LeBlanc et al., 2012) | 4659 women aged 65 years with baseline 25(OH)D measurements were monitored for 4.5 years in a population-based multicentre US prospective cohort study of 9704 people (Study of Osteoporotic Fractures [SOF]).  Higher 25(OH)D levels have been found to be associated with lower weight gain. These results suggest that low vitamin D levels may predispose towards fat accumulation. |
|  |  |  |  | Mouse *in vitro*, differentiated 3T3-L1 preadipocytes,  (1-100 nM) 24-48 h | Yes (increased gene expression, 100 nM, 24 h) | No (decreased gene expression, 100 nM, 24 h) | No (decreased lipid accumulation in mature adipocytes) | (Chang & Kim, 2016) | 1,25(OH)2D exposure (24 h, 100 nmol/L) caused a decrease in intracellular fat accumulation and an increase of basal and isoproterenol-stimulated lipolysis without cytotoxicity in adipocytes. Adipogenic gene levels were decreased. Conversely, mRNA levels of beta oxidation–related genes, lipolytic enzymes, and vitamin D responsive gene were elevated by 1,25(OH)2D exposure. Additionally, significant incremental changes in NAD levels, the ratio of NAD to NADH, and SIRT1 expression and activity were noted in 1,25(OH)2D-treated 3T3-L1 adipocytes. |
|  |  |  |  | Mouse *in vitro,*  3T3-L1 differentiated adipocytes, (5-25 μM) 2-6 d  Zebrafish *in vivo,*  WT 5 dpf  (2 or 4 μM) 12-15 d | N/A | decreased gene expression in zebrafish | No, suppression of adipogenesis/ lipid accumulation | (J. H. Kim et al., 2016) | Cholecalciferol inhibited lipid accumulation in both experimental models (3T3-L1 murine differentiated adipocytes and juvenile zebrafish), especially when administered simultaneously with the hormonal adipogenesis induction cocktail. |
|  |  |  |  | Review | N/A | N/A | Uncertain (in humans) | (Szymczak-Pajor et al., 2022) | Although results from clinical trials are not consistent, vitamin D has the potential to be a treatment option for adipose tissue dysfunction.  While some showed improvement, some had no effect of this vitamin on metabolic and insulin resistance parameters. |
|  |  |  |  | Human, *in vitro*, THP-1 monocyte-derived macrophages  (0.1-100 μM)  24 h | N/A | Decreased PPARγ1 expression | N/A (reduced lipid accumulation in macrophages) | (Marino et al., 2022) | Macrophages were co-exposed in fatty acid-enriched culture medium (500 μM oleic/palmitic acid, 2:1 ratio).  50 and 100 nM Vit D3 significantly decreased accumulation of fatty acids in macrophages by 27% and 32%, respectively. |
|  |  |  |  | Review | N/A | Yes increased in human cells, decreased in murine cells | Yes increased in human cells, decreased in murine cells | (Park & Han, 2021) | Adipose tissue serves as the main site for vitamin D storage and is among the most important extraskeletal targets of vitamin D which can modulate multiple aspects of adipose tissue biology. Vitamin D may exert inhibitory or stimulatory effects on adipocyte differentiation depending on cell type, stage of differentiation, and the treatment time point. |
|  |  |  |  | Review | N/A | Uncertain (potentially yes, via β-catenin and WNT10 suppression) (PPARγ is suppressed in murine 3T3-L1) | Yes, in human preadipocytes (likely via VDR) and porcine MSCs, not in murine 3T3-L1 preadipocytes | (Mutt et al., 2014) | While a higher BMI was causally related to lower 25-hydroxyvitamin D (25(OH)D), no evidence was obtained for a BMI lowering effect by higher 25(OH)D.  The molecular basis of the interactions of 1,25(OH)2D3, vitamin D binding proteins (VDBPs) and nuclear vitamin D receptor (VDR) after sequestration in adipose tissue and their regulations are still unclear. 1,25(OH)2D3 and its inactive metabolites are known to **inhibit the formation of adipocytes in mouse 3T3-L1 cell line. In humans, 1,25(OH)2D3 promotes preadipocyte differentiation under cell culture conditions**.  In transgenic mice, the specific overexpression of human VDR in adipose tissue leads to increased adipose tissue mass.  1,25(OH)2D3 has an active role in adipose tissue by **modulating inflammation, adipogenesis** and adipokine secretion. |
|  |  |  |  | Human *in vitr*o, hASCs  (0.1-10 nM)  14 d | N/A | Yes | Equivocal (significant decrease in lipid accumulation at 0.1 nM; significant increase at 10 nM) | (Salehpour et al., 2021) | Treatment of human preadipocytes with 1,25-dihydroxyvitamin D3 significantly altered expression of adipogenic markers and triglyceride accumulation in a concentration-dependent manner. 1,25-dihydroxyvitamin D3 at concentration of 10 nM enhanced expression of SREBP1c, C/EBPβ, PPARγ, FASN, LPL. |
|  |  |  |  | Human *in vivo* (clinical open trial) | N/A | N/A | N/A | (Dantas et al., 2021) | ASCs: adipose tissue-derived stromal stem cells  prospective, dual-center, open trial, patients with recent onset T1D received one dose of allogenic ASC (1 x 10^6^  cells/kg) and cholecalciferol 2,000 UI/day for 6 months (n=7 patients) compared to patients who received cholecalciferol only (n=4), or standard insulin treatment (n=6).  Allogenic ASC + cholecalciferol without immunosuppression was safe and might have a role in the preservation of β-cells in patients with recent-onset T1D. |
|  |  |  |  | *In vitro*, 3T3-L1 murine differentiated adipocytes, 100 nM,  24 h | N/A | N/A | N/A, uncertain (lipid accumulation not quantified) | (Zoico et al., 2014) | Vitamin D3, as well as 1,25[OH]2D3 showed **anti-inflammatory** effects on 3T3-L1, as well as that adipocytes have the enzymatic pathways necessary to locally regulate the production of active forms of vitamin D, capable of influencing adipocyte phenotype and function.  CYP27A1 and CYP27A1 were induced upon exposure to Vitamin D3. It reduced secretion of IL-6, IL-10, and TNFα under inflammatory cell culture conditions (LPS stimulation).  Adipocytes are larger (cell area) and have lipid droplets with a lower optical density. However, these parameters are not related to/ discussed in relation to adipocyte differentiation; lipid accumulation is not quantified. It is hypothesised that Vitamin D3 can protect cells from insulin resistance together with its anti-inflammatory properties. |
|  |  |  |  | Mouse *in vivo* (C57BL/6J male mice, n=6-8 per group, 15,000 IU/kg bw/d oral gavage, 4 d) | N/A | N/A | No effect (*in vivo*; N/A *in vitro*) | (Bonnet et al., 2018) | Via VDR activation: decreased gene expression in WAT for:  cholecalciferol metabolism (CYP24A1, CYP27A1), cubilin (confirmed in 3T3-L1 murine preadipocytes and human primary adipocytes).  Human/mouse *in vitro* (3T3-L1 adipocytes or primary human adipocytes, 1-100 nM, 24 h). |
|  |  |  |  | Human, *in vivo* (clinical trial, n=334 healthy overweight or obese individuals, 12 months, 20,000 IU cholecalciferol 1-2x per week) | N/A | N/A | No effect | (Sneve, Figenschau, & Jorde, 2008) | Randomized double blind clinical trial with 20 000 IU cholecalciferol twice a week, or 20 000 IU once a week plus placebo, or placebo twice a week, for 12 months. All subjects were given 500 mg calcium supplementation. (age 21–70 years, body mass index (BMI) 28.0–47.0 kg/m2).  No significant change in weight, waist-to-hip ratio (WHR) or percentage body fat in any of the groups, nor between them. |
| Pyridaben | 96489-71-3 | 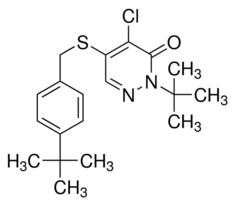 | Pesticide | **SUMMARY** | N/A | Potentially Yes | Uncertain (one report of strong adipogenesis inhibition) |  | Pyridaben was de-prioritised from proficiency testing in the Goliath pre-validation study due to inconsistent performance both in different adipocyte differentiation experiments, and during the HG5LN PPARγ transcriptional activation test method pre-validation. These were thought to be in part due to solubility difficulties, but could not be resolved. Therefore pyridaben is not considered a high-priority chemical for this chemical selection. |
|  |  |  |  | High-throughput screening assay, ToxCast and ToxPi  *In vitro*, 3T3-L1 murine pre-adipocytes,  and mBMSCs  Adipogenesis assay concentrations: 0.02, 0.2, 2, and 20 μM  14 d | N/A | Yes (moderate; EC50 = 2.98 μM, EC10 = 0.53 μM) | Inhibitor (at all test concentrations) | (Janesick et al., 2016) | Range tested 3x10^-8^ – 10^-4^ M  In Attagene PPARγ assay: EC50 = 10.08 μM, EC10 = 2.57 μM  Pyridaben (a PPARγ activator) strongly inhibited adipogenesis. |
| LGD1069 (Targretin)  Bexarotene | 153559-49-0 | 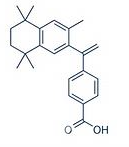 | Pharmaceutical | **SUMMARY**: | - | Uncertain/ potentially yes | ? | (Schierle & Merk, 2019) | Selective RXR agonist. An RAR activator at high concentrations. 9-*cis* RA was excluded because it activates RAR as does all-*trans* RA. Moreover, it can be photo- or thermally isomerized to other forms (same with atRA). The best available RXR ligands are LGD100268 and AGN 194204  Adipogenesis inducer in 3T3 L1 cell line (Filer et al 2022) |
|  |  |  |  | Mouse *in vivo*,  OAB-14 treatment groups (25, 50, and 100 mg/kg),  3 m  *In vitro*, murine microglial cell line BV-2  (0.1 µM, 1 µM, 2 µM, 4 µM)  24 h |  |  |  | (Zhang et al., 2023) | OAB-14 is a small-molecule derived from bexarotene.  OAB-14 promoted the M2 polarization of LPS-activated BV2 cells through the PPARγ pathway.  OAB-14 is reported to regulate the polarization of microglia and promote their differentiation from the M1 to M2 phenotype mediated through the PPARγ pathway, thereby effectively reducing neuroinflammation. |
|  |  |  |  | Rat *in vitro*, NMU417 cell line,  (10^-11^-10^-6^ M)  up to 80 h |  | Yes (inferred from expression of PPRE-controlled genes) | Yes | (Agarwal et al., 2000) | Targretin caused an increase of adipocyte differentiation in responding tumors.  Increased expression of adipocyte-related genes aP2, adipsin and PPARγ correlate with targretin mediated regression of rat mammary carcinomas. |
|  |  |  |  | Rat *in vivo*, (0.1 mg/kg)  7 d  Rat *in vitro,* synaptosomes obtained from brain tissue |  | Potentially yes (inferred from gene expression) |  | (Hacioglu, Kar, & Kanbak, 2021) | Bexarotene caused a moderate increase in SIRT1 levels with PPARγ activation.  Bexarotene and nicotinamide treatments significantly  increased PPARγ levels against Aβ(1-42) and the optimum  result was obtained in bexarotene treatment.  No statistically signifcant increase in PARP1 levels. |
| AGN194204 (IRX194204) | 220619-73-8 | 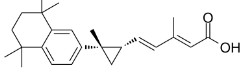 |  | **SUMMARY** | Weak antagonist | ? | ? |  | PPARα weak antagonist  Selective RXR agonist  Anti-inflammatory and anticarcinogenic properties. |
|  |  |  |  |  | - Inactive (potentially weak antagonist) |  |  | (Wang et al., 2016) | PPARα weak antagonist  Selective RXR agonist (0.1-1 nM); slight activation of RARα/β/γ 0.1-1 µM, no activation of FXR, LXRα/β, PPARγ up to 11-10 µM.  Anti-inflammatory and anticarcinogenic properties. |
|  |  |  |  | Human *in vitro,*  breast cancer cell lines  (MCF7, T47D, MDA-MB-468, SK-BR-3)  (100 nmol/l)  24 h |  |  |  | (Crowe & Chandraratna, 2004) | AGN194204 potentiated the antitumor effects of PPAR ligands.  PPARα and PPARγ ligands induced apoptotic and antiproliferative responses in breast cancer cell lines, respectively, which were associated with specific changes in gene expression. These responses were potentiated by the RXR-selective ligand AGN194204. RXR-α overexpressing RA-resistant breast cancer cell lines were more sensitive to the effects of the RXR-selective compound. |
| TTNPB 4-[(E)-2-(5,6,7,8-Tetrahydro-5,5,8,8-tetramethyl-2-naphthalenyl)-1-propenyl] benzoic acid, Arotinoid acid | 71441-28-6 | 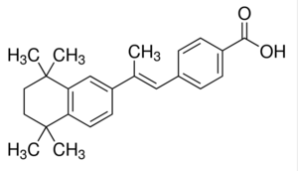 |  | **SUMMARY** | ? | ? | strong inhibitor of adipogenesis, but only one reference |  | Synthetic retinoid that acts as a selective agonist for the retinoic acid receptors (RARs).Strong inhibitor of adipogenesis; unlike retinoic acids (*9cisRA*) that promotes adipogenesis. Antineoplastic agent, a retinoic acid receptor agonist, and a teratogenic agent. It is a member of benzoic acids, a retinoid and a member of naphthalenes. |
|  |  |  |  | Human *in vitro*, HepG2 and  HeLa cells  (1 µM)  24 h |  |  |  | (Li et al., 2005) | Retinoids increase apo C-III expression at the transcriptional  Level. Increased apo C-III transcription is not mediated by TTNPB. |
| Fludioxonil | 131341-86-1 | 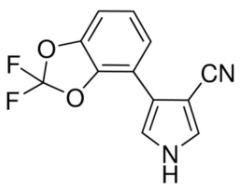 | Non-systemic fungicide | **SUMMARY** | N/A | -? | Uncertain/ potentially “Yes” |  | RXRα agonist (AC50 = 14.3 µM). Adipogenic in 3T3-L1 and mBMSCs. |
|  |  |  |  | High-throughput screening assay, ToxCast and ToxPi  *In vitro*, 3T3-L1 murine pre-adipocytes,  and mBMSCs  Adipogenesis assay concentrations: 0.02, 0.2, 2, and 20 μM  14 d | N/A | No | Yes (significant from 0.2 μM in 3T3-L1 and at 20 µM in mBMSC) | (Janesick et al., 2016) | Fludioxonil activated RXRα.  RXRα agonist (AC50 = 14.3 µM). Adipogenic in 3T3-L1 and mBMSCs. |
|  |  |  |  | Human, *in vitro*,  (several assays, models, endpoints, and times) | N/A | Yes, likely (4 hits for PPARγ gene expression) | Yes, strong (“positive” in all 8 assays) | (Foley et al., 2017) | Fludioxonil was positive in all adipogenesis-related assays (8 in total), including PPARγ-related activity, lipid acccumulation in human subcutaneous adipose-derived stem cells, and adiponectin secretion. AC50 values are in the range of ~10(-100) μM (median AC50=18.77 μM).  Data were analysed with a pipeline similar to that implemented in ToxCast, but with slight adaptations, such as setting of activity thresholds based on multiples of background noise and removal of outliers. |
|  |  |  |  | *In vitro*, 3T3-L1 murine preadipocytes and OP9  10 d  Human *in vitro*, primary human subcutaneous preadipocytes  14 d  Max. Tested Conc.: 2x10^-5^ M  Max. Non-Toxic Conc.: 2x10^-6^ M |  | Yes, (potential agonist from literature, reinforced herein) | Uncertain (increased vs. Control in 3T3-L1 and OP9, but not statistically significant) | (Kim et al., 2021) | Endpoints: lipid accumulation, RNA-Seq and confirmation of gene expression by RT-qPCR  Data-driven hierarchical lustering approach to identify PPARγ modulating chemicals, and transcriptional changes related to differentiation into white or brite/beige adipose tissue.  The authors compared a strong PPARγ therapeutic agonist that also was shown to modify PPARγ phosphorylation (i.e., ROSI, a chemical that was shown to modify only PPARγ phosphorylation (i.e., roscovitine), a weak PPARγ agonist and endogenous molecule (i.e., 15d-PGJ2), and two known environmental PPARγ ligands [i.e., TBBPA and TPhP].  Important genes for predicting PPARγ ligand/modification status, specifically the down-regulation of *Rpl13* and the upregulation of *Cidec*.  3T3-L1: lipid accumulation ~2-fold higher than in solvent control, but not statistically significantly different. |
| GW3965 hydrochloride | 405911-17-3 | 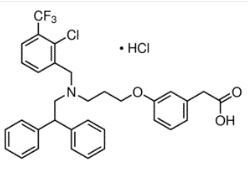 | Experimental pharmaceutical | **SUMMARY** | Uncertain/ likely “No” | Uncertain/ weak | Uncertain/ weak |  | LXR(β) agonist; commits human mesenchymal cells to adipose lineage, but evidence is mixed/conflicting.  LXR is involved in the tissue distribution of fat (visceral vs. Sub-cutaneous vs. Skeletal muscle) and activation reverses cholesterol transport. LXR activation induces steatosis and may affect pancreatic beta cells. Crosstalk with PPARγ.  One study noted, that effects accounted to LXR might, in fact, be mediated by PXR (Mitro et al., 2007). |
|  |  |  |  | HG5LN reporter cell lines for PPARγ (10^-8^ – 10^-5^ M)  24 h | N/A | Yes (EC20: 4.6 μM, EC50: n.d., 38% max activity) | N/A | (Garoche et al., 2021) | Human, mouse, zebrafish, and *Xenopus* PPARγ were tested using known hPPARγ ligands, and environmental chemicals.  HPPARγ and mPPARγ showed similar activities, with marked differences in xPPARγ (less pronounced) and zfPPARγ (more pronounced).  Only data for hPPARγ were extracted here (activity parameters for other receptors/ species is given in the publication). |
|  |  |  |  | Mouse *in vivo* (*ob/ob* female Mice, 4-5 weeks old, 10 mg/kg bw/d)  5 w | N/A | N/A | Uncertain/ no.  (No effect on total body fat, but fat re-distribution) | (Archer et al., 2013) | Redistribution of fat: decreased visceral fat. Increased subcutaneous fat without affecting whole body fat content. Lipidomics: change in lipid composition towards less lipotoxic lipids. No statistical effect, but a trend for improvement, were observed for insulin sensitivity after treatment, esp. In peripheral tissues (adipose + skeletal muscle). |
|  |  |  |  | Human *in vitro*, primary human hepatocytes  (2 μM)  48 h | N/A | N/A | N/A | (Santinha et al., 2020) | Lipid metabolism gene transcription, fatty acid profile, cell membrane biochemical properties. ELOLV6 and SCD1 mRNA increase was consistent with higher C16:1 and C18:1n9 at the expense of C16:0 and C18:0. The reduction of C18:2n6 and increase in C20:2n6 was in agreement with ELOVL5 upregulation. AGXT2L1 gene as induced by **LXR activation** by both synthetic and endogenous agonist treatment. |
|  |  |  |  | Mouse *in vitro,* mBM-MSC  (1 μM)  2 d | N/A (likely negligible) | N/A (likely negligible) | No | (Shoucri et al., 2017) | Cells were exposed to chemicals for 2 d, followed by 14 d differentiation/ growth *in vitro* (commitment assay).  GW3965 was used as a model LXR agonist. |
|  |  |  |  | Mouse *in vitro*, 3T3-L1 differentiated adipocytes,  (1-10 μM)  24 h | N/A | Uncertain (possibly via interference from LXR) | N/A | (Zheng et al., 2014) | Decreased adiponectin expression mediated via LXRα.  LXR activation may induce insulin resistance with decreased adiponectin signalling in epididymal fat, probably due to negative regulation of PPARγ signalling. |
|  |  |  |  | Hamster, *in vivo* (dietary-induced dyslipidaemic hamster model, 30mg/kg bw in diet)  10 d | N/A | N/A | N/A (possibly no effect) | (Briand et al., 2010) | The cholesterol-enriched diet increased VLDL-triglycerides and total cholesterol levels in all lipoprotein fractions and strongly increased liver lipids. Overall, **GW3965 failed to improve both dyslipidemia and liver steatosis**.  Despite no beneficial effect on dyslipidemia, LXR activation promotes macrophage-to-feces RCT (reverse cholesterol transport) in dyslipidemic hamsters. These results emphasize the use of species with a more human-like lipoprotein metabolism for drug profiling.  LXR activation has no beneficial effect on dyslipidaemia despite reversing cholesterol transport to faeces. |
|  |  |  |  | Human, *in vitro*, chimeric reporter gene assay, 12-point dilution from 10 μM)  16 h | N/A | N/A | N/A | (Mitro et al., 2007) | GW3965 is an LXR ligand.  T0901317 is dual agonist for LXR & PXR (nM potency). PXR genes activated: CD36 (scavenger receptor). Induces dramatic liver steatosis, while **GW3965 has a milder effect on steatosis**.  **Effects accredited to LXR could, in fact, be mediated by PXR**. |

**Note:** Natural/endogenous ligands for LXR, oxysterols, and positive control for GR, dexamethasone, are lower priority chemical considerations at this stage. Established that there is no GR crosstalk for the PPAR assays, and the intended minimum number of chemicals to be proposed.

**Supplementary Table 2. Summary of other cell types and nonselected chemicals for the (pre-)validation of the hPPARα, hPPARγ transcriptional agonism, and hMSC adipogenesis test methods.** Unless stated differently, p ≤ 0.05 in considered statistically significantly different. ag: agonist, antag: antagonist.

| **Chemical** | **Model** | **hPPARα ag/antag** | **hPPARγ ag/antag** | **hMSC adipogenesis (lipid accumulation)** | **References** | **Comments/ inclusion in test method reference chemical set?** |
| --- | --- | --- | --- | --- | --- | --- |
| Dexamethasone  ROSI | *In vitro,* 3T3-L1 murine cell line  Dexamethasone  (250nM)  ROSI  (5 μM)  4 and 8 h | N/A | N/A | Epigenetic miRNA mechanisms in the adipogenesis process | (Peshdary & Atlas, 2018) | miR-155 levels were up-regulated (2.4-fold) by glucocorticoids in differentiating 3T3-L1 preadipocytes, and this enhancement was abolished in the presence of RU486, a glucocorticoid receptor antagonist. In contrast, treatment with ROSI, another adipogenic inducer decreased the expression levels of miR-155 in these cells. Further, authors stated that the data shows that endogenous miR-155 is unlikely to be involved in adipogenesis as both dexamethasone and ROSI induced adipogenesis to similar levels. Using miR-155 inhibitor, the dexamethasone mediated miR-155 enhancement did not alter adipogenesis.  Dexamethasone but not ROSI increases miR-155 expression and increased expression of miR-155 is not involved in the dexamethasone-mediated adipogenesis in the 3T3-L1 model. |
| Polybrominated diphenyl ethers (PBDE) mixture | *In vitro,* 3T3-L1 murine cell line  (1.6, 3.2, 6.4, 12.8, 25.5 μM)  DE–71 39.3%  DE-79 0.7%  BDE-209 1.0%  BDE-47 40.8%  BDE-100 5.0%  BDE-28 3.9%  BDE-153 9.3%  DE-71 (0.8, 1.5, 3, 6, or 12 μM), BDE-47 (0.8, 1.5, 3, 6, or 12 μM) |  | ag |  | (Tung et al., 2014) | The mixture, DE-71, and BDE-47 induced adipocyte differentiation as assessed by markers for terminal differentiation [fatty acid binding protein 4 (aP2) and perilipin] and lipid accumulation. Characterization of the differentiation process in response to PBDEs indicated that adipogenesis induced by a minimally effective concentration of dexamethasone was enhanced by these PBDEs. Moreover, C/EBPα, PPARγ, and LXRα were induced late in the differentiation process. Taken together, these data indicate that adipocyte differentiation is induced by PBDEs; they act in the absence of glucocorticoid and enhance glucocorticoid-mediated adipogenesis.  PPARγ mRNA levels were induced by dexamethasone beginning at day 2, and this increase was sustained throughout the differentiation process. Treatment with the PBDE mixture, DE-71, or BDE-47 throughout the differentiation process caused increases in PPARγ transcript levels at day 8, although only the treatment with the PBDE mixture reached statistical significance.  PBDEs on the Stockholm POPs convention list. |
| Glucocorticoids | *In vitro,* 3T3-L1 murine cell line  Human subcutaneous primary preadipocytes  Dexamethasone  (10^−6^ M, 10^−7^ M, 10^−8^ M, 10^−9^ M)  48 h |  |  | Transcription factors for adipogenesis | (Tomlinson et al., 2010) | Exposure of human primary preadipocytes to glucocorticoids increases their sensitivity to insulin and enhances their subsequent response to stimuli that promote differentiation. This effect was observed in primary human preadipocytes but not in immortalized 3T3-L1 murine preadipocytes or in fully differentiated primary human adipocytes. Stimulation of insulin signalling was mediated through induction of insulin receptor (IR), IR substrate protein 1 (IRS1), IRS2, and the p85 regulatory subunit of phosphoinositide-3-3-kinase, which led to enhanced insulin-mediated activation of Akt. Although induction of IRS2 was direct, induction of IR and IRS1 by glucocorticoids occurred subsequent to primary induction of the forkhead family transcription factors FoxO1A and FoxO3A. |
| Dechlorane Plus (DP) | *In vitro,* 3T3-L1 murine cell line  Human primary subcutaneous (Sc) and omental (mesenchymal) preadipocytes  (0.001–10 µM)  2, 4, 6, or 8 d |  | antag | Adipogenic markers  (Fabp4, Lpl, Plin, adipsin, and adiponectin) | (Peshdary et al., 2019) | DP significantly (p < 0.05) increased lipid accumulation (2.5-fold) and the mRNA expression of adipogenic markers: fatty acid binding protein 4 (Fabp4), lipoprotein lipase (Lpl), perilipin (Plin), adipsin, and adiponectin. DP also significantly (p < 0.05) increased the protein levels of selected mature adipocyte markers. DP increased PPARγ transcriptional activity by threefold (p < 0.05) in luciferase reporter assays. When the PPARγ agonist was replaced by DP in the human preadipocyte differentiation cocktail, DP significantly (p < 0.05) increased the mRNA levels of adipogenic markers, PPARγ, FABP4, and PLIN in human Sc as well as Om cultures. PPARγ antagonist studies indicated that DP-mediated upregulation of adipogenic markers Fabp4 and Lpl did not occur via PPARγ activation. |
| MIX (an inhibitor of cAMP phosphodiesterases that elevate cAMP levels) | Monkey *in vitro,* COS7 cells  Mouse *in vitro,* NIH 3T3 and 3T3-L1 cell lines  (MIX: 500 μm and insulin (MI): 100 nm, MIX, insulin, and dexamethasone (MID): 250 nm)  24 h |  |  | Epigenetic markers for repression of pre- adipocyte differentiation (HDAC1) | (Abdou, Atlas, & Haché, 2011) | Requirements for binding of mSin3A/HDAC1 to LAP/LAP* and LIP to a 4-amino acid motif in the central region of LAP/LAP* (residues 153–156) and the N terminus of LIP were mapped. Reducing mSin3A/HDAC1 binding to LAP/LAP* and LIP through deletion of this motif reduced the recruitment of HDAC1 to the C/EBPα promoter and increased preadipocyte differentiation stimulated by insulin and 1-methyl-3-isobutylxanthine. Additional studies on the interaction of HDAC1 with LIP showed active repression of C/EBPα transcription. This was considered to be largely responsible for the ability of LIP and HDAC1 to repress preadipocyte differentiation. Although mSin3A/HDAC1 interacted readily with LAP/LAP* in addition to LIP, and expression of LAP/LAP* was sufficient to recruit HDAC1 to the C/EBPα promoter, mutations in C/EBPβ that abrogated HDAC1 association to LAP/LAP* in the absence of LIP, did not provide additional stimulation of differentiation or transcription, beyond the deletion of LIP alone. |
| Methylisobutylxanthine (MIX) Troglitazone Dexamethasone | Human *in vitro,* Human primary preadipocytes  *In vitro,* 3T3-L1 murine cell line  MIX (0.5 mM) from d 0–4,  Troglitazone (5 μM) and dexamethasone (1 μM) |  |  |  | (Tomlinson et al., 2006) | Glucocorticoid treatment stimulated the early accumulation of CCAAT enhancer binding protein-β (C/EBPβ) in primary human preadipocytes. Induction of C/EBPα in primary human preadipocytes was observed within 4 h of adipogenic stimulus, whereas C/EBPα induction was not detected until 24–48 h in the murine 3T3 L1 preadipocyte model. **In contrast to human primary preadipocytes, which do not undergo post confluent mitosis, 3T3 L1 murine preadipocytes stimulated to differentiate under chemically defined conditions, required glucocorticoids** to survive the clonal expansion that precedes terminal differentiation, revealing a novel signal imparted by glucocorticoids in this murine cell system. |
| Firemaster® 550 (FM550): comprised of TBB, TBPH, TPP and IPTP | *In vitro,* 3T3-L1 murine cell line |  | ag |  | (Tung et al., 2017) | FM550 and its components TPP, IPTP, and TBPH, but not TBB induced lipid accumulation in a concentration-dependent manner. TBPH did not alter the mRNA or protein expression of terminal differentiation markers. In contrast, FM550, TPP, and IPTP treatment enhanced lipid accumulation, and mRNA and protein expression of terminal differentiation markers. Exposure to FM550, IPTP, and TPP significantly increased PPARγ mediated aP2 enhancer activity. TPP- and IPTP-dependent upregulation of aP2 was significantly inhibited by the selective PPARγ antagonist GW9662.  Chromatin immunoprecipitation experiments showed that IPTP and TPP treatment led to the recruitment of PPARγ to the regulatory region of aP2. |
| - | Mouse *in vitro,* NIH 3T3 and 3T3-L1 cell line |  |  | Epigenetic marker (HDAC1) | (Kuzmochka et al., 2014) | Small interference RNA-mediated knockdown of HDAC1 with generation of an enzymatically inactive HDAC1_D181A_ by site-directed mutagenesis, showed that HDAC1, but not HDAC2, suppresses glucocorticoid receptor-potentiated preadipocyte differentiation by decreasing CCAAT/enhancer-binding protein (C/ebp)_α_ and PPARγ expression levels, at the onset of differentiation.  HDAC1_D181A_ acts as a dominant negative mutant of HDAC1 during adipogenesis by modulating C/EBP_β_ transcriptional activity on the C/ebp_α_ promoter. |
| - | Monkey *in vitro,* COS7 cells  Mouse *in vitro,* NIH 3T3 cell line |  |  |  | (Abdou, Atlas, & Haché, 2013) | C/EBPβΔ141–149 transcriptional activity was compromised on the C/ebpα, but not on the PPARγ, promoter. Additionally, the ability of C/EBPβΔ141–149 to induce adipogenesis in NIH 3T3 cells was compromised when compared with C/EBPβwt owing to a delayed expression of C/ebpα at the onset of differentiation.  The reduced expression of C/ebpα in cells expressing C/EBPβΔ141–149 was due to a persistent recruitment of HDAC1 to the C/ebpα promoter after glucocorticoid treatment. Amino acids 141–149 of C/EBPβ act as a positive regulatory domain required for maximum transcriptional  activity. |
| 2,4-DTBP  2,6-DTBP | Human MSCs  (1-5-10µM) |  | ag |  | (Ren et al., 2023) | 2,4-DTBP exposure induced lipid accumulation in human MSCs with a corresponding increase in the induction of marker genes for white adipocyte differentiation. In contrast, 2.6-DTBP was inactive. 2,4-DTBP activated the PPARγ-RXRα heterodimer.  2,4-DTBP activated the TRβ/RXRα heterodimer by activating RXRα.  2,4-DTBP activated the RXRα component of PPARγ/RXRα, LXRα/RXRα, and TRβ/RXR heterodimers.  2,4,6-TTBP was reported to be a more potent activator of RXR, and receptor activation assays confirmed this. |

**1 References**

Abdou, H.-S., Atlas, E., & Haché, R. J. G. (2013). A Positive Regulatory Domain in CCAAT/Enhancer Binding Protein β (C/EBPβ) Is Required for the Glucocorticoid-Mediated Displacement of Histone Deacetylase 1 (HDAC1) from the C/ebpα Promoter and Maximum Adipogenesis. *Endocrinology*, *154*(4), 1454-1464. <https://doi.org/10.1210/en.2012-2061>

Abdou, H. S., Atlas, E., & Haché, R. J. (2011). Liver-enriched inhibitory protein (LIP) actively inhibits preadipocyte differentiation through histone deacetylase 1 (HDAC1). *J Biol Chem*, *286*(24), 21488-21499. <https://doi.org/10.1074/jbc.M110.211540>

Agarwal, V. R., Bischoff, E. D., Hermann, T., & Lamph, W. W. (2000). Induction of adipocyte-specific gene expression is correlated with mammary tumor regression by the retinoid X receptor-ligand LGD1069 (targretin). *Cancer Res*, *60*(21), 6033-6038.

Ahmed, S., & Atlas, E. (2016). Bisphenol S- and bisphenol A-induced adipogenesis of murine preadipocytes occurs through direct peroxisome proliferator-activated receptor gamma activation. *Int J Obes (Lond)*, *40*(10), 1566-1573. <https://doi.org/10.1038/ijo.2016.95>

Alfhili, M. A., & Lee, M. H. (2019). Triclosan: An Update on Biochemical and Molecular Mechanisms. *Oxid Med Cell Longev*, *2019*, 1607304. <https://doi.org/10.1155/2019/1607304>

Alonso-Magdalena, P., Ropero, A. B., Soriano, S., García-Arévalo, M., Ripoll, C., Fuentes, E., Quesada, I., & Nadal, Á. (2012). Bisphenol-A acts as a potent estrogen via non-classical estrogen triggered pathways. *Mol Cell Endocrinol*, *355*(2), 201-207. <https://doi.org/10.1016/j.mce.2011.12.012>

Angle, B. M., Do, R. P., Ponzi, D., Stahlhut, R. W., Drury, B. E., Nagel, S. C., Welshons, W. V., Besch-Williford, C. L., Palanza, P., Parmigiani, S., vom Saal, F. S., & Taylor, J. A. (2013). Metabolic disruption in male mice due to fetal exposure to low but not high doses of bisphenol A (BPA): evidence for effects on body weight, food intake, adipocytes, leptin, adiponectin, insulin and glucose regulation. *Reprod Toxicol*, *42*, 256-268. <https://doi.org/10.1016/j.reprotox.2013.07.017>

ANSES. (2014). *OPINION of the French Agency for Food, Environmental and Occupational Health & Safety in response to the consultation of the European Food Safety Authority on its draft Opinion regarding the assessment of risks to human health related to dietary exposure to Bisphenol A* (ANSES – Request no. 2014-SA-0033). <https://www.anses.fr/en/system/files/SUBSTANCES2014sa0033EN.pdf>

Archer, A., Stolarczyk, E., Doria, M. L., Helguero, L., Domingues, R., Howard, J. K., Mode, A., Korach-André, M., & Gustafsson, J. (2013). LXR activation by GW3965 alters fat tissue distribution and adipose tissue inflammation in ob/ob female mice. *J Lipid Res*, *54*(5), 1300-1311. <https://doi.org/10.1194/jlr.M033977>

Ariemma, F., D'Esposito, V., Liguoro, D., Oriente, F., Cabaro, S., Liotti, A., Cimmino, I., Longo, M., Beguinot, F., Formisano, P., & Valentino, R. (2016). Low-Dose Bisphenol-A Impairs Adipogenesis and Generates Dysfunctional 3T3-L1 Adipocytes. *PLoS One*, *11*(3), e0150762. <https://doi.org/10.1371/journal.pone.0150762>

Atlas, E., Pope, L., Wade, M. G., Kawata, A., Boudreau, A., & Boucher, J. G. (2014). Bisphenol A increases aP2 expression in 3T3L1 by enhancing the transcriptional activity of nuclear receptors at the promoter. *Adipocyte*, *3*(3), 170-179. <https://doi.org/10.4161/adip.28436>

Babiloni-Chust, I., Dos Santos, R. S., Medina-Gali, R. M., Perez-Serna, A. A., Encinar, J. A., Martinez-Pinna, J., Gustafsson, J. A., Marroqui, L., & Nadal, A. (2022). G protein-coupled estrogen receptor activation by bisphenol-A disrupts the protection from apoptosis conferred by the estrogen receptors ERα and ERβ in pancreatic beta cells. *Environ Int*, *164*, 107250. <https://doi.org/10.1016/j.envint.2022.107250>

Bastos Sales, L., Kamstra, J. H., Cenijn, P. H., van Rijt, L. S., Hamers, T., & Legler, J. (2013). Effects of endocrine disrupting chemicals on in vitro global DNA methylation and adipocyte differentiation. *Toxicol In Vitro*, *27*(6), 1634-1643. <https://doi.org/10.1016/j.tiv.2013.04.005>

Bertuloso, B. D., Podratz, P. L., Merlo, E., de Araújo, J. F., Lima, L. C., de Miguel, E. C., de Souza, L. N., Gava, A. L., de Oliveira, M., Miranda-Alves, L., Carneiro, M. T., Nogueira, C. R., & Graceli, J. B. (2015). Tributyltin chloride leads to adiposity and impairs metabolic functions in the rat liver and pancreas. *Toxicol Lett*, *235*(1), 45-59. <https://doi.org/10.1016/j.toxlet.2015.03.009>

Biasiotto, G., Zanella, I., Masserdotti, A., Pedrazzani, R., Papa, M., Caimi, L., & Di Lorenzo, D. (2016). Municipal wastewater affects adipose deposition in male mice and increases 3T3-L1 cell differentiation. *Toxicol Appl Pharmacol*, *297*, 32-40. <https://doi.org/10.1016/j.taap.2016.02.023>

Biemann, R., Navarrete Santos, A., Navarrete Santos, A., Riemann, D., Knelangen, J., Blüher, M., Koch, H., & Fischer, B. (2012). Endocrine disrupting chemicals affect the adipogenic differentiation of mesenchymal stem cells in distinct ontogenetic windows. *Biochem Biophys Res Commun*, *417*(2), 747-752. <https://doi.org/10.1016/j.bbrc.2011.12.028>

Bonnet, L., Karkeni, E., Couturier, C., Astier, J., Dalifard, J., Defoort, C., Svilar, L., Martin, J.-C., Tourniaire, F., & Landrier, J.-F. (2018). Gene Expression Pattern in Response to Cholecalciferol Supplementation Highlights Cubilin as a Major Protein of 25(OH)D Uptake in Adipocytes and Male Mice White Adipose Tissue. *Endocrinology*, *159*(2), 957-966. <https://doi.org/10.1210/en.2017-00650>

Boucher, J. G., Boudreau, A., Ahmed, S., & Atlas, E. (2015). In Vitro Effects of Bisphenol A β-D-Glucuronide (BPA-G) on Adipogenesis in Human and Murine Preadipocytes. *Environ Health Perspect*, *123*(12), 1287-1293. <https://doi.org/10.1289/ehp.1409143>

Boucher, J. G., Gagné, R., Rowan-Carroll, A., Boudreau, A., Yauk, C. L., & Atlas, E. (2016). Bisphenol A and Bisphenol S Induce Distinct Transcriptional Profiles in Differentiating Human Primary Preadipocytes. *PLoS One*, *11*(9), e0163318. <https://doi.org/10.1371/journal.pone.0163318>

Bougarne, N., Weyers, B., Desmet, S. J., Deckers, J., Ray, D. W., Staels, B., & De Bosscher, K. (2018). Molecular Actions of PPARα in Lipid Metabolism and Inflammation. *Endocr Rev*, *39*(5), 760-802. <https://doi.org/10.1210/er.2018-00064>

Briand, F., Tréguier, M., André, A., Grillot, D., Issandou, M., Ouguerram, K., & Sulpice, T. (2010). Liver X receptor activation promotes macrophage-to-feces reverse cholesterol transport in a dyslipidemic hamster model. *J Lipid Res*, *51*(4), 763-770. <https://doi.org/10.1194/jlr.M001552>

Burkhardt, P., Palma-Duran, S. A., Tuck, A. R. R., Norgren, K., Li, X., Nikiforova, V., Griffin, J. L., & Munic Kos, V. (2024). Environmental chemicals change extracellular lipidome of mature human white adipocytes. *Chemosphere*, *349*, 140852. <https://doi.org/https://doi.org/10.1016/j.chemosphere.2023.140852>

Burns, J. S., Williams, P. L., Korrick, S. A., Hauser, R., Sergeyev, O., Revich, B., Lam, T., & Lee, M. M. (2014). Association between chlorinated pesticides in the serum of prepubertal Russian boys and longitudinal biomarkers of metabolic function. *Am J Epidemiol*, *180*(9), 909-919. <https://doi.org/10.1093/aje/kwu212>

Cai, A., Portengen, L., Govarts, E., Martin, L. R., Schoeters, G., Legler, J., Vermeulen, R., Lenters, V., & Remy, S. (2023). Prenatal exposure to persistent organic pollutants and changes in infant growth and childhood growth trajectories. *Chemosphere*, *314*, 137695. <https://doi.org/10.1016/j.chemosphere.2022.137695>

Cano-Sancho, G., Smith, A., & La Merrill, M. A. (2017). Triphenyl phosphate enhances adipogenic differentiation, glucose uptake and lipolysis via endocrine and noradrenergic mechanisms. *Toxicol In Vitro*, *40*, 280-288. <https://doi.org/10.1016/j.tiv.2017.01.021>

Carwile, J. L., & Michels, K. B. (2011). Urinary bisphenol A and obesity: NHANES 2003-2006. *Environ Res*, *111*(6), 825-830. <https://doi.org/10.1016/j.envres.2011.05.014>

Ceotto Freitas-Lima, L., Merlo, E., Campos Zicker, M., Navia-Pelaez, J. M., de Oliveira, M., Dos Santos Aggum Capettini, L., Nogueira, C. R., Versiani Matos Ferreira, A., Sousa Santos, S. H., & Bernardes Graceli, J. (2018). Tributyltin impacts in metabolic syndrome development through disruption of angiotensin II receptor signaling pathways in white adipose tissue from adult female rats. *Toxicol Lett*, *299*, 21-31. <https://doi.org/10.1016/j.toxlet.2018.08.018>

Chamorro-García, R., Kirchner, S., Li, X., Janesick, A., Casey, S. C., Chow, C., & Blumberg, B. (2012). Bisphenol A diglycidyl ether induces adipogenic differentiation of multipotent stromal stem cells through a peroxisome proliferator-activated receptor gamma-independent mechanism. *Environ Health Perspect*, *120*(7), 984-989. <https://doi.org/10.1289/ehp.1205063>

Chandalia, A., Clarke, H. J., Clemens, L. E., Pandey, B., Vicena, V., Lee, P., Lavan, B. E., & Gregoire, F. M. (2009). MBX-102/JNJ39659100, a novel non-TZD selective partial PPAR-γ agonist lowers triglyceride independently of PPAR-α activation. *PPAR Res*, *2009*, 706852. <https://doi.org/10.1155/2009/706852>

Chang, E., & Kim, Y. (2016). Vitamin D decreases adipocyte lipid storage and increases NAD-SIRT1 pathway in 3T3-L1 adipocytes. *Nutrition*, *32*(6), 702-708. <https://doi.org/10.1016/j.nut.2015.12.032>

Chang, R. C., Joloya, E. M., Li, Z., Shoucri, B. M., Shioda, T., & Blumberg, B. (2023). miR-223 Plays a Key Role in Obesogen-Enhanced Adipogenesis in Mesenchymal Stem Cells and in Transgenerational Obesity. *Endocrinology*, *164*(5). <https://doi.org/10.1210/endocr/bqad027>

Cimmino, I., Fiory, F., Perruolo, G., Miele, C., Beguinot, F., Formisano, P., & Oriente, F. (2020). Potential Mechanisms of Bisphenol A (BPA) Contributing to Human Disease. *Int J Mol Sci*, *21*(16). <https://doi.org/10.3390/ijms21165761>

Cronet, P., Petersen, J. F., Folmer, R., Blomberg, N., Sjöblom, K., Karlsson, U., Lindstedt, E. L., & Bamberg, K. (2001). Structure of the PPARalpha and -gamma ligand binding domain in complex with AZ 242; ligand selectivity and agonist activation in the PPAR family. *Structure*, *9*(8), 699-706. <https://doi.org/10.1016/s0969-2126(01)00634-7>

Crowe, D. L., & Chandraratna, R. A. (2004). A retinoid X receptor (RXR)-selective retinoid reveals that RXR-alpha is potentially a therapeutic target in breast cancer cell lines, and that it potentiates antiproliferative and apoptotic responses to peroxisome proliferator-activated receptor ligands. *Breast Cancer Res*, *6*(5), R546-555. <https://doi.org/10.1186/bcr913>

Dantas, J. R., Araújo, D. B., Silva, K. R., Souto, D. L., de Fátima Carvalho Pereira, M., Luiz, R. R., Dos Santos Mantuano, M., Claudio-da-Silva, C., Gabbay, M. A. L., Dib, S. A., Couri, C. E. B., Maiolino, A., Rebelatto, C. L. K., Daga, D. R., Senegaglia, A. C., Brofman, P. R. S., Baptista, L. S., de Oliveira, J. E. P., Zajdenverg, L., & Rodacki, M. (2021). Adipose tissue-derived stromal/stem cells + cholecalciferol: a pilot study in recent-onset type 1 diabetes patients. *Arch Endocrinol Metab*, *65*(3), 342-351. <https://doi.org/10.20945/2359-3997000000368>

Desvergne, B., Feige, J. N., & Casals-Casas, C. (2009). PPAR-mediated activity of phthalates: A link to the obesity epidemic? *Mol Cell Endocrinol*, *304*(1-2), 43-48. <https://doi.org/10.1016/j.mce.2009.02.017>

Dirtu, A. C., Roosens, L., Geens, T., Gheorghe, A., Neels, H., & Covaci, A. (2008). Simultaneous determination of bisphenol A, triclosan, and tetrabromobisphenol A in human serum using solid-phase extraction and gas chromatography-electron capture negative-ionization mass spectrometry. *Anal Bioanal Chem*, *391*(4), 1175-1181. <https://doi.org/10.1007/s00216-007-1807-9>

Do, M. T., Chang, V. C., Mendez, M. A., & de Groh, M. (2017). Urinary bisphenol A and obesity in adults: results from the Canadian Health Measures Survey. *Health Promot Chronic Dis Prev Can*, *37*(12), 403-412. <https://doi.org/10.24095/hpcdp.37.12.02> (Concentration urinaire de bisphénol A et obésité chez les adultes : résultats de l’Enquête canadienne sur les mesures de la santé.)

Drobna, Z., Talarovicova, A., Schrader, H. E., Fennell, T. R., Snyder, R. W., & Rissman, E. F. (2019). Bisphenol F has different effects on preadipocytes differentiation and weight gain in adult mice as compared with Bisphenol A and S. *Toxicology*, *420*, 66-72. <https://doi.org/10.1016/j.tox.2019.03.016>

Dunder, L., Lejonklou, M. H., Lind, P. M., & Lind, L. (2019). Urinary bisphenol A and serum lipids: a meta-analysis of six NHANES examination cycles (2003-2014). *J Epidemiol Community Health*, *73*(11), 1012-1019. <https://doi.org/10.1136/jech-2019-212555>

EFSA. (2010). Panel on Dietetic Products, Nutrition, and Allergies (NDA), Scientific opinion on dietary reference values for fats, including saturated fatty acids, polyunsaturated fatty acids, monounsaturated fatty acids, trans fatty acids, and cholesterol. *EFSA Journal*, *8*(3), 1461. <https://doi.org/https://doi.org/10.2903/j.efsa.2010.1461>

EFSA. (2019). Updated statement on the available outcomes of the human health assessment in the context of the pesticides peer review of the active substance chlorpyrifos-methyl. *Efsa j*, *17*(11), e05908. <https://doi.org/10.2903/j.efsa.2019.5908>

EFSA Panel on Food Contact Materials, E., Flavourings, & Aids, P. (2015). Scientific Opinion on the risks to public health related to the presence of bisphenol A (BPA) in foodstuffs. *EFSA Journal*, *13*(1), 3978. <https://doi.org/https://doi.org/10.2903/j.efsa.2015.3978>

Eskenazi, B., Harley, K., Bradman, A., Weltzien, E., Jewell, N. P., Barr, D. B., Furlong, C. E., & Holland, N. T. (2004). Association of in utero organophosphate pesticide exposure and fetal growth and length of gestation in an agricultural population. *Environ Health Perspect*, *112*(10), 1116-1124. <https://doi.org/10.1289/ehp.6789>

FAO. (2006). *Joint FAO/WHO Meeting on Pesticide Residues, Pesticide residues in food 2006*. WHO Food and Agriculture Organization of the United Nations. <https://www.fao.org/fileadmin/templates/agphome/documents/Pests_Pesticides/JMPR/report2006jmpr.pdf>

Feige, J. N., Gelman, L., Rossi, D., Zoete, V., Métivier, R., Tudor, C., Anghel, S. I., Grosdidier, A., Lathion, C., Engelborghs, Y., Michielin, O., Wahli, W., & Desvergne, B. (2007). The endocrine disruptor monoethyl-hexyl-phthalate is a selective peroxisome proliferator-activated receptor gamma modulator that promotes adipogenesis. *J Biol Chem*, *282*(26), 19152-19166. <https://doi.org/10.1074/jbc.M702724200>

Feige, J. N., Gerber, A., Casals-Casas, C., Yang, Q., Winkler, C., Bedu, E., Bueno, M., Gelman, L., Auwerx, J., Gonzalez, F. J., & Desvergne, B. (2010). The pollutant diethylhexyl phthalate regulates hepatic energy metabolism via species-specific PPARalpha-dependent mechanisms. *Environ Health Perspect*, *118*(2), 234-241. <https://doi.org/10.1289/ehp.0901217>

Félix-Soriano, E., Sáinz, N., Fernández-Galilea, M., Gil-Iturbe, E., Celay, J., Martínez-Climent, J. A., & Moreno-Aliaga, M. J. (2023). Chronic docosahexaenoic acid supplementation improves metabolic plasticity in subcutaneous adipose tissue of aged obese female mice. *J Nutr Biochem*, *111*, 109153. <https://doi.org/10.1016/j.jnutbio.2022.109153>

Filer, D. L., Hoffman, K., Sargis, R. M., Trasande, L., & Kassotis, C. D. (2022). On the Utility of ToxCast-Based Predictive Models to Evaluate Potential Metabolic Disruption by Environmental Chemicals. *Environ Health Perspect*, *130*(5), 57005. <https://doi.org/10.1289/ehp6779>

Foley, B., Doheny, D. L., Black, M. B., Pendse, S. N., Wetmore, B. A., Clewell, R. A., Andersen, M. E., & Deisenroth, C. (2017). Editor's Highlight: Screening ToxCast Prioritized Chemicals for PPARG Function in a Human Adipose-Derived Stem Cell Model of Adipogenesis. *Toxicol Sci*, *155*(1), 85-100. <https://doi.org/10.1093/toxsci/kfw186>

Forman, B. M., Tontonoz, P., Chen, J., Brun, R. P., Spiegelman, B. M., & Evans, R. M. (1995). 15-Deoxy-delta 12, 14-prostaglandin J2 is a ligand for the adipocyte determination factor PPAR gamma. *Cell*, *83*(5), 803-812. <https://doi.org/10.1016/0092-8674(95)90193-0>

Gao, P., Wang, L., Yang, N., Wen, J., Zhao, M., Su, G., Zhang, J., & Weng, D. (2020). Peroxisome proliferator-activated receptor gamma (PPARγ) activation and metabolism disturbance induced by bisphenol A and its replacement analog bisphenol S using in vitro macrophages and in vivo mouse models. *Environ Int*, *134*, 105328. <https://doi.org/10.1016/j.envint.2019.105328>

García-Rojas, P., Antaramian, A., González-Dávalos, L., Villarroya, F., Shimada, A., Varela-Echavarría, A., & Mora, O. (2010). Induction of peroxisomal proliferator-activated receptor gamma and peroxisomal proliferator-activated receptor gamma coactivator 1 by unsaturated fatty acids, retinoic acid, and carotenoids in preadipocytes obtained from bovine white adipose tissue1,2. *J Anim Sci*, *88*(5), 1801-1808. <https://doi.org/10.2527/jas.2009-2579>

Garoche, C., Boulahtouf, A., Grimaldi, M., Chiavarina, B., Toporova, L., den Broeder, M. J., Legler, J., Bourguet, W., & Balaguer, P. (2021). Interspecies Differences in Activation of Peroxisome Proliferator-Activated Receptor γ by Pharmaceutical and Environmental Chemicals. *Environ Sci Technol*, *55*(24), 16489-16501. <https://doi.org/10.1021/acs.est.1c04318>

Grün, F. (2014). Chapter Eleven - The Obesogen Tributyltin. In G. Litwack (Ed.), *Vitamins & Hormones* (Vol. 94, pp. 277-325). Academic Press. <https://doi.org/https://doi.org/10.1016/B978-0-12-800095-3.00011-0>

Grün, F., & Blumberg, B. (2006). Environmental obesogens: organotins and endocrine disruption via nuclear receptor signaling. *Endocrinology*, *147*(6 Suppl), S50-55. <https://doi.org/10.1210/en.2005-1129>

Grün, F., Watanabe, H., Zamanian, Z., Maeda, L., Arima, K., Cubacha, R., Gardiner, D. M., Kanno, J., Iguchi, T., & Blumberg, B. (2006). Endocrine-disrupting organotin compounds are potent inducers of adipogenesis in vertebrates. *Mol Endocrinol*, *20*(9), 2141-2155. <https://doi.org/10.1210/me.2005-0367>

Grundy, S. M., Ahrens, E. H., Jr., Salen, G., Schreibman, P. H., & Nestel, P. J. (1972). Mechanisms of action of clofibrate on cholesterol metabolism in patients with hyperlipidemia. *J Lipid Res*, *13*(4), 531-551.

Hacioglu, C., Kar, F., & Kanbak, G. (2021). Ex Vivo Investigation of Bexarotene and Nicotinamide Function as a Protectıve Agent on Rat Synaptosomes Treated with Aβ(1-42). *Neurochem Res*, *46*(4), 804-818. <https://doi.org/10.1007/s11064-020-03216-7>

Heindel, J. J., Howard, S., Agay-Shay, K., Arrebola, J. P., Audouze, K., Babin, P. J., Barouki, R., Bansal, A., Blanc, E., Cave, M. C., Chatterjee, S., Chevalier, N., Choudhury, M., Collier, D., Connolly, L., Coumoul, X., Garruti, G., Gilbertson, M., Hoepner, L. A., . . . Blumberg, B. (2022). Obesity II: Establishing causal links between chemical exposures and obesity. *Biochem Pharmacol*, *199*, 115015. <https://doi.org/10.1016/j.bcp.2022.115015>

Héliès-Toussaint, C., Peyre, L., Costanzo, C., Chagnon, M. C., & Rahmani, R. (2014). Is bisphenol S a safe substitute for bisphenol A in terms of metabolic function? An in vitro study. *Toxicol Appl Pharmacol*, *280*(2), 224-235. <https://doi.org/10.1016/j.taap.2014.07.025>

Hellgren, L. I. (2010). Phytanic acid--an overlooked bioactive fatty acid in dairy fat? *Ann N Y Acad Sci*, *1190*, 42-49. <https://doi.org/10.1111/j.1749-6632.2009.05254.x>

Ho, J. C. H., Hsiao, C. D., Kawakami, K., & Tse, W. K. F. (2016). Triclosan (TCS) exposure impairs lipid metabolism in zebrafish embryos. *Aquat Toxicol*, *173*, 29-35. <https://doi.org/10.1016/j.aquatox.2016.01.001>

Hoogduijn, M. J., Rakonczay, Z., & Genever, P. G. (2006). The effects of anticholinergic insecticides on human mesenchymal stem cells. *Toxicol Sci*, *94*(2), 342-350. <https://doi.org/10.1093/toxsci/kfl101>

Hu, Y., Ding, G., Lv, C., Zhang, Q., Zhang, Y., Yuan, T., Ao, J., Gao, Y., Xia, Y., Yu, X., & Tian, Y. (2022). Association between triclosan exposure and obesity measures among 7-year-old children in northern China. *Ecotoxicol Environ Saf*, *239*, 113610. <https://doi.org/10.1016/j.ecoenv.2022.113610>

Husøy, T., Andreassen, M., Hjertholm, H., Carlsen, M. H., Norberg, N., Sprong, C., Papadopoulou, E., Sakhi, A. K., Sabaredzovic, A., & Dirven, H. A. A. M. (2019). The Norwegian biomonitoring study from the EU project EuroMix: Levels of phenols and phthalates in 24-hour urine samples and exposure sources from food and personal care products. *Environment International*, *132*, 105103. <https://doi.org/https://doi.org/10.1016/j.envint.2019.105103>

Jacobs, M. N., & Lewis, D. F. (2002). Steroid hormone receptors and dietary ligands: a selected review. *Proc Nutr Soc*, *61*(1), 105-122. <https://doi.org/10.1079/pns2001140>

Janesick, A. S., Dimastrogiovanni, G., Vanek, L., Boulos, C., Chamorro-García, R., Tang, W., & Blumberg, B. (2016). On the Utility of ToxCast™ and ToxPi as Methods for Identifying New Obesogens. *Environ Health Perspect*, *124*(8), 1214-1226. <https://doi.org/10.1289/ehp.1510352>

Jugan, J., Lind, P. M., Salihovic, S., Stubleski, J., Kärrman, A., Lind, L., & La Merrill, M. A. (2020). The associations between p,p'-DDE levels and plasma levels of lipoproteins and their subclasses in an elderly population determined by analysis of lipoprotein content. *Lipids Health Dis*, *19*(1), 249. <https://doi.org/10.1186/s12944-020-01417-1>

Kalliora, C., Kyriazis, I. D., Oka, S. I., Lieu, M. J., Yue, Y., Area-Gomez, E., Pol, C. J., Tian, Y., Mizushima, W., Chin, A., Scerbo, D., Schulze, P. C., Civelek, M., Sadoshima, J., Madesh, M., Goldberg, I. J., & Drosatos, K. (2019). Dual peroxisome-proliferator-activated-receptor-α/γ activation inhibits SIRT1-PGC1α axis and causes cardiac dysfunction. *JCI Insight*, *5*(17). <https://doi.org/10.1172/jci.insight.129556>

Kalloo, G., Calafat, A. M., Chen, A., Yolton, K., Lanphear, B. P., & Braun, J. M. (2018). Early life Triclosan exposure and child adiposity at 8 Years of age: a prospective cohort study. *Environ Health*, *17*(1), 24. <https://doi.org/10.1186/s12940-018-0366-1>

Kassotis, C. D., Hoffman, K., Völker, J., Pu, Y., Veiga-Lopez, A., Kim, S. M., Schlezinger, J. J., Bovolin, P., Cottone, E., Saraceni, A., Scandiffio, R., Atlas, E., Leingartner, K., Krager, S., Tischkau, S. A., Ermler, S., Legler, J., Chappell, V. A., Fenton, S. E., . . . Stapleton, H. M. (2021). Reproducibility of adipogenic responses to metabolism disrupting chemicals in the 3T3-L1 pre-adipocyte model system: An interlaboratory study. *Toxicology*, *461*, 152900. <https://doi.org/10.1016/j.tox.2021.152900>

Kassotis, C. D., Masse, L., Kim, S., Schlezinger, J. J., Webster, T. F., & Stapleton, H. M. (2017). Characterization of Adipogenic Chemicals in Three Different Cell Culture Systems: Implications for Reproducibility Based on Cell Source and Handling. *Sci Rep*, *7*, 42104. <https://doi.org/10.1038/srep42104>

Kim, H. K., Della-Fera, M., Lin, J., & Baile, C. A. (2006). Docosahexaenoic acid inhibits adipocyte differentiation and induces apoptosis in 3T3-L1 preadipocytes. *J Nutr*, *136*(12), 2965-2969. <https://doi.org/10.1093/jn/136.12.2965>

Kim, J., Sun, Q., Yue, Y., Yoon, K. S., Whang, K. Y., Marshall Clark, J., & Park, Y. (2016). 4,4'-Dichlorodiphenyltrichloroethane (DDT) and 4,4'-dichlorodiphenyldichloroethylene (DDE) promote adipogenesis in 3T3-L1 adipocyte cell culture. *Pestic Biochem Physiol*, *131*, 40-45. <https://doi.org/10.1016/j.pestbp.2016.01.005>

Kim, J. H., Kang, S., Jung, Y. N., & Choi, H. S. (2016). Cholecalciferol inhibits lipid accumulation by regulating early adipogenesis in cultured adipocytes and zebrafish. *Biochem Biophys Res Commun*, *469*(3), 646-653. <https://doi.org/10.1016/j.bbrc.2015.12.049>

Kim, S., Reed, E., Monti, S., & Schlezinger, J. J. (2021). A Data-Driven Transcriptional Taxonomy of Adipogenic Chemicals to Identify White and Brite Adipogens. *Environ Health Perspect*, *129*(7), 77006. <https://doi.org/10.1289/ehp6886>

Kim, U. J., & Oh, J. E. (2014). Tetrabromobisphenol A and hexabromocyclododecane flame retardants in infant-mother paired serum samples, and their relationships with thyroid hormones and environmental factors. *Environ Pollut*, *184*, 193-200. <https://doi.org/10.1016/j.envpol.2013.08.034>

Kitareewan, S., Burka, L. T., Tomer, K. B., Parker, C. E., Deterding, L. J., Stevens, R. D., Forman, B. M., Mais, D. E., Heyman, R. A., McMorris, T., & Weinberger, C. (1996). Phytol metabolites are circulating dietary factors that activate the nuclear receptor RXR. *Molecular Biology of the Cell*, *7*(8), 1153-1166. <https://doi.org/10.1091/mbc.7.8.1153>

Ko, A., Hwang, M. S., Park, J. H., Kang, H. S., Lee, H. S., & Hong, J. H. (2014). Association between Urinary Bisphenol A and Waist Circumference in Korean Adults. *Toxicol Res*, *30*(1), 39-44. <https://doi.org/10.5487/tr.2014.30.1.039>

Koshy, T. T., Attina, T. M., Ghassabian, A., Gilbert, J., Burdine, L. K., Marmor, M., Honda, M., Chu, D. B., Han, X., Shao, Y., Kannan, K., Urbina, E. M., & Trasande, L. (2017). Serum perfluoroalkyl substances and cardiometabolic consequences in adolescents exposed to the World Trade Center disaster and a matched comparison group. *Environ Int*, *109*, 128-135. <https://doi.org/10.1016/j.envint.2017.08.003>

Kratochvil, I., Hofmann, T., Rother, S., Schlichting, R., Moretti, R., Scharnweber, D., Hintze, V., Escher, B. I., Meiler, J., Kalkhof, S., & von Bergen, M. (2019). Mono(2-ethylhexyl) phthalate (MEHP) and mono(2-ethyl-5-oxohexyl) phthalate (MEOHP) but not di(2-ethylhexyl) phthalate (DEHP) bind productively to the peroxisome proliferator-activated receptor γ. *Rapid Commun Mass Spectrom*, *33 Suppl 1*(Suppl 1), 75-85. <https://doi.org/10.1002/rcm.8258>

Kuzmochka, C., Abdou, H. S., Haché, R. J., & Atlas, E. (2014). Inactivation of histone deacetylase 1 (HDAC1) but not HDAC2 is required for the glucocorticoid-dependent CCAAT/enhancer-binding protein α (C/EBPα) expression and preadipocyte differentiation. *Endocrinology*, *155*(12), 4762-4773. <https://doi.org/10.1210/en.2014-1565>

Lankester, J., Patel, C., Cullen, M. R., Ley, C., & Parsonnet, J. (2013). Urinary triclosan is associated with elevated body mass index in NHANES. *PLoS One*, *8*(11), e80057. <https://doi.org/10.1371/journal.pone.0080057>

Le Magueresse-Battistoni, B., Multigner, L., Beausoleil, C., & Rousselle, C. (2018). Effects of bisphenol A on metabolism and evidences of a mode of action mediated through endocrine disruption. *Mol Cell Endocrinol*, *475*, 74-91. <https://doi.org/10.1016/j.mce.2018.02.009>

LeBlanc, E. S., Rizzo, J. H., Pedula, K. L., Ensrud, K. E., Cauley, J., Hochberg, M., & Hillier, T. A. (2012). Associations between 25-hydroxyvitamin D and weight gain in elderly women. *J Womens Health (Larchmt)*, *21*(10), 1066-1073. <https://doi.org/10.1089/jwh.2012.3506>

Li, J., Ren, F., Li, Y., Luo, J., & Pang, G. (2019). Chlorpyrifos Induces Metabolic Disruption by Altering Levels of Reproductive Hormones. *J Agric Food Chem*, *67*(38), 10553-10562. <https://doi.org/10.1021/acs.jafc.9b03602>

Li, X., Hansen, P. A., Xi, L., Chandraratna, R. A., & Burant, C. F. (2005). Distinct mechanisms of glucose lowering by specific agonists for peroxisomal proliferator activated receptor gamma and retinoic acid X receptors. *J Biol Chem*, *280*(46), 38317-38327. <https://doi.org/10.1074/jbc.M505853200>

Liang, Y., Liu, D., Zhan, J., Luo, M., Han, J., Wang, P., & Zhou, Z. (2020). New insight into the mechanism of POP-induced obesity: Evidence from DDE-altered microbiota. *Chemosphere*, *244*, 125123. <https://doi.org/10.1016/j.chemosphere.2019.125123>

Liu, J., Chen, D., Huang, Y., Bigambo, F. M., Chen, T., & Wang, X. (2021). Effect of Maternal Triclosan Exposure on Neonatal Birth Weight and Children Triclosan Exposure on Children's BMI: A Meta-Analysis. *Front Public Health*, *9*, 648196. <https://doi.org/10.3389/fpubh.2021.648196>

Liu, Q., Wang, Q., Xu, C., Shao, W., Zhang, C., Liu, H., Jiang, Z., & Gu, A. (2017). Organochloride pesticides impaired mitochondrial function in hepatocytes and aggravated disorders of fatty acid metabolism. *Scientific Reports*, *7*(1), 46339. <https://doi.org/10.1038/srep46339>

Longo, M., Zatterale, F., Naderi, J., Nigro, C., Oriente, F., Formisano, P., Miele, C., & Beguinot, F. (2020). Low-dose Bisphenol-A Promotes Epigenetic Changes at Pparγ Promoter in Adipose Precursor Cells. *Nutrients*, *12*(11). <https://doi.org/10.3390/nu12113498>

Mangum, L. H., Howell, G. E., 3rd, & Chambers, J. E. (2015). Exposure to p,p'-DDE enhances differentiation of 3T3-L1 preadipocytes in a model of sub-optimal differentiation. *Toxicol Lett*, *238*(2), 65-71. <https://doi.org/10.1016/j.toxlet.2015.07.009>

Marino, M., Venturi, S., Del Bo, C., Møller, P., Riso, P., & Porrini, M. (2022). Vitamin D Counteracts Lipid Accumulation, Augments Free Fatty Acid-Induced ABCA1 and CPT-1A Expression While Reducing CD36 and C/EBPβ Protein Levels in Monocyte-Derived Macrophages. *Biomedicines*, *10*(4). <https://doi.org/10.3390/biomedicines10040775>

Marmugi, A., Ducheix, S., Lasserre, F., Polizzi, A., Paris, A., Priymenko, N., Bertrand-Michel, J., Pineau, T., Guillou, H., Martin, P. G. P., & Mselli-Lakhal, L. (2012). Low doses of bisphenol a induce gene expression related to lipid synthesis and trigger triglyceride accumulation in adult mouse liver. *Hepatology*, *55*(2), 395-407. <https://doi.org/https://doi.org/10.1002/hep.24685>

Martinez-Pinna, J., Marroqui, L., Hmadcha, A., Lopez-Beas, J., Soriano, S., Villar-Pazos, S., Alonso-Magdalena, P., Dos Santos, R. S., Quesada, I., Martin, F., Soria, B., Gustafsson, J., & Nadal, A. (2019). Oestrogen receptor β mediates the actions of bisphenol-A on ion channel expression in mouse pancreatic beta cells. *Diabetologia*, *62*(9), 1667-1680. <https://doi.org/10.1007/s00125-019-4925-y>

McMullen, P. D., Bhattacharya, S., Woods, C. G., Pendse, S. N., McBride, M. T., Soldatow, V. Y., Deisenroth, C., LeCluyse, E. L., Clewell, R. A., & Andersen, M. E. (2020). Identifying qualitative differences in PPARα signaling networks in human and rat hepatocytes and their significance for next generation chemical risk assessment methods. *Toxicology in Vitro*, *64*, 104463. <https://doi.org/https://doi.org/10.1016/j.tiv.2019.02.017>

McMullen, P. D., Bhattacharya, S., Woods, C. G., Sun, B., Yarborough, K., Ross, S. M., Miller, M. E., McBride, M. T., LeCluyse, E. L., Clewell, R. A., & Andersen, M. E. (2014). A map of the PPARα transcription regulatory network for primary human hepatocytes. *Chem Biol Interact*, *209*, 14-24. <https://doi.org/10.1016/j.cbi.2013.11.006>

Menale, C., Piccolo, M. T., Cirillo, G., Calogero, R. A., Papparella, A., Mita, L., Del Giudice, E. M., Diano, N., Crispi, S., & Mita, D. G. (2015). Bisphenol A effects on gene expression in adipocytes from children: association with metabolic disorders. *J Mol Endocrinol*, *54*(3), 289-303. <https://doi.org/10.1530/jme-14-0282>

Mitro, N., Vargas, L., Romeo, R., Koder, A., & Saez, E. (2007). T0901317 is a potent PXR ligand: implications for the biology ascribed to LXR. *FEBS Lett*, *581*(9), 1721-1726. <https://doi.org/10.1016/j.febslet.2007.03.047>

Mutt, S. J., Hyppönen, E., Saarnio, J., Järvelin, M.-R., & Herzig, K.-H. (2014). Vitamin D and adipose tissue—more than storage [Review]. *Frontiers in Physiology*, *5*. <https://doi.org/10.3389/fphys.2014.00228>

Nasab, H., Rajabi, S., Mirzaee, M., & Hashemi, M. (2022). Association of urinary triclosan, methyl triclosan, triclocarban, and 2,4-dichlorophenol levels with anthropometric and demographic parameters in children and adolescents in 2020 (case study: Kerman, Iran). *Environ Sci Pollut Res Int*, *29*(20), 30754-30763. <https://doi.org/10.1007/s11356-021-18466-3>

Neff, L. M., Culiner, J., Cunningham-Rundles, S., Seidman, C., Meehan, D., Maturi, J., Wittkowski, K. M., Levine, B., & Breslow, J. L. (2011). Algal docosahexaenoic acid affects plasma lipoprotein particle size distribution in overweight and obese adults. *J Nutr*, *141*(2), 207-213. <https://doi.org/10.3945/jn.110.130021>

Norgren, K., Tuck, A., Vieira Silva, A., Burkhardt, P., Öberg, M., & Munic Kos, V. (2022). High throughput screening of bisphenols and their mixtures under conditions of low-intensity adipogenesis of human mesenchymal stem cells (hMSCs). *Food Chem Toxicol*, *161*, 112842. <https://doi.org/10.1016/j.fct.2022.112842>

Oliver, M. (2012). The clofibrate saga: a retrospective commentary. *Br J Clin Pharmacol*, *74*(6), 907-910. <https://doi.org/10.1111/j.1365-2125.2012.04282.x>

Pannu, P. K., Zhao, Y., & Soares, M. J. (2016). Reductions in body weight and percent fat mass increase the vitamin D status of obese subjects: a systematic review and metaregression analysis. *Nutr Res*, *36*(3), 201-213. <https://doi.org/10.1016/j.nutres.2015.11.013>

Park, C. Y., & Han, S. N. (2021). The Role of Vitamin D in Adipose Tissue Biology: Adipocyte Differentiation, Energy Metabolism, and Inflammation. *J Lipid Atheroscler*, *10*(2), 130-144. <https://doi.org/10.12997/jla.2021.10.2.130>

Pereira-Fernandes, A., Demaegdt, H., Vandermeiren, K., Hectors, T. L., Jorens, P. G., Blust, R., & Vanparys, C. (2013). Evaluation of a screening system for obesogenic compounds: screening of endocrine disrupting compounds and evaluation of the PPAR dependency of the effect. *PLoS One*, *8*(10), e77481. <https://doi.org/10.1371/journal.pone.0077481>

Pereira-Fernandes, A., Vanparys, C., Vergauwen, L., Knapen, D., Jorens, P. G., & Blust, R. (2014). Toxicogenomics in the 3T3-L1 cell line, a new approach for screening of obesogenic compounds. *Toxicol Sci*, *140*(2), 352-363. <https://doi.org/10.1093/toxsci/kfu092>

Persistent Organic Pollutants Review Committee (POPRC). (2022). *Call for information and follow-up to the eighteenth meeting of the Persistent Organic Pollutants Review Committee*. Retrieved 12 December from <https://www.pops.int/TheConvention/POPsReviewCommittee/Meetings/POPRC18/POPRC18Followup/tabid/9352/Default.aspx>

Peshdary, V., & Atlas, E. (2018). Dexamethasone induced miR-155 up-regulation in differentiating 3T3-L1 preadipocytes does not affect adipogenesis. *Sci Rep*, *8*(1), 1264. <https://doi.org/10.1038/s41598-018-19704-4>

Peshdary, V., Calzadilla, G., Landry, A., Sorisky, A., & Atlas, E. (2019). Dechlorane Plus increases adipogenesis in 3T3-L1 and human primary preadipocytes independent of peroxisome proliferator-activated receptor γ transcriptional activity. *Int J Obes (Lond)*, *43*(3), 545-555. <https://doi.org/10.1038/s41366-018-0072-7>

Pesta, M., Cedikova, M., Dvorak, P., Dvorakova, J., Kulda, V., Srbecka, K., Muller, L., Bouchalova, V., Kralickova, M., Babuska, V., Kuncova, J., & Mullerova, D. (2018). Trends in gene expression changes during adipogenesis in human adipose derived mesenchymal stem cells under dichlorodiphenyldichloroethylene exposure. *Molecular & Cellular Toxicology*, *14*(4), 369-379. <https://doi.org/10.1007/s13273-018-0041-1>

Pomatto, V., Cottone, E., Cocci, P., Mozzicafreddo, M., Mosconi, G., Nelson, E. R., Palermo, F. A., & Bovolin, P. (2018). Plasticizers used in food-contact materials affect adipogenesis in 3T3-L1 cells. *J Steroid Biochem Mol Biol*, *178*, 322-332. <https://doi.org/10.1016/j.jsbmb.2018.01.014>

Prueitt, R. L., Goodman, J. E., Bailey, L. A., & Rhomberg, L. R. (2011). Hypothesis-based weight-of-evidence evaluation of the neurodevelopmental effects of chlorpyrifos. *Critical Reviews in Toxicology*, *41*(10), 822-903. <https://doi.org/10.3109/10408444.2011.616877>

Regnault, C., Usal, M., Veyrenc, S., Couturier, K., Batandier, C., Bulteau, A. L., Lejon, D., Sapin, A., Combourieu, B., Chetiveaux, M., Le May, C., Lafond, T., Raveton, M., & Reynaud, S. (2018). Unexpected metabolic disorders induced by endocrine disruptors in Xenopus tropicalis provide new lead for understanding amphibian decline. *Proc Natl Acad Sci U S A*, *115*(19), E4416-e4425. <https://doi.org/10.1073/pnas.1721267115>

Ren, X. M., Chang, R. C., Huang, Y., Amorim Amato, A., Carivenc, C., Grimaldi, M., Kuo, Y., Balaguer, P., Bourguet, W., & Blumberg, B. (2023). 2,4-Di-tert-butylphenol Induces Adipogenesis in Human Mesenchymal Stem Cells by Activating Retinoid X Receptors. *Endocrinology*, *164*(4). <https://doi.org/10.1210/endocr/bqad021>

Report of the Committee of Principal Investigators. (1984). WHO cooperative trial on primary prevention of ischaemic heart disease with clofibrate to lower serum cholesterol: final mortality follow-up. . *Lancet*, *2*(8403), 600-604.

Riu, A., Grimaldi, M., le Maire, A., Bey, G., Phillips, K., Boulahtouf, A., Perdu, E., Zalko, D., Bourguet, W., & Balaguer, P. (2011). Peroxisome proliferator-activated receptor γ is a target for halogenated analogs of bisphenol A. *Environ Health Perspect*, *119*(9), 1227-1232. <https://doi.org/10.1289/ehp.1003328>

Riu, A., McCollum, C. W., Pinto, C. L., Grimaldi, M., Hillenweck, A., Perdu, E., Zalko, D., Bernard, L., Laudet, V., Balaguer, P., Bondesson, M., & Gustafsson, J. A. (2014). Halogenated bisphenol-A analogs act as obesogens in zebrafish larvae (Danio rerio). *Toxicol Sci*, *139*(1), 48-58. <https://doi.org/10.1093/toxsci/kfu036>

Rock, C. L., Emond, J. A., Flatt, S. W., Heath, D. D., Karanja, N., Pakiz, B., Sherwood, N. E., & Thomson, C. A. (2012). Weight loss is associated with increased serum 25-hydroxyvitamin D in overweight or obese women. *Obesity (Silver Spring)*, *20*(11), 2296-2301. <https://doi.org/10.1038/oby.2012.57>

Rolle-Kampczyk, U., Gebauer, S., Haange, S. B., Schubert, K., Kern, M., Moulla, Y., Dietrich, A., Schön, M. R., Klöting, N., von Bergen, M., & Blüher, M. (2020). Accumulation of distinct persistent organic pollutants is associated with adipose tissue inflammation. *Sci Total Environ*, *748*, 142458. <https://doi.org/10.1016/j.scitotenv.2020.142458>

Routti, H., Berg, M. K., Lille-Langøy, R., Øygarden, L., Harju, M., Dietz, R., Sonne, C., & Goksøyr, A. (2019). Environmental contaminants modulate the transcriptional activity of polar bear (Ursus maritimus) and human peroxisome proliferator-activated receptor alpha (PPARA). *Scientific Reports*, *9*(1), 6918. <https://doi.org/10.1038/s41598-019-43337-w>

Salehpour, A., Hedayati, M., Shidfar, F., Neshatbini Tehrani, A., Farshad, A. A., & Mohammadi, S. (2021). 1,25-Dihydroxyvitamin D3 modulates adipogenesis of human adipose-derived mesenchymal stem cells dose-dependently. *Nutr Metab (Lond)*, *18*(1), 29. <https://doi.org/10.1186/s12986-021-00561-4>

Salehpour, A., Hosseinpanah, F., Shidfar, F., Vafa, M., Razaghi, M., Dehghani, S., Hoshiarrad, A., & Gohari, M. (2012). A 12-week double-blind randomized clinical trial of vitamin D₃ supplementation on body fat mass in healthy overweight and obese women. *Nutr J*, *11*, 78. <https://doi.org/10.1186/1475-2891-11-78>

Salihovic, S., Ganna, A., Fall, T., Broeckling, C. D., Prenni, J. E., van Bavel, B., Lind, P. M., Ingelsson, E., & Lind, L. (2016). The metabolic fingerprint of p,p′-DDE and HCB exposure in humans. *Environment International*, *88*, 60-66. <https://doi.org/https://doi.org/10.1016/j.envint.2015.12.015>

Santinha, D., Klopot, A., Marques, I., Ellis, E., Jorns, C., Johansson, H., Melo, T., Antonson, P., Jakobsson, T., Félix, V., Gustafsson, J., Domingues, M. R., Mode, A., & Helguero, L. A. (2020). Lipidomic analysis of human primary hepatocytes following LXR activation with GW3965 identifies AGXT2L1 as a main target associated to changes in phosphatidylethanolamine. *J Steroid Biochem Mol Biol*, *198*, 105558. <https://doi.org/10.1016/j.jsbmb.2019.105558>

Sargis, R. M., Johnson, D. N., Choudhury, R. A., & Brady, M. J. (2010). Environmental endocrine disruptors promote adipogenesis in the 3T3-L1 cell line through glucocorticoid receptor activation. *Obesity (Silver Spring)*, *18*(7), 1283-1288. <https://doi.org/10.1038/oby.2009.419>

Savastano, S., Tarantino, G., D'Esposito, V., Passaretti, F., Cabaro, S., Liotti, A., Liguoro, D., Perruolo, G., Ariemma, F., Finelli, C., Beguinot, F., Formisano, P., & Valentino, R. (2015). Bisphenol-A plasma levels are related to inflammatory markers, visceral obesity and insulin-resistance: a cross-sectional study on adult male population. *J Transl Med*, *13*, 169. <https://doi.org/10.1186/s12967-015-0532-y>

SCCP. (2009). *Scientific Committee on Consumer Products, Opinion on triclosan, 21 January 2009*. <https://ec.europa.eu/health/ph_risk/committees/04_sccp/docs/sccp_o_166.pdf>

Schaffert, A., Krieg, L., Weiner, J., Schlichting, R., Ueberham, E., Karkossa, I., Bauer, M., Landgraf, K., Junge, K. M., Wabitsch, M., Lehmann, J., Escher, B. I., Zenclussen, A. C., Körner, A., Blüher, M., Heiker, J. T., von Bergen, M., & Schubert, K. (2021). Alternatives for the worse: Molecular insights into adverse effects of bisphenol a and substitutes during human adipocyte differentiation. *Environ Int*, *156*, 106730. <https://doi.org/10.1016/j.envint.2021.106730>

Schierle, S., & Merk, D. (2019). Therapeutic modulation of retinoid X receptors - SAR and therapeutic potential of RXR ligands and recent patents. *Expert Opin Ther Pat*, *29*(8), 605-621. <https://doi.org/10.1080/13543776.2019.1643322>

Schluter, A., Giralt, M., Iglesias, R., & Villarroya, F. (2002). Phytanic acid, but not pristanic acid, mediates the positive effects of phytol derivatives on brown adipocyte differentiation. *FEBS Lett*, *517*(1-3), 83-86. <https://doi.org/10.1016/s0014-5793(02)02583-8>

Schlüter, A., Yubero, P., Iglesias, R., Giralt, M., & Villarroya, F. (2002). The chlorophyll-derived metabolite phytanic acid induces white adipocyte differentiation. *Int J Obes Relat Metab Disord*, *26*(9), 1277-1280. <https://doi.org/10.1038/sj.ijo.0802068>

Seimandi, M., Lemaire, G., Pillon, A., Perrin, A., Carlavan, I., Voegel, J. J., Vignon, F., Nicolas, J.-C., & Balaguer, P. (2005). Differential responses of PPARα, PPARδ, and PPARγ reporter cell lines to selective PPAR synthetic ligands. *Analytical Biochemistry*, *344*(1), 8-15. <https://doi.org/https://doi.org/10.1016/j.ab.2005.06.010>

Shankar, A., Teppala, S., & Sabanayagam, C. (2012). Urinary bisphenol a levels and measures of obesity: results from the national health and nutrition examination survey 2003-2008. *ISRN Endocrinol*, *2012*, 965243. <https://doi.org/10.5402/2012/965243>

Sharma, S., Ahmad, S., Khan, M. F., Parvez, S., & Raisuddin, S. (2018). In silico molecular interaction of bisphenol analogues with human nuclear receptors reveals their stronger affinity vs. classical bisphenol A. *Toxicol Mech Methods*, *28*(9), 660-669. <https://doi.org/10.1080/15376516.2018.1491663>

Shoucri, B. M., Martinez, E. S., Abreo, T. J., Hung, V. T., Moosova, Z., Shioda, T., & Blumberg, B. (2017). Retinoid X Receptor Activation Alters the Chromatin Landscape To Commit Mesenchymal Stem Cells to the Adipose Lineage. *Endocrinology*, *158*(10), 3109-3125. <https://doi.org/10.1210/en.2017-00348>

Sneve, M., Figenschau, Y., & Jorde, R. (2008). Supplementation with cholecalciferol does not result in weight reduction in overweight and obese subjects. *Eur J Endocrinol*, *159*(6), 675-684. <https://doi.org/10.1530/eje-08-0339>

Sol, C. M., Santos, S., Duijts, L., Asimakopoulos, A. G., Martinez-Moral, M. P., Kannan, K., Philips, E. M., Trasande, L., & Jaddoe, V. W. V. (2020). Fetal exposure to phthalates and bisphenols and childhood general and organ fat. A population-based prospective cohort study. *Int J Obes (Lond)*, *44*(11), 2225-2235. <https://doi.org/10.1038/s41366-020-00672-7>

Spratlen, M. J., Perera, F. P., Lederman, S. A., Robinson, M., Kannan, K., Herbstman, J., & Trasande, L. (2020). The Association Between Perfluoroalkyl Substances and Lipids in Cord Blood. *J Clin Endocrinol Metab*, *105*(1), 43-54. <https://doi.org/10.1210/clinem/dgz024>

Staels, B., Dallongeville, J., Auwerx, J., Schoonjans, K., Leitersdorf, E., & Fruchart, J. C. (1998). Mechanism of action of fibrates on lipid and lipoprotein metabolism. *Circulation*, *98*(19), 2088-2093. <https://doi.org/10.1161/01.cir.98.19.2088>

Sugeng, E. J., Symeonides, C., O'Hely, M., Vuillermin, P., Sly, P. D., Vijayasarathy, S., Thompson, K., Pezic, A., Mueller, J. F., & Ponsonby, A. L. (2020). Predictors with regard to ingestion, inhalation and dermal absorption of estimated phthalate daily intakes in pregnant women: The Barwon infant study. *Environ Int*, *139*, 105700. <https://doi.org/10.1016/j.envint.2020.105700>

Szymczak-Pajor, I., Miazek, K., Selmi, A., Balcerczyk, A., & Śliwińska, A. (2022). The Action of Vitamin D in Adipose Tissue: Is There the Link between Vitamin D Deficiency and Adipose Tissue-Related Metabolic Disorders? *Int J Mol Sci*, *23*(2). <https://doi.org/10.3390/ijms23020956>

Taxvig, C., Dreisig, K., Boberg, J., Nellemann, C., Schelde, A. B., Pedersen, D., Boergesen, M., Mandrup, S., & Vinggaard, A. M. (2012). Differential effects of environmental chemicals and food contaminants on adipogenesis, biomarker release and PPARγ activation. *Mol Cell Endocrinol*, *361*(1-2), 106-115. <https://doi.org/10.1016/j.mce.2012.03.021>

Teppala, S., Madhavan, S., & Shankar, A. (2012). Bisphenol A and Metabolic Syndrome: Results from NHANES. *Int J Endocrinol*, *2012*, 598180. <https://doi.org/10.1155/2012/598180>

Tomlinson, J. J., Boudreau, A., Wu, D., Abdou Salem, H., Carrigan, A., Gagnon, A., Mears, A. J., Sorisky, A., Atlas, E., & Haché, R. J. (2010). Insulin sensitization of human preadipocytes through glucocorticoid hormone induction of forkhead transcription factors. *Mol Endocrinol*, *24*(1), 104-113. <https://doi.org/10.1210/me.2009-0091>

Tomlinson, J. J., Boudreau, A. l., Wu, D., Atlas, E., & Haché, R. J. G. (2006). Modulation of Early Human Preadipocyte Differentiation by Glucocorticoids. *Endocrinology*, *147*(11), 5284-5293. <https://doi.org/10.1210/en.2006-0267>

Trasande, L., Attina, T. M., Sathyanarayana, S., Spanier, A. J., & Blustein, J. (2013). Race/ethnicity-specific associations of urinary phthalates with childhood body mass in a nationally representative sample. *Environ Health Perspect*, *121*(4), 501-506. <https://doi.org/10.1289/ehp.1205526>

Trunnelle, K. J., Bennett, D. H., Tulve, N. S., Clifton, M. S., Davis, M. D., Calafat, A. M., Moran, R., Tancredi, D. J., & Hertz-Picciotto, I. (2014). Urinary pyrethroid and chlorpyrifos metabolite concentrations in Northern California families and their relationship to indoor residential insecticide levels, part of the Study of Use of Products and Exposure Related Behavior (SUPERB). *Environ Sci Technol*, *48*(3), 1931-1939. <https://doi.org/10.1021/es403661a>

Tung, E. W., Boudreau, A., Wade, M. G., & Atlas, E. (2014). Induction of adipocyte differentiation by polybrominated diphenyl ethers (PBDEs) in 3T3-L1 cells. *PLoS One*, *9*(4), e94583. <https://doi.org/10.1371/journal.pone.0094583>

Tung, E. W. Y., Ahmed, S., Peshdary, V., & Atlas, E. (2017). Firemaster® 550 and its components isopropylated triphenyl phosphate and triphenyl phosphate enhance adipogenesis and transcriptional activity of peroxisome proliferator activated receptor (Pparγ) on the adipocyte protein 2 (aP2) promoter. *PLoS One*, *12*(4), e0175855. <https://doi.org/10.1371/journal.pone.0175855>

Uauy, R., Mena, P., & Rojas, C. (2000). Essential fatty acids in early life: structural and functional role. *Proc Nutr Soc*, *59*(1), 3-15. <https://doi.org/10.1017/s0029665100000021>

Valentino, R., D'Esposito, V., Passaretti, F., Liotti, A., Cabaro, S., Longo, M., Perruolo, G., Oriente, F., Beguinot, F., & Formisano, P. (2013). Bisphenol-A impairs insulin action and up-regulates inflammatory pathways in human subcutaneous adipocytes and 3T3-L1 cells. *PLoS One*, *8*(12), e82099. <https://doi.org/10.1371/journal.pone.0082099>

Vannice, G., & Rasmussen, H. (2014). Position of the academy of nutrition and dietetics: dietary fatty acids for healthy adults. *J Acad Nutr Diet*, *114*(1), 136-153. <https://doi.org/10.1016/j.jand.2013.11.001>

Veiga-Lopez, A., Kannan, K., Liao, C., Ye, W., Domino, S. E., & Padmanabhan, V. (2015). Gender-Specific Effects on Gestational Length and Birth Weight by Early Pregnancy BPA Exposure. *J Clin Endocrinol Metab*, *100*(11), E1394-1403. <https://doi.org/10.1210/jc.2015-1724>

Verbanck, M., Canouil, M., Leloire, A., Dhennin, V., Coumoul, X., Yengo, L., Froguel, P., & Poulain-Godefroy, O. (2017). Low-dose exposure to bisphenols A, F and S of human primary adipocyte impacts coding and non-coding RNA profiles. *PLoS One*, *12*(6), e0179583. <https://doi.org/10.1371/journal.pone.0179583>

Wada, K., Sakamoto, H., Nishikawa, K., Sakuma, S., Nakajima, A., Fujimoto, Y., & Kamisaki, Y. (2007). Life style-related diseases of the digestive system: endocrine disruptors stimulate lipid accumulation in target cells related to metabolic syndrome. *J Pharmacol Sci*, *105*(2), 133-137. <https://doi.org/10.1254/jphs.fm0070034>

Waits, A., Chen, H. C., Kuo, P. L., Wang, C. W., Huang, H. B., Chang, W. H., Shih, S. F., & Huang, P. C. (2020). Urinary phthalate metabolites are associated with biomarkers of DNA damage and lipid peroxidation in pregnant women - Tainan Birth Cohort Study (TBCS). *Environ Res*, *188*, 109863. <https://doi.org/10.1016/j.envres.2020.109863>

Wang, J., Bi, W., Zhao, W., Varghese, M., Koch, R. J., Walker, R. H., Chandraratna, R. A., Sanders, M. E., Janesick, A., Blumberg, B., Ward, L., Ho, L., & Pasinetti, G. M. (2016). Selective brain penetrable Nurr1 transactivator for treating Parkinson's disease. *Oncotarget*, *7*(7), 7469-7479. <https://doi.org/10.18632/oncotarget.7191>

Wang, L., Asimakopoulos, A. G., & Kannan, K. (2015). Accumulation of 19 environmental phenolic and xenobiotic heterocyclic aromatic compounds in human adipose tissue. *Environ Int*, *78*, 45-50. <https://doi.org/10.1016/j.envint.2015.02.015>

Wang, W. L., Welsh, J., & Tenniswood, M. (2013). 1,25-Dihydroxyvitamin D3 modulates lipid metabolism in prostate cancer cells through miRNA mediated regulation of PPARA. *J Steroid Biochem Mol Biol*, *136*, 247-251. <https://doi.org/10.1016/j.jsbmb.2012.09.033>

Wassenaar, P. N. H., & Legler, J. (2017). Systematic review and meta-analysis of early life exposure to di(2-ethylhexyl) phthalate and obesity related outcomes in rodents. *Chemosphere*, *188*, 174-181. <https://doi.org/https://doi.org/10.1016/j.chemosphere.2017.08.165>

Watkins, A. M., Wood, C. R., Lin, M. T., & Abbott, B. D. (2015). The effects of perfluorinated chemicals on adipocyte differentiation in vitro. *Mol Cell Endocrinol*, *400*, 90-101. <https://doi.org/10.1016/j.mce.2014.10.020>

Werner, L. B., Hellgren, L. I., Raff, M., Jensen, S. K., Petersen, R. A., Drachmann, T., & Tholstrup, T. (2011). Effect of dairy fat on plasma phytanic acid in healthy volunteers - a randomized controlled study. *Lipids in Health and Disease*, *10*(1), 95. <https://doi.org/10.1186/1476-511X-10-95>

WHO. (2009). *FAO Specifications and Evaluations for Agricultural Pesticides. Chlorpyrifos. O,O-diethyl O-3,5,6-trichloro-2-pyridyl phosphorothioate*. WHO Food and Agriculture Organization of the United Nations. <https://www.fao.org/3/i1277e/i1277e.pdf>

Wu, Y., Wu, Q., Beland, F. A., Ge, P., Manjanatha, M. G., & Fang, J. L. (2014). Differential effects of triclosan on the activation of mouse and human peroxisome proliferator-activated receptor alpha. *Toxicol Lett*, *231*(1), 17-28. <https://doi.org/10.1016/j.toxlet.2014.09.001>

Yaghjyan, L., Sites, S., Ruan, Y., & Chang, S. H. (2015). Associations of urinary phthalates with body mass index, waist circumference and serum lipids among females: National Health and Nutrition Examination Survey 1999-2004. *Int J Obes (Lond)*, *39*(6), 994-1000. <https://doi.org/10.1038/ijo.2015.8>

Yokoyama, Y., Xin, B., Shigeto, T., Umemoto, M., Kasai-Sakamoto, A., Futagami, M., Tsuchida, S., Al-Mulla, F., & Mizunuma, H. (2007). Clofibric acid, a peroxisome proliferator-activated receptor alpha ligand, inhibits growth of human ovarian cancer. *Mol Cancer Ther*, *6*(4), 1379-1386. <https://doi.org/10.1158/1535-7163.Mct-06-0722>

Yueh, M.-F., He, F., Chen, C., Vu, C., Tripathi, A., Knight, R., Karin, M., Chen, S., & Tukey, R. H. (2020). Triclosan leads to dysregulation of the metabolic regulator FGF21 exacerbating high fat diet-induced nonalcoholic fatty liver disease. *Proceedings of the National Academy of Sciences*, *117*(49), 31259-31266. <https://doi.org/10.1073/pnas.2017129117>

Zamora, A. N., Jansen, E. C., Tamayo-Ortiz, M., Goodrich, J. M., Sánchez, B. N., Watkins, D. J., Tamayo-Orozco, J. A., Téllez-Rojo, M. M., Mercado-García, A., Baylin, A., Meeker, J. D., & Peterson, K. E. (2021). Exposure to Phenols, Phthalates, and Parabens and Development of Metabolic Syndrome Among Mexican Women in Midlife. *Front Public Health*, *9*, 620769. <https://doi.org/10.3389/fpubh.2021.620769>

Zhang, F., Cao, R. L., Liu, P., Chi, T. Y., Ji, X. F., Zheng, Z. H., Chen, G. L., & Zou, L. B. (2023). The bexarotene derivative OAB-14 ameliorates cognitive decline in APP/PS1 transgenic mice by suppressing microglia-mediated neuroinflammation through the PPAR-γ pathway. *Int Immunopharmacol*, *124*(Pt A), 110911. <https://doi.org/10.1016/j.intimp.2023.110911>

Zheng, F., Zhang, S., Lu, W., Wu, F., Yin, X., Yu, D., Pan, Q., & Li, H. (2014). Regulation of insulin resistance and adiponectin signaling in adipose tissue by liver X receptor activation highlights a cross-talk with PPARγ. *PLoS One*, *9*(6), e101269. <https://doi.org/10.1371/journal.pone.0101269>

Zoete, V., Grosdidier, A., & Michielin, O. (2007). Peroxisome proliferator-activated receptor structures: ligand specificity, molecular switch and interactions with regulators. *Biochim Biophys Acta*, *1771*(8), 915-925. <https://doi.org/10.1016/j.bbalip.2007.01.007>

Zoico, E., Franceschetti, G., Chirumbolo, S., Rossi, A. P., Mazzali, G., Rizzatti, V., Budui, S., & Zamboni, M. (2014). Phenotypic Shift of Adipocytes by Cholecalciferol and 1α,25 Dihydroxycholecalciferol in Relation to Inflammatory Status and Calcium Content. *Endocrinology*, *155*(11), 4178-4188. <https://doi.org/10.1210/en.2013-1969>

Zomer, A. W., van Der Burg, B., Jansen, G. A., Wanders, R. J., Poll-The, B. T., & van Der Saag, P. T. (2000). Pristanic acid and phytanic acid: naturally occurring ligands for the nuclear receptor peroxisome proliferator-activated receptor alpha. *J Lipid Res*, *41*(11), 1801-1807.
